# One‐Pot Synthesis of Chiral Succinate Dehydrogenase Inhibitors and Antifungal Activity Studies

**DOI:** 10.1002/advs.202416250

**Published:** 2025-05-19

**Authors:** Donghua Du, Yu Chen, Chengbing Yang, Zheng Jin, Huailong Teng

**Affiliations:** ^1^ College of Chemistry Huazhong Agricultural University Wuhan 430070 P. R. China; ^2^ Department of Chemical and Environmental Engineering The University of Nottingham Ningbo China Ningbo 315100 P. R. China

**Keywords:** antifungal activity, asymmetric hydrogenation, chiral fungicides, succinate dehydrogenase inhibitors

## Abstract

In this work, a series of novel chiral succinate dehydrogenase inhibitors (SDHIs) are synthesized through a one‐pot Rh‐catalyzed asymmetric hydrogenation‐condensation strategy. This method exhibits high efficiency (up to 1000 Ton, 94% yield over two steps), high stereoselectivity (up to 99% ee), and broad substrate scope (68 examples in total), providing a superior pathway for the synthesis of such chiral fungicides. Mechanistic studies indicate that the amino group at the 2‐position of the phenyl ring acts as an activating group, enhancing the reactivity and stereoselectivity control of the reaction. Furthermore, these molecules exhibit broad‐spectrum and highly effective antifungal biological activity. Notably, enantiomers show significant differences in both in vitro and in vivo fungi‐inhibiting experiments. Especially, (*S*)‐**5f** showcases an antifungal activity against *Botrytis cinerea* (EC_50_ = 0.48 µm) that is much higher than that of its *R* enantiomer (EC_50_ = 36.7 µm). Molecular docking calculations, molecular dynamic simulation, enzyme activity assays, and ligand‐target interaction experiments demonstrate that (*S*)‐**5f** (ΔG_MM‐PBSA_ = −18.86 kcal mol^−1^, K_D_ = 6.04 µm) inhibits succinate dehydrogenase more effectively than its *R* enantiomer (ΔG_MM‐PBSA_ = −13.01 kcal mol^−1^, K_D_ = 8.5 µm). Moreover, the two enantiomers have significantly different effects on spore germination and the destruction of fungal phenotype.

## Graphical abstract

This work describes an efficient synthetic method for chiral succinate dehydrogenase inhibitors, showcasing their potent and broad‐spectrum antifungal properties. Notably, substantial differences in biological activity between the enantiomers have been observed, underscoring the pivotal role of stereochemistry in the development of fungicides.

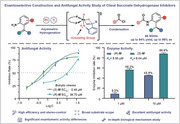

## 1 Introduction

**Scheme 1:**
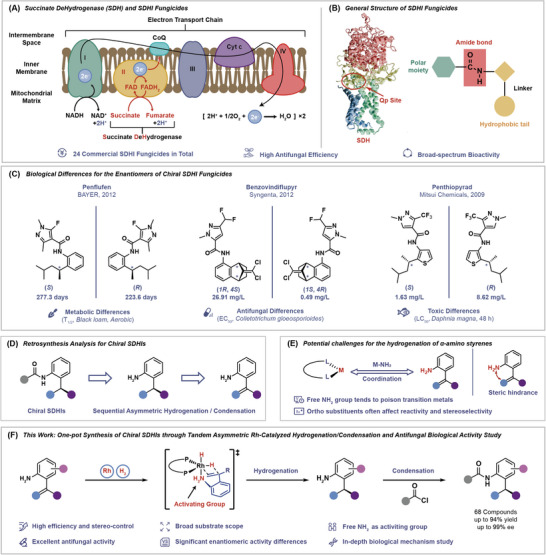
Synthetic strategy of chiral succinate dehydrogenase inhibitors.

Fungal diseases are widespread and diverse, accounting for more than 70% of crop diseases, which seriously threaten agricultural production.^[^ ^1^ ^]^ They can also produce toxins such as deoxynivalenol (DON) that can cause food safety concerns.^[^ ^2^ ^]^ Although chemical control is the main treatment method at present, the long‐term and widespread use of chemical pesticides has led to environmental pollution, food safety problems, and fungal resistance.^[^ ^3^ ^]^ Therefore, reducing the application of pesticides is a key issue in current agricultural production, and developing efficient and environmentally friendly antifungal molecules has always been an effective approach. In this respect, targeted pesticides have been a hot spot in the field of pesticide creation due to their precision, high efficiency, environmental protection, and low resistance risk characteristics.^[^ ^4^ ^]^ Particularly, succinate dehydrogenase inhibitors (SDHIs) are effective in fungal treatment by blocking fungal respiration (**Scheme** **1**A).^[^ ^5^ ^]^ The well‐defined structure of succinate dehydrogenase (SDH) facilitates the design of targeted inhibitors, and 24 related fungicides have been commercialized since the first SDHI (Carboxin) was marketed in 1964, the structure of such fungicides generally contains three parts: protein affinity groups, amide bond linkers, and hydrophobic tails. (Scheme 1B).^[^ ^6^ ^]^ To date, the development of succinate dehydrogenase inhibitors remains an extremely hot research field, G. Yang and other researchers have conducted extensive and in‐depth research in this area,^[^ ^7^ ^]^ and some chemists have paid attention to the study of chiral SDHIs.^[^ ^8^ ^]^ In fact, the use of chiral pesticides represents an effective method of reduced dose application, as enantiomers often exhibit significant differences in biological activity, toxicity, environmental behaviors, and metabolic kinetic properties.^[^ ^9^ ^]^ For examples of three SDHIs in Scheme 1C, (*S*)‐Penflufen has a longer half‐life (t_1/2_ = 277 d) than its *R* enantiomer (t_1/2_ = 223 d),^[^ ^10^ ^]^ while the biological activity of (1*S*, 4*R*)‐Benzovindiflupyr (EC_50_ = 0.49 mg L^−1^) against *Colleto‐trichum gloeosporiides* is much higher than that of (1*R*, 4*S*) enantiomer (EC_50_ = 26.91 mg L^−1^),^[^ ^11^ ^]^ and the toxicity of (*S*)‐Penthiopyrad (LC_50_ = 1.63 mg L^−1^) toward *Daphnia magna* is lower compared to its *R* configuration (LC_50_ = 8.62 mg L^−1^).^[^ ^12^ ^]^ Therefore, the development of chiral succinate dehydrogenase inhibitors has the potential to reduce the required dose of pesticides, thereby improving environmental and food safety.

On the other hand, developing efficient and low‐cost asymmetric synthetic methods for chiral fragments containing amino group (NH_2_) is a key step for chiral SDHIs creation. In the past few decades, asymmetric catalysis has long been a research hotspot, and numerous asymmetric catalytic synthesis methods have been developed.^[^ ^13^, ^14^ ^]^ Among them, asymmetric hydrogenation of olefins has been widely used in scientific research and industry due to its advantages of cleanliness, high efficiency, and low cost.^[^ ^15^, ^16^ ^]^ In theory, asymmetric hydrogenation of 2‐aminostilbene compounds could access chiral analine intermediates, which were then grafted to protein affinity groups to obtain chiral succinate dehydrogenase inhibitors (Scheme 1D). However, this strategy possesses potential challenges: First, in most transition metal catalyzed organic reactions, the free amino group (NH_2_) can poison catalysts through strong coordination with transition metals, thereby reducing catalytic activity; Second, the adjacent substituents of the phenyl ring often affect reaction activity and stereoselectivity control due to the steric hindrance (Scheme 1E). So far, only a few asymmetric transition metal catalytic reactions with free amino group (NH_2_) have been reported.^[^ ^17^ ^]^ For example, X. Zhang and other researchers have reported a Rh‐catalyzed asymmetric hydrogenation reaction involving free enamines.^[^ ^18^ ^]^ B. Shi has achieved the construction of axially chiral amines through a Pd‐catalyzed process, utilizing free amines to direct atroposelective C‐H olefination.^[^ ^19^ ^]^ N. Jiao has developed an efficient Cu‐catalyzed azidation reaction for anilines, offering a new method for the synthesis of azides.^[^ ^20^ ^]^ Also, X. Luan has synthesized imine‐containing dibenzo[*b*,*d*]azepines by employing a Pd‐catalyzed [5+2] oxidative annulation of *o*‐arylanilines with alkynes, demonstrating a creative strategy for the formation of complex heterocyclic structures.^[^ ^21^ ^]^

In this work, we efficiently and stereoselectively constructed 2‐chiral aniline fragments through Rh‐catalyzed asymmetric hydrogenation of 2‐aminostilbenes and obtained a series of chiral SDHIs through a one‐pot strategy (Scheme 1F). This method features the advantages of high activity, excellent stereoselectivity, broad substrate applicability, and mild conditions. Control experiments showed that the free amino group acts as an activating group to promote catalytic progress and stereoselective control. Antifungal tests revealed that these compounds have broad‐spectrum and efficient antifungal activity, especially compounds **5f** gave the best biological activity, and (*S*)‐**5f** exhibited much higher activity than that of (*R*)‐**5f** and commercial varieties of Boscalid both in vivo and in vitro experiments. The antifungal mechanism studies prove that (*S*)‐**5f** inhibits fungal growth by inhibiting fungal spore germination, destructing fungal cell walls and membranes, and suppressing fungal mitochondrial respiration.

## 2 Results and Discussion

### 2.1 Reaction Condition Optimization

In the beginning, we selected 2‐(1‐Phenylethenyl)aniline **1a** as substrate and screened the reaction conditions for the asymmetric hydrogenation and the results are summarized in **Table** **1**. Using 1% Rh(NBD)_2_BF_4_ as metal salt, (1*R*, 1′*R*, 2*S*, 2′*S*)‐DuanPhos **L1** as chiral ligand, and 1,2‐Dichloroethane (DCE) as solvent, the reaction can be completed within 4 h under 50 atm H_2_ atmosphere at room temperature, with a yield of 99% and an ee value of 95% (Table 1, entry 1). When (*S*)‐BINAP **L2** was used as a chiral ligand, the yield and stereoselectivity of product **2a** were significantly reduced (83% yield, 57% ee) (Table 1, entry 2), while (*S*)‐DBTM‐Segphos **L3** provided perfect result, with the conversion of raw material equivalents to the target product (99% yield) and stereoselectivity of 98% ee (Table 1, entry 3). Next, we investigated the effects of other transition metals on the reaction. Pd(OAc)_2_ and [Ir(COD)Cl]_2_ combined with **L3** were able to facilitate the reaction (92% and 99% yield), but only the racemic form of **2a** was obtained (Table 1, entries 4–5). Although we have achieved good results, considering the solvent effect in asymmetric catalytic hydrogenation reactions, we investigated the influence of various commonly used solvents on the reactivity. As shown, solvents with different properties have a significant impact on the catalytic activity and stereoselectivity of the reaction (Table 1, entries 6–9). The non‐polar solvent toluene and the strongly coordinating solvent 1,4‐dioxane gave poor yield (29%, 21%) and stereoselectivities (67%, 66% ee) (Table 1, entries 6,8). The reaction cannot proceed at all in the strongly polar solvent acetonitrile (MeCN) (Table 1, entry 7), while protonic solvent isopropanol (*^i−^* PrOH) can promote the hydrogenation reaction, yielding desired product **2a** with 99% yield and 94% ee, respectively (Table 1, entry 9). When the hydrogen pressure was reduced to 25 atm, the yield and stereoselectivity of the reaction decreased to 25% and 78% (Table 1, entry 10). Based on these results, we finally determined the optimal reaction conditions for catalytic hydrogenation: 1 mol% Rh(NBD)_2_BF_4_ as a metal precursor, (*S*)‐DTBM‐Segphos **L3** as chiral ligand, DCE as a solvent, under 50 atm H_2,_ at room temperature.

**Table 1:** Optimization of reaction conditions.

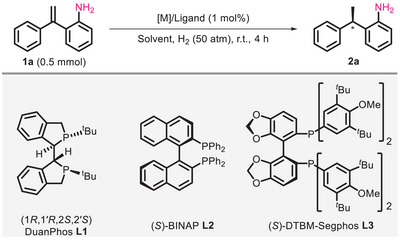					
Entry^a^ ^)^	[Metal]	Ligand	Solvent	Yield [%]^b^ ^)^	Ee [%]^c^ ^)^
1	Rh(NBD)_2_BF_4_	**L1**	DCE	99	95
2	Rh(NBD)_2_BF_4_	**L2**	DCE	83	57
3	Rh(NBD)_2_BF_4_	**L3**	DCE	99	98
4	Pd(OAc)_2_	**L3**	DCE	92	0
5	[Ir(COD)Cl]_2_	**L3**	DCE	99	0
6	Rh(NBD)_2_BF_4_	**L3**	Toluene	29	67
7	Rh(NBD)_2_BF_4_	**L3**	MeCN	trace	NA
8	Rh(NBD)_2_BF_4_	**L3**	1,4‐Dioxane	21	66
9	Rh(NBD)_2_BF_4_	**L3**	*^i−^* PrOH	99	94
10^d^ ^)^	Rh(NBD)_2_BF_4_	**L3**	DCE	25	78

^a)^ All reactions were carried out with 0.5 mmol of **1a** and 1 mol% of [M]/Ligand at room temperature under 50 atm H_2_ pressure for 4 h;

^b)^ Isolated yields;

^c)^ Determined by chiral HPLC analysis;

^d)^ 25 atm.

### 2.2 Substrate Scope Investigation

With the optimized reaction conditions in hand, we explored the substrate scope of the Rh/**L3**‐catalyzed asymmetric hydrogenation reaction, the results are summarized in **Table** **2**. In this part, intermediates **2** were transformed to chiral SDHI **3** through condensation with difluoropyrazolyl chloride using one pot strategy. The yields and ee values shown in Table 2 are given for the final product **3**. To facilitate the comparative biological activity investigation of enantiomers in the following studies, both enantiomers of chiral succinate dehydrogenase inhibitors were prepared by using (*S*) and (*R*)‐DTBM‐Segphos, and the following descriptions are given for the *S* enantiomer of products.

**Table 2:** Substrate scope investigation (see table footnote).

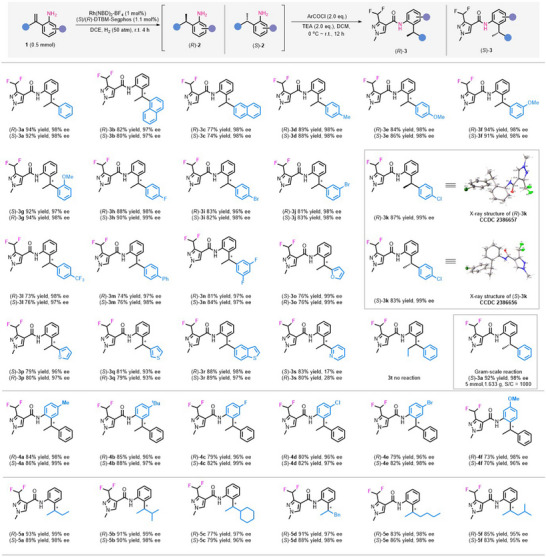

^a)^ All reactions were carried out with 0.5 mmol of **1** and 1 mol% of Rh/**L3** at room temperature under 50 atm H_2_ pressure for 4 h. Yields were given as isolated yields. Ee values were determined by chiral HPLC analysis.

First, we investigated the effect of aryl substituents on the reaction. This catalytic reaction system is suitable for various substituted substrates, both electron‐withdrawing and electron‐donating groups can participate in the reaction, providing the target products with high yields and stereoselectivities. The position of the substituent on the phenyl ring has little effect on the reaction (**3a**–**3g**), even with two *ortho* substituents on both phenyl rings, excellent results can still be obtained (**3g**, 92% yield, 97% ee). Halogens and heterocycles are important pharmacophores in drug molecules, and the introduction of these groups is crucial for enhancing biological activity. We are pleased that various halogen (F, Cl, Br) or halogen‐containing groups (CF_3_) can also be compatible with this catalytic system without the occurrence of dehalogenation (**3h**–**3l**, **3n**). Substrates containing heterocyclic compounds such as furan (**3o**) and thiophene (**3p**–**3r**) are also suitable for this reaction to access corresponding antifungal molecules with high yield (76%–89%) and stereoselectivity (93%–99% ee). Unfortunately, substrates substituted with 2‐pyridine can only provide extremely low ee values (**3s**, 17% ee) although with good yield (83% yield), which may be due to the strong coordination of pyridine with metals. Introducing methyl or phenyl groups **3t** at the end of double bonds, the reaction did not happen even under harsh conditions (80 °C). Since the aniline fragment is the linker of succinate dehydrogenase inhibitors, the substituents on this unit directly affect the binding mode between the antifungal molecules and the target protein pocket. Therefore, we investigated the substituents on aniline and found that both the electron properties and positions of the substituents on the phenyl ring had no obvious effect on the reactivity and stereoselectivity of the reaction (**4a**–**4f**). After two steps of reaction, the corresponding products were obtained in high yields (70%–88%) and with excellent stereoselectivities (96%–99% ee). Meanwhile, alkyl chains are often used as hydrophobic tails in many succinate dehydrogenase inhibitors, we also investigated the applicability of this catalytic system for alkyl‐substituted substrates. As shown, alkyl substituents such as ethyl (Et), isopropyl (*^i−^* Pr), cyclohexyl (Cy), benzyl (Bn), n‐butyl (*^n−^* Bu), and isobutyl (*^i−^* Bu) can all participate well in the reaction (**5a**–**5f**). The yield and ee value of other products remains at a high level (79%–90% yields, 95%–98% ee). The results of the substrate range investigation above indicate that the catalytic reaction system has wide applicability and potential application value. The absolute configuration of these products were determined by X‐ray analysis of (*R*)‐**3k** and (*S*)‐**3k**. (Note: The absolute configurations of **3g**, **3o**, **3p**, **3q**, and **3s** are opposite to those of other products due to the priority of the substituents at terminal groups). Also, to test the practicality of the synthetic method, we carried out a gram‐scale reaction (5 mmol) using 0.1 mol% of catalyst. The reaction proceeded smoothly and was completed within 72 h, yielding the desired product (*S*)‐**3a** with 92% yield and 98% ee.

**Figure 1:**
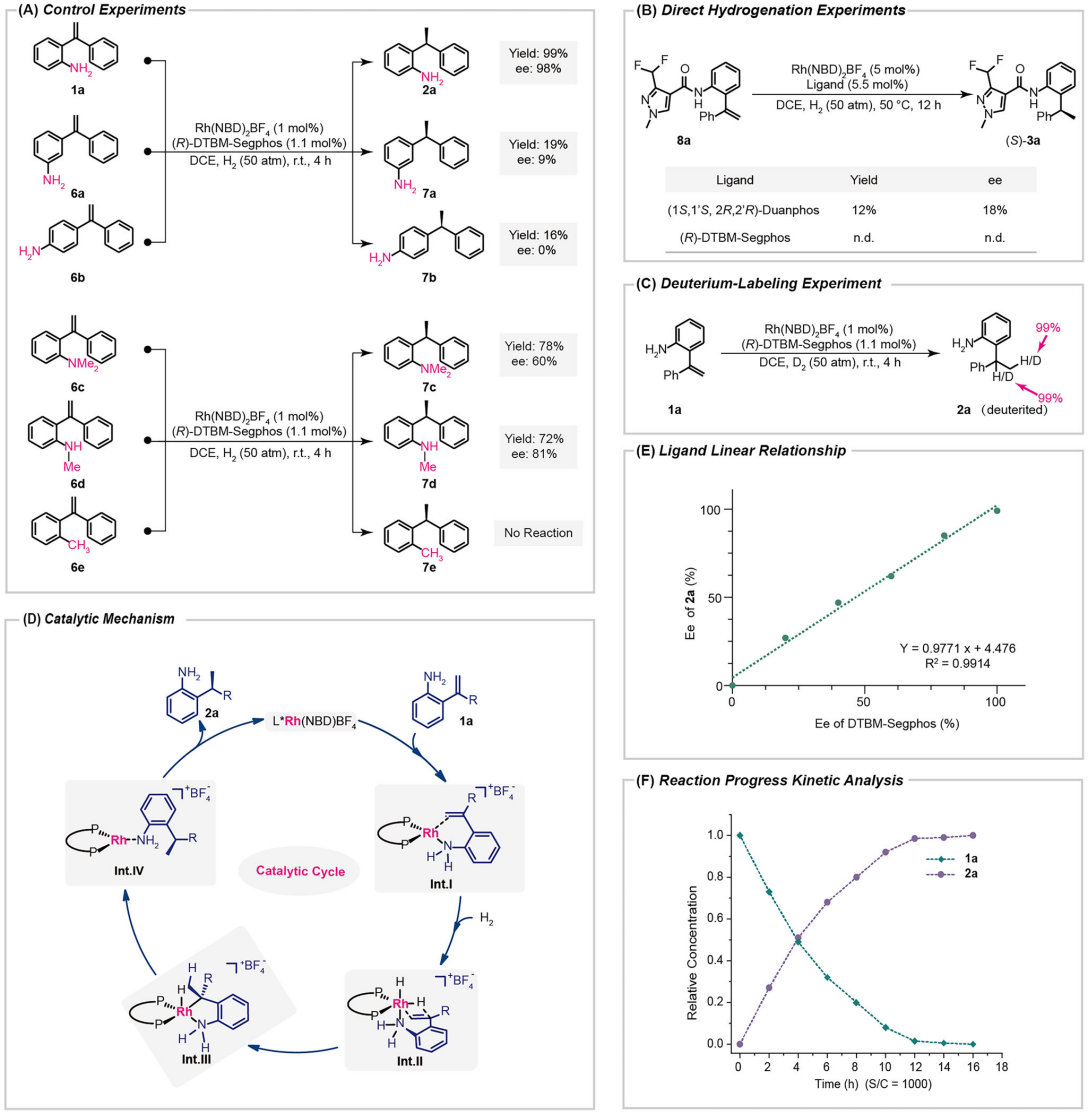
Mechanism studies.

To reveal the possible mechanism of the catalytic reaction and the effect of free amino groups on the catalytic process, we conducted a series of control experiments, as shown in **Figure** **1**A. The position of NH_2_ on the phenyl ring has a huge impact on the catalytic reaction. Under the same catalytic conditions, *ortho*‐NH_2_ substrate **1a** gave 99% yield and 98% ee value. However, when NH_2_ is located at the *meta* or *para* position, the activity and stereoselectivity of the reaction decrease significantly (**7a**, 19% yield, 9% ee; **7b**, 16% yield, 0% ee). These results indicated that the *ortho* amino group can act as an activating group through coordination to metal center together with a double bond and simultaneously promote the reaction. When the amino group is located at the *meta* or *para* position, it tends to compete with the olefin for coordination with the metal catalyst, thereby poisoning the metal catalyst and inhibiting the reaction. At the same time, when NH_2_ is protected by one (**6d**) or two methyl groups (**6c**), both catalytic activity and stereoselective control ability are greatly affected (**7c**, 78% yield, 60% ee; **7d**, 72% yield, 81% ee). Meanwhile, when the amino group was replaced with a methyl group (**6e**), the hydrogenation reaction could not proceed at all. This fully highlights the crucial role of free amino groups in the catalytic system. In addition, we also tried substrate **8a** which was formed by the condensation of difluoropyrazole carboxylic acid with 2‐aminostilbene, in which case, the desired product **3a** was obtained with poor results even with 5 mol% of Rh/DuanPhos as a catalyst at 50 °C for 12 h, only 12% yield, and 18% ee was obtained, while (*R*)‐DTBM‐Segphos could not make this reaction happen at all (Figure 1B). The deuteration experiment has unequivocally demonstrated that during the reaction process, the hydrogen atoms incorporated into the olefin originate exclusively from hydrogen gas and do not undergo any exchange with the hydrogen atoms present in the amino group (NH_2_) or the solvent (DCE) (Figure 1C). Based on the above mechanism verification experiments, a catalytic cycle as shown in Figure 1D was proposed. First, the metal center within the catalyst engages in a double coordination process with both the amino group and the olefinic moiety present in the substrate **1a** to form the intermediate **Int I**. Subsequently, this intermediate undergoes the reaction with hydrogen (H_2_), yielding the Rh‐H species **Int. II**. Further on, the C‐C double bond is inserted into the Rh─H bond via a transition state of a quaternary ring, leading to **Int. III**, which undergoes reduction elimination to form **Int. IV**, and decoordination to obtain the target product **2a**, while regenerating the catalyst. The nonlinear effect experiment showed a good linear relationship between the ee value of product **2a** and the ee value of the chiral ligand **L3**, indicating that in this catalytic system, the chiral ligand **L3** and metal Rh adopt a 1:1 coordination mode (Figure 1E). Finally, we investigated the kinetics of the reaction using the template reaction of **1a** with a catalyst dosage of 0.1 mol%, as shown in Figure 1F, throughout the catalytic reaction process, as the concentration of reactant **1a** decreased, the content of product **2a** increased until complete conversion was achieved.

### 2.3 Antifungal Activity Studies

**Figure 2:**
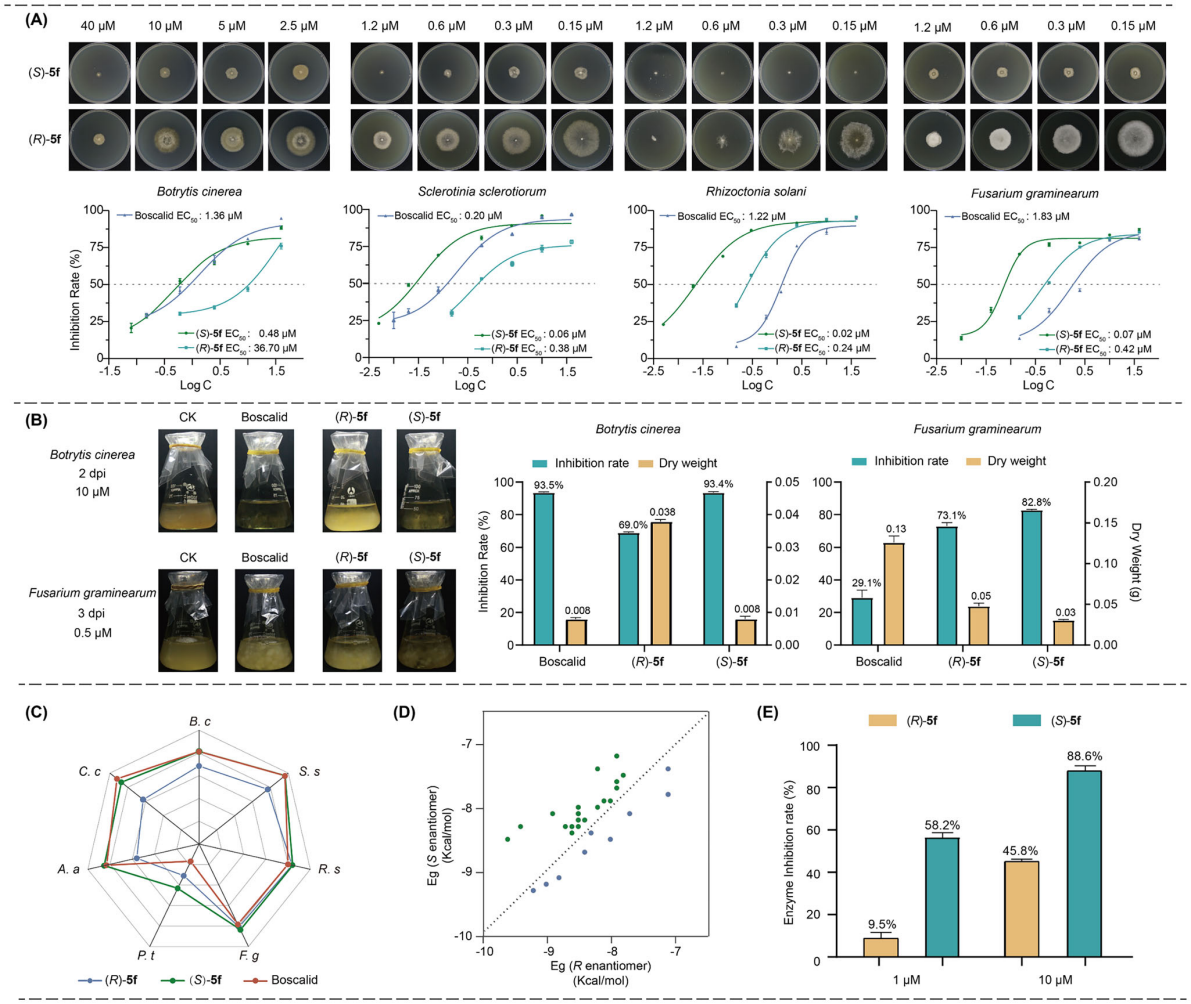
In vitro antifungal experiments. A) Inhibitory effects on *B. c*, *S. s*, *R. s*, *F. g*. B) Inhibition effects in liquid mycelium of *B. c* and *F. g*. C) Comparison of the inhibition effects of (*S*)─**5f** and (*R*)─**5f** at 20 µM toward seven common fungi. D) Binding energies of all enantiomers of SDHIs. E) Enzyme inhibition effectiveness.

With these chiral SDHIs in our hands, we next proceeded to investigate their antifungal activity.^[^ ^22^ ^]^ Initially, *Botrytis cinerea* (*B. c*) was chosen as the treating target, these compounds exhibited efficient and broad‐spectrum antifungal effects, and most compounds achieved inhibition rates of over 50% at a concentration of 20 µm (Table S1, Supporting Information). We selected four pairs of enantiomers (**5b**, **5c**, **5e**, **5f)** with significant differences in activity for EC_50_ testing (Table S3, Supporting Information). Under identical conditions, most of the *S*‐enantiomers demonstrated enhanced antifungal potency compared to their *R*‐configuration counterparts. Notably, compound **5f**, featuring an isobutyl group, stood out for its exceptional performance. As depicted in **Figure** **2**A, against *B. c*, the half‐effective inhibitory concentration (EC_50_) for (*S*)‐**5f** was an impressive 0.48 µm, significantly lower than that of the commercial fungicide Boscalid (EC_50_ = 1.36 µm) and its enantiomer (*R*)‐**5f** (EC_50_ = 36.70 µm). When tested against *Sclerotinia sclerotiorum* (*S. s*), the EC_50_ values were as follows: (*S*)‐**5f** (EC_50_ = 0.06 µm), Boscalid (EC_50_ = 0.20 µm), and (*R*)‐**5f** (EC_50_ = 0.38 µm). It is noteworthy that compounds **5f** exhibited superior inhibitory effects on rice *Rhizoctonia solani* (*R. s*) and wheat *Fusarium graminearum* (*F. g*) even at minimal concentrations, and (*S*)‐**5f** showed markedly greater inhibition than (*R*)‐**5f**. In the case of *Rhizoctonia solani*, (*S*)‐**5f** achieved a half inhibitory concentration of 0.02 µm, which is 12 times lower than that of (*R*)‐**5f**. Furthermore, the *S*‐enantiomer displayed a 6‐fold increase in activity against *Fusarium graminearum* compared to the *R*‐enantiomer, highlighting the significant impact of stereochemistry on the antifungal activity of these compounds. It not only highlights the importance of stereochemistry in determining the efficacy of these compounds but also opens up the prospect of utilizing a single enantiomer in the development of more targeted and potentially more environmentally friendly pesticides.

In addition, we delved into the activity disparities of enantiomers within a liquid‐phase system, as illustrated in Figure 2B. Our experiments involved cultivating *Botrytis cinerea* in a solution containing 10 µm of enantiomers of **5f**. The results revealed striking differences in the mycelial growth of the fungus, underscoring the significant impact of enantiomeric configuration on antifungal potency. Samples treated with Boscalid and the (*S*)‐**5f** displayed virtually no mycelial growth, indicating a potent antifungal effect. In stark contrast, the sample treated with the (*R*)‐enantiomer showed significantly more mycelial growth. This observation suggests that the (*R*)‐enantiomer was less effective against *Botrytis cinerea*. The inhibition rate, as determined by the dry weight measurements of the mycelium, revealed that both the (*S*)‐**5f** and Boscalid demonstrated comparable high levels of inhibition, ≈93.5%. While the (*R*)‐**5f** showed a somewhat lower inhibition rate of 69%, suggesting that while it still provided significant antifungal activity, it was less potent than its (*S*)‐**5f** and Boscalid. Similar results were also observed in the case of *Fusarium graminearum*, after 3 days of cultivation at a concentration of 0.5 µm, (*S*)‐**5f** delivered an inhibition rate of 82%, while Boscalid and (*R*)‐**5f** provided lower inhibition rates of 29% and 73%, respectively. Furthermore, we conducted comprehensive antifungal experiments across a spectrum of fungi, as detailed in Figure 2C. The results from seven distinct assays demonstrated that (*S*)‐**5f** significantly outperformed (*R*)‐**5f** in inhibiting the growth of *Botrytis cinerea* (*B. c*)*, Sclerotinia sclerotiorum* (*S. s*)*, Rhizoctonia solani* (*R. s*)*, Fusarium graminearum* (*F. g*)*, Pestalotiopsis tea* (*P. t*)*, Alternaria alternata* (*A. a*) *and Corynespora cassiicola* (*C. c*). This suggests that (*S*)‐**5f** has a broad‐spectrum efficacy against these specific fungal strains, which is a promising attribute for potential antifungal agents. Molecular docking calculations were performed to assess the interaction between these compounds and succinate dehydrogenase (SDH). The binding energies for all enantiomers of the compounds were determined (Figure 2D), revealing a consistent trend: in most cases, the *S*‐enantiomers exhibited stronger binding affinity to SDH, as indicated by higher binding energies, compared to their *R*‐enantiomers. This observation aligns well with the antifungal activity data, suggesting that the enhanced binding of the *S*‐enantiomers to SDH may contribute to their increased potency against fungi. Furthermore, enzyme activity assays provided compelling evidence of the distinct inhibitory effects of the enantiomers. At a concentration of 1 µm, the (*S*)‐enantiomer of compound **5f** demonstrated a substantial 58.2% inhibition rate on the target protein, whereas the (*R*)‐**5f** showed a significantly lower inhibition rate of only 9.5%. This pronounced difference was further accentuated at a higher concentration of 10 µm, with the (*S*)‐enantiomer achieving an 88.6% inhibition rate and the (*R*)‐enantiomer only reaching 45.8% (Figure 2E). These results offer a clear biochemical explanation for the observed differences in antifungal activity between the enantiomers.

Additionally, we delved into the impact of protein affinity groups on antifungal activity. As shown in **Table** **3**, we combined the chiral amine fragment of (*S*)‐**5f** with four common SDHI carboxylic acid fragments to successfully synthesize five chiral succinate dehydrogenase inhibitors (*S*)‐**9a** to (*S*)‐**9e**. By comparing with (*S*)‐**5f** in an experiment at a concentration of 1 µm, we assessed the inhibitory effects on four types of pathogenic fungi. The results indicated that aside from (*S*)‐**9a** showing comparable efficacy to (*S*)‐**5f** against *S.s*, (*S*)‐**5f** outperformed the other five compounds in inhibiting the other fungi. This finding highlights the significant advantage of difluoropyrazole carboxylic acid as a succinate dehydrogenase inhibitor.

**Table 3:** Influence of protein affinity groups on antifungal activity.

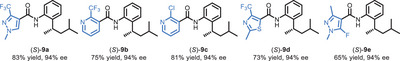				
SDHIs [1.0 µm]	Inhibition rate [%]			
	*B. c*	*S. s*	*R. s*	*F. g*
(*S*)‐**5f**	58.65	86.65	88.23	77.30
(*S*)‐**9a**	45.08	90.66	74.34	76.87
(*S*)‐**9b**	27.41	77.86	69.73	75.83
(*S*)‐**9c**	26.13	77.64	71.71	44.58
(*S*)‐**9d**	34.01	80.25	70.61	76.45
(*S*)‐**9e**	45.06	82.96	88.73	85.20

**Figure 3:**
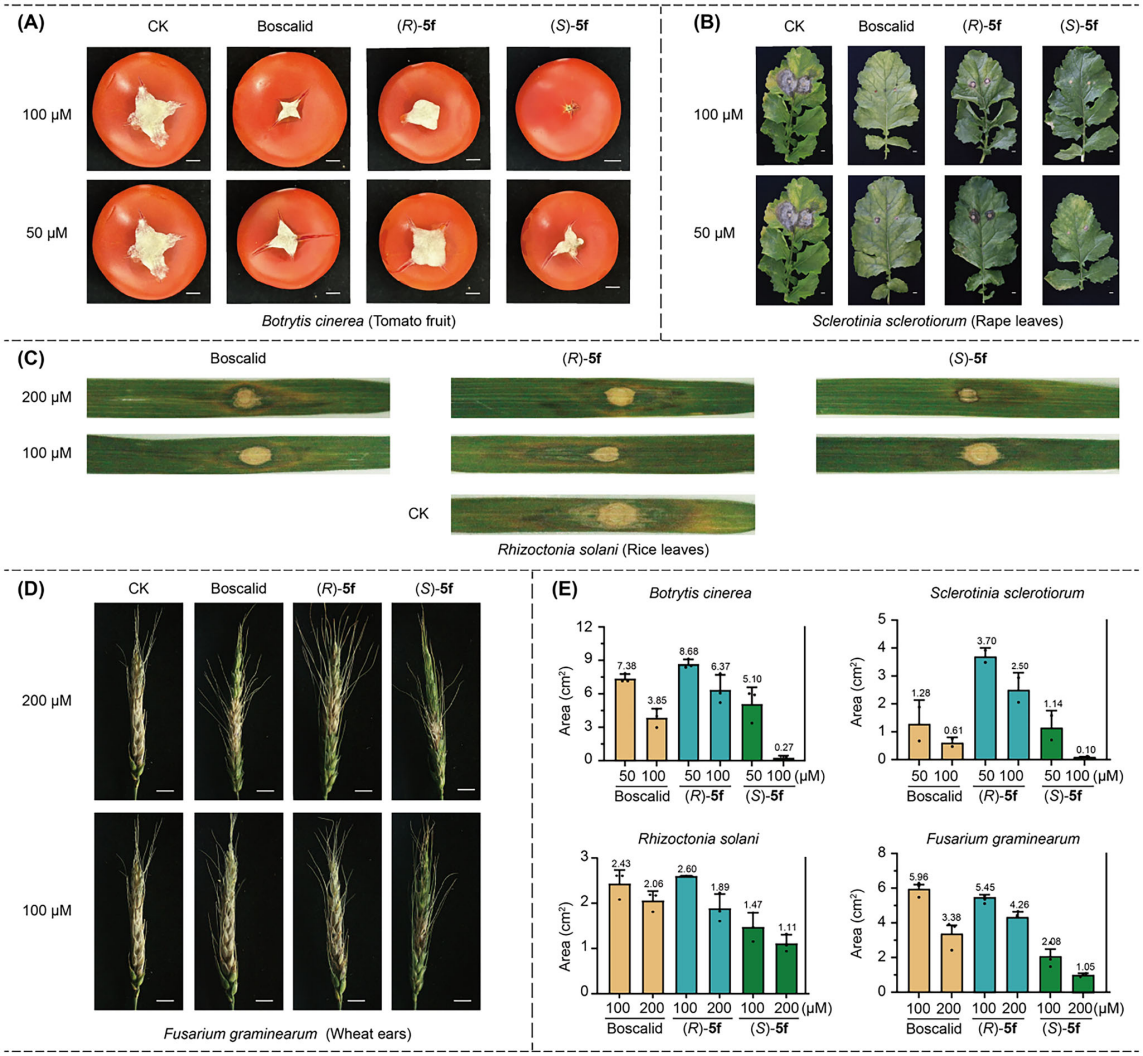
In vivo antifungal experiments. A) Inhibitory effects of *B. c* on tomato fruit. B) Inhibitory effects of *S. s* in rape leaves. C) Inhibitory effects of *R. s* in rape leaves. D) Inhibitory effects of *F. g* in wheat ears. (E) In vivo lesion area statistics of four pathogenic fungi.

Encouraged by the promising in vitro results, we proceeded to evaluate the in vivo antifungal activity of (*S*)‐**5f** and (*R*)‐**5f**. In our initial experiments, we focused on the inhibitory effect of *Botrytis cinerea* on tomato fruits, as depicted in **Figure** **3**A. At a concentration of 100 µm, (*S*)‐**5f** was able to completely inhibit fungal growth on the tomato fruits. In contrast, treatments with Boscalid and (*R*)‐**5f** allowed *Botrytis cinerea* to grow considerably. Fungi inoculation experiment on rape leaves showed that (*S*)‐**5f** and Boscalid can completely restrict hyphal growth at 50 µm, which is much stronger than its *R* enantiomer (Figure 3B). For rice stripe fungus, whether at the concentration of 100 or 200 µm, the effect of (*S*)‐**5f** is better than (*R*)‐**5f** and Boscalid (Figure 3C). Meanwhile, Fusarium head blight on wheat ears is a devastating fungal disease in wheat, (*S*)‐**5f** can still greatly inhibit its spread, while Boscalid and *R* enantiomer of **5f** cannot limit the growth of Fusarium head blight even under a concentration of 200 µm (Figure 3D). The in vivo experiments detailed above clearly demonstrate the potential application value of this chiral antifungal agent in practical agricultural production. In addition, we evaluated the enantiomer **5f** using acute toxicity tests in zebrafish. The experimental results showed that (*S*)‐**5f** (LC_50_ = 0.59 mg L^−1^) exhibited significantly higher toxicity than the *R*‐configured compound (LC_50_ = 7.54 mg L^−1^). (Figure S4, Supporting Information)

**Figure 4:**
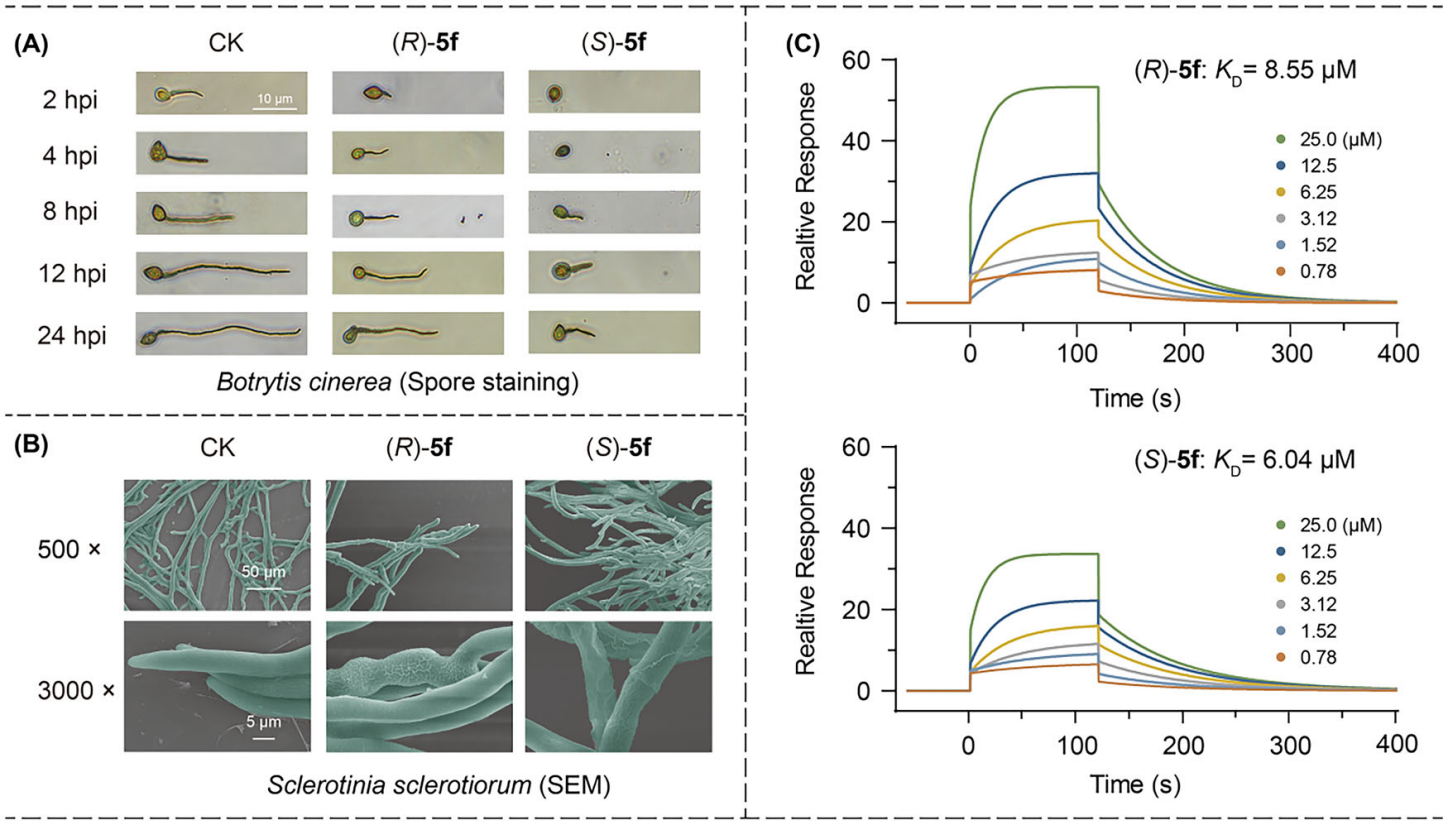
A) Staining of appressoria treated with (*R*)─**5f**, (*S*)─**5f** and Botrytis (4.0 µm). B) The effects of (*R*)─**5f**, (*S*)─**5f**, and Boscalid on the morphology of *S. s* (0.6 µm). C) Affinity assays between (*R*)─**5f**, (*S*)─**5f** and SDH using SPR.

To investigate the high antifungal activity and enantiomeric biological differences of compounds **5f**, we conducted the following antifungal experiments. Spore germination is a critical stage of fungal growth, through spore staining experiments on *Botrytis cinerea*, we found that compared to Boscalid and (*R*)‐**5f**, (*S*)‐**5f** can effectively inhibit spore germination and detachment of attachment cells (**Figure** **4**A). The scanning electron microscopy (SEM) images provide a striking visual comparison of the effects of (*S*)‐**5f** and its *R* enantiomer on the *Sclerotinia sclerotiorum* hyphae. The images reveal that mycelium treated with (*S*)‐**5f** experienced significant structural damage, with breaks and pronounced wrinkling on the surface (Figure 4B). These research results indicate that (*S*)‐**5f** inhibits fungal activity by disrupting fungal hyphal growth. As depicted in Figure 4C, we confirmed the difference in binding between enantiomers of **5f** and the target protein through ligand‐protein interaction experiments by surface plasmon resonance (SPR). The results showed that (*S*)‐**5f** had a stronger binding capacity to the target protein, with a K_D_ value of 6.04 µm, which is lower than that of (*R*)‐**5f** (K_D_ = 8.55 µm).

**Figure 5:**
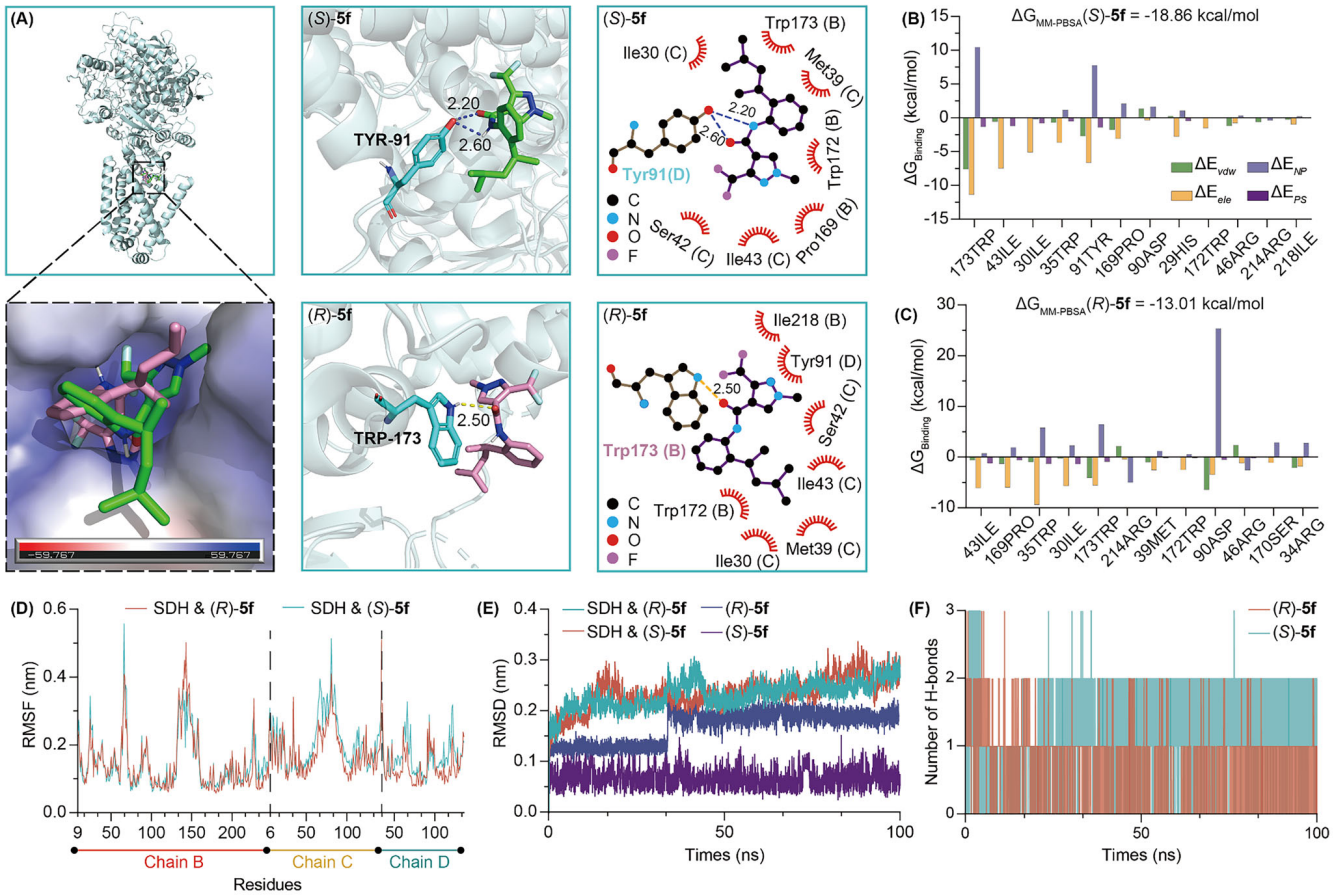
A) Molecular docking results of compounds (*S*)─**5f** and (*R*)─**5f** with SDH. B) MM‐PBSA energy decomposition of SDH with (*S*)─**5f**. C) MM‐PBSA energy decomposition of SDH with (*R*)─**5f**. D) RMSF values of the complexes of SDH with the two different chiral configurations of **5f** during MD. E) RMSD values of the protein and ligand in the complexes of SDH with (*S*)─**5f** and (*R*)─**5f**. F) Changes in the number of hydrogen bonds between SDH and the two different chiral configurations of **5f** during MD.

Finally, molecular docking experiments indicate that the embedding modes and angles adopted by (*S*)‐**5f** and (*R*)‐**5f** within the binding pocket of the target protein are completely different. This observation underscores the critical role of stereochemistry in molecular recognition and binding affinity. Also, the docking results indicate that (*S*)‐**5f** primarily interacts with the TYP‐91 residue of succinate dehydrogenase, while (*R*)‐**5f** engages with the TRP‐173 residue. (**Figure** **5**A). The results of the molecular dynamics simulation (MD) indicate that compound **5f** effectively binds to SDH.^[^ ^23^ ^]^ The Molecular Mechanics/Poisson‐Boltzmann Surface Area (MM‐PBSA) energy decomposition analysis reveals that the binding energy of (*S*)‐**5f** is −18.86 kcal mol^−1^, while that of (*R*)‐**5f** is −13.01 kcal mol^−1^ (Figure 5B,C). This indicates that the (*S*)‐configured compound binds more tightly to the protein. In the analysis of the MD trajectory, similar Root Mean Square Fluctuation (RMSF) values are observed for the two compounds with different chirality (Figure 5D). Additionally, the Root Mean Square Deviation (RMSD) values reveal significant differences in the stability of the binding of the two chiral compounds to the target protein, with the binding process of SDH and (*S*)‐**5f** being more stable (Figure 5E). Through the analysis of hydrogen bond interactions, the number of hydrogen bonds between (*R*)‐**5f** and SDH was significantly lower than that between (*S*)‐**5f** and SDH during the period of MD simulation. The aforementioned findings confirm that there are notable differences in the interactions between the two chiral molecules and the protein‐ligand interactions. These results confirm the different activities between enantiomers. The above results indicate that the significant differences in biological activity between enantiomers are multifaceted, originating not just from their unique inhibitory effects on target proteins, but also from their disparate capacities to impact fungal spore germination and to compromise the surface of mycelium. This nuanced distinction is crucial for understanding the selective action of chiral compounds in biological systems.

## 3 Conclusion

In conclusion, we have designed a series of novel chiral lead compounds focusing on succinate dehydrogenase, and developed an efficient asymmetric catalytic strategy for their synthesis, yielding 68 chiral antifungal molecules with remarkable efficiency and stereoselectivity. Mechanistic investigations have elucidated that the *ortho*‐NH_2_ substituent on the phenyl ring does not deactivate the metal catalyst, but serves as an activating group to enhance the reactivity and stereoselectivity. The antifungal assays have demonstrated that these compounds exhibit potent and broad‐spectrum activity against several common plant fungi, with a pronounced difference in efficacy between enantiomers. Additionally, a comprehensive study of the antifungal mechanisms has shed light on the origins of the differential activities among chiral enantiomers. Altogether, this work not only identifies a new class of chiral succinate dehydrogenase inhibitors as lead compounds but also offers theoretical guidance for the development of chiral pesticides.

## Conflict of Interest

The authors declare no conflict of interest.

## Supporting information



Supporting Information

Supporting cif

## Data Availability

The data that support the findings of this study are available in the Supporting Information of this article.
