# Supplementary material for: One‐Pot Synthesis of Chiral Succinate Dehydrogenase Inhibitors and Antifungal Activity Studies
Source: Adv Sci (Weinh). 2025 May 19;12(22):2416250. doi: 10.1002/advs.202416250 (PMC12165080; doi:10.1002/advs.202416250)
Supplement: Supplementary file 1 — Supporting Information [file ADVS-12-2416250-s001.pdf]

ADVANCED SCIENCE
Open Access

Supporting Information

for *Adv. Sci.*, DOI 10.1002/advs.202416250

One-Pot Synthesis of Chiral Succinate Dehydrogenase Inhibitors and Antifungal Activity Studies

*Donghua Du, Yu Chen, Chengbing Yang, Zheng Jin and Huailong Teng**

Supporting Information

One-pot Synthesis of Chiral Succinate Dehydrogenase Inhibitors and Antifungal Activity Studies

Donghua Du, Yu Chen, Chengbing Yang, Zheng Jin and Huailong Teng*

College of Chemistry, Huazhong Agricultural University, Wuhan, 430070, P. R. China.

Table of Contents

1. General remarks 1

2. Preparation of substrates 2

3. Synthesis of chiral succinate dehydrogenase inhibitors 3

4. Characterization data 4

5. Antifungal activity investigation 14

6. Antifungal experimental results 16

7. Absolute configuration determination 20

8. References 23

9. ^{1}H NMR and ^{13}C NMR spectras 25

10. HPLC chromatograms 63

1. General remarks

Reagents information: Unless otherwise stated, all reactions were carried out under an air atmosphere in screw cap reaction tubes, all chemical reagents were purchased from Shanghai Titan Scientific Co. Ltd, Bide Pharmatech Ltd, Aladdin Chemical Reagent Co. (China) and Sinopharm Chemical Reagent Company. Acetonitrile (MeCN), 1,2-dichloroethane (DCE), toluene, isopropyl alcohol (^{i}-PrOH) and 1,4-dioxane were freshly dried over calcium hydride or sodium.

Analytical information: ^{1}H NMR and ^{13}C NMR spectra were obtained on Bruker Avance II 600MHz NMR instrument. Chemical shifts were reported on the form of per million (ppm), and the residual solvent peak was used as an internal reference: ^{1}H NMR (chloroform δ 7.26), ^{13}C NMR (chloroform δ 77.16). Data are reported as follows: chemical shift, multiplicity (s = singlet, d = doublet, t = triplet, q = quartet, dd = doublet of doublets, dt = doublet of triplets, ddd = doublet of doublet of doublets, m = multiplet, etc.), coupling constants (Hz) and integration. High Resolution Mass Spectra was obtained on a Bruker FTMS. Scanning electron microscopy (SEM) was measured by using the H-7650 model from Japan. All reactions were monitored by thin-layer chromatography (TLC) with 0.2 mm silica gel-coated plates. Enantiomeric ratios were determined by an Agilent 1220 Infinity autosampler, using chiralpak AD-3 column, chiralcel OD-3 column and chiralcel OJ-H column with hexane and ^{i}-PrOH as solvents. The absolute configurations of (*R*)-**3k** and (*S*)-**3k** were determined by X-ray diffraction analysis. Optical rotations $[\alpha]^{25}_{D}$ were measured on a SGW®-533 polarimeter instrument.

2. Preparation of substrates

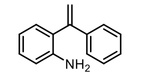

1a

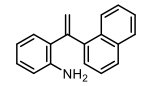

1b

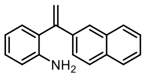

1c

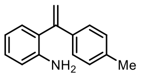

1d

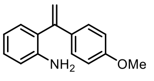

1e

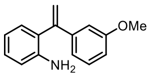

1f

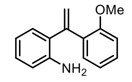

1g

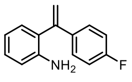

1h

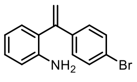

1i

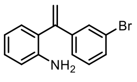

1j

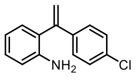

1k

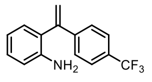

1l

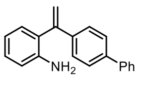

1m

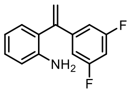

1n

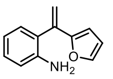

1o

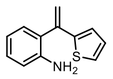

1p

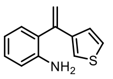

1q

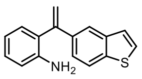

1r

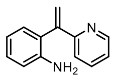

1s

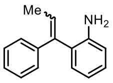

1t

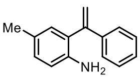

1u

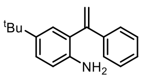

1v

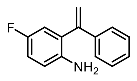

1w

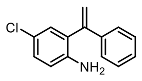

1x

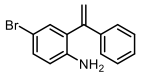

1y

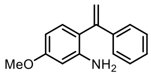

1z

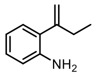

1a'

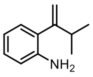

1b'

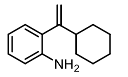

1c'

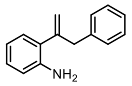

1d'

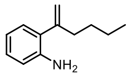

1e'

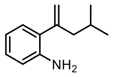

1f'

These substrates were synthesized according to reported methods.[1-4]

3. Synthesis of chiral succinate dehydrogenase inhibitors

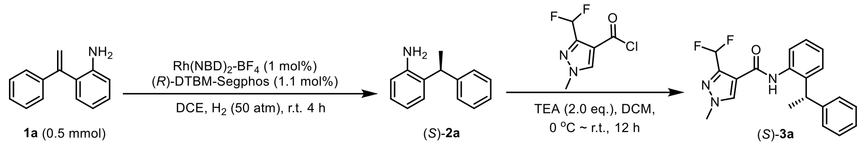

Step 1: Under a nitrogen atmosphere, Rh(NBD)$_2$BF$_4$ (5 μmol, 1 mol%) and (*R*)-DTBM-Segphos (5.5 μmol, 1.1 mol%) were dissolved in DCE (2 mL) and stirred for 30 minutes. Then the mixture was transferred into a vial containing substrate **1a** (0.5 mmol), transferred to a nitrogen-filled autoclave and carefully pressurized with hydrogen (50 bar). After being stirred at room temperature for 4 hours, the hydrogen gas was carefully released in a well-ventilated fume hood. The mixture was concentrated under reduced pressure, and the crude intermediate (*S*)-**2a** is directly used in the next step without further purification.

Step 2: Under nitrogen atmosphere, the intermediate (*S*)-**2a** (1.0 eq.) was dissolved in 2 mL of anhydrous DCM and transferred into a Schlenk tube sealed with a rubber stopper, and triethylamine (1.0 mmol, 2.0 eq.) was added. Then, the mixture was cooled in an ice bath, the pre-prepared acyl chloride (1.0 mmol, 2.0 eq.) was slowly added to the reaction mixture by a syringe. Upon completion of the reaction at room temperature for 12 hours, the reaction was quenched with 2 mL of water, and the mixture was extracted three times with DCM. The combined organic phase was dried over anhydrous Na_2SO_4, and the final product (*S*)-**3a** was purified by column chromatography.

Note: (*R*)-SDHIs were prepared following the same procedure with (*S*)-DTBM-Segphos as ligand.

4. Characterization data

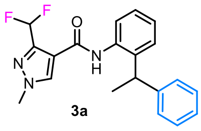

3a

(3a) 3-(difluoromethyl)-1-methyl-N-(2-(1-phenylethyl)phenyl)-1H-pyrazole-4-carboxamide: White solid, ^{1}H NMR (600 MHz, Chloroform-*d*) δ 7.75 (d, *J* = 6.0 Hz, 1H), 7.44 (s, 1H), 7.37 (d, *J* = 6.0 Hz, 1H), 7.32 – 7.28 (m, 3H), 7.25 (d, *J* = 6.0 Hz, 2H), 7.23 (d, *J* = 6.0 Hz, 1H), 7.18 (d, *J* = 12.0 Hz, 2H), 7.02 (t, *J* = 54.0 Hz, 1H), 4.27 (q, *J* = 6.0 Hz, 1H), 3.84 (s, 3H), 1.62 (d, *J* = 6.0 Hz, 3H); ^{13}C NMR (151 MHz, Chloroform-*d*) δ 159.8, 145.1, 144.7 (t, *J* = 27.1 Hz), 137.6, 134.8, 133.0, 128.9, 127.9, 127.7, 127.2, 126. 8, 126.1, 125.2, 116.7, 110.5 (t, *J* = 235.5 Hz), 40.3, 39.6, 21.7; ^{19}F NMR (565 MHz, Chloroform-*d*) δ -111.58 (d, *J* = 310.7 Hz), -112.71 (d, *J* = 310.7 Hz). HRMS Calcd. For $[C_{20}H_{20}F_{2}N_{3}O]^{+}$: 356.1569, found: 356.1570.

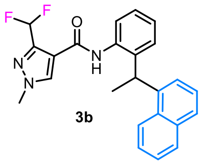

3b

(3b) 3-(difluoromethyl)-1-methyl-N-(2-(1-(naphthalen-1-yl)ethyl)phenyl)-1H-pyrazole-4-carboxamide: White solid, ^{1}H NMR (600 MHz, Chloroform-*d*) δ 8.22 (d, *J* = 6.0 Hz, 1H), 7.96 (d, *J* = 6.0 Hz, 1H), 7.90 (d, *J* = 6.0 Hz, 1H), 7.78 (d, *J* = 6.0 Hz, 1H), 7.57 – 7.51 (m, 3H), 7.39 (t, *J* = 6.0 Hz, 1H), 7.34 – 7.32 (m, 1H), 7.29 – 7.25 (m, 1H), 7.14 (d, *J* = 6.0 Hz, 1H), 7.07 (t, *J* = 54.0 Hz, 1H), 6.98 (s, 1H), 5.99 (s, 1H), 4.99 (q, *J* = 6.0 Hz, 1H), 3.53 (s, 3H), 1.76 (d, *J* = 6.0 Hz, 3H); ^{13}C NMR (151 MHz, Chloroform-*d*) δ 159.4, 145.4 (t, *J* = 25.6 Hz), 141.1, 136.7, 134.7, 134.0, 130.9, 130.8, 129.3, 127.6, 127.3, 126.8, 126.4, 126.0, 125.6, 125.28, 124.3, 122.8, 116.2, 109.8 (t, *J* = 235.5 Hz), 39.3, 35.5, 20.9; ^{19}F NMR (565 MHz, Chloroform-*d*) δ -114.19. HRMS Calcd. For $[C_{24}H_{22}F_{2}N_{3}O]^{+}$: 406.1725, found: 406.1727.

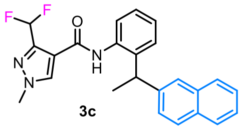

3c

(3c) 3-(difluoromethyl)-1-methyl-N-(2-(1-(naphthalen-2-yl)ethyl)phenyl)-1H-pyrazole-4-carboxamide: White solid, ^{1}H NMR (600 MHz, Chloroform-*d*) δ 7.83 (t, *J* = 6.0 Hz, 2H), 7.78 (t, *J* = 6.0 Hz, 2H), 7.72 (s, 1H), 7.53 – 7.49 (m, 2H), 7.44 (d, *J* = 12.0 Hz, 1H), 7.40 (s, 1H), 7.32 (t, *J* = 6.0 Hz, 1H), 7.27 – 7.24 (m, 2H), 7.04 (t, *J* = 54.0 Hz, 1H), 6.73 (s, 1H), 4.42 (q, *J* = 6.0 Hz, 1H), 3.63 (s, 3H), 1.73 (d, *J* = 12.0 Hz, 3H); ^{13}C NMR (151 MHz, Chloroform-*d*) δ 159.6, 144.9 (t, *J* = 25.7 Hz), 142.6, 136.6, 135.1, 133.5, 132.4, 129.0, 128.3, 127.9, 127.6, 127.5, 126.6, 126.4, 126.1, 125.9, 125.5, 124.7, 116.7, 110.3 (t, *J* = 235.5 Hz), 41.0, 39.4, 21.4; ^{19}F NMR (565 MHz, Chloroform-*d*) δ -112.27 (d, *J* = 310.7 Hz), -113.26 (d, *J* = 310.7 Hz). HRMS Calcd. For $[C_{24}H_{22}F_{2}N_{3}O]^{+}$: 406.1725, found: 406.1727.

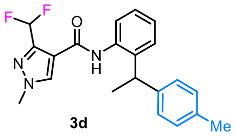

3d

(3d) 3-(difluoromethyl)-1-methyl-N-(2-(1-(p-tolyl)ethyl)phenyl)-1H-pyrazole-4-carboxamide: White solid, ^{1}H NMR (600 MHz, Chloroform-*d*) δ 7.81 (d, *J* = 12.0 Hz, 1H), 7.40 (s, 1H), 7.37 (d, *J* = 6.0 Hz, 1H), 7.29 (t, *J* = 12.0 Hz, 1H), 7.23 (t, *J* = 6.0 Hz, 1H), 7.19 (s, 1H), 7.14 (d, *J* = 6.0 Hz, 2H), 7.08 (d, *J* = 6.0 Hz, 2H), 7.03 (t, *J* = 54.0 Hz, 1H), 4.22 (q, *J* = 6.0 Hz, 1H), 3.89 (s, 3H), 2.34 (s, 3H), 1.61 (d, *J* = 6.0 Hz, 3H); ^{13}C NMR (151 MHz, Chloroform-*d*) δ 159.6, 144.8, 142.0, 137.3, 136.4, 134.8, 132.9, 129.6, 127.9, 127.6, 127.2, 125.9, 124.8, 116.9, 110.5 (t, *J* = 235.5 Hz), 40.0, 39.6, 21.8, 21.1; ^{19}F NMR (565 MHz, Chloroform-*d*) δ -111.82 (d, *J* = 305.1 Hz), -112.88 (d, *J* = 305.1 Hz). HRMS Calcd. For $[C_{21}H_{22}F_2N_3O]^+$: 370.1725, found: 370.1726.

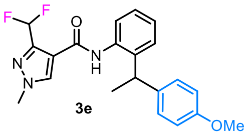

3e

(3e) 3-(difluoromethyl)-N-(2-(1-(4-methoxyphenyl)ethyl)phenyl)-1-methyl-1H-pyrazole-4-carboxamide: White solid, ^{1}H NMR (600 MHz, Chloroform-*d*) δ 7.80 (d, *J* = 6.0 Hz, 1H), 7.46 (s, 1H), 7.36 (d, *J* = 6.0 Hz, 1H), 7.30 – 7.22 (m, 3H), 7.11 (d, *J* = 6.0 Hz, 2H), 7.03 (t, *J* = 54.0 Hz, 1H), 6.85 (d, *J* = 6.0 Hz, 2H), 4.21 (q, *J* = 6.0 Hz, 1H), 3.89 (s, 3H), 3.79 (s, 3H), 1.60 (d, *J* = 6.0 Hz, 3H); ^{13}C NMR (151 MHz, Chloroform-*d*) δ 159.7, 158.4, 137.5, 137.0, 134.8, 133.1, 128.7, 127.9, 127.2, 125.9, 124.9, 116.9, 114.3, 110.6 (t, *J* = 235.5 Hz), 55.4, 39.6, 39.5, 21.9; ^{19}F NMR (565 MHz, Chloroform-d) δ -111.54 (d, *J* = 305.1 Hz), -112.53 (d, *J* = 305.1 Hz). HRMS Calcd. For $[C_{21}H_{22}F_2N_3O_2]^+$: 386.1675, found: 386.1675.

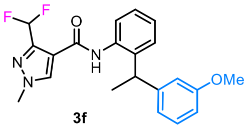

3f

(3f) 3-(difluoromethyl)-N-(2-(1-(3-methoxyphenyl)ethyl)phenyl)-1-methyl-1H-pyrazole-4-carboxamide: White solid, ^{1}H NMR (600 MHz, Chloroform-*d*) δ 7.79 (d, *J* = 6.0 Hz, 1H), 7.40 (d, *J* = 6.0 Hz, 2H), 7.31 – 7.23 (m, 4H), 7.07 (t, *J* = 54.0 Hz, 1H), 6.80 – 6.76 (m, 3H), 4.21 (q, *J* = 6.0 Hz, 1H), 3.90 (s, 3H), 3.76 (s, 3H), 1.62 (d, *J* = 6.0 Hz, 3H); ^{13}C NMR (151 MHz, Chloroform-*d*) δ 160.1, 159.6, 146.9, 145.0, 137.1, 134.9, 132.7, 130.2, 127.8, 127.3, 125.9, 124.9, 120.1, 116.8, 113.8, 111.8, 110.4 (t, *J* = 235.5 Hz), 55.3, 40.6, 39.6, 21.5; ^{19}F NMR (565 MHz, Chloroform-*d*) δ -112.19 (d, *J* = 305.1 Hz), -113.16 (d, *J* = 305.1 Hz). HRMS Calcd. For $[C_{21}H_{22}F_2N_3O_2]^+$: 386.1675, found: 386.1675.

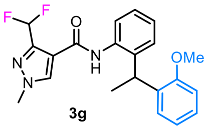

3g

(3g) 3-(difluoromethyl)-N-(2-(1-(2-methoxyphenyl)ethyl)phenyl)-1-methyl-1H-pyrazole-4-carboxamide: White solid, ^{1}H NMR (600 MHz, Chloroform-*d*) δ 7.81 (s, 2H), 7.53 (s, 1H), 7.35 (d, *J* = 6.0 Hz, 1H), 7.26 – 7.17 (m, 3H), 7.11 (d, *J* = 6.0 Hz, 1H), 7.05 (t, *J* = 54.0 Hz, 1H), 6.93 (t, *J* = 6.0 Hz, 1H), 6.84 (d, *J* = 12.0 Hz, 1H), 4.62 (q, *J* = 6.0 Hz, 1H), 3.92 (s, 3H), 3.68 (s, 3H), 1.56 (d, *J* = 6.0 Hz, 3H); ^{13}C NMR (151 MHz, Chloroform-*d*) δ 159.9, 156.3, 144.8 (t, *J* = 26.4 Hz), 137.6, 134.6, 133.4, 132.8, 127.8, 127.6, 127.4, 126.7, 125.7, 124.6, 121.4, 117.3, 110.6, 110.4 (t, *J* = 235.5 Hz), 55.5, 39.6, 32.2, 20.5; ^{19}F NMR (565 MHz, Chloroform-*d*) δ -112.46. HRMS Calcd. For $[C_{21}H_{22}F_2N_3O_2]^+$: 386.1675, found: 386.1675.

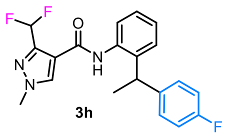

(3h) 3-(difluoromethyl)-N-(2-(1-(4-fluorophenyl)ethyl)phenyl)-1-methyl-1H-pyrazole-4-carboxamide: White solid, ^{1}H NMR (600 MHz, Chloroform-*d*) δ 7.67 (d, *J* = 12.0 Hz, 1H), 7.56 (s, 2H), 7.32 – 7.23 (m, 3H), 7.13 – 7.11 (m, 2H), 6.96 – 6.92 (m, 2H), 6.92 (t, *J* = 54.0 Hz, 1H), 4.30 (q, *J* = 6.0 Hz, 1H), 3.88 (s, 3H), 1.59 (d, *J* = 12.0 Hz, 3H); ^{13}C NMR (151 MHz, Chloroform-*d*) δ 162.3, 160.7, 159.9, 143.7, 140.8 (d, *J* = 3.2 Hz), 138.6, 134.4 (d, *J* = 30.3 Hz), 129.1 (d, *J* = 7.8 Hz), 127.8, 127.2, 126.5, 125.9, 116.7, 115.5 (d, *J* = 21.1 Hz), 111.1 (t, *J* = 234.0 Hz), 39.6, 39.1, 21.9; ^{19}F NMR (565 MHz, Chloroform-*d*) δ -109.89 (d, *J* = 305.1 Hz), -110.78 (d, *J* = 305.1 Hz), -116.48. HRMS Calcd. For $[C_{20}H_{19}F_3N_3O]^+$: 374.1475, found: 374.1475.

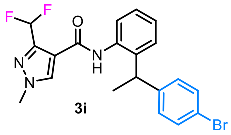

(3i) N-(2-(1-(4-bromophenyl)ethyl)phenyl)-3-(difluoromethyl)-1-methyl-1H-pyrazole-4-carboxamide: White solid, ^{1}H NMR (600 MHz, Chloroform-*d*) δ 7.45 (d, *J* = 6.0 Hz, 1H), 7.34 (d, *J* = 24.0 Hz, 2H), 7.18 (d, *J* = 6.0 Hz, 2H), 7.09 (t, *J* = 6.0 Hz, 2H), 7.07 – 7.03 (m, 1H), 6.84 (d, *J* = 12.0 Hz, 2H), 6.74 (d, *J* = 54.0 Hz, 1H), 4.08 (q, *J* = 6.0 Hz, 1H), 3.68 (s, 3H), 1.39 (d, *J* = 6.0 Hz, 3H); ^{13}C NMR (151 MHz, Chloroform-*d*) δ 159.9, 144.2, 143.7 (t, *J* = 28.69 Hz), 138.3, 134.5, 134.4, 131.7, 129.4, 127.9, 127.4, 126.6, 126.0, 120.3, 116.7, 111.2 (t, *J* = 234.0 Hz), 39.7, 39.3, 21.6; ^{19}F NMR (565 MHz, Chloroform-*d*) δ -109.77 (d, *J* = 310.7 Hz), -110.57 (d, *J* = 305.1 Hz). HRMS Calcd. For $[C_{20}H_{19}BrF_2N_3O]^+$: 434.0674, found: 434.0675.

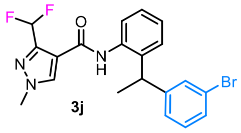

(3j) N-(2-(1-(3-bromophenyl)ethyl)phenyl)-3-(difluoromethyl)-1-methyl-1H-pyrazole-4-carboxamide: White solid, ^{1}H NMR (600 MHz, Chloroform-*d*) δ 7.61 (d, *J* = 6.0 Hz, 1H), 7.56 (s, 1H), 7.47 (s, 1H), 7.35 (s, 1H), 7.32 (t, *J* = 6.0 Hz, 2H), 7.28 – 7.24 (m, 2H), 7.10 (t, *J* = 6.0 Hz, 1H), 7.02 (t, *J* = 54.0 Hz, 1H), 7.01 (s, 1H), 4.25 (q, *J* = 6.0 Hz, 1H), 3.84 (s, 3H), 1.57 (d, *J* = 12.0 Hz, 3H); ^{13}C NMR (151 MHz, Chloroform-*d*) δ 160.0, 147.7, 144.3, 138.3, 134.5, 133.4, 130.5, 129.6, 127.7, 127.4, 126.6, 126.3, 126.2, 122.6, 116.3, 110.7 (t, *J* = 234.5 Hz), 39.8, 39.6, 21.4; ^{19}F NMR (377 MHz, Chloroform-*d*) δ -111.40. HRMS Calcd. For $[C_{20}H_{19}BrF_2N_3O]$+: 434.0674, found: 434.0675.

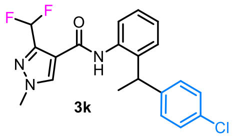

(3k) N-(2-(1-(4-chlorophenyl)ethyl)phenyl)-3-(difluoromethyl)-1-methyl-1H-pyrazole-4-carboxamide: White solid, ^{1}H NMR (600 MHz, Chloroform-*d*) δ 7.65 (d, *J* = 6.0 Hz, 1H), 7.55 (d, *J* = 12.0 Hz, 2H), 7.32 – 7.27 (m, 2H), 7.27 – 7.21 (m, 3H), 7.09 (d, *J* = 6.0 Hz, 2H), 6.93 (t, *J* = 54.0 Hz, 1H), 4.29 (q, *J* = 7.1 Hz, 1H), 3.88 (s, 3H), 1.59 (d, *J* = 7.2 Hz, 3H); ^{13}C NMR (151 MHz, Chloroform-*d*) δ 159.9, 143.7, 138.4, 134.4 (d, *J* = 16.6 Hz), 132.2, 129.0, 128.8, 127.9, 127.3, 126.6, 126.0, 116.6,

111.22 (t, *J* = 234.0 Hz), 39.6, 39.2, 21.6; ^{19}F NMR (565 MHz, Chloroform-*d*) δ -109.79 (d, *J* = 305.1 Hz), -110.61 (d, *J* = 305.1 Hz). HRMS Calcd. For $[C_{20}H_{19}ClF_2N_3O]^+$: 390.1179, found: 390.1180.

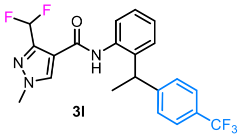

(3l) 3-(difluoromethyl)-1-methyl-N-(2-(1-(4-(trifluoromethyl)phenyl)ethyl)phenyl)-1H-pyrazole-4-carboxamide: White solid, ^{1}H NMR (600 MHz, Chloroform-*d*) δ 7.59 (d, *J* = 6.0 Hz, 3H), 7.48 (d, *J* = 6.0 Hz, 2H), 7.29 (d, *J* = 6.0 Hz, 2H), 7.27 – 7.23 (m, 3H), 6.87 (t, *J* = 54.2 Hz, 1H), 4.38 (q, *J* = 6.0 Hz, 1H), 3.85 (s, 3H), 1.61 (d, *J* = 6.0 Hz, 1H); ^{13}C NMR (151 MHz, Chloroform-*d*) δ 160.0, 149.4, 143.36 (t, *J* = 24.1 Hz), 138.8, 134.8, 134.5, 128.68 (d, *J* = 31.7 Hz), 128.0, 127.9, 127.4, 126.9, 126.6, 125.54 (q, *J* = 3.7 Hz), 125.1, 123.3, 116.5, 111.48 (t, *J* = 234.0 Hz), 39.64, 39.58, 21.51; ^{19}F NMR (565 MHz, Chloroform-*d*) δ -62.39, -109.30 (d, *J* = 305.1 Hz), -110.00 (d, *J* = 322.0 Hz). HRMS Calcd. For $[C_{21}H_{19}F_5N_3O]^+$: 424.1443, found: 424.1443.

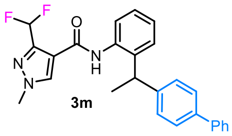

(3m) N-(2-(1-([1,1'-biphenyl]-4-yl)ethyl)phenyl)-3-(difluoromethyl)-1-methyl-1H-pyrazole-4-carboxamide: White solid, ^{1}H NMR (600 MHz, Chloroform-*d*) δ 7.78 (d, *J* = 6.0 Hz, 1H), 7.56 (d, *J* = 6.0 Hz, 2H), 7.53 (d, *J* = 6.0 Hz, 2H), 7.46 (s, 1H), 7.43 (t, *J* = 6.0 Hz, 2H), 7.40 (d, *J* = 6.0 Hz, 1H), 7.35 (d, *J* = 6.0 Hz, 1H), 7.33 – 7.29 (m, 2H), 7.28 – 7.25 (m, 3H), 6.97 (t, *J* = 54.0 Hz, 1H), 4.32 (q, *J* = 6.0 Hz, 1H), 3.78 (s, 3H), 1.66 (d, *J* = 6.0 Hz, 3H); ^{13}C NMR (151 MHz, Chloroform-*d*) δ 159.8, 144.3 (t, *J* = 25.6 Hz), 144.1, 140.5, 139.6, 137.6, 134.8, 133.4, 129.0, 128.1, 128.0, 127.5, 127.3, 126.9, 126.2, 125.3, 116.9, 110.8 (t, *J* = 234.0 Hz), 40.0, 39.6, 21.7; ^{19}F NMR (565 MHz, Chloroform-*d*) δ -111.01 (d, *J* = 305.1 Hz), -112.00 (d, *J* = 305.1 Hz). HRMS Calcd. For $[C_{26}H_{24}F_2N_3O]^+$: 432.1882, found: 432.1882.

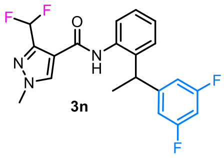

(3n) 3-(difluoromethyl)-N-(2-(1-(3,5-difluorophenyl)ethyl)phenyl)-1-methyl-1H-pyrazole-4-carboxamide: White solid, ^{1}H NMR (600 MHz, Chloroform-*d*) δ 7.72 (s, 1H), 7.69 (s, 1H), 7.57 (d, *J* = 6.0 Hz, 1H), 7.30 – 7.24 (m, 3H), 6.91 (t, *J* = 54.0 Hz, 1H), 6.71 – 6.63 (m, 2H), 6.62 – 6.60 (m, 1H), 4.32 (q, *J* = 6.0 Hz, 1H), 3.86 (s, 3H), 1.58 (d, *J* = 6.0 Hz, 3H); ^{13}C NMR (151 MHz, Chloroform-*d*) δ 163.9 (d, *J* = 13.6 Hz), 162.3 (d, *J* = 12.1 Hz), 160.2, 149.4 (t, *J* = 7.5 Hz), 143.5 (t, *J* = 28.7 Hz), 138.8, 134.8, 134.4, 127.7, 127.5, 127.0, 126.7, 116.4, 111.4 (t, *J* = 234.0Hz), 110.57 (dd, *J* = 21.1, 6.0 Hz), 101.92 (t, *J* = 24.1 Hz), 39.6, 39.3, 21.3; ^{19}F NMR (565 MHz, Chloroform-*d*) δ -109.82 (d, *J* = 310.7 Hz), -109.69, -110.12 (d, *J* = 310.7 Hz). HRMS Calcd. For $[C_{20}H_{18}F_4N_3O]^+$: 392.1381, found: 392.1382.

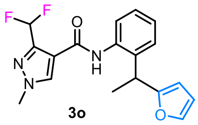

(3o) 3-(difluoromethyl)-N-(2-(1-(furan-2-yl)ethyl)phenyl)-1-methyl-1H-pyrazole-4-carboxamide: Light yellow solid, ^{1}H NMR (600 MHz, Chloroform-*d*) δ 8.05 (s, 1H), 7.81 (s, 1H), 7.69 (d, *J* = 6.0 Hz,

1H), 7.30 (s, 1H), 7.29 – 7.26 (m, 1H), 7.24 – 7.19 (m, 2H), 6.97 (t, *J* = 54.0 Hz, 1H), 6.30 (dd, *J* = 3.1, 1.9 Hz, 1H), 6.05 (d, *J* = 6.0 Hz, 1H), 4.35 (q, *J* = 6.0 Hz, 1H), 3.87 (s, 3H), 1.59 (d, *J* = 12.0 Hz, 3H); ^{13}C NMR (151 MHz, Chloroform-*d*) δ 160.2, 158.0, 143.71 (t, *J* = 21.1Hz), 141.7, 137.2, 134.7, 134.3, 127.6, 126.7, 126.3, 116.7, 111.2 (t, *J* = 234.0 Hz), 110.2, 105.3, 39.6, 34.0, 18.9; ^{19}F NMR (565 MHz, Chloroform-*d*) δ -110.36. HRMS Calcd. For $[C_{18}H_{18}F_2N_3O_2]^+$: 346.1362, found: 346.1363.

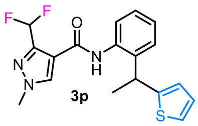
3p

(3p) 3-(difluoromethyl)-1-methyl-N-(2-(1-(thiophen-2-yl)ethyl)phenyl)-1H-pyrazole-4-carboxamide: White solid, ^{1}H NMR (400 MHz, Chloroform-*d*) δ 7.76 (d, *J* = 8.0 Hz, 1H), 7.67 (s, 1H), 7.51 (s, 1H), 7.34 (t, *J* = 8.0 Hz, 1H), 7.30 (d, *J* = 4.0 Hz, 1H), 7.26 (d, *J* = 4.0 Hz, 1H), 7.22 (t, *J* =4.0 Hz, 1H), 6.99 (t, *J* = 56.0 Hz, 1H), 6.95 (t, *J* = 4.0 Hz, 1H), 6.79 (d, *J* = 4.0 Hz, 1H), 4.52 (q, *J* = 4.0 Hz, 1H), 3.90 (s, 3H), 1.70 (d, *J* = 8.0 Hz, 3H); ^{13}C NMR (101 MHz, Chloroform-*d*) δ 159.9, 149.3, 144.3, 137.9, 134.4, 133.8, 127.9, 127.6, 126.9, 126.4, 125.6, 124.4, 116.8, 110.9 (t, *J* = 235.3 Hz), 39.7, 36.3, 22.5; ^{19}F NMR (377 MHz, Chloroform-*d*) δ -111.22 (d, *J* = 56.5 Hz). HRMS Calcd. For $[C_{18}H_{18}F_2N_3OS]^+$: 362.1133, found: 362.1134.

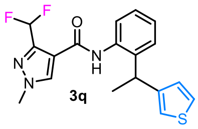
3q

(3q) 3-(difluoromethyl)-1-methyl-N-(2-(1-(thiophen-3-yl)ethyl)phenyl)-1H-pyrazole-4-carboxamide: White solid, ^{1}H NMR (600 MHz, Chloroform-*d*) δ 7.73 (d, *J* = 12.0 Hz, 2H), 7.49 (s, 1H), 7.35 (d, *J* = 12.0 Hz, 1H), 7.29 (t, *J* = 6.0 Hz, 1H), 7.24 (t, *J* = 6.0 Hz, 1H), 7.19 (d, *J* = 6.0 Hz, 1H), 7.01 (t, *J* = 54.0 Hz, 1H), 6.94 (t, *J* = 6.0 Hz, 1H), 6.79 (d, *J* = 6.0 Hz, 1H), 4.54 (q, *J* = 6.0 Hz, 1H), 3.85 (s, 3H), 1.68 (d, *J* = 6.0 Hz, 3H); ^{13}C NMR (151 MHz, Chloroform-*d*) δ 159.9, 149.3, 144.3 (t, *J* = 24.1 Hz), 138.1, 134.4, 133.7, 127.9, 127.5, 126.9, 126.4, 125.7, 124.3, 116.7, 110.8 (t, *J* = 234.0 Hz), 39.6, 36.2, 22.4; ^{19}F NMR (565 MHz, Chloroform-*d*) δ -111.34. HRMS Calcd. For $[C_{18}H_{18}F_2N_3OS]^+$: 362.1133, found: 362.1134.

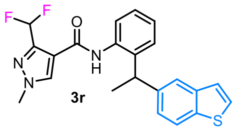
3r

(3r) N-(2-(1-(benzo[b]thiophen-5-yl)ethyl)phenyl)-3-(difluoromethyl)-1-methyl-1H-pyrazole-4-carboxamide: White solid, ^{1}H NMR (600 MHz, Chloroform-*d*) δ 7.80 (d, *J* = 12.0 Hz, 2H), 7.68 (s, 1H), 7.48 (d, *J* = 6.0 Hz, 1H), 7.42 (d, *J* = 6.0 Hz, 1H), 7.38 (s, 1H), 7.31 (t, *J* = 6.0 Hz, 1H), 7.27 - 7.24 (m, 2H), 7.14 (d, *J* = 6.0 Hz, 1H), 7.03 (t, *J* = 54.0 Hz, 1H), 6.84 (s, 1H), 4.37 (q, *J* = 6.0 Hz, 1H), 3.71 (s, 3H), 1.68 (d, *J* = 6.0 Hz, 3H); ^{13}C NMR (151 MHz, Chloroform-*d*) δ 159.6, 144.8 (t, *J* = 28.6 Hz), 141.4, 140.1, 138.2, 137.1, 134.9, 132.5, 128.1, 127.5, 127.4, 125.9, 124.8, 124.5, 123.7, 123.2, 122.1, 116.7, 110.4 (t, *J* = 234.0 Hz), 40.61, 39.48, 21.92; ^{19}F NMR (565 MHz, Chloroform-*d*) δ -112.0 (d, *J* = 310.7 Hz), -113.13 (d, *J* = 310.7 Hz),. HRMS Calcd. For $[C_{22}H_{20}F_2N_3OS]^+$: 412.1290, found: 412.1293.

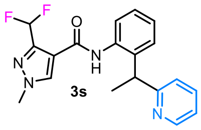
3s

(3s) 3-(difluoromethyl)-1-methyl-N-(2-(1-(pyridin-2-yl)ethyl)phenyl)-1H-pyrazole-4-carboxamide: Yellow solid, ^{1}H NMR (600 MHz, Chloroform-*d*) δ 10.99 (s, 1H), 8.46 (d, *J* = 6.0 Hz, 1H), 8.28 (s, 1H), 8.04 (d, *J* = 12.0 Hz, 1H), 7.71 (td, *J* = 12.0, 6.0 Hz, 1H), 7.42 (t, *J* = 54.0 Hz, 1H), 7.36 (t, *J* = 12.0 Hz, 2H), 7.26 – 7.23 (m, 1H), 7.20 (dd, *J* = 6.8, 5.1 Hz, 1H), 7.13 – 7.11 (m, 1H), 4.50 (q, *J* = 6.0 Hz, 1H), 4.06 (s, 3H), 1.77 (d, *J* = 6.0 Hz, 3H); ^{13}C NMR (151 MHz, Chloroform-*d*) δ 164.9, 159.9, 147.7, 146.8 (t, *J* = 24.1 Hz), 138.3, 136.2, 134.6, 131.4, 127.4, 126.7, 125.0, 124.4, 122.3, 121.5, 117.9, 109.6 (t, *J* = 237.1 Hz), 43.6, 40.0, 17.9; ^{19}F NMR (565 MHz, Chloroform-*d*) δ -115.24 (d, *J* = 305.1 Hz), -116.01 (d, *J* = 305.1 Hz). HRMS Calcd. For $[C_{19}H_{19}F_2N_4O]^+$: 357.1521, found: 357.1519.

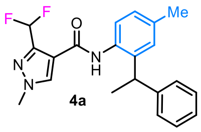

4a

(4a) 3-(difluoromethyl)-1-methyl-N-(4-methyl-2-(1-phenylethyl)phenyl)-1H-pyrazole-4-carboxamide: White solid, ^{1}H NMR (600 MHz, Chloroform-*d*) δ 7.58 (d, *J* = 6.0 Hz, 1H), 7.35 (s, 1H), 7.30 (t, *J* = 6.0 Hz, 3H), 7.23 (t, *J* = 6.0 Hz, 1H), 7.18 (t, *J* = 6.0 Hz, 3H), 7.09 (d, *J* = 12.0 Hz, 1H), 7.00 (t, *J* = 54.0 Hz, 1H), 4.24 (q, *J* = 6.0 Hz, 1H), 3.86 (s, 3H), 2.36 (s, 3H), 1.60 (d, *J* = 6.0 Hz, 3H); ^{13}C NMR (151 MHz, Chloroform-*d*) δ 159.8, 145.3, 144.5, 137.9, 135.9, 133.1, 132.0, 128.9, 128.5, 127.8, 127.7, 126.6, 125.41, 116.8, 110.59 (t, *J* = 235.5 Hz), 40.2, 39.6, 21.7, 21.4; ^{19}F NMR (565 MHz, Chloroform-*d*) δ -111.47 (d, *J* = 305.1 Hz), -112.52 (d, *J* = 305.1 Hz). HRMS Calcd. For $[C_{21}H_{22}F_2N_3O]^+$: 370.1725, found: 370.1726.

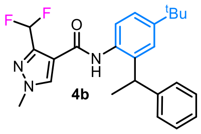

4b

(4b) N-(4-(tert-butyl)-2-(1-phenylethyl)phenyl)-3-(difluoromethyl)-1-methyl-1H-pyrazole-4-carboxamide: White solid, ^{1}H NMR (600 MHz, Chloroform-*d*) δ 7.65 (d, *J* = 12.0 Hz, 1H), 7.39 (d, *J* = 6.0 Hz, 1H), 7.31 (t, *J* = 6.0 Hz, 4H), 7.26 – 7.23 (m, 2H), 7.19 (d, *J* = 6.0 Hz, 2H), 7.00 (t, *J* = 54.0 Hz, 1H), 4.26 (q, *J* = 6.0 Hz, 1H), 3.88 (s, 3H), 1.63 (d, *J* = 6.0 Hz, 3H), 1.33 (s, 9H); ^{13}C NMR (151 MHz, Chloroform-*d*) δ 159.7, 148.9, 145.3, 144.6, 137.0, 133.0, 132.0, 128.9, 127.7, 126.7, 124.9, 124.2, 116.9, 110.5 (t, *J* = 235.5 Hz), 40.7, 39.6, 34.7, 31.5, 21.7; ^{19}F NMR (565 MHz, Chloroform-*d*) δ -111.55 (d, *J* = 305.1 Hz), -112.56 (d, *J* = 310.7 Hz). HRMS Calcd. For $[C_{24}H_{28}F_2N_3O]^+$: 412.2195, found: 412.2196.

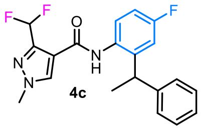

4c

(4c) 3-(difluoromethyl)-N-(4-fluoro-2-(1-phenylethyl)phenyl)-1-methyl-1H-pyrazole-4-carboxamide: White solid, ^{1}H NMR (600 MHz, Chloroform-*d*) δ 7.57 (t, *J* = 6.0 Hz, 1H), 7.31 (d, *J* = 18.0 Hz, 2H), 7.24 (t, *J* = 6.0 Hz, 2H), 7.18 (d, *J* = 6.0 Hz, 1H), 7.09 (d, *J* = 6.0 Hz, 2H), 6.98 (dd, *J* = 12.0, 6.0 Hz, 1H), 6.92 – 6.89 (m, 1H), 6.88 (t, *J* = 54.0 Hz, 1H), 4.18 (q, *J* = 6.0 Hz, 1H), 3.81 (s, 3H), 1.52 (d, *J* = 6.0 Hz, 3H); ^{13}C NMR (151 MHz, Chloroform-*d*) δ 161.7, 160.0 (d, *J* = 25.7 Hz), 144.4, 141.1, 133.6, 130.5, 129.0, 127.6, 127.4, 126.9, 116.5, 114.8 (d, *J* = 22.6 Hz), 113.8 (d, *J* = 21.1 Hz), 110.8 (t, *J* = 235.5 Hz), 40.1, 39.6, 21.6; ^{19}F NMR (565 MHz, Chloroform-*d*) δ -110.71 (d, *J* = 305.1 Hz), -111.87 (d, *J* = 310.7 Hz), -115.26. HRMS Calcd. For $[C_{20}H_{19}F_3N_3O]^+$: 374.1475, found: 374.1475.

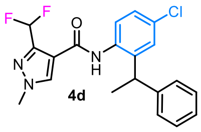

(4d) N-(4-chloro-2-(1-phenylethyl)phenyl)-3-(difluoromethyl)-1-methyl-1H-pyrazole-4-carboxamide: White solid, ^{1}H NMR (600 MHz, Chloroform-*d*) δ 7.74 (d, *J* = 6.0 Hz, 1H), 7.39 (s, 1H), 7.37 – 7.29 (m, 3H), 7.28 – 7.23 (m, 3H), 7.17 (d, *J* = 6.0 Hz, 2H), 6.98 (t, *J* = 54.0 Hz, 1H), 4.22 (q, *J* = 6.0 Hz, 1H), 3.87 (s, 3H), 1.60 (d, *J* = 6.0 Hz, 3H); ^{13}C NMR (151 MHz, Chloroform-*d*) δ 159.6, 144.5 (t, *J* = 27.2 Hz), 144.2, 139.3, 133.3, 133.2, 131.3, 129.1, 128.0, 127.6, 127.2, 127.0, 126.2, 116.4, 110.6 (t, *J* = 235.5 Hz), 40.2, 39.6, 21.7; ^{19}F NMR (565 MHz, Chloroform-*d*) δ -111.26 (d, *J* = 305.1 Hz), -112.48 (d, *J* = 305.1 Hz). HRMS Calcd. For $[C_{20}H_{19}ClF_2N_3O]^+$: 390.1179, found: 390.1180.

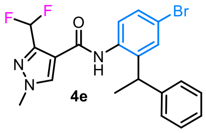

(4e) N-(4-bromo-2-(1-phenylethyl)phenyl)-3-(difluoromethyl)-1-methyl-1H-pyrazole-4-carboxamide: White solid, ^{1}H NMR (600 MHz, Chloroform-*d*) δ 7.72 (d, *J* = 12.0 Hz, 1H), 7.48 (d, *J* = 6.0 Hz, 1H), 7.41 (dd, *J* = 12.0, 6.0 Hz, 1H), 7.36 – 7.32 (m, 3H), 7.28 (d, *J* = 12.0 Hz, 1H), 7.24 (s, 1H), 7.18 (d, *J* = 6.0 Hz, 2H), 6.97 (t, *J* = 54.0 Hz, 1H), 4.22 (q, *J* = 6.0 Hz, 1H), 3.89 (s, 3H), 1.61 (d, *J* = 12.0 Hz, 3H); ^{13}C NMR (151 MHz, Chloroform-*d*) δ 159.6, 144.5 (t, *J* = 25.6 Hz), 144.2, 139.3, 133.9, 133.2, 130.9, 130.3, 129.1, 127.6, 127.1, 126.4, 119.2, 116.5, 110.6 (t, *J* = 234.0 Hz), 40.2, 39.7, 21.7; ^{19}F NMR (565 MHz, Chloroform-*d*) δ -111.26 (d, *J* = 305.1 Hz), -112.42 (d, *J* = 305.1 Hz). HRMS Calcd. For $[C_{20}H_{19}BrF_2N_3O]^+$: 434.0674, found: 434.0675.

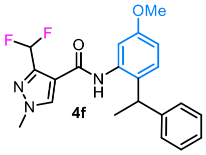

(4f) 3-(difluoromethyl)-N-(5-methoxy-2-(1-phenylethyl)phenyl)-1-methyl-1H-pyrazole-4-carboxamide: White solid, ^{1}H NMR (600 MHz, Chloroform-*d*) δ 7.52 (s, 1H), 7.37 (s, 1H), 7.32 (t, *J* = 12.0 Hz, 2H), 7.26 (t, *J* = 6.0 Hz, 2H), 7.19 (d, *J* = 6.0 Hz, 2H), 7.10 (s, 1H), 7.01 (d, *J* = 54.0 Hz, 1H), 6.79 (dd, *J* = 12.0, 6.0 Hz, 1H), 4.18 (q, *J* = 6.0 Hz, 1H), 3.88 (s, 3H), 3.81 (s, 3H), 1.61 (d, *J* = 6.0 Hz, 3H); ^{13}C NMR (151 MHz, Chloroform-*d*) δ 159.6, 158.7, 145.5, 144.8 (t, *J* = 25.6 Hz), 135.8, 132.8, 129.0, 128.6, 128.5, 127.7, 126.8, 116.9, 111.8, 110.4 (t, *J* = 235.5 Hz), 109.4, 55.5, 39.8, 39.6, 22.0; ^{19}F NMR (565 MHz, Chloroform-*d*) δ -113.62 (d, *J* = 305.1 Hz), -115.01 (d, *J* = 305.1 Hz). HRMS Calcd. For $[C_{21}H_{22}F_2N_3O_2]^+$: 386.1675, found: 386.1675.

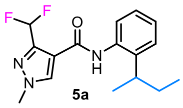

(5a) N-(2-(sec-butyl)phenyl)-3-(difluoromethyl)-1-methyl-1H-pyrazole-4-carboxamide: White solid, ^{1}H NMR (600 MHz, Chloroform-*d*) δ 8.08 (s, 1H), 7.93 (s, 1H), 7.66 (d, *J* = 6.0 Hz, 1H), 7.28 (dd, *J* = 12.0, 6.0 Hz, 1H), 7.23 – 7.19 (m, 2H), 6.93 (t, *J* = 54.0 Hz, 1H), 3.83 (s, 3H), 2.89 – 2.84 (m, 1H), 1.64 – 1.53 (m, 2H), 1.20 (d, *J* = 6.0 Hz, 3H), 0.82 (t, *J* = 12.0 Hz, 3H); ^{13}C NMR (151 MHz, Chloroform-*d*) δ 160.1, 142.8 (t, *J* = 27.1 Hz), 141.2, 135.8, 134.3, 126.6, 126.3, 126.2, 125.9, 116.8, 113.4, 111.9,

110.3, 39.5, 34.7, 30.4, 21.0, 12.0; ^{19}F NMR (565 MHz, Chloroform-*d*) δ -108.70. HRMS Calcd. For $[C_{16}H_{20}F_2N_3O]^+$: 308.1569, found: 308.1570.

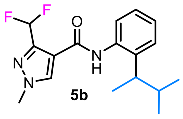

5b

(5b) 3-(difluoromethyl)-1-methyl-N-(2-(3-methylbutan-2-yl)phenyl)-1H-pyrazole-4-carboxamide: White solid, ^{1}H NMR (600 MHz, Chloroform-*d*) δ 8.04 (s, 1H), 7.96 (s, 1H), 7.65 (s, 1H), 7.28 – 7.25 (m, 1H), 7.24 – 7.20 (m, 2H), 6.93 (t, *J* = 54.0 Hz, 1H), 3.87 (s, 3H), 2.72 – 2.67 (m, 1H), 1.82 – 1.75 (m, 1H), 1.20 (d, *J* = 6.0 Hz, 3H), 0.95 (d, *J* = 6.0 Hz, 3H), 0.74 (d, *J* = 6.0 Hz, 3H); ^{13}C NMR (151 MHz, Chloroform-*d*) δ 160.0, 142.7 (t, *J* = 30.2 Hz), 141.1, 136.0, 134.3, 127.0, 126.4, 126.1, 126.0, 116.9, 112.0 (t, *J* = 232.5 Hz), 39.8, 39.5, 34.0, 21.3, 19.9, 18.4; ^{19}F NMR (565 MHz, Chloroform-*d*) δ -108.25. HRMS Calcd. For $[C_{17}H_{22}F_2N_3O]^+$: 322.1725, found: 322.1728.

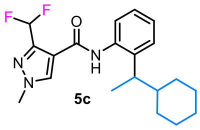

5c

(5c) N-(2-(1-cyclohexylethyl)phenyl)-3-(difluoromethyl)-1-methyl-1H-pyrazole-4-carboxamide: White solid, ^{1}H NMR (600 MHz, Chloroform-*d*) δ 7.98 (d, *J* = 18.0 Hz, 2H), 7.66 (s, 1H), 7.26 – 7.24 (m, 1H), 7.23– 7.19 (m, 2H), 6.90 (t, *J* = 54.0 Hz, 1H), 3.90 (s, 1H), 2.73 – 2.69 (m, 1H), 1.91 (d, *J* = 18.0 Hz, 1H), 1.74 (d, *J* = 12.0 Hz, 1H), 1.62 – 1.58 (m, 2H), 1.41 (d, *J* = 12.0 Hz, 2H), 1.19 (d, *J* = 6.0 Hz, 3H), 1.12 – 1.04 (m, 2H), 0.97 – 0.90 (m, 1H), 0.84 – 0.77 (m, 1H); ^{13}C NMR (151 MHz, Chloroform-*d*) δ 159.9, 142.6 (t, *J* = 30.2 Hz), 140.8, 136.2, 134.4, 127.2, 126.4, 126.0, 125.8, 117.0, 112.1 (t, *J* = 232.5 Hz), 43.8, 39.6, 38.8, 31.4, 30.3, 26.6, 18.6; ^{19}F NMR (565 MHz, Chloroform-*d*) δ -107.68 (d, *J* = 305.1 Hz), -108.27 (d, *J* = 305.1 Hz). HRMS Calcd. For $[C_{20}H_{26}F_2N_3O]^+$: 362.2038, found: 362.2040.

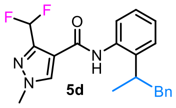

5d

(5d) 3-(difluoromethyl)-1-methyl-N-(2-(1-phenylpropan-2-yl)phenyl)-1H-pyrazole-4-carboxamide: White solid, ^{1}H NMR (600 MHz, Chloroform-*d*) δ 7.70 (s, 1H), 7.55 (d, *J* = 18.0 Hz, 2H), 7.40 (d, *J* = 12.0 Hz, 1H), 7.28 (t, *J* = 6.0 Hz, 1H), 7.22 (t, *J* = 12.0 Hz, 1H), 7.20 (t, *J* = 6.0 Hz, 2H), 7.16 (t, *J* = 6.0 Hz, 1H), 7.03 (d, *J* = 6.0 Hz, 2H), 6.99 (t, *J* = 54.0 Hz, 1H), 3.85 (s, 3H), 3.26 (q, *J* = 6.0 Hz, 1H), 2.86 (ddd, *J* = 42.0, 12.0, 6.0 Hz, 2H), 1.25 (d, *J* = 6.0 Hz, 3H); ^{13}C NMR (101 MHz, Chloroform-*d*) δ 160.2, 140.9, 140.5, 134.6, 134.0, 129.1, 128.3, 126.9, 126.6, 126.5, 126.4, 126.1, 116.6, 111.2 (t, *J* = 234.0 Hz), 44.3, 39.5, 35.3, 20.9; ^{19}F NMR (377 MHz, Chloroform-*d*) δ -109.84. HRMS Calcd. For $[C_{21}H_{22}F_2N_3O]^+$: 370.1725, found: 370.1726.

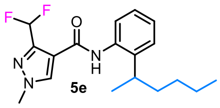

5e

(5e) 3-(difluoromethyl)-N-(2-(hexan-2-yl)phenyl)-1-methyl-1H-pyrazole-4-carboxamide: White solid, ^{1}H NMR (600 MHz, Chloroform-*d*) δ 8.06 (s, 1H), 7.95 (s, 1H), 7.67 (s, 1H), 7.30 – 7.26 (m, 1H), 7.24 – 7.20 (m, 2H), 6.92 (t, *J* = 54.0 Hz, 1H), 3.85 (s, 3H), 2.97 – 2.91 (m, 1H), 1.62 – 1.50 (m, 2H), 1.26 – 1.22 (m, 3H), 1.19 (d, *J* = 6.0 Hz, 3H), 1.17 – 1.10 (m, 1H), 0.82 (t, *J* = 6.0 Hz, 3H); ^{13}C NMR (151 MHz, Chloroform-*d*) δ 158.9, 141.6 (t, *J* = 30.2 Hz), 140.2, 134.9, 133.0, 125.4, 125.2, 125.0, 124.7,

115.7, 110.8 (t, J = 231.0 Hz), 38.4, 36.1, 31.9, 28.6, 21.7, 20.4, 12.9; ^{19}F NMR (565 MHz, Chloroform-*d*) δ -108.70. HRMS Calcd. For $[C_{18}H_{24}F_2N_3O]^+$: 336.1882, found: 336.1883.

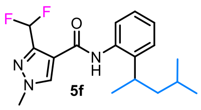

5f

(5f) 3-(difluoromethyl)-1-methyl-N-(2-(4-methylpentan-2-yl)phenyl)-1H-pyrazole-4-carboxamide: White solid, ^{1}H NMR (600 MHz, Chloroform-*d*) δ 8.15 (s, 1H), 7.88 (s, 1H), 7.59 (d, J = 6.0 Hz, 1H), 7.30 (d, J = 6.0 Hz, 1H), 7.26 – 7.18 (m, 2H), 6.95 (t, J = 54.0 Hz, 1H), 3.78 (s, 3H), 3.09 – 3.03 (m, 1H), 1.52 (m, 1H), 1.47 (dt, J = 12.0, 6.0 Hz, 1H), 1.42 – 1.37 (m, 1H), 1.15 (d, J = 6.0 Hz, 3H), 0.80 (d, J = 6.0 Hz, 6H); ^{13}C NMR (151 MHz, Chloroform-*d*) δ 160.2, 143.1 (t, J = 27.1 Hz), 141.9, 135.6, 134.1, 126.7, 126.5, 126.3, 126.1, 116.6, 111.7 (t, J = 232.5 Hz), 46.6, 39.4, 30.7, 25.6, 22.7, 22.6, 21.9; ^{19}F NMR (565 MHz, Chloroform-*d*) δ -109.06. HRMS Calcd. For $[C_{18}H_{24}F_2N_3O]^+$: 336.1882, found: 336.1883.

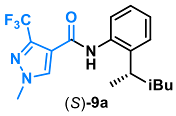

(*S*)-9a

(9a) (*S*)-1-methyl-N-(2-(4-methylpentan-2-yl)phenyl)-3-(trifluoromethyl)-1H-pyrazole-4-carboxamid: White solid, ^{1}H NMR (600 MHz, Chloroform-d) δ 8.03 (s, 1H), 7.63 (d, J = 24.0 Hz, 2H), 7.32 – 7.28 (m, 1H), 7.23 (m, 2H), 3.96 (s, 3H), 2.99 (q, J = 6.0 Hz, 1H), 1.57 – 1.51 (m, 2H), 1.49 – 1.42 (m, 1H), 1.18 (d, J = 6.0 Hz, 3H), 0.82 (d, J = 6.0 Hz, 6H); ^{13}C NMR (101 MHz, Chloroform-*d*) δ 159.3, 141.2, 136.4, 133.8, 126.9, 126.6, 126.3, 125.9, 119.9 (t, J = 269.6 Hz), 46.6, 39.9, 30.8, 25.6, 22.7, 22.6, 22.0. ^{19}F NMR (377 MHz, Chloroform-*d*) δ -59.44. HRMS Calcd. For $[C_{18}H_{23}F_3N_3O]^+$: 354.1788, found: 354.1789.

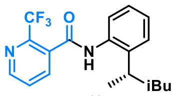

(*S*)-9b

(9b) (*S*)-N-(2-(4-methylpentan-2-yl)phenyl)-2-(trifluoromethyl)nicotinamide: Light yellow solid, ^{1}H NMR (600 MHz, Chloroform-*d*) δ ^{1}H NMR (600 MHz, Chloroform-d) δ 8.8 (d, J = 6.0 Hz, 1H), 8.01 (d, J = 6.0 Hz, 1H), 7.71 (d, 1H), 7.61 (d, J = 6.0 Hz, 1H), 7.35 (s, 1H), 7.32 – 7.28 (m, 1H), 7.29 – 7.23 (m, 2H), 2.99 – 2.93 (m, 1H), 1.54 – 1.48 (m, 2H), 1.45 – 1.37 (m, 1H), 1.20 (d, J = 6.0 Hz, 3H), 0.82 (d, J = 12.0, 6.0 Hz, 6H); ^{13}C NMR (151 MHz,) δ 164.54, 150.48, 144.17 (q, J = 34.8 Hz), 140.97, 137.33, 133.36, 132.21, 126.75, 126.*66* (d, J = 5.5 Hz), 125.38, 124.66 – 118.46 (m), 46.91, 31.03, 25.67, 22.94, 22.64, 21.78; ^{19}F NMR (565 MHz, Chloroform-*d*) δ -63.65. HRMS Calcd. For $[C_{19}H_{22}F_3N_2O]^+$: 351.1679, found: 351.1680.

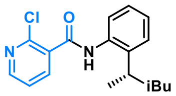

(*S*)-9c

(9c) (*S*)-2-chloro-N-(2-(4-methylpentan-2-yl)phenyl)nicotinamide: White solid, ^{1}H NMR (600 MHz, Chloroform-*d*) δ 8.49 (d, J = 6.0 Hz, 1H), 8.22 (d, J = 6.0 Hz, 1H), 8.14 (s, 1H), 7.74 (d, J = 12.0 Hz, 1H), 7.40-7.39 (m, 1H), 7.32 (d, J = 6.0 Hz, 1H), 7.26 – 7.23 (m, 2H), 3.10 – 3.04 (m, 1H), 1.57 – 1.49 (m, 2H), 1.49 – 1.42 (m, 1H), 1.22 (d, J = 6.0 Hz, 3H), 0.84 (t, J = 6.0 Hz, 6H); ^{13}C NMR (151 MHz, Chloroform-*d*) δ 163.2, 151.3, 146.9, 141.0, 140.3, 133.6, 131.5, 127.1, 126.6, 126.5, 125.3, 123.1, 46.8, 31.1, 25.6, 23.0, 22.7, 21.9. HRMS Calcd. For $[C_{18}H_{22}ClN_2O]^+$: 317.1415, found: 317.1418.

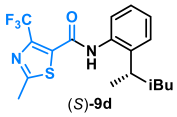

(*S*)-**9d**

(9d) (*S*)-2-methyl-N-(2-(4-methylpentan-2-yl)phenyl)-4-(trifluoromethyl)thiazole-5-carboxamide: Light yellow solid, ^{1}H NMR (600 MHz, Chloroform-*d*) δ 7.68 (d, *J* = 6.0 Hz, 1H), 7.64 (s, 1H), 7.30 (d, *J* = 6.0 Hz, 1H), 7.28–7.23 (m, 2H), 2.96 – 2.93 (m, 1H), 2.77 (s, 3H), 1.55 – 1.48 (m, 2H), 1.46–1.41 (m, 1H), 1.20 (d, *J* = 6.0 Hz, 3H), 0.84 (d, *J* = 6.0 Hz, 6H); ^{13}C NMR (151 MHz, Chloroform-*d*) δ 168.7, 157.5, 140.9, 136.0, 133.2, 127.4, 126.7, 126.6, 125.3, 120.4 (d, *J* = 271.9 Hz), 46.7, 31.0, 25.6, 22.9, 22.6, 21.8, 19.4; ^{19}F NMR (377 MHz, Chloroform-*d*) δ -59.21. HRMS Calcd. For $[C_{18}H_{22}F_3N_2OS]^+$: 371.1399, found: 371.1402.

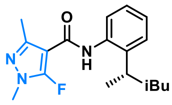

(*S*)-**9e**

^{1}H NMR (600 MHz, Chloroform-*d*) δ 7.80 (d, *J* = 12.0 Hz, 1H), 7.35 (s, 1H), 7.27 – 7.25 (m, 1H), 7.23 – 7.17 (m, 2H), 3.75 (s, 3H), 3.0 – 2.94 (m, 1H), 2.49 (s, 3H), 1.56 – 1.49 (m, 2H), 1.46 – 1.40 (m, 1H), 1.22 (d, *J* = 6.0 Hz, 3H), 0.85 (q, *J* = 3.0 Hz, 6H); ^{13}C NMR (151 MHz, Chloroform-*d*) δ 159.5, 151.4 (d, *J* = 279.3 Hz), 150.2 (d, *J* = 9.0 Hz), 139.6, 134.0, 126.4, 126.3, 126.0, 124.9, 96.0, 46.8, 34.1, 30.9, 25.6, 22.9, 22.6, 21.6, 14.9; ^{19}F NMR (565 MHz, Chloroform-*d*) δ -127.71.

5. Antifungal activity investigation

5.1 Biological assay

In vitro antifungal tests of chiral SDHIs were conducted against seven pathogenic fungi: *Botrytis cinerea* (*B. c*, B05.10), *Sclerotinia sclerotiorum* (*S. s*, 1980 UF-70), *Rhizoctonia solani* (*R. s*, WH-1), *Fusarium graminearum* (*F. g*, PH-1), *Pestalotiopsis tea* (*P. t*, Pt1), *Alternaria alternata* (*A. a*) and *Corynespora cassiicola* (*C. c*).[5] These fungi were cultivated on a potato dextrose agar (PDA) medium at 28 ℃ under in dark. The commercial fungicide Boscalid served as a positive control in the experiments. All trials were conducted three times to ensure the reliability of the results. Enantiopure SDHIs were precisely weighed and dissolved in chromatography-grade DMSO to prepare a solution with a uniform concentration. In each experiment, a predetermined volume of the compound solution was mixed with the growth medium to formulate the medicated medium. After achieving robust growth, the fungi were cut into small blocks approximately 1mm in diameter and were inoculated at the center of the medium. A control containing DMSO at an equivalent concentration was used to assess the impact of the SDHIs. When the control reached about 80% coverage of the medium's diameter, the diameter of the fungal growth in all media was measured, and the inhibition rate was calculated accordingly.

The well-established method was employed to assess the *in vivo* antifungal activity of novel SDHIs.[6] A range of concentrations for compounds (*R*)-**5f** or (*S*)-**5f**, and the positive control Boscalid were evenly applied to living specimens. Initially, tomato fruits and rape leaves were disinfected with 75% ethanol for 30 seconds, followed by rinsing under running water and air-drying. Subsequently, wounds were inflicted on the surfaces of these leaves and fruits. Spore suspensions of *Botrytis cinerea* and *Fusarium graminearum* were prepared and diluted with compound stock solutions to achieve the desired test concentrations. These mixtures were then injected into the wounds of tomatoes and wheat ears (10 microliters each). For the wounds on rape leaves and rice leaves, 10 microliters of the compound stock solution at the test concentration were injected, and mycelial discs of *Sclerotinia sclerotiorum* and *Rhizoctonia solani*, each with a diameter of 5 millimeters, were placed onto the wound sites with the mycelial side facing down. The inoculated rape leaves, tomato fruits, and rice leaves were incubated at 25 ℃ for three to four days, while the infected wheat ears were cultured *in vivo* for two weeks. Each experiment was conducted in triplicate. The lesion areas were statistically analyzed using ImageJ software.

5.2 Staining assay

The staining of conidia was performed according to the method described in references.[7-9] A solution consisting of 60 mg/mL potassium iodide (KI) and 10 mg/mL iodine (I_2) was utilized for glycogen staining. This staining solution was added to conidia suspensions that had been treated with SDHIs. After incubation for 5 minutes, the samples were examined under a microscope.[10]

5.3 Scanning electron microscopy (SEM) measurement

The sample collection procedure adhered to the method previously outlined.[11] Sterile cellophane was laid onto the surface of the culture medium, both with and without enantiomers of **5f** and Boscalid at a concentration of 0.6 μM. Subsequently, the *Sclerotinia sclerotiorum* mycelium was inoculated onto this setup and incubated at 28 ℃ for a period of 3 days. The cellophane containing the mycelium was then treated with 2.5% glutaraldehyde, removed from the medium and freeze-dried to preserve the structural integrity, and subsequently subjected to SEM analysis.

5.4 Surface plasmon resonance (SPR) analysis

The interaction assays of the enantiomers of **5f** with SDH were performed as described previously.[12,13]

5.4.1 Chip Preparation: The activator is prepared by mixing 400 mM EDC and 100 mM NHS immediately prior to injection. The CM5 sensor chip is activated for 420 s with the mixture at a flow rate of 10 μL/min.

5.4.2 Ligand immobilization: Dilute CII to 50 μg/mL in immobilization buffer, then injected to sample channel (Fc2) at a flow rate of 10 μL/min, and typically result in immobilization levels of 13300 RU, the reference channel (Fc1) does not need ligand immobilization step. The chip is deactivated by 1 M Ethanolamine hydrochloride at a flow rate of 10 μL/min for 420 s.

5.4.3 Running analyte by multi-cycle method: (*R*)-**5f** and (*S*)-**5f** were diluted with the same analyte buffer to six concentrations (25, 12.5, 6.25, 3.125, 1.5625, 0.78 μM). (*R*)-**5f**, (*S*)-**5f** is injected to channel Fc1-Fc2 at a flow rate of 30 μL/min for an association phase of 120 s, followed by 300 s dissociation. The association and dissociation process are both handled in the analyte buffer. Repeat 7 cycles of analyte addition according to analyte concentrations in ascending order. After each cycle of interaction analysis, the analyte will dissociate naturally.

5.5 Modeling and molecular docking

The structures of (*S*)-**5f** and (*R*)-**5f** were drawn by using ChemDraw 20.0. Molecular docking experiments were conducted as previously described.[14] The molecular model was pre-optimized using the MM2 molecular force field in ChemDraw 3D.[15] Protein-molecule interactions were performed using auto-dock. 2D protein-ligand interaction diagram was produced using LigPlot$^+$ 2.2 software,[16] and 3D protein-ligand interaction diagram was generated using Pymol 2.5 software.

6. Antifungal experimental results

Table S1. Preliminary antifungal results against *Botrytis cinerea* (20 μM)

SDHI	Inhibition rate	SDHI	Inhibition rate	SDHI	Inhibition rate
Blank control	0	(*R*)-**3k**	63.59 ±0.84	(*R*)-**4c**	42.78 ±0.84
DMSO	0.497 ±0.13	(*S*)-**3k**	69.01 ±1.27	(*S*)-**4c**	43.69 ±1.10
Boscalid	81.23 ±0.63	*rac*-**3k**	64.76 ±0.76	*rac*-**4c**	37.47 ±0.289
(*R*)-**3a**	43.01 ±0.55	(*R*)-**3l**	61.51 ±0.69	(*R*)-**4d**	41.88 ±0.63
(*S*)-**c**	45.04 ±0.55	(*S*)-**3l**	63.97 ±0.60	(*S*)-**4d**	36.68 ±1.27
rac-**3a**	40.34 ±0.50	*rac*-**3l**	59.15 ±0.28	*rac*-**4d**	39.84 ±1.263
(*R*)-**3b**	35.78 ±1.51	(*R*)-**3m**	47.76 ±0.55	(*R*)-**4e**	38.26 ±1.46
(*S*)-**3b**	23.52 ±1.588	(*S*)-**3m**	47.53 ±0.63	(*S*)-**4e**	38.48 ±0.31
rac-**3b**	25.84 ±1.76	*rac*-**3m**	45.67 ±0.76	*rac*-**4e**	30.91 ±1.04
(*R*)-**3c**	58.57 ±1.38	(*R*)-**3n**	48.77 ±2.42	(*R*)-**4f**	23.33 ±0.95
(*S*)-**3c**	57.35 ±0.60	(*S*)-**3n**	46.81 ±2.10	(*S*)-**4f**	37.13 ±0.84
rac-**3c**	57.22 ±0.76	*rac*-**3n**	52.84 ±0.76	*rac*-**4f**	26.04 ±0.50
(*R*)-**3d**	48.66 ±0.31	(*R*)-**3o**	42.64 ±1.58	(*R*)-**5a**	66.91 ±0.60
(*S*)-**3d**	52.96 ±1.15	(*S*)-**3o**	39.46 ±1.83	(*S*)-**5a**	74.75 ±0.91
rac-**3d**	48.95 ±0.50	*rac*-**3o**	38.39 ±0.28	*rac*-**5a**	64.73 ±1.26
(*R*)-**3e**	41.65 ±3.45	(*R*)-**3p**	64.95 ±0.34	(*R*)-**5b**	65.93 ±0.91
(*S*)-**3e**	46.40 ±0	(*S*)-**3p**	56.61 ±0.60	(*S*)-**5b**	78.43 ±0.34
rac-**3e**	35.99 ±0.57	*rac*-**3p**	58.89 ±0.76	*rac*-**5b**	68.22 ±1.15
(*R*)-**3f**	24.92 ±0.84	(*R*)-**3q**	65.19 ±0.34	(*R*)-**5c**	66.66 ±0.34
(*S*)-**3f**	28.31 ±1.27	(*S*)-**3q**	55.14 ±1.58	(*S*)-**5c**	73.28 ±1.24
rac-**3f**	25.37 ±1.04	*rac*-**3q**	57.46 ±1.04	*rac*-**5c**	67.81 ±0.28
(*R*)-**3g**	27.86 ±1.27	(*R*)-**3r**	55.39 ±0.69	(*R*)-**5d**	63.72 ±0.69
(*S*)-**3g**	17.23 ±1.91	(*S*)-**3r**	55.39 ±1.24	(*S*)-**5d**	63.48 ±1.24
rac-**3g**	20.45 ±1.26	*rac*-**3r**	53.21 ±1.61	*rac*-**5d**	60.02 ±1.50
(*R*)-**3h**	51.83 ±1.46	(*R*)-**3s**	6.372 ±1.24	(*R*)-**5e**	69.85 ±0.60
(*S*)-**3h**	58.38 ±0.84	(*S*)-**3s**	12.00 ±1.73	(*S*)-**5e**	72.05 ±0
rac-**3h**	48.13 ±0.57	*rac*-**3s**	8.97 ±1.07	*rac*-**5e**	65.14 ±1.04
(*R*)-**3i**	61.78 ±0.63	(*R*)-**4a**	24.69 ±0.55	(*R*)-**5f**	68.62 ±0.34
(*S*)-**3i**	66.75 ±0.95	(*S*)-**4a**	32.15 ±1.91	(*S*)-**5f**	81.37 ±1.24
rac-**3i**	57.76 ±0.76	*rac*-**4a**	26.51 ±0.50	*rac*-**5f**	71.29 ± 1.614
(*R*)-**3j**	49.34 ±0.846	(*R*)-**4b**	32.38 ±0.84		
(*S*)-**3j**	52.96 ±1.15	(*S*)-**4b**	37.58 ±0.55		
rac-**3j**	48.65 ±0.50	*rac*-**4b**	31.07 ±1.61		

Table S2. Broad-spectrum antifungal activities against seven pathogenic fungi (20 μM)

SDHI	Fungi species						
	B. c	*S. s*	*R. s*	*F. g*	*P. t*	*A. a*	*C. c*
**Boscalid**	81.23 ±0.64	96.25 ±0.17	80.00 ±1.01	78.31 ±1.79	16.88 ±2.06	83.2 ±0.65	91.96 ±0.65
(*R*)-**5b**	65.93 ±0.91	75.12 ±2.66	57.38 ±1.78	80.13 ±1.11	25.80 ±2.37	51.46 ±0.37	76.15 ±1.36
(*S*)-**5b**	78.43 ±0.34	96.25 ±0.17	89.04 ±0.89	81.05 ±0.32	19.34 ±3.71	83.73 ±0.37	92.49 ±1.00
(*R*)-**5c**	66.66 ± 0.34	61.11 ±2.39	58.57 ±1.74	79.68 ±0.64	46.30 ±0.83	53.86 ±0.75	45.87 ±0.37
(*S*)-**5c**	73.28 ±1.24	93.84 ±0.78	88.09 ±0.33	81.73 ±1.29	55.43 ±0.83	67.46 ±0.75	84.99 ±1.00
(*R*)-**5e**	69.85 ±0.60	85.74 ±0.34	88.09 ±1.21	79.90 ±1.79	37.16 ±1.89	63.73 ±0.75	80.17 ±3.03
(*S*)-**5e**	72.05 ±0.00	93.23 ±0.34	89.04 ±1.21	80.36 ±1.79	43.62 ±3.28	63.73 ±0.37	82.04 ±1.65
(*R*)-**5f**	68.62 ±0.34	77.05 ±0.90	83.57 ±0.58	78.76 ±0.55	30.92 ±0.63	56.00 ±1.30	62.75 ±7.07
(*S*)-**5f**	81.37 ±1.24	96.49 ±0.17	84.28 ±1.01	83.10 ±0.85	43.19 ±1.09	85.33 ±0.37	87.13 ±2.86

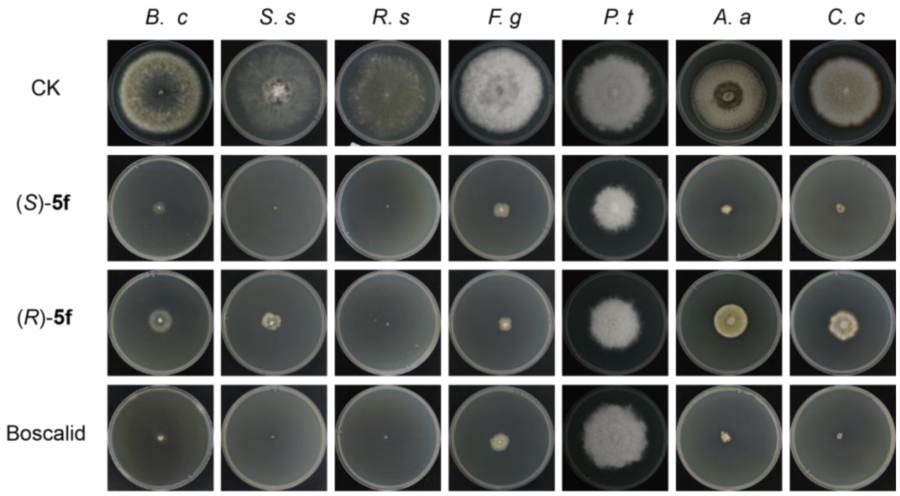

Figure S1. Broad-spectrum antifungal experiments (20 μM)

Table S3. The EC_{50} values against four pathogenic fungi

Fungi	EC_{50} (μM)									
	Boscalid	(*R*)-**5b**	(*S*)-**5b**	(*R*)-**5c**	(*S*)-**5c**	(*R*)-**5e**	(*S*)-**5e**	(*R*)-**5f**	(*S*)-**5f**	*rac*-**5f**
B. c	1.36	13.39	1.26	19.28	6.24	10.28	5.19	36.70	0.48	1.65
S. s	0.20	1.48	0.10	1.07	0.19	0.56	0.21	0.38	0.06	0.19
R. s	1.22	12.20	0.54	28.42	0.10	0.28	0.04	0.24	0.02	0.06
F. g	1.83	0.48	0.10	0.15	0.14	0.14	0.18	0.42	0.07	0.14

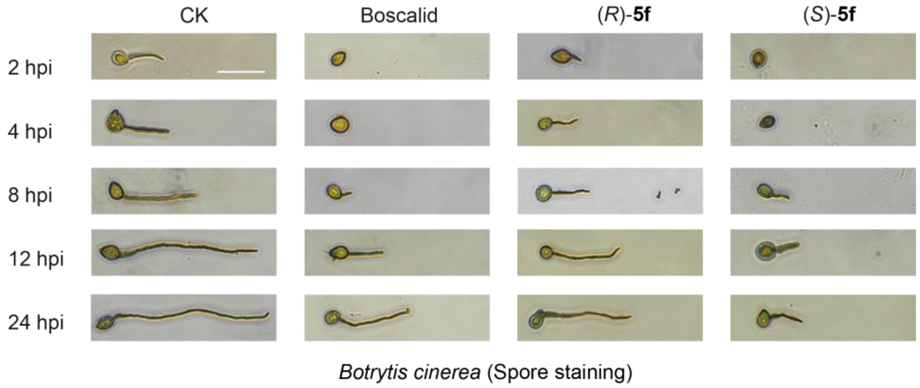

Figure S2. Staining of appressoria treated with enantiomers of **5f** and Boscalid (4 μM)

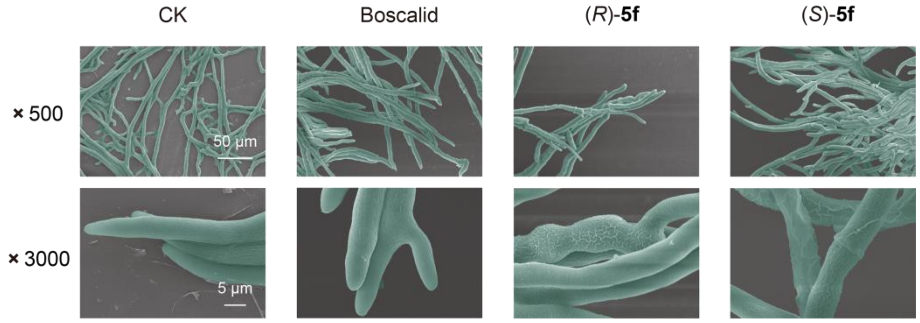

Figure S3. Effects of enantiomers of **5f** and Boscalid on mycelial surface of *Sclerotinia sclerotiorum* (0.6 μM)

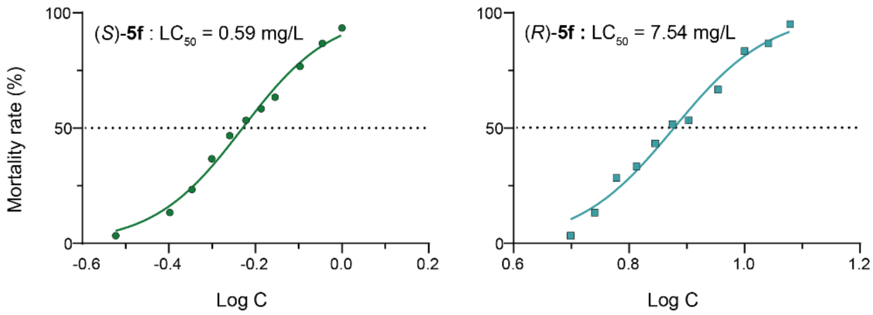

Figure S4. The toxic testing for the enantiomer of **5f** against zebrafish

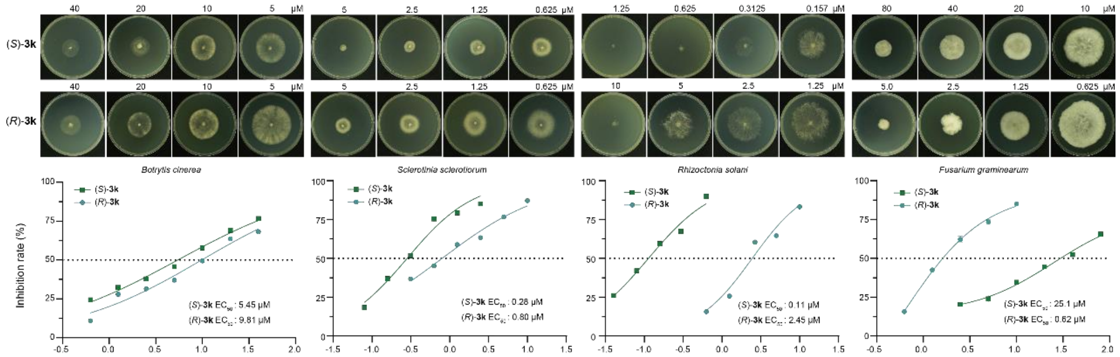

Figure S5. Inhibitory effects of **3k** on *B. c*, *S. s*, *R. s*, *F. g*

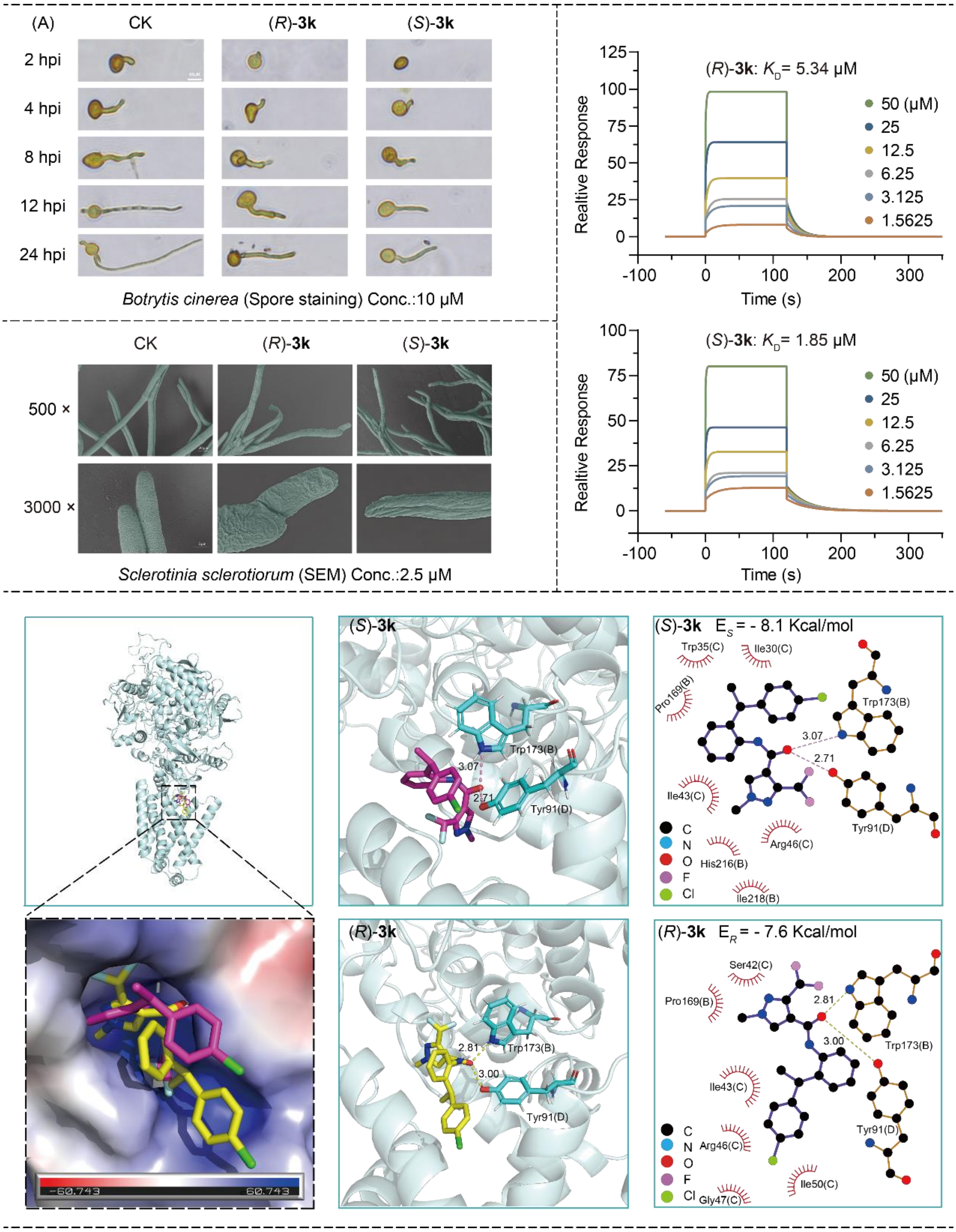

Figure S6. Investigation of the biological mechanism for the enantiomers of **3k**

7. Molecular dynamic simulation

Materials and Methods:

Molecular dynamics simulations (MD) were conducted based on the protein-ligand complex model obtained from molecular docking, which is consistent with the aforementioned docking results.

The enantiomers (R)-5f and (S)-5f were subjected to hydrogenation processing using GaussView 6.0 software,[17] followed by RESP2(0.5) molecular charge fitting calculations.[18] The specific procedure involved optimizing the geometry and performing frequency analysis of the hydrogenated small molecule models at the B3LYP-D3(BJ)/Def2-SVP level to ensure that the obtained lowest energy conformations had no imaginary frequencies.[19-21] Subsequently, the models were analyzed at the M06-2X-D3/Def2-TZVPP level to compute the energies in both vacuum and aqueous solution (using the SMD solvent model).[22, 23] The RESP2(0.5) molecular charges were fitted using the Multiwfn 3.8(dev) and were employed in the subsequent MD simulations.[24] The topology files for molecules were constructed or inferred using Sobtop 1.0(dev3.1) software based on GAFF molecular force field parameters to obtain the relevant force constants.[25, 26]

MD simulations were conducted using the GROMACS 2020.3 software.[27] The protein model of SDH had the A chain, which showed no interaction with molecules during molecular docking, removed in advance to save computational resources.[28] The protein topology and subsequent MD simulations were performed using the Amber14SB_Parmbsc1 force field.[29] The protein-ligand complex model was immersed in a cubic water box with a minimum distance of 1.2 Å from the boundaries, described using the SCP/E three-point water model.[30] Additionally, 0.145 mol/L of chloride ions (Cl^-) and sodium ions (Na^+) were added to the system to balance the charge and simulate a physiological saline environment.

First, the system underwent energy minimization using the steepest descent method with a time step of 1 fs, ensuring that the maximum force (Fmax) was less than 1×10^3 kJ/mol/nm. Subsequently, NVT and NPT equilibrium simulations were performed at 300 K and 1.0 atm for 50,000 steps, with a time step of 2 fs.

Based on such processing, the MD simulations were set to a duration of 100 ns with a time step of 2 fs. By evaluating the root-mean-square deviation (RMSD) of the system, trajectories from 90

to 100 ns were selected for MM-PBSA energy decomposition analysis, which was performed using the gmx_mmpbsa.bsh script.[31]

8. Absolute configuration determination

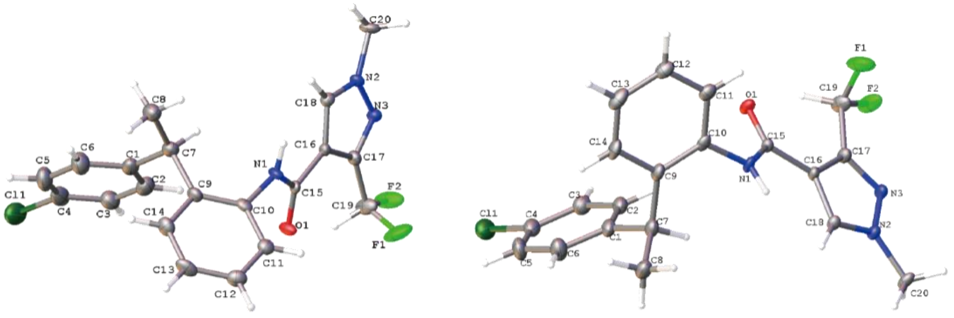

(*R*)-**3k** (*S*)-**3k**

Figure S7. X-ray structure of (*R*)-**3k** and (*S*)-**3k**

Crystal data for (*R*)-**3k**: $C_{20}H_{18}ClF_2N_3O$, Mr = 389.11, T = 100 K, Orthorhombic, space group $C222_1$, *a* = 11.6584(14), *b* = 30.144(4), *c* = 44.779(5) Å, *V* = 15737(3) Å^3, *Z* = 32, Independent reflections = 16077, final R_1 = 0.0891 and wR_2 =0.2345, CCDC 2386657.

Crystal data for (*S*)-**3k**: $C_{20}H_{18}ClF_2N_3O$, Mr = 389.11, T = 100 K, Orthorhombic, space group $C222_1$, *a* = 11.6597(11), *b* = 30.166(3), *c* = 45.051(4) Å, *V* = 15846(3) Å^3, *Z* = 32, Independent reflections = 16357, final R_1 = 0.0718 and wR_2 =0.1945, CCDC 2386656.

9. References

[1] A. Boelke, L. D. Caspers and B. J. Nachtsheim, *Org. Lett.* **2017**, *19*, 5344-5347.

[2] Q. Li, X. Gu, Y. Wei and M. Shi, *Chem. Sci.* **2022**, *13*, 11623-11632.

[3] A. Chatupheeraphat, M. Rueping and M. Magre, *Org. Lett.* **2019**, *21*, 9153-9157.

[4] W. C. de Souza, B. T. Matsuo, P. M. Matos, J. T. M. Correia, M. S. Santos, B. König and M. W. Paixão, *Chem-Eur J.* **2021**, *27*, 3722-3728.

[5] L. Zhang, W. Wu, Z. Li and X. Xu, *J. Agric. Food Chem.* **2023**, *71*, 10575-10589.

[6] W. Wang, J. Wang, F. Wu, H. Zhou, D. Xu and G. Xu, *J. Agric. Food Chem.* **2021**, *69*, 5746-5754.

[7] J. Zhao, P. Sun, Q. Sun, R. Li, Z. Qin, G. Sha, Y. Zhou, R. Bi, H. *Mol. Plant Pathol.* **2022**, *23*, 720-732.

[8] E. Thines, R. W. Weber and N. J. Talbot, *Plant Cell*, **2000**, *12*, 1703-1718.

[9] P. Greenspan, E. P. Mayer and S. D. Fowler, *J. Cell Biol.* **1985**, *100*, 965-973.

[10] M. Ding, N. Wu, Q. Lin, Y. Yan, Y. Yang, G. Tian, L. An and X. Bao, *J. Agric. Food Chem.* **2022**, *70*, 10100-10110.

[11] R. O. Rocha, C. Elowsky, N. T. Pham and R. A. Wilson, *Nat. Microbiol*, **2020**, *5*, 1472-1480.

[12] H. Bonnet, L. Coche-Guérente, E. Defrancq, N. Spinelli, A. Van der Heyden and J. Dejeu, *Anal. Chem.* **2021**, *93*, 4134-4140.

[13] J. Matsui, K. Akamatsu, N. Hara, D. Miyoshi, H. Nawafune, K. Tamaki and N. Sugimoto, *Anal. Chem.* **2005**, *77*, 4282-4285.

[14] G. M. Morris, R. Huey, W. Lindstrom, M. F. Sanner, R. K. Belew, D. S. *J. Comput. Chem.* **2009**, *30*, 2785-2791.

[15] N. L. Allinger, *J. Am. Chem. Soc.* **1977**, *99*, 8127-8134.

[16] R. A. Laskowski and M. B. Swindells, *J. Chem Inf. Model.* **2011**, *51*, 2778-2786.

[17] M. J. Frisch, G. W. Trucks, H. B. Schlegel, G. E. Scuseria, M. A. Robb, J. R. Cheeseman, G. Scalmani, V. Barone, G. A. Petersson, H. Nakatsuji, X. Li, M. Caricato, A. V. Marenich, J. Bloino, B. G. Janesko, R. Gomperts, B. Mennucci, H. P. Hratchian, J. V. Ortiz, A. F. Izmaylov, J. L. Sonnenberg, Williams, F. Ding, F. Lipparini, F. Egidi, J. Goings, B. Peng, A. Petrone, T. Henderson, D. Ranasinghe, V. G. Zakrzewski, J. Gao, N. Rega, G. Zheng, W. Liang, M. Hada, M. Ehara, K. Toyota, R. Fukuda, J. Hasegawa, M. Ishida, T. Nakajima, Y. Honda, O. Kitao, H. Nakai, T. Vreven, K. Throssell, J. A. Montgomery Jr., J. E. Peralta, F. Ogliaro, M. J. Bearpark, J. J. Heyd, E. N. Brothers, K. N. Kudin, V. N. Staroverov, T. A. Keith, R. Kobayashi, J. Normand, K. Raghavachari, A. P. Rendell, J. C. Burant, S. S. Iyengar, J. Tomasi, M. Cossi, J. M. Millam, M. Klene, C. Adamo, R. Cammi, J. W. Ochterski, R. L. Martin, K. Morokuma, O. Farkas, J. B. Foresman, D. J. Fox, Wallingford, CT, 2016.

[18] M. Schauperl, P. S. Nerenberg, H. Jang, L.-P. Wang, C. I. Bayly, D. L. Mobley, M. K. Gilson, *Commun Chem.* **2020**, *3*, 44.

[19] P. J. Stephens, F. J. Devlin, C. F. Chabalowski, M. J. Frisch, *J. Phys. Chem.* **1994**, *98*, 11623-11627.

[20] S. Grimme; J. Antony; S. Ehrlich; H. Krieg, *J. Chem. Phys.* **2010**, *32*, 154104.

[21] F. Weigend, R. Ahlrichs, *Phys. Chem. Chem. Phys.* **2005**, *7*, 3297-3305.

[22] Y. Zhao, D. G. Truhlar, *Theor. Chem. Acc.* **2007**, *120*, 215-241.

[23] A. V. Marenich, C. J. Cramer, D. G. Truhlar, *J. Phys. Chem.* **2009**, *113*, 6378-6396.

[24] T. Lu, F. Chen, *J. Comput. Chem.* **2011**, *33*, 580-592.

[25] J. Wang, R. M. Wolf, J. W. Caldwell, P. A. Kollman, D. A. Case, *J. Comput. Chem.* **2004**, *25*, 1157–1174.

[26] Tian Lu, Sobtop, 1.0(dev3.1), http://sobereva.com/soft/Sobtop. Accessed Feb 24, 2025.
[27] M. J. Abraham, T. Murtola, R. Schulz, S. Páll, J. C. Smith, B. Hess, E. Lindahl, SoftwareX, **2015**, *1-2*, 19-25.
[28] X. L. Zhu, L. Xiong, H. Li, X. Y. Song, J. J. Liu, G. F. Yang, *ChemMedChem*. **2014,** *9*, 1512-1521.
[29] J. Graf, P. H. Nguyen, G. Stock, H. Schwalbe, *J. Am. Chem. Soc.* **2007**, *129*, 1179-1189.
[30] H. J. C. Berendsen, J. R. Grigera, T. P. Straatsma, *J. Phys. Chem.* **1987**, *91*, 6269-6271.
[31] N. Homeyer, H. Gohlke, *Mol. Inform.* **2012**, *31*, 114-122.

10. ^{1}H NMR and ^{13}C NMR spectras

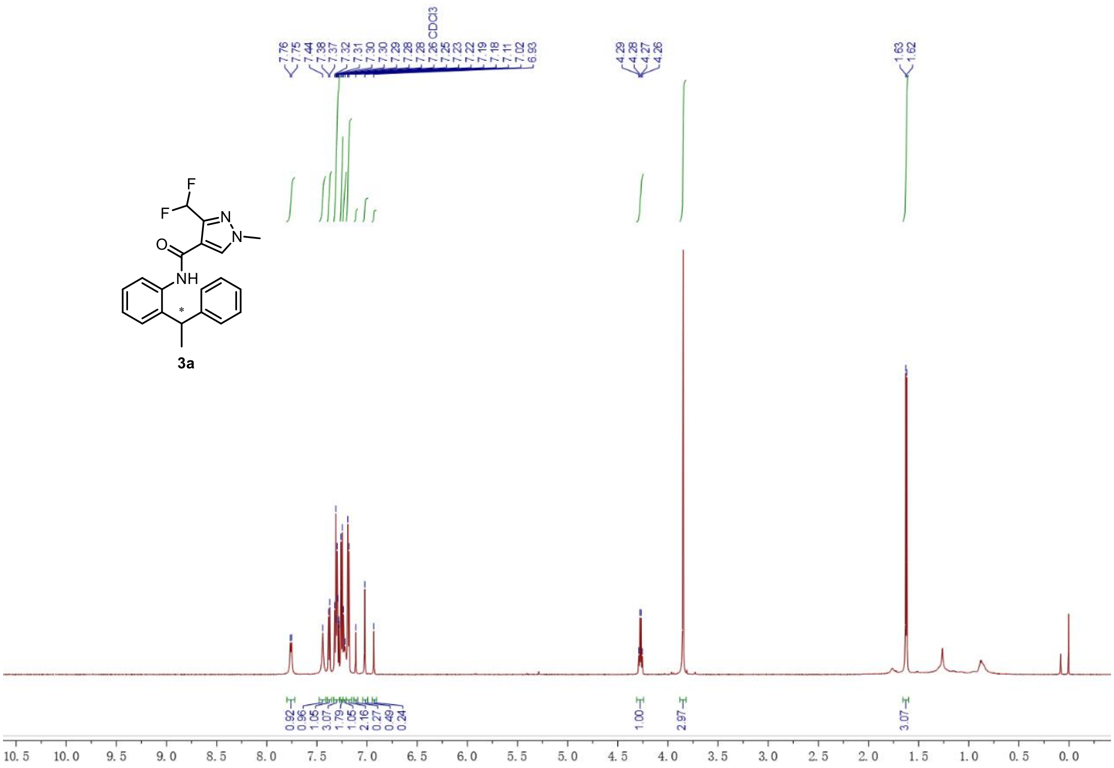

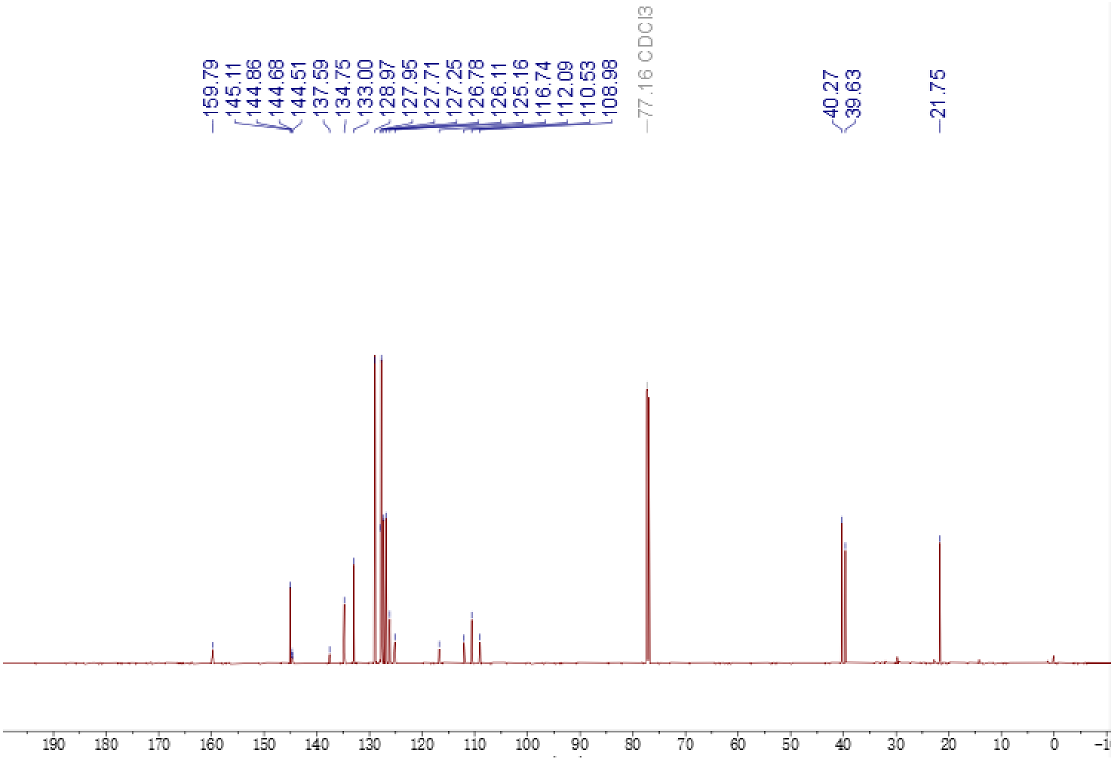

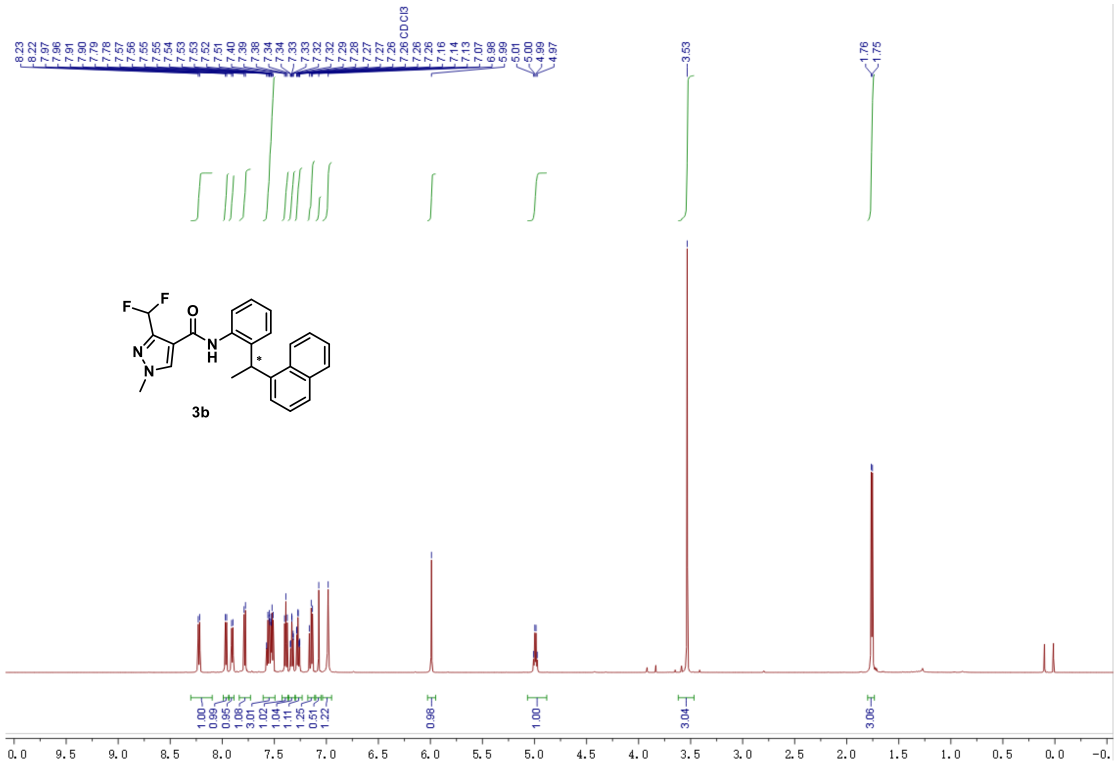
8.23 8.22 7.97 7.96 7.91 7.90 7.79 7.78 7.57 7.56 7.55 7.55 7.54 7.53 7.53 7.52 7.51 7.40 7.39 7.38 7.34 7.34 7.34 7.33 7.33 7.32 7.32 7.29 7.28 7.27 7.27 7.26 7.26 CDCl3 7.26 7.26 7.16 7.14 7.13 7.07 6.98 5.99 5.01 5.00 4.99 4.97
3.53
1.76 1.75
F
F
O
N
N
N
H
*
3b
1.00 0.99 0.95 1.08 3.01 1.02 1.04 1.11 1.25 0.51 1.22
0.98
1.00
3.04
3.06
10.0 9.5 9.0 8.5 8.0 7.5 7.0 6.5 6.0 5.5 5.0 4.5 4.0 3.5 3.0 2.5 2.0 1.5 1.0 0.5 0.0

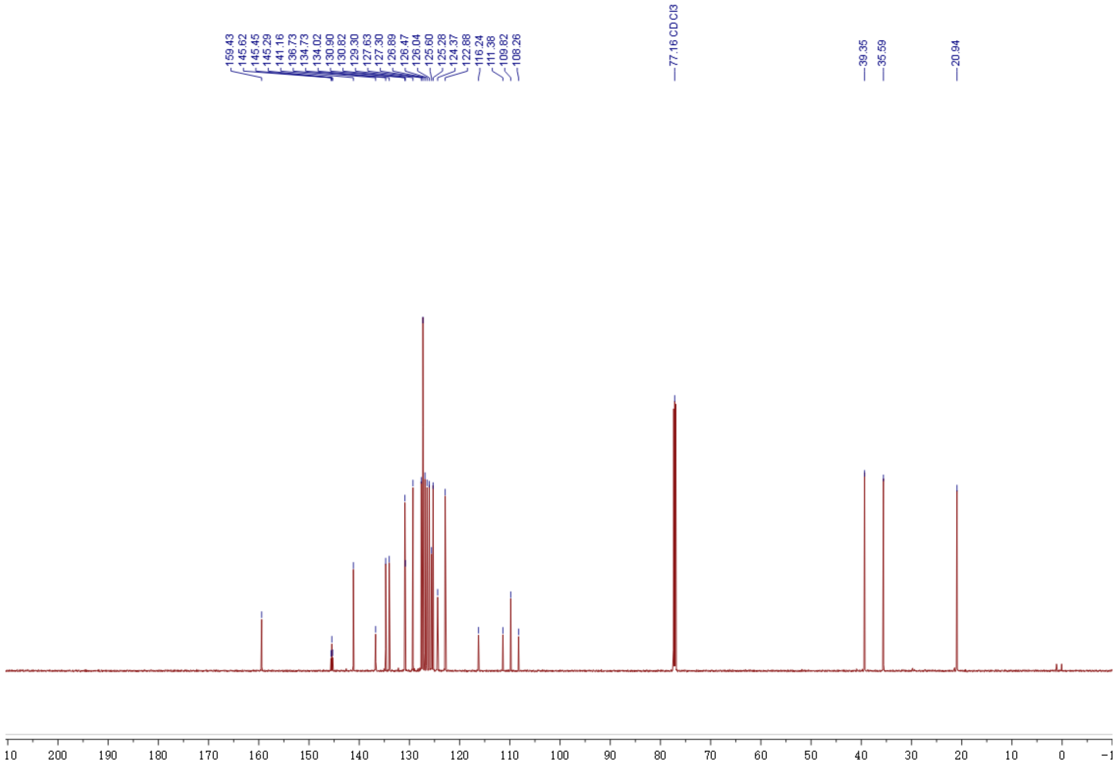
159.43 145.62 145.45 145.29 141.16 136.73 134.73 134.02 130.90 130.82 129.30 127.63 127.30 126.89 126.47 126.04 125.60 125.28 124.37 122.88 116.24 111.38 109.82 108.26
77.16 CDCl3
39.35
35.59
20.94
200 190 180 170 160 150 140 130 120 110 100 90 80 70 60 50 40 30 20 10 0

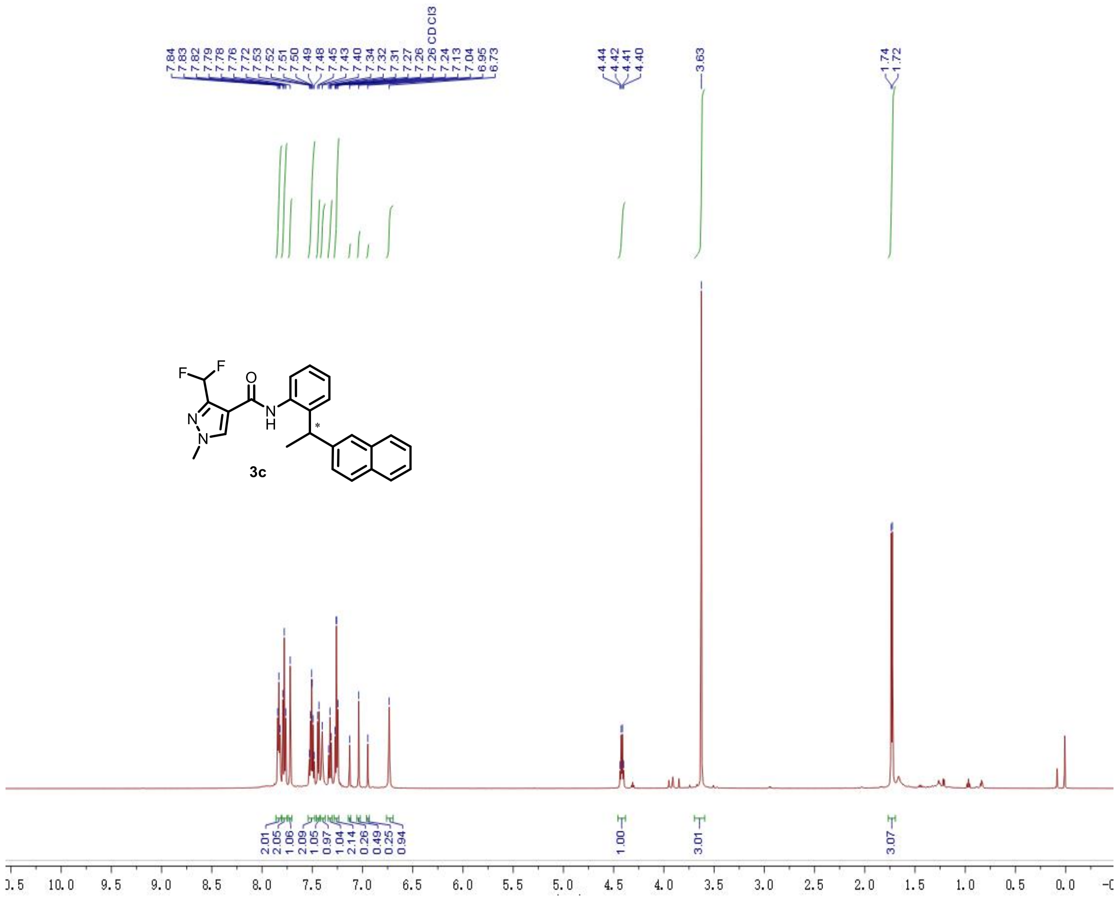
7.84 7.83 7.82 7.79 7.78 7.76 7.72 7.53 7.52 7.51 7.50 7.49 7.48 7.45 7.43 7.40 7.34 7.32 7.31 7.27 7.26 CDCl3 7.24 7.13 7.04 6.95 6.73
4.44 4.42 4.41 4.40
3.63
1.74 1.72
F
F
O
N
H
N
N
*
3c
2.01 2.05 1.06 2.09 1.05 0.97 1.04 2.14 0.26 0.49 0.25 0.94
1.00
3.01
3.07
10.0 9.5 9.0 8.5 8.0 7.5 7.0 6.5 6.0 5.5 5.0 4.5 4.0 3.5 3.0 2.5 2.0 1.5 1.0 0.5 0.0

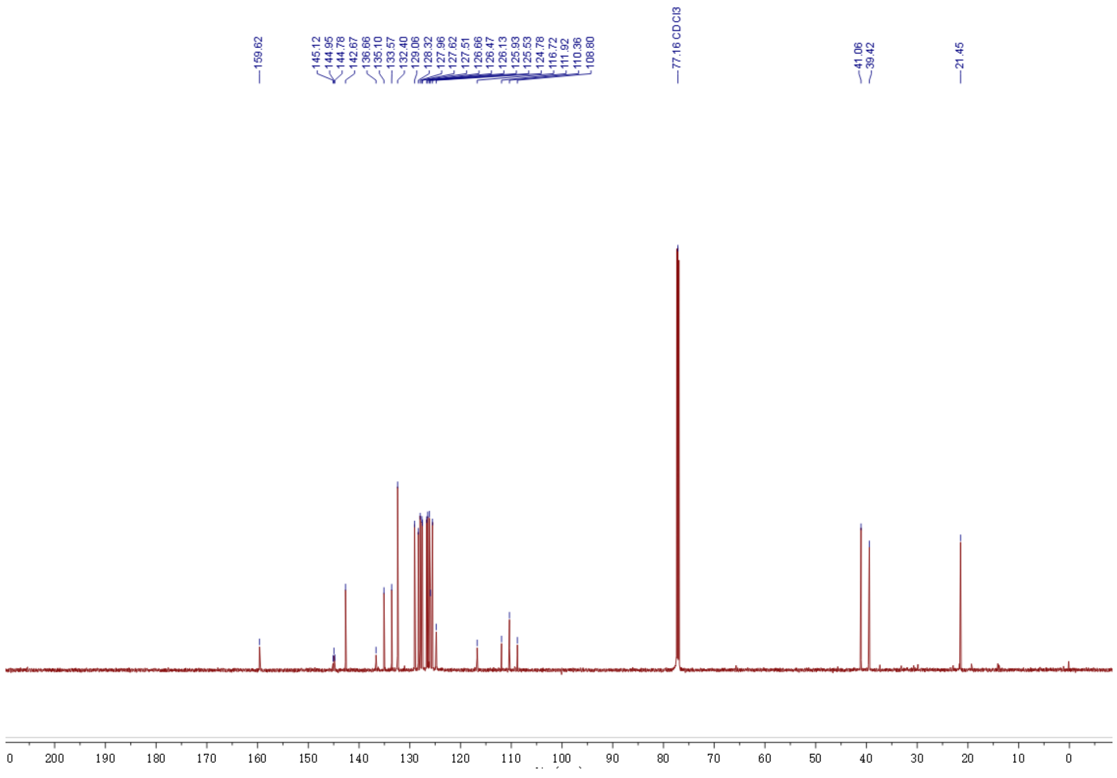
159.62
145.12 144.95 144.78 142.67 136.66 135.10 133.57 132.40 129.06 128.32 127.96 127.62 127.51 126.86 126.47 126.13 125.93 125.53 124.78 116.72 111.92 110.36 108.80
77.16 CDCl3
41.06 39.42
21.45
200 190 180 170 160 150 140 130 120 110 100 90 80 70 60 50 40 30 20 10 0

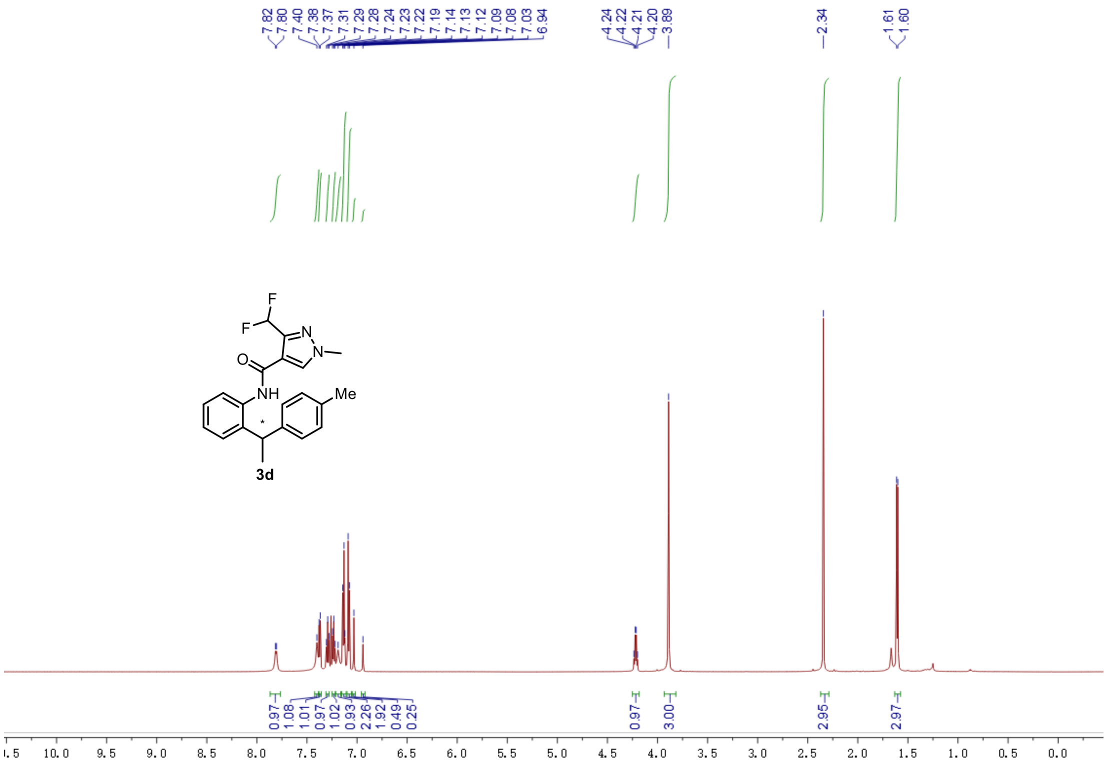
7.82
7.80
7.40
7.38
7.37
7.31
7.29
7.28
7.24
7.23
7.22
7.19
7.14
7.13
7.12
7.09
7.08
7.03
6.94
4.24
4.22
4.21
4.20
3.89
2.34
1.61
1.60
F
F
N
N
O
NH
Me
*
3d
0.97
1.08
1.01
0.97
1.02
0.93
2.26
1.92
0.49
0.25
0.97
3.00
2.95
2.97

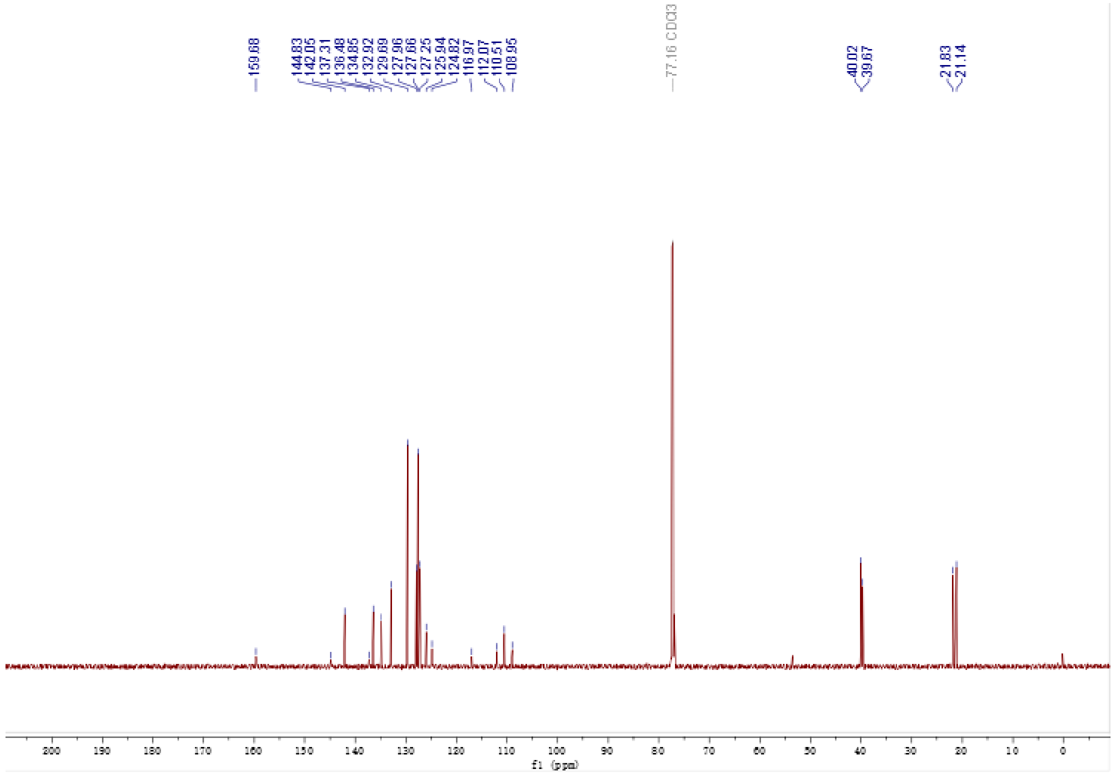
159.68
144.83
142.05
137.31
136.48
134.85
132.92
129.69
127.96
127.66
127.25
125.94
124.82
116.97
112.07
110.51
108.95
77.16 CDCl3
40.02
39.67
21.83
21.14
f1 (ppm)

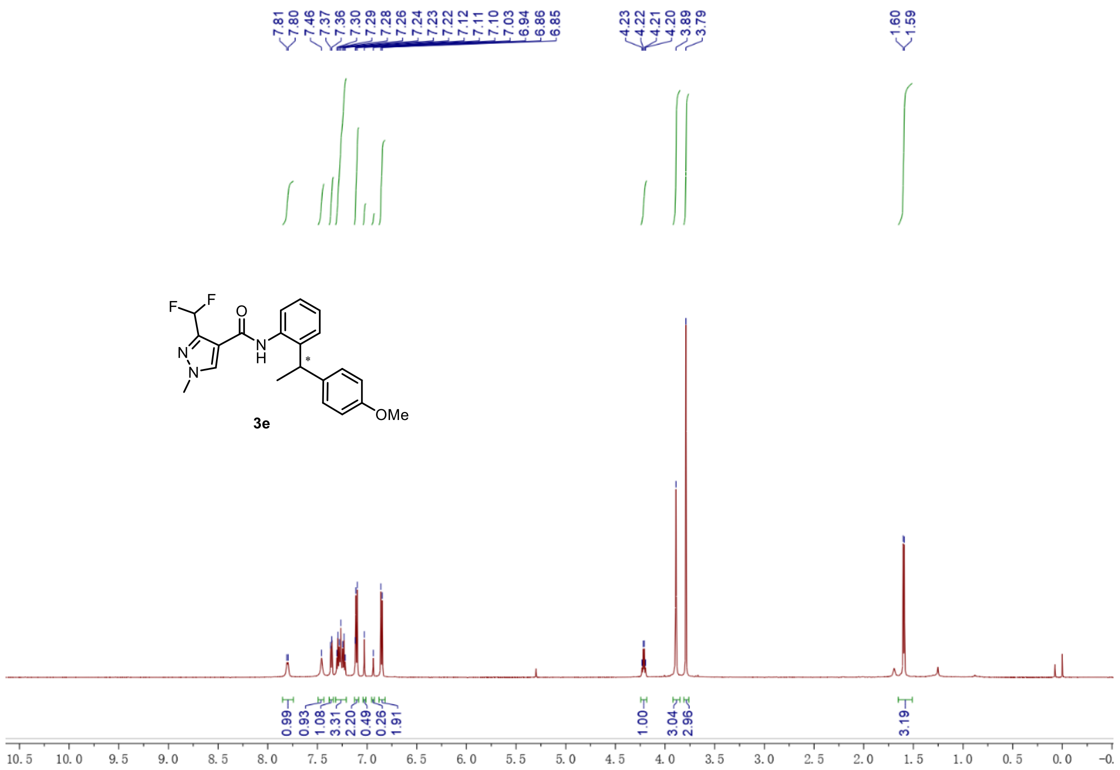
7.81 7.80 7.46 7.37 7.36 7.30 7.29 7.28 7.26 7.24 7.23 7.22 7.12 7.11 7.10 7.03 6.94 6.86 6.85
4.23 4.22 4.21 4.20 3.89 3.79
1.60 1.59
F F O N H N N * OMe
3e
0.99 0.93 1.08 3.31 2.20 0.49 0.26 1.91
1.00 3.04 2.96
3.19
10.5 10.0 9.5 9.0 8.5 8.0 7.5 7.0 6.5 6.0 5.5 5.0 4.5 4.0 3.5 3.0 2.5 2.0 1.5 1.0 0.5 0.0

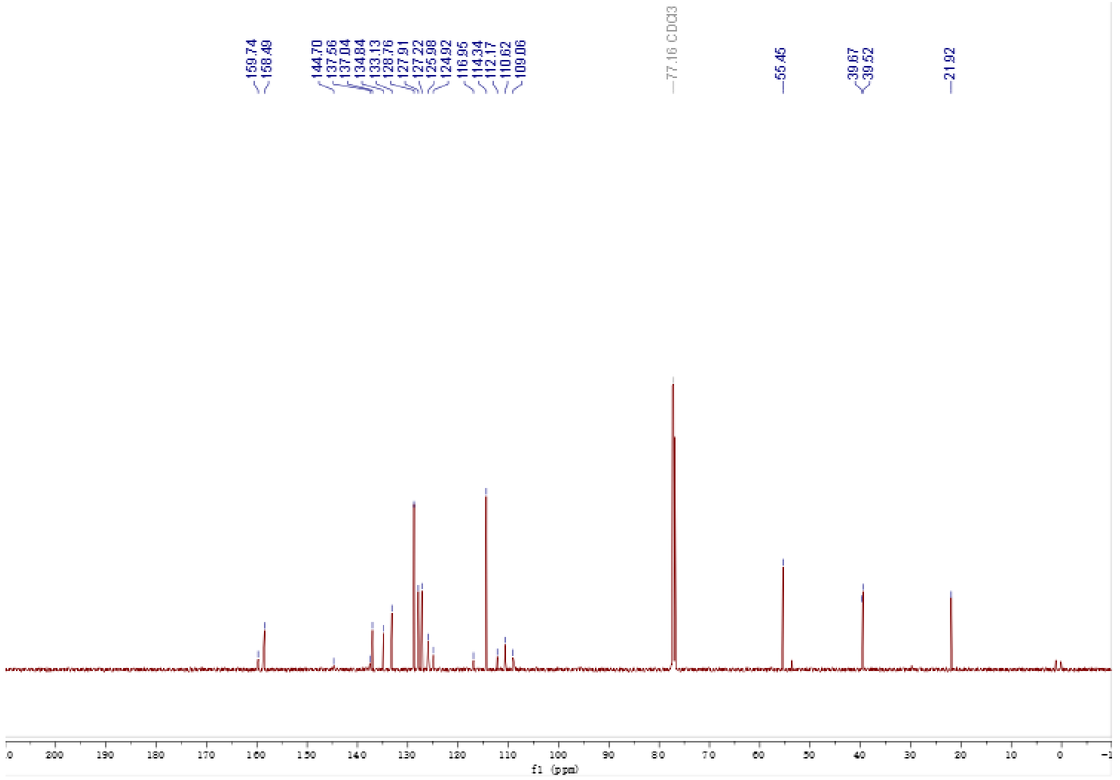
159.74 158.49
144.70 137.56 137.04 134.84 133.13 128.76 127.91 127.22 125.98 124.92 116.95 114.34 112.17 110.62 109.06
77.16 CDCl3
55.45
39.67 39.52
21.92
200 190 180 170 160 150 140 130 120 110 100 90 80 70 60 50 40 30 20 10 0
f1 (ppm)

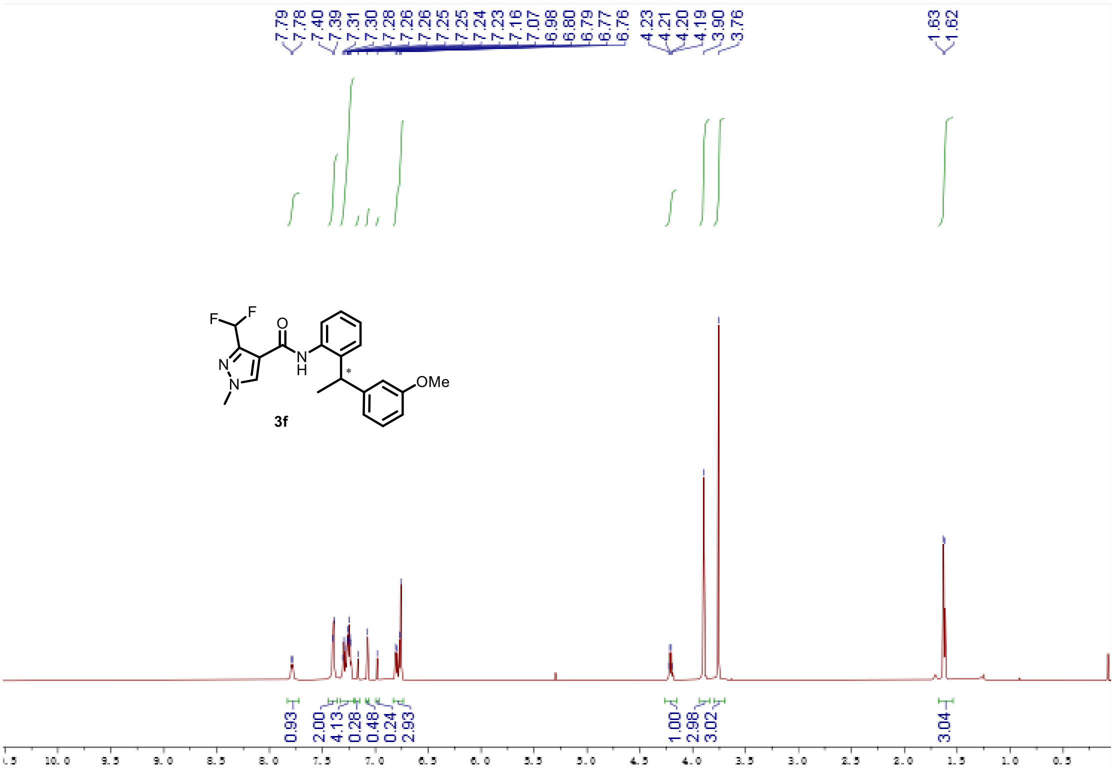
7.79
7.78
7.40
7.39
7.31
7.30
7.28
7.26
7.26
7.25
7.25
7.24
7.23
7.16
7.07
6.98
6.80
6.79
6.77
6.76
4.23
4.21
4.20
4.19
3.90
3.76
1.63
1.62
F
F
O
N
H
N
N
OMe
3f
0.93
2.00
4.13
0.28
0.48
0.24
2.93
1.00
2.98
3.02
3.04

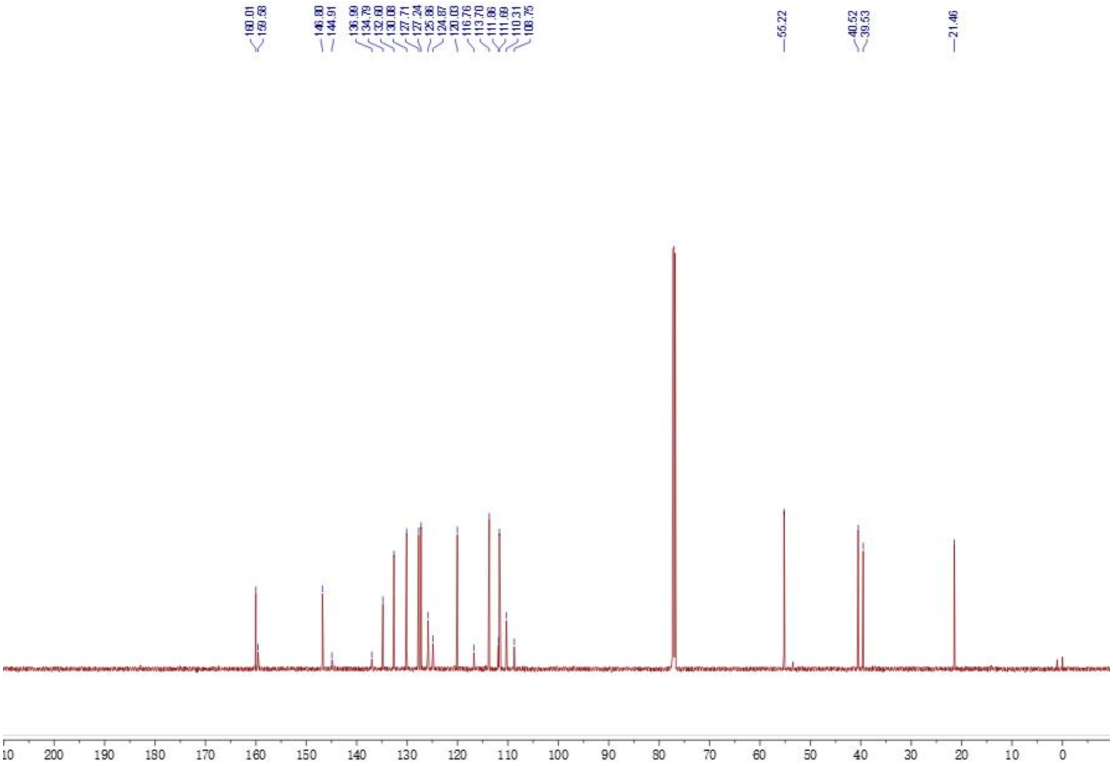
160.01
159.58
146.80
144.91
136.99
134.79
132.60
130.08
127.71
127.24
125.88
124.87
120.03
116.76
113.70
111.86
111.69
110.31
108.75
55.22
40.52
39.53
21.46

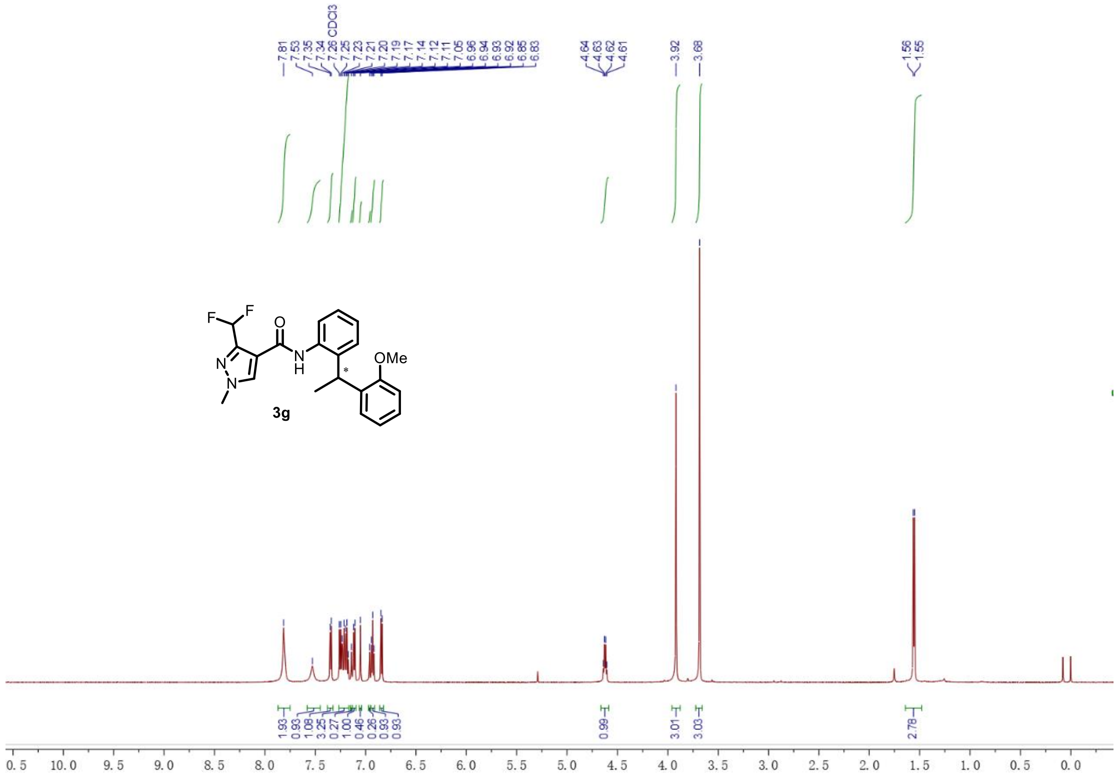
7.81
7.53
7.35
7.34
7.26 CDCl3
7.25
7.23
7.21
7.20
7.19
7.17
7.14
7.12
7.11
7.05
6.96
6.94
6.93
6.92
6.85
6.83
4.64
4.63
4.62
4.61
3.92
3.68
1.56
1.55
F
F
O
N
H
N
N
OMe
*
3g
1.93
0.93
1.08
3.25
0.27
1.00
0.46
0.26
0.93
0.93
0.99
3.01
3.03
2.78
0.5
10.0
9.5
9.0
8.5
8.0
7.5
7.0
6.5
6.0
5.5
5.0
4.5
4.0
3.5
3.0
2.5
2.0
1.5
1.0
0.5
0.0

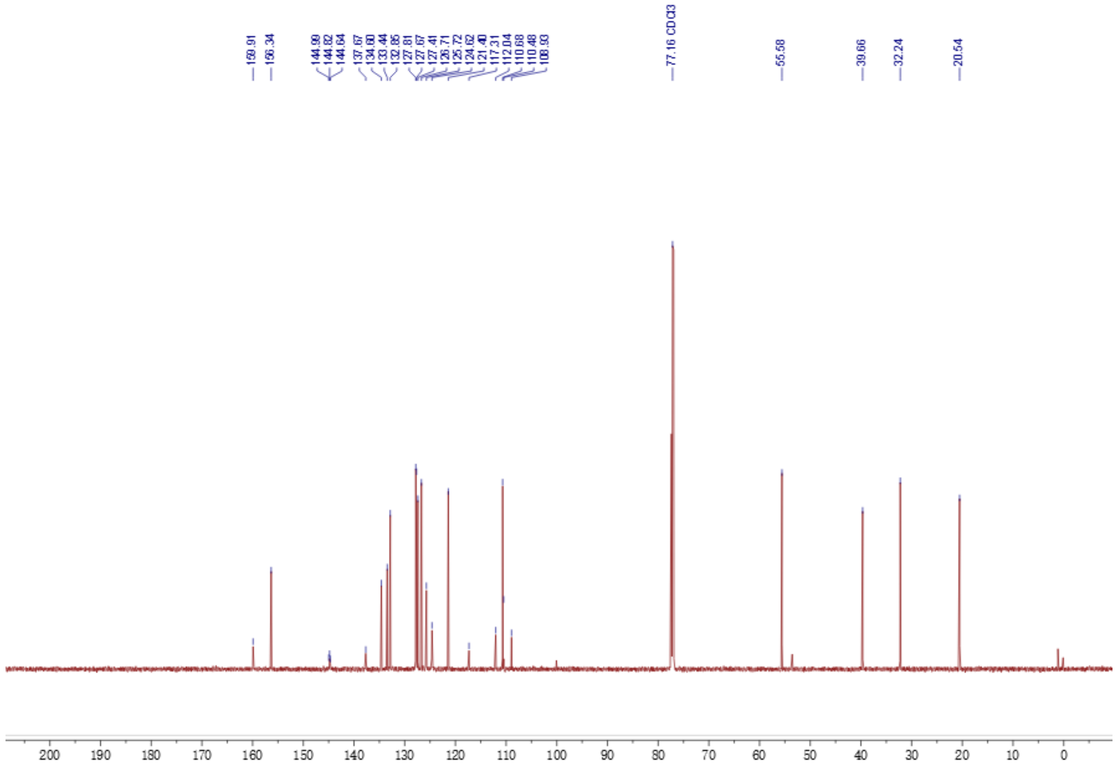
159.91
156.34
144.99
144.82
144.64
137.67
134.60
133.44
132.85
127.81
127.67
127.41
126.71
125.72
124.62
121.40
117.31
112.04
110.68
110.48
108.93
77.16 CDCl3
55.58
39.66
32.24
20.54
200
190
180
170
160
150
140
130
120
110
100
90
80
70
60
50
40
30
20
10
0

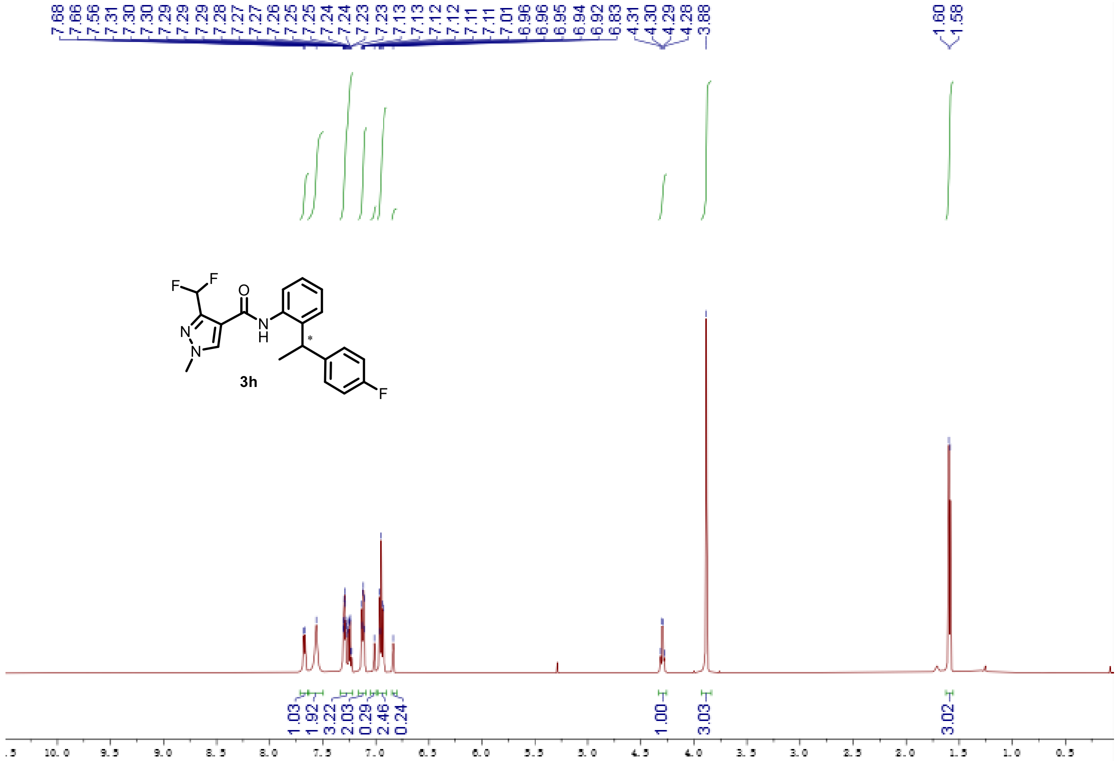
7.68 7.66 7.56 7.31 7.30 7.30 7.29 7.29 7.29 7.28 7.27 7.27 7.26 7.25 7.25 7.24 7.24 7.23 7.23 7.13 7.13 7.12 7.12 7.11 7.11 7.01 6.96 6.96 6.95 6.94 6.92 6.83 4.31 4.30 4.29 4.28 3.88
1.60 1.58
F F O N H N N * F
3h
1.03 1.92 3.22 2.03 0.29 2.46 0.24
1.00
3.03
3.02

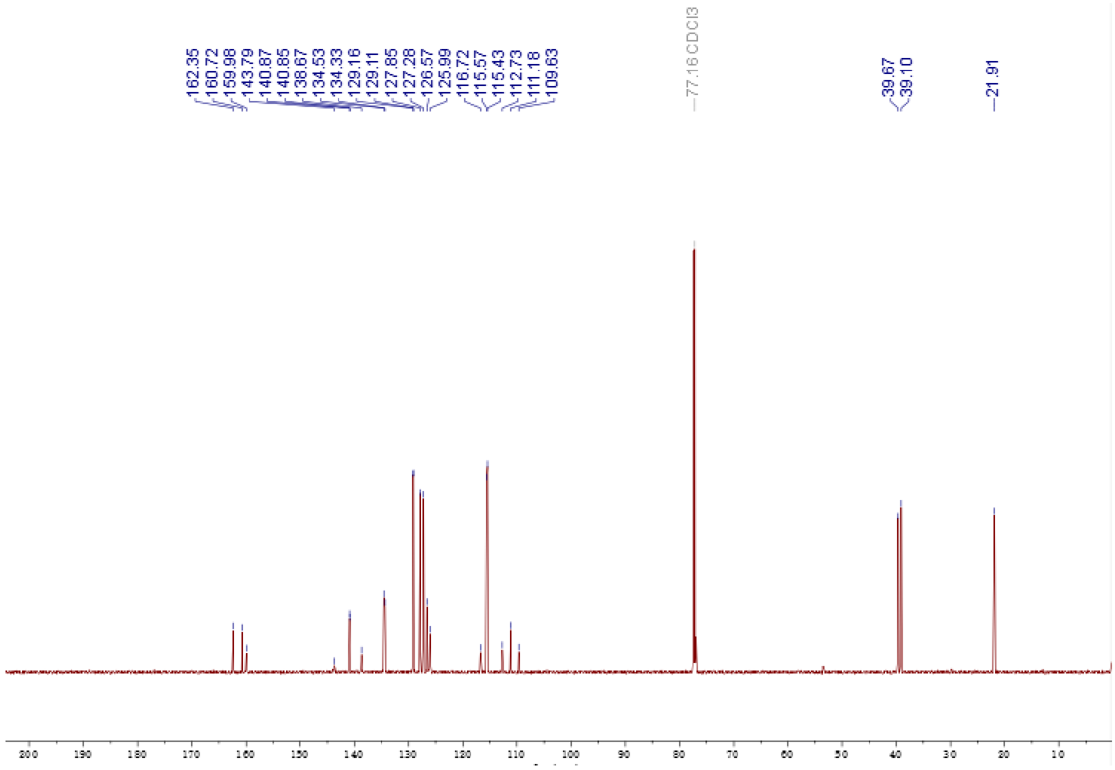
162.35 160.72 159.98 143.79 140.87 140.85 138.67 134.53 134.33 129.16 129.11 127.85 127.28 126.57 125.99 116.72 115.57 115.43 112.73 111.18 109.63
77.16 CDCl3
39.67 39.10
21.91

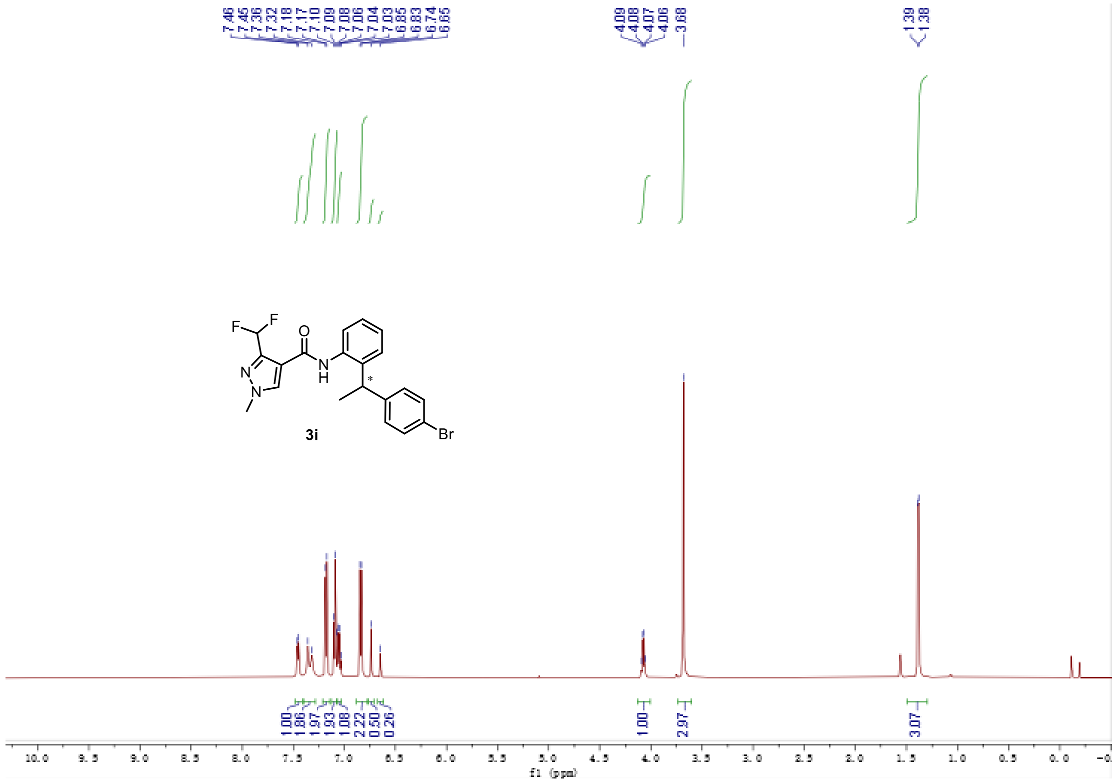
7.46
7.45
7.36
7.32
7.18
7.17
7.10
7.09
7.08
7.06
7.04
7.03
6.85
6.83
6.74
6.65
4.09
4.08
4.07
4.06
3.68
1.39
1.38
F
F
O
N
H
N
N
*
Br
3i
1.00
1.86
1.97
1.93
1.08
2.22
0.50
0.26
1.00
2.97
3.07
f1 (ppm)

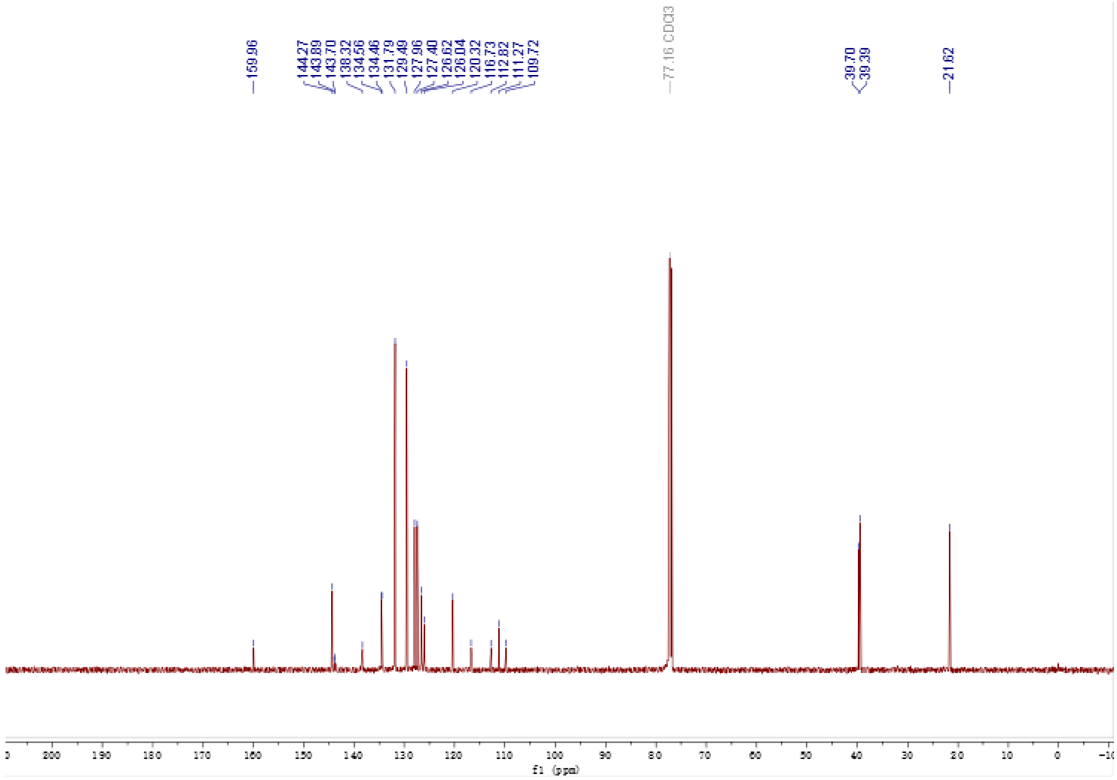
159.96
144.27
143.89
143.70
138.32
134.56
134.46
131.79
129.49
127.96
127.40
126.62
126.04
120.32
116.73
112.82
111.27
109.72
77.16 CDCl3
39.70
39.39
21.62
f1 (ppm)

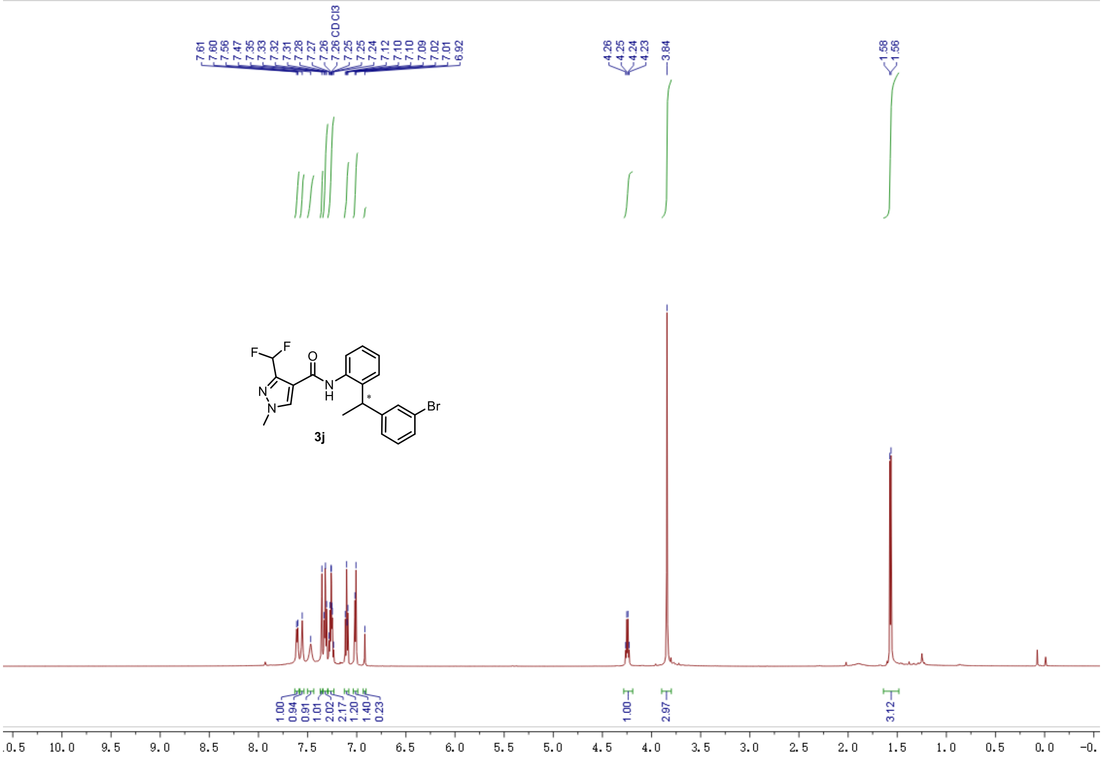
7.61
7.60
7.56
7.47
7.35
7.33
7.32
7.31
7.28
7.27
7.26 CDCl3
7.26
7.25
7.25
7.24
7.12
7.10
7.10
7.09
7.02
7.01
6.92
4.26
4.25
4.24
4.23
3.84
1.58
1.56
3j
Br
F
F
O
N
N
N
H
1.00
0.94
0.91
1.01
2.02
2.17
1.20
1.40
0.23
1.00
2.97
3.12

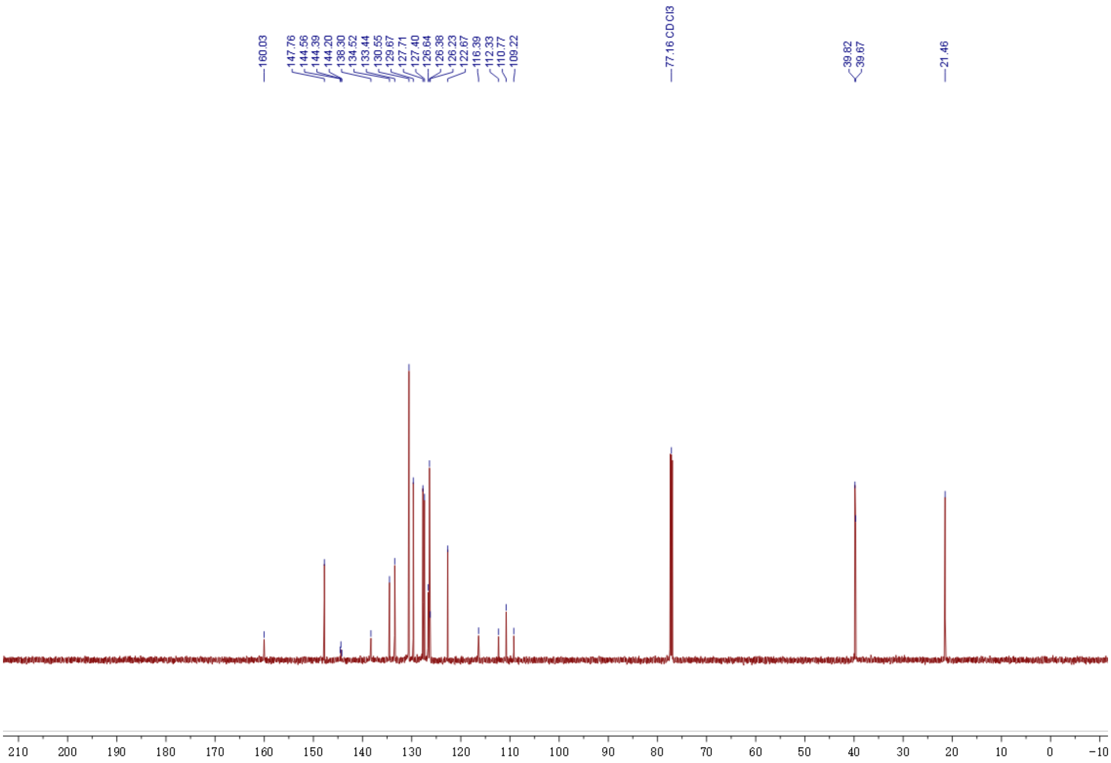
160.03
147.76
144.56
144.39
144.20
138.30
134.52
133.44
130.55
129.67
127.71
127.40
126.64
126.38
126.23
122.67
116.39
112.33
110.77
109.22
77.16 CDCl3
39.82
39.67
21.46

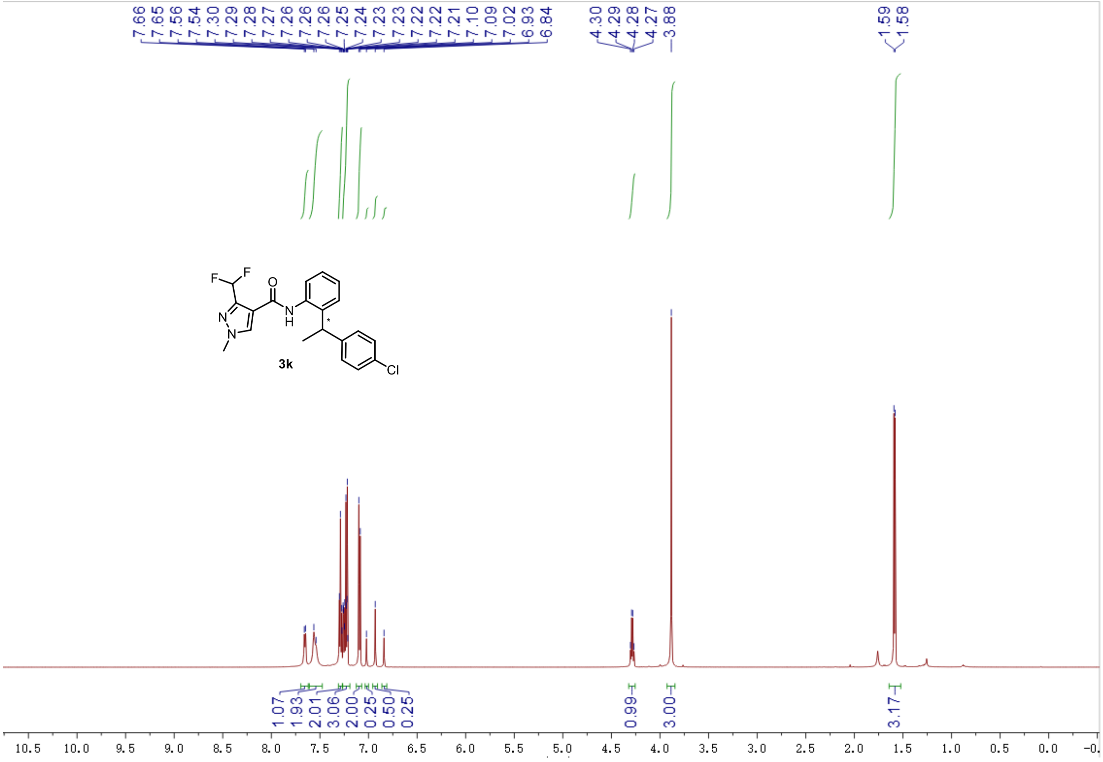
7.66
7.65
7.56
7.54
7.30
7.29
7.28
7.27
7.26
7.26
7.26
7.25
7.24
7.23
7.23
7.22
7.22
7.21
7.10
7.09
7.02
6.93
6.84
4.30
4.29
4.28
4.27
3.88
1.59
1.58
F
F
O
N
H
N
N
3k
Cl
1.07
1.93
2.01
3.06
2.00
0.25
0.50
0.25
0.99
3.00
3.17
10.5 10.0 9.5 9.0 8.5 8.0 7.5 7.0 6.5 6.0 5.5 5.0 4.5 4.0 3.5 3.0 2.5 2.0 1.5 1.0 0.5 0.0

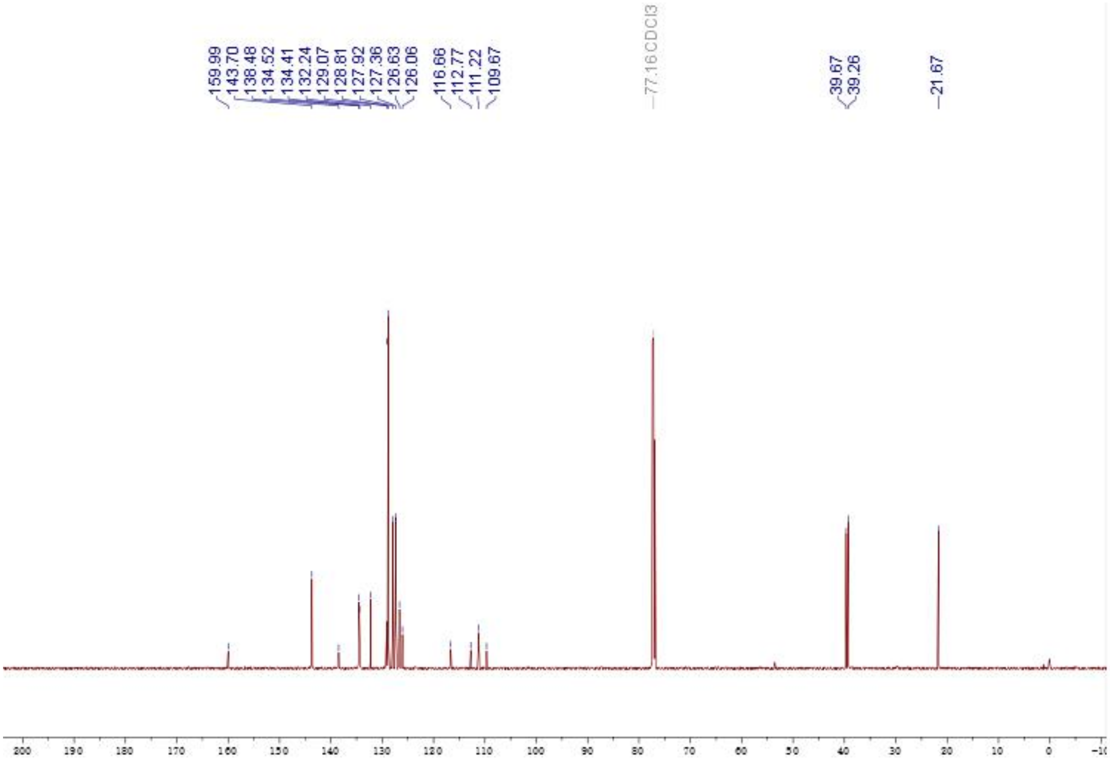
159.99
143.70
138.48
134.52
134.41
132.24
129.07
128.81
127.92
127.36
126.63
126.06
116.66
112.77
111.22
109.67
77.16 CDCl3
39.67
39.26
21.67
200 190 180 170 160 150 140 130 120 110 100 90 80 70 60 50 40 30 20 10 0

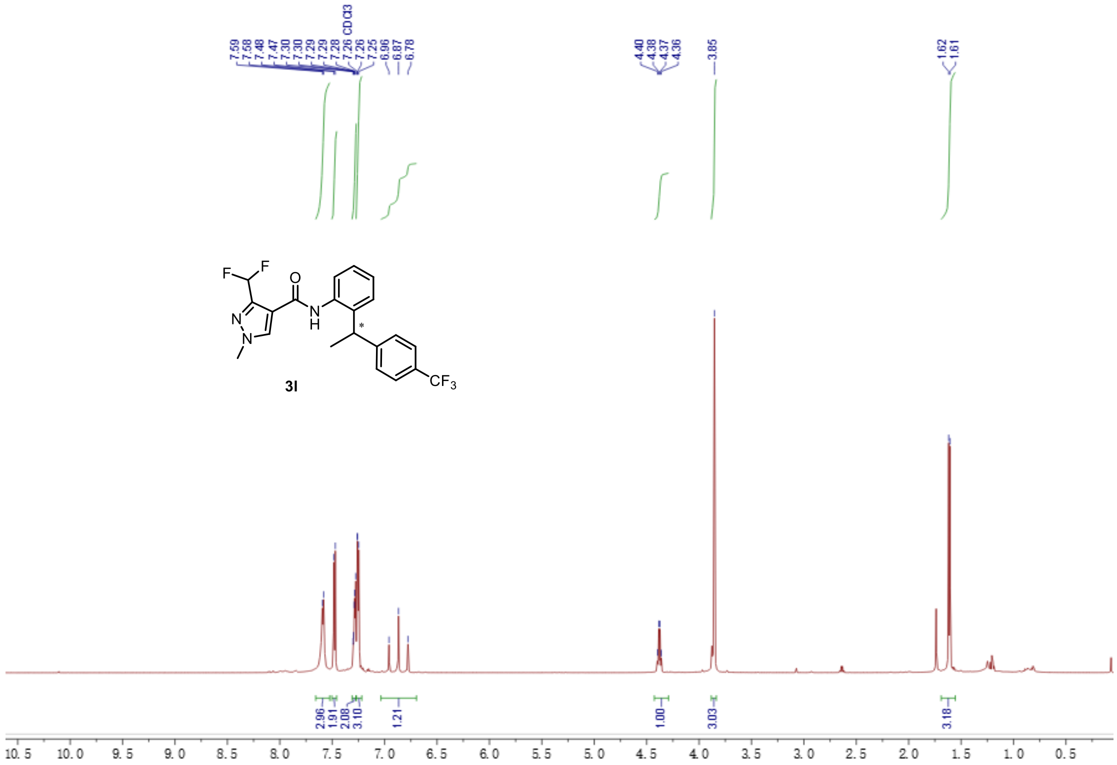
7.59 7.58 7.48 7.47 7.30 7.30 7.29 7.29 7.28 7.26 CDCl3 7.26 7.25 6.96 6.87 6.78
4.40 4.38 4.37 4.36 3.85
1.62 1.61
F F O N H N N *
3l
CF3
2.96 1.91 2.08 3.10 1.21 1.00 3.03 3.18

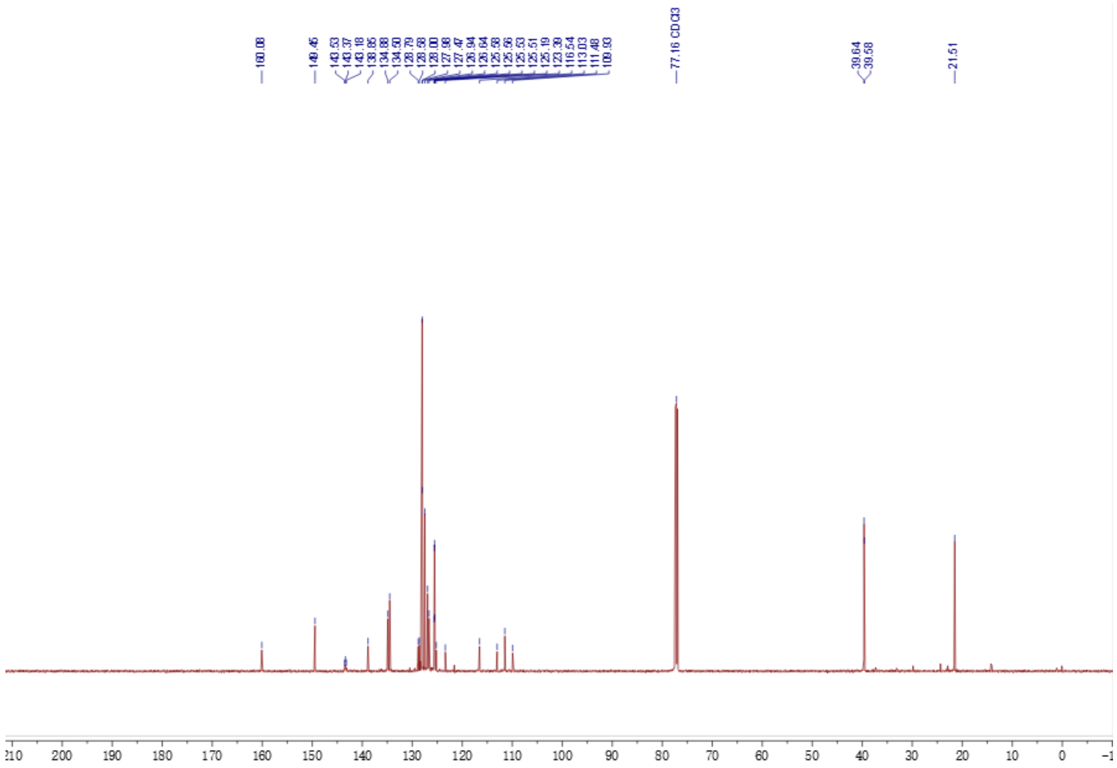
160.08 149.46 143.53 143.37 143.18 138.86 134.88 134.50 128.79 128.58 128.00 127.98 127.47 126.94 126.64 125.58 125.56 125.53 125.51 125.19 123.39 116.54 113.03 111.48 109.93
77.16 CDCl3
39.64 39.58
21.51

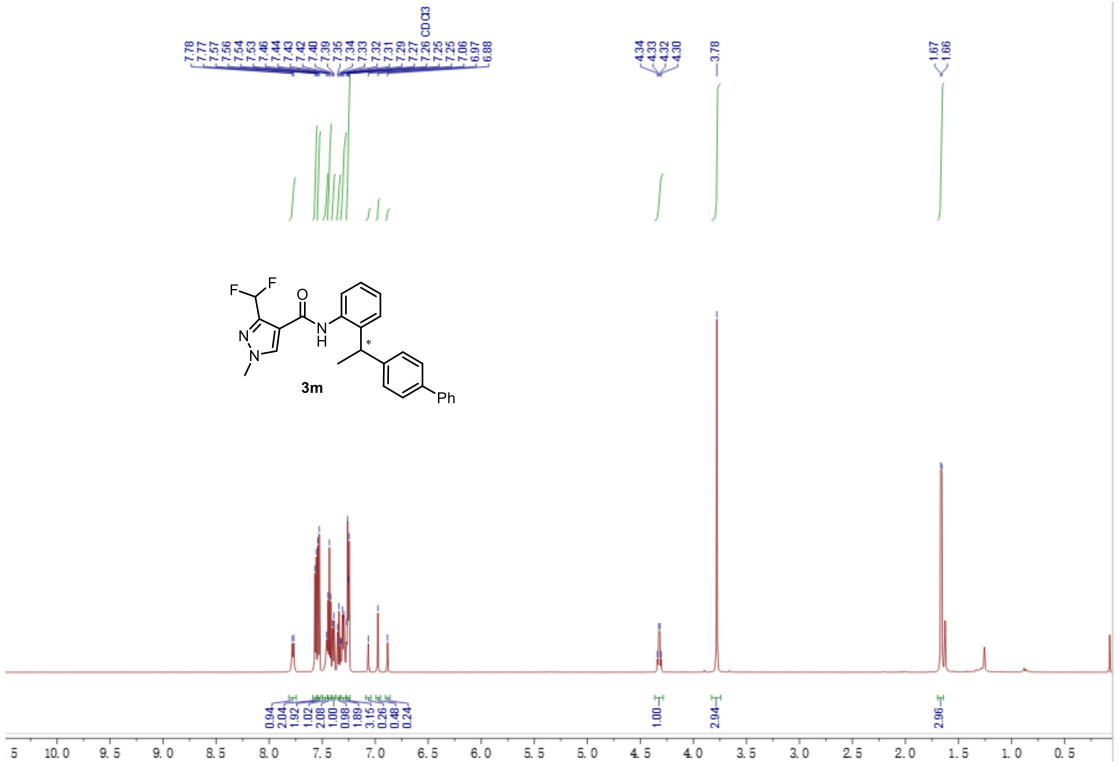
7.78
7.77
7.57
7.56
7.54
7.53
7.46
7.44
7.43
7.42
7.40
7.39
7.35
7.34
7.33
7.32
7.31
7.29
7.27
7.26 CDCl3
7.25
7.25
7.06
6.97
6.88
4.34
4.33
4.32
4.30
3.78
1.67
1.66
3m
Ph
0.94
2.04
1.92
1.02
2.08
1.00
0.98
1.89
3.15
0.26
0.48
0.24
1.00
2.94
2.96

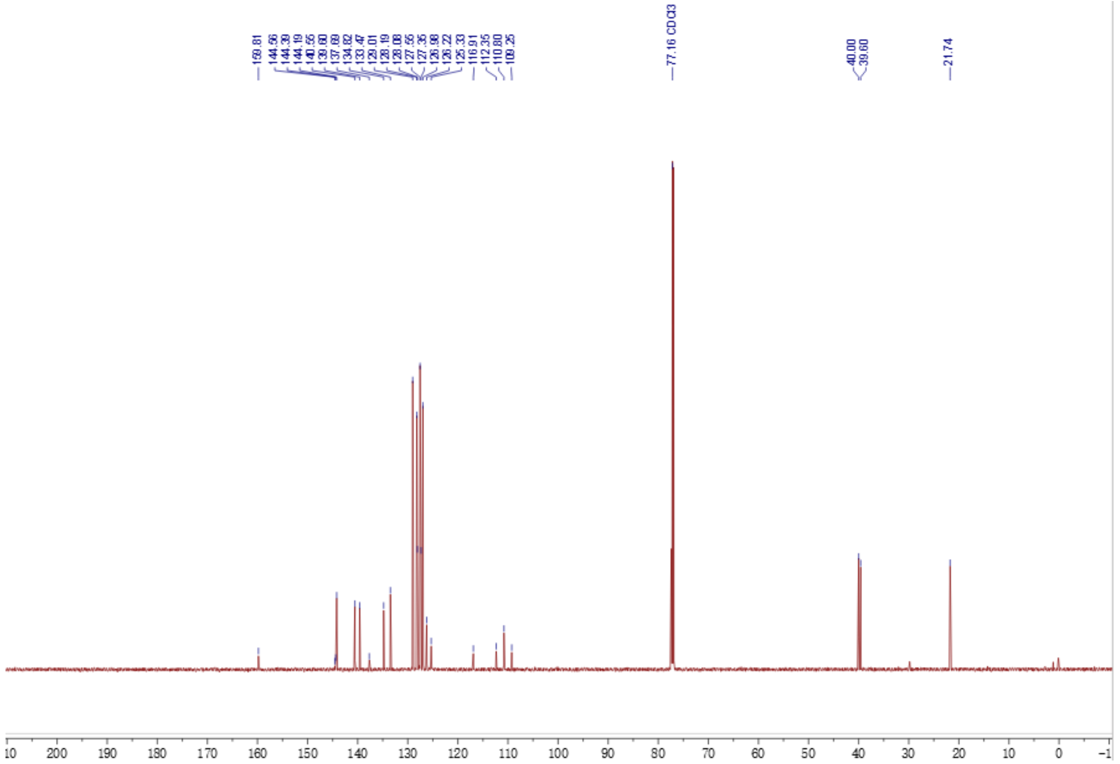
159.81
144.56
144.39
144.19
140.55
139.60
137.69
134.82
133.47
129.01
128.19
128.08
127.55
127.36
126.98
126.22
125.33
116.91
112.35
110.80
109.25
77.16 CDCl3
40.00
39.60
21.74

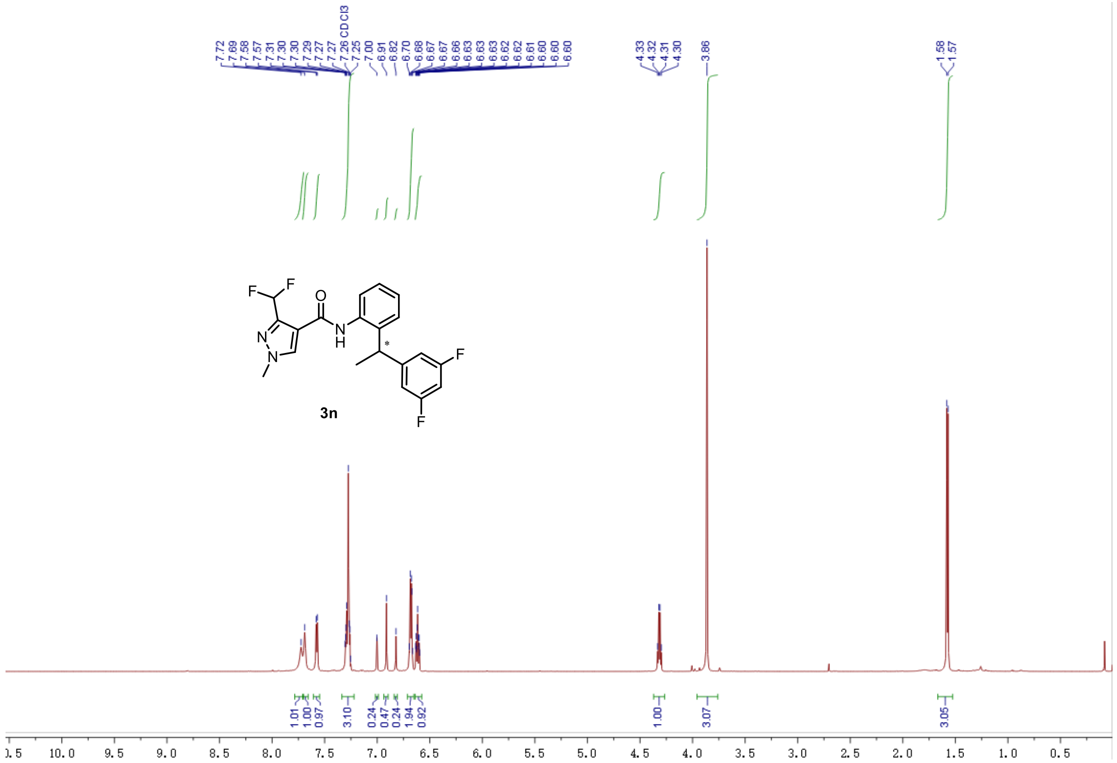
7.72
7.69
7.58
7.57
7.31
7.30
7.30
7.29
7.27
7.27
7.26 CDCl3
7.25
7.00
6.91
6.82
6.70
6.68
6.67
6.67
6.66
6.63
6.63
6.63
6.62
6.62
6.61
6.60
6.60
6.60
4.33
4.32
4.31
4.30
3.86
1.58
1.57
3n
1.01
1.00
0.97
3.10
0.24
0.47
0.24
1.94
0.92
1.00
3.07
3.05

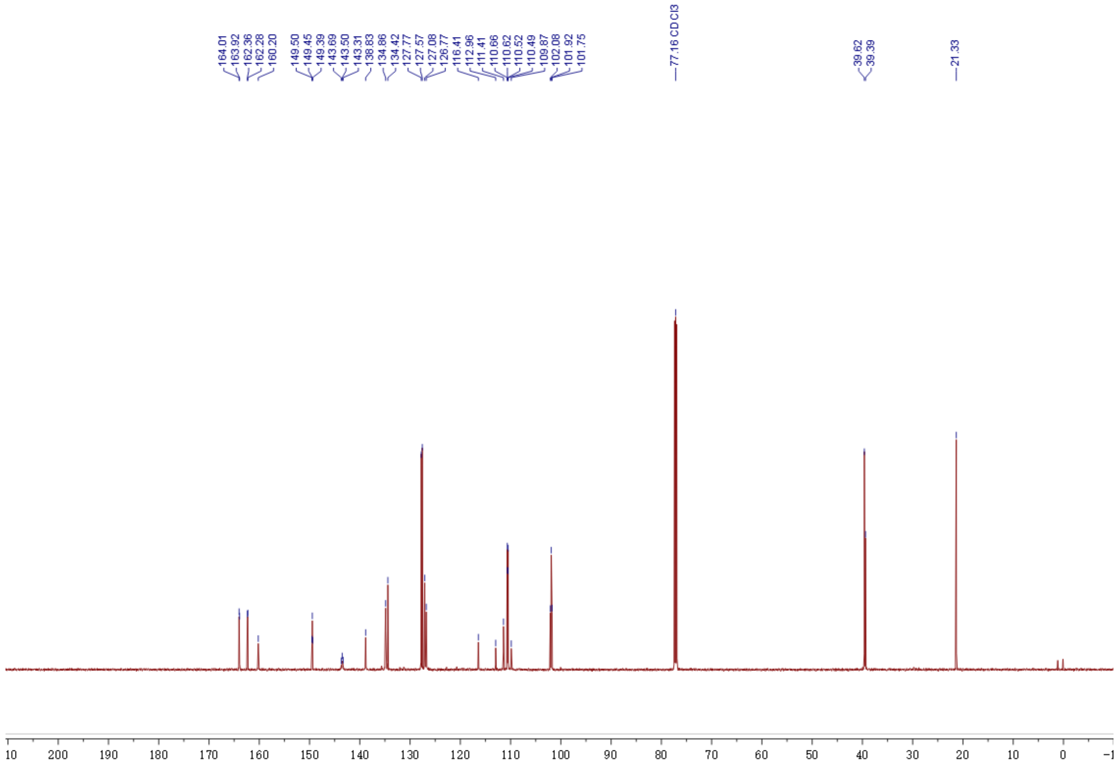
164.01
163.92
162.36
162.28
160.20
149.50
149.45
149.39
143.69
143.50
143.31
138.83
134.86
134.42
127.77
127.57
127.08
126.77
116.41
112.96
111.41
110.66
110.62
110.52
110.49
109.87
102.08
101.92
101.75
77.16 CDCl3
39.62
39.39
21.33

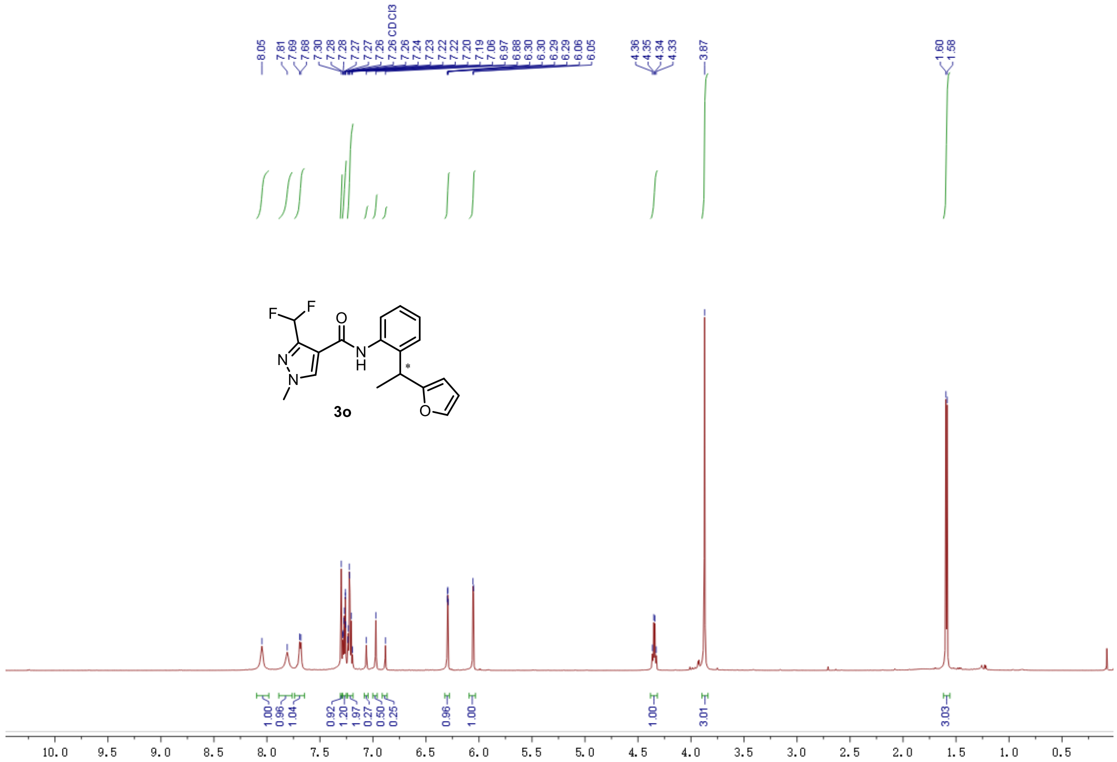
8.05
7.81
7.69
7.68
7.30
7.28
7.28
7.27
7.27
7.26 CDCl3
7.26
7.24
7.23
7.22
7.22
7.20
7.19
7.06
6.97
6.88
6.30
6.30
6.29
6.29
6.06
6.05
4.36
4.35
4.34
4.33
3.87
1.60
1.58
F
F
O
N
N
N
H
*
O
3o
1.00
0.96
1.04
0.92
1.20
1.97
0.27
0.50
0.25
0.96
1.00
1.00
3.01
3.03
10.0
9.5
9.0
8.5
8.0
7.5
7.0
6.5
6.0
5.5
5.0
4.5
4.0
3.5
3.0
2.5
2.0
1.5
1.0
0.5

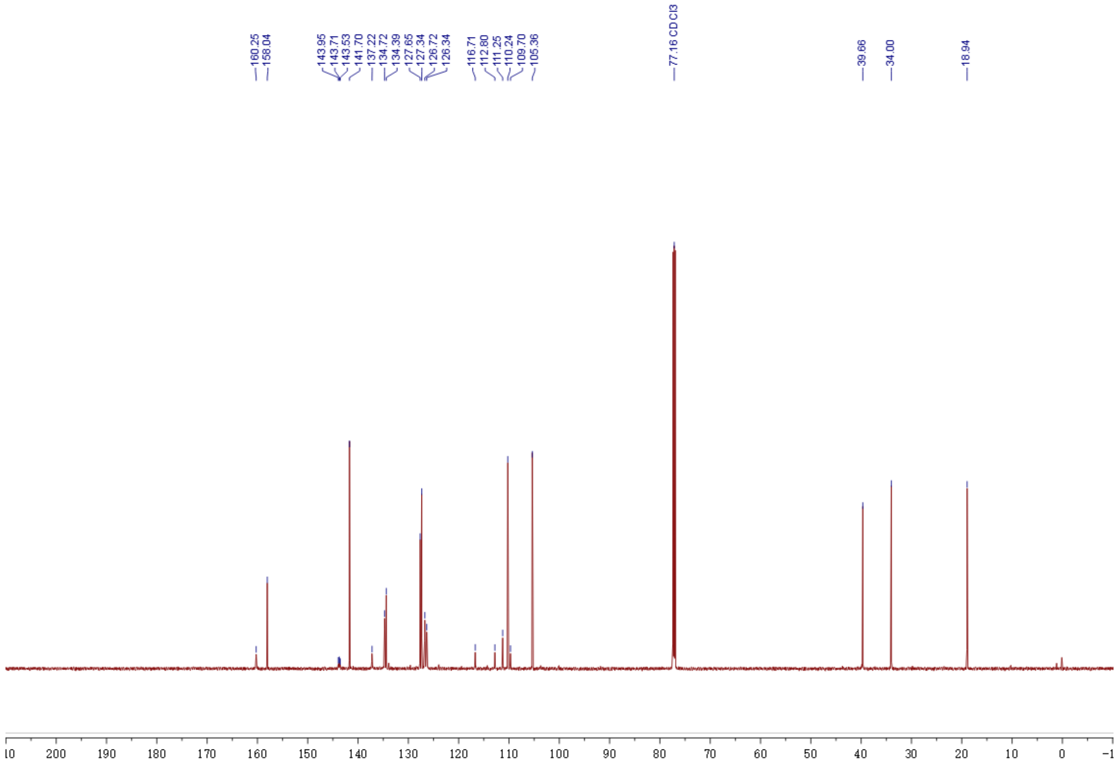
160.25
158.04
143.95
143.71
143.53
141.70
137.22
134.72
134.39
127.65
127.34
126.72
126.34
116.71
112.80
111.25
110.24
109.70
105.36
77.16 CDCl3
39.66
34.00
18.94
200
190
180
170
160
150
140
130
120
110
100
90
80
70
60
50
40
30
20
10
0

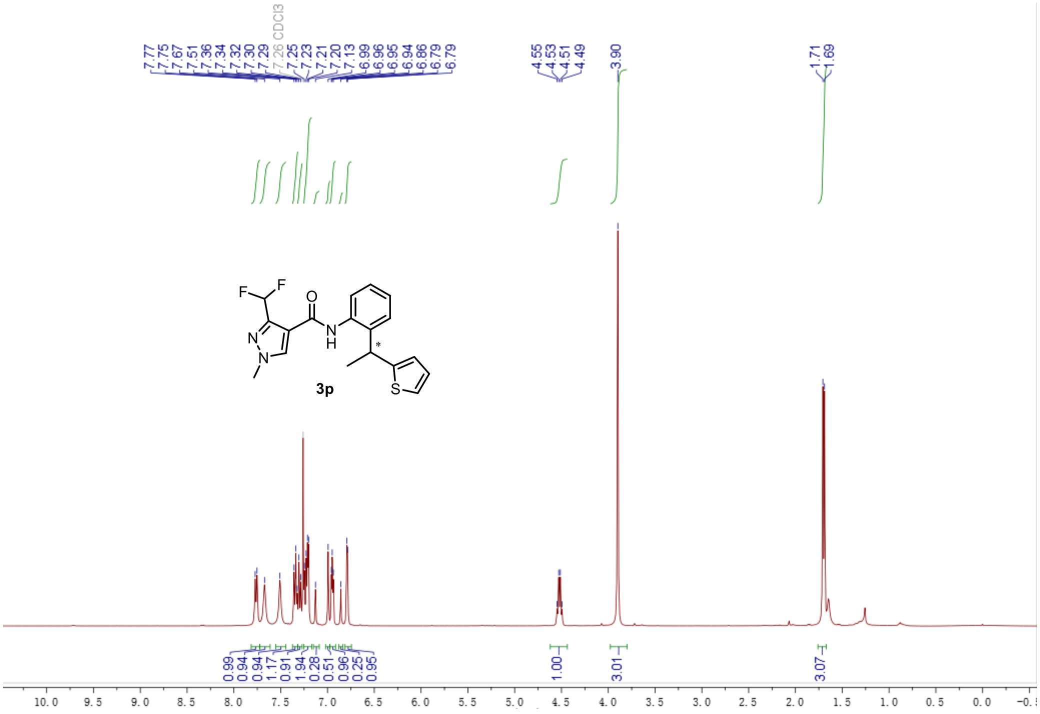
7.77 7.75 7.67 7.51 7.36 7.34 7.32 7.30 7.29 7.26 CDCl3 7.25 7.23 7.21 7.20 7.13 6.99 6.96 6.95 6.94 6.86 6.79 6.79
4.55 4.53 4.51 4.49
3.90
1.71 1.69
3p
0.99 0.94 0.94 1.17 0.91 1.94 0.28 0.51 0.96 0.25 0.95
1.00
3.01
3.07

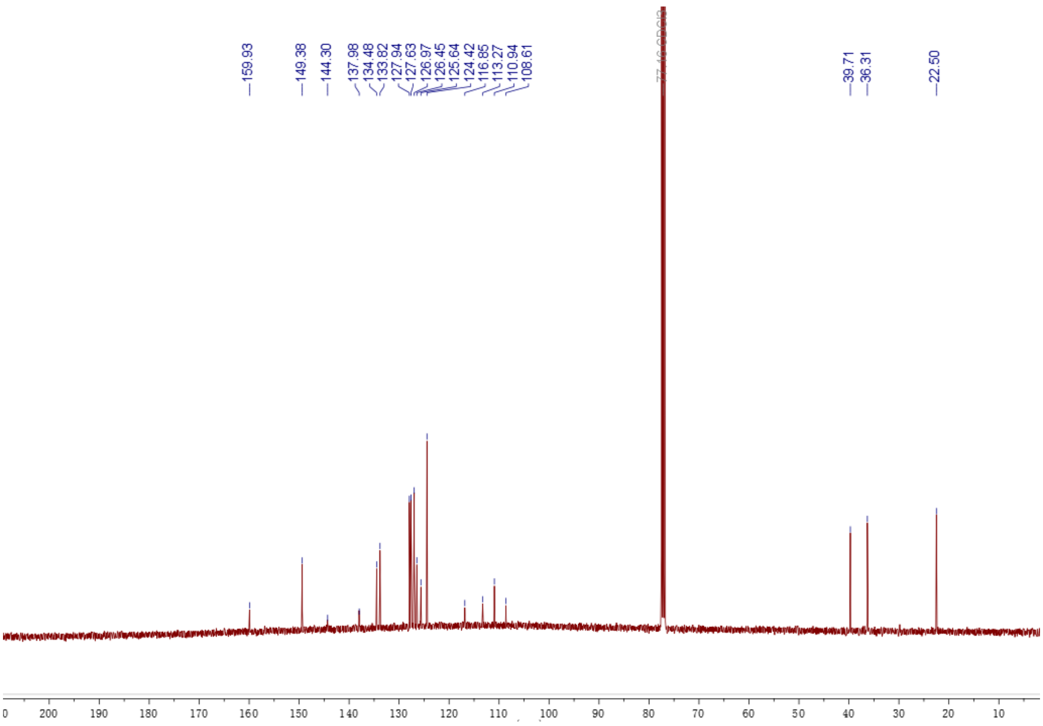
159.93
149.38
144.30
137.98 134.48 133.82 127.94 127.63 126.97 126.45 125.64 124.42 116.85 113.27 110.94 108.61
39.71 36.31
22.50

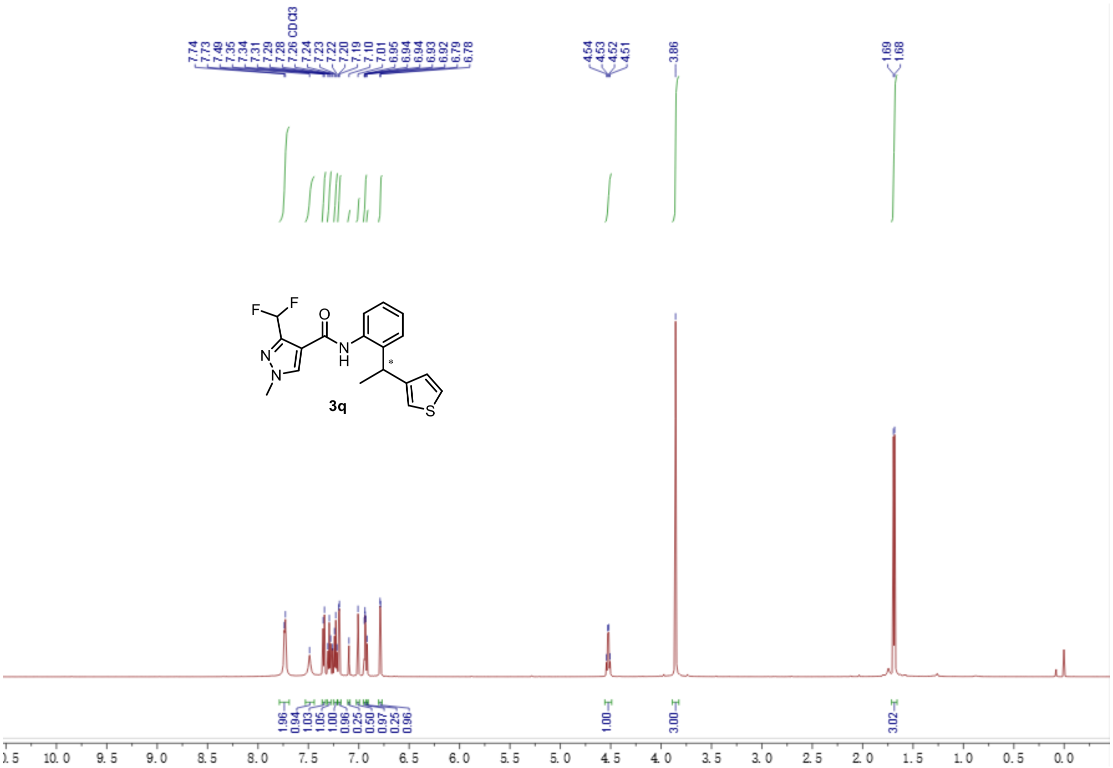
7.74 7.73 7.49 7.35 7.34 7.31 7.29 7.28 7.26 CDCl3 7.24 7.23 7.22 7.20 7.19 7.10 7.01 6.95 6.94 6.94 6.93 6.92 6.79 6.78
4.54 4.53 4.52 4.51
3.86
1.69 1.68
F F O N H N N S *
3q
1.96 0.94 1.03 1.05 1.00 0.96 0.25 0.50 0.97 0.25 0.96
1.00
3.00
3.02
10.0 9.5 9.0 8.5 8.0 7.5 7.0 6.5 6.0 5.5 5.0 4.5 4.0 3.5 3.0 2.5 2.0 1.5 1.0 0.5 0.0

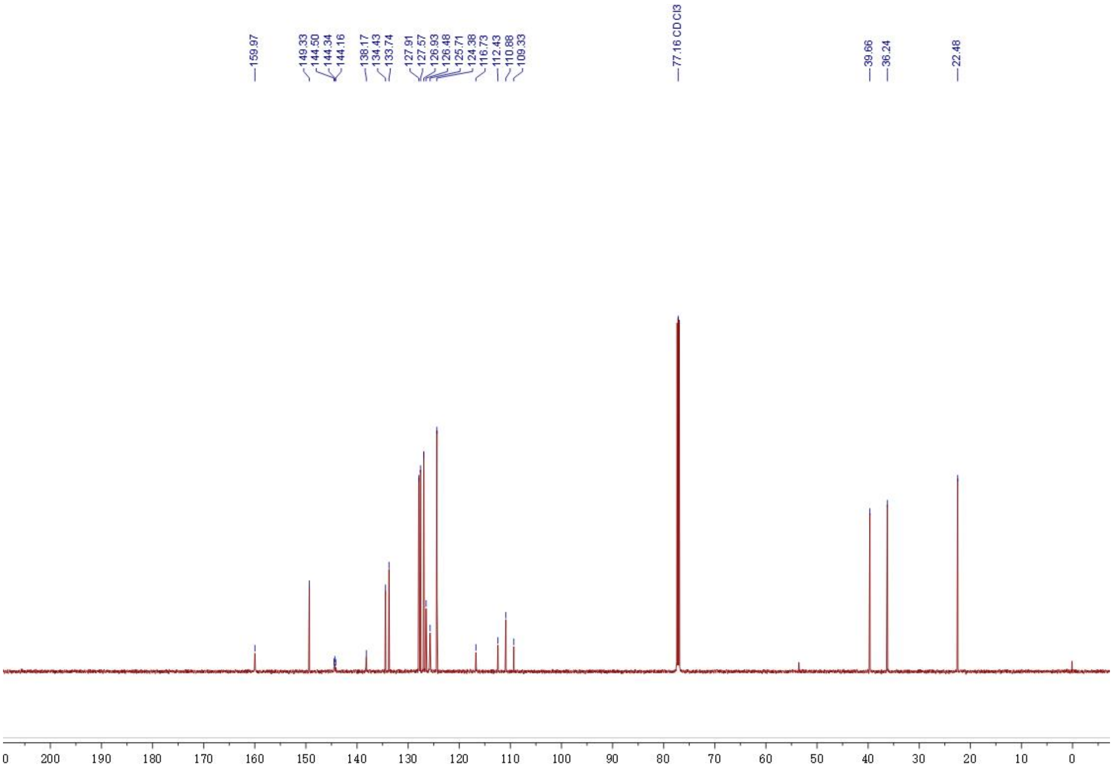
159.97
149.33 144.50 144.34 144.16
138.17 134.43 133.74
127.91 127.57 126.93 126.48 125.71 124.38
116.73 112.43 110.88 109.33
77.16 CDCl3
39.66 36.24
22.48
200 190 180 170 160 150 140 130 120 110 100 90 80 70 60 50 40 30 20 10 0

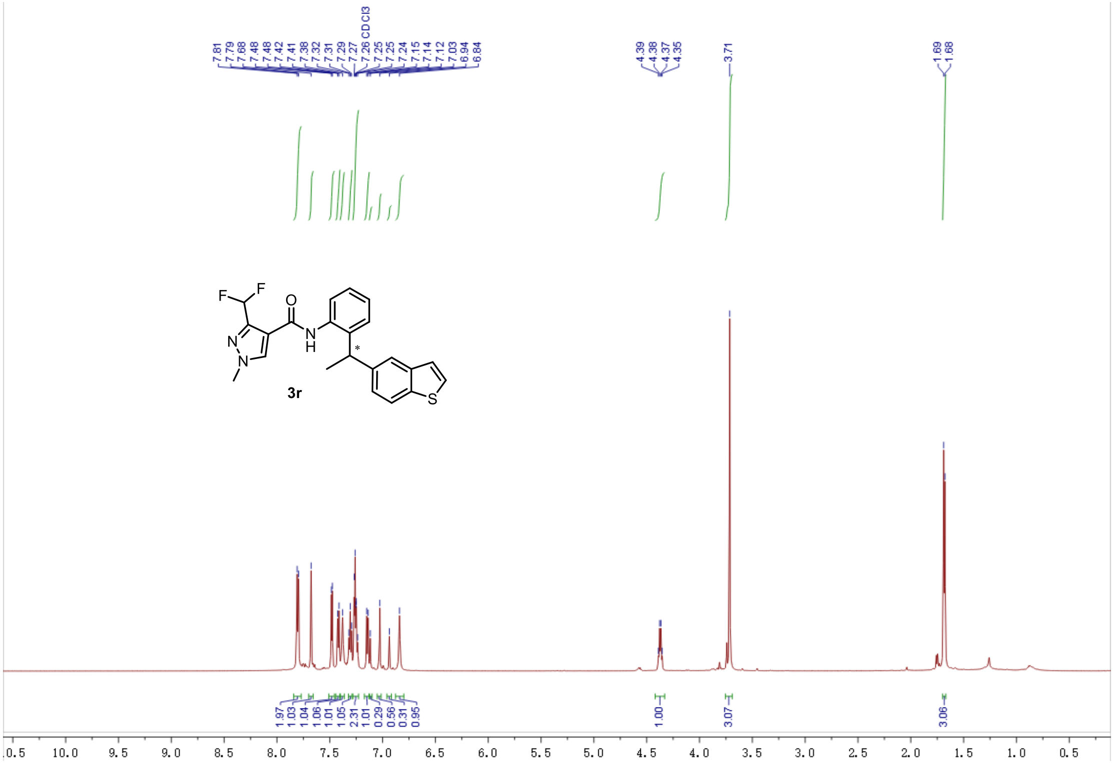
7.81
7.79
7.68
7.48
7.42
7.41
7.38
7.32
7.31
7.29
7.27
7.26 CDCl3
7.25
7.25
7.24
7.15
7.14
7.12
7.03
6.94
6.84
4.39
4.38
4.37
4.35
3.71
1.69
1.68
3r
1.97
1.03
1.04
1.06
1.01
1.05
2.31
1.01
0.29
0.56
0.31
0.95
1.00
3.07
3.06

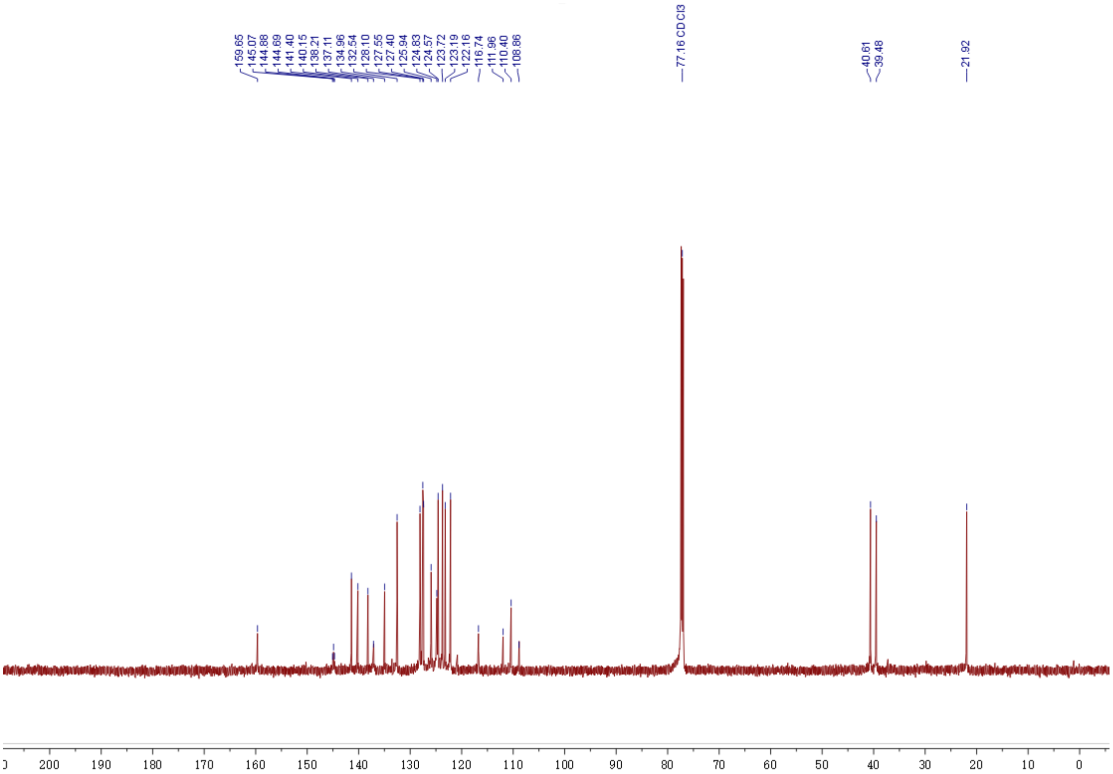
159.65
145.07
144.88
144.69
141.40
140.15
138.21
137.11
134.96
132.54
128.10
127.55
127.40
125.94
124.83
124.57
123.72
123.19
122.16
116.74
111.96
110.40
108.86
77.16 CDCl3
40.61
39.48
21.92

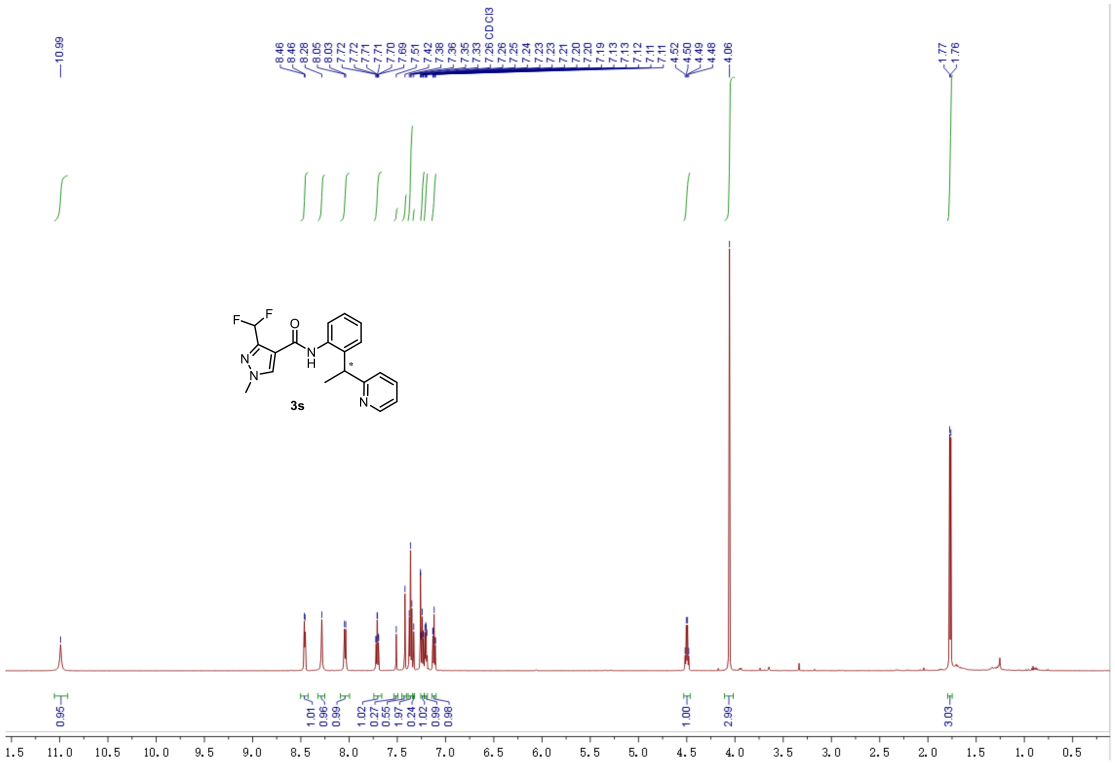
10.99
8.46
8.46
8.28
8.05
8.03
7.72
7.72
7.71
7.70
7.69
7.51
7.42
7.38
7.36
7.35
7.33
7.26 CDCl3
7.26
7.25
7.24
7.23
7.23
7.21
7.20
7.20
7.19
7.13
7.13
7.12
7.11
7.11
4.52
4.50
4.49
4.48
4.06
1.77
1.76
3s
0.95
1.01
0.96
0.99
1.02
0.27
0.55
1.97
0.24
1.02
0.99
0.98
1.00
2.99
3.03

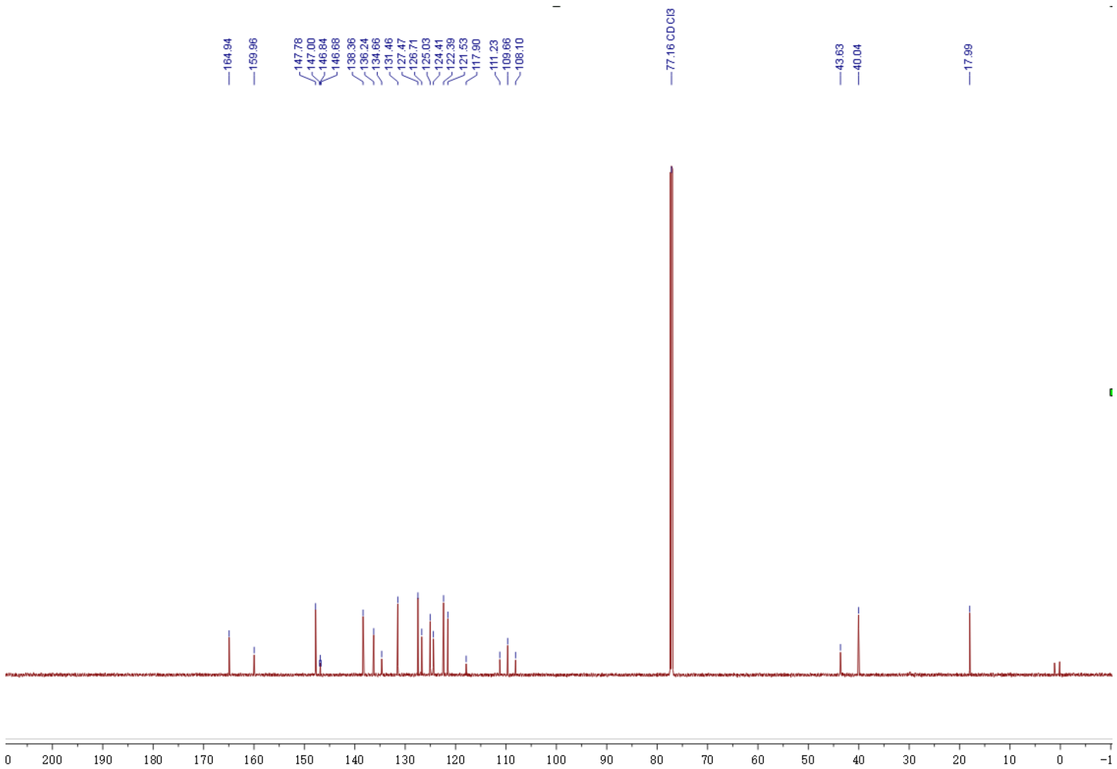
164.94
159.96
147.78
147.00
146.84
146.68
138.36
136.24
134.66
131.46
127.47
126.71
125.03
124.41
122.39
121.53
117.90
111.23
109.66
108.10
77.16 CDCl3
43.63
40.04
17.99

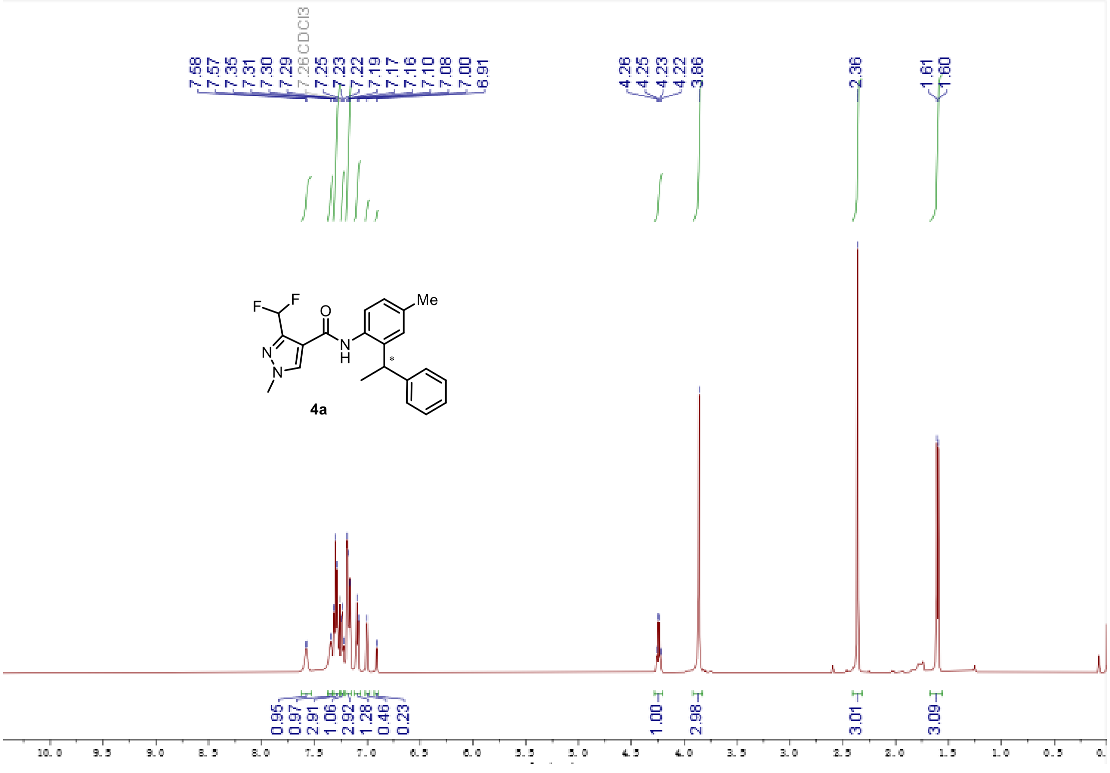
7.58 7.57 7.35 7.31 7.30 7.29 7.26 CDCl3 7.25 7.23 7.22 7.19 7.17 7.16 7.10 7.08 7.00 6.91
4.26 4.25 4.23 4.22 3.86
2.36
1.61 1.60
4a
Me
F
F
O
N
N
H
0.95 0.97 2.91 1.06 2.92 1.28 0.46 0.23
1.00
2.98
3.01
3.09

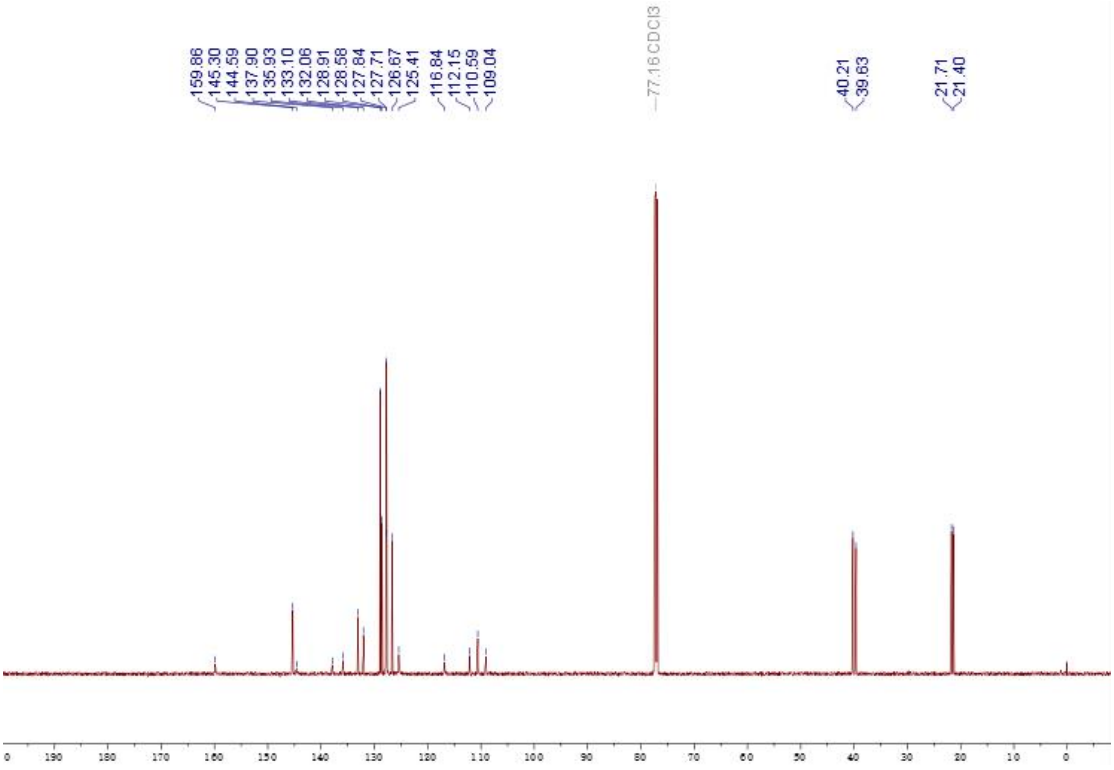
159.86 145.30 144.59 137.90 135.93 133.10 132.06 128.91 128.58 127.84 127.71 126.67 125.41 116.84 112.15 110.59 109.04
77.16 CDCl3
40.21 39.63
21.71 21.40

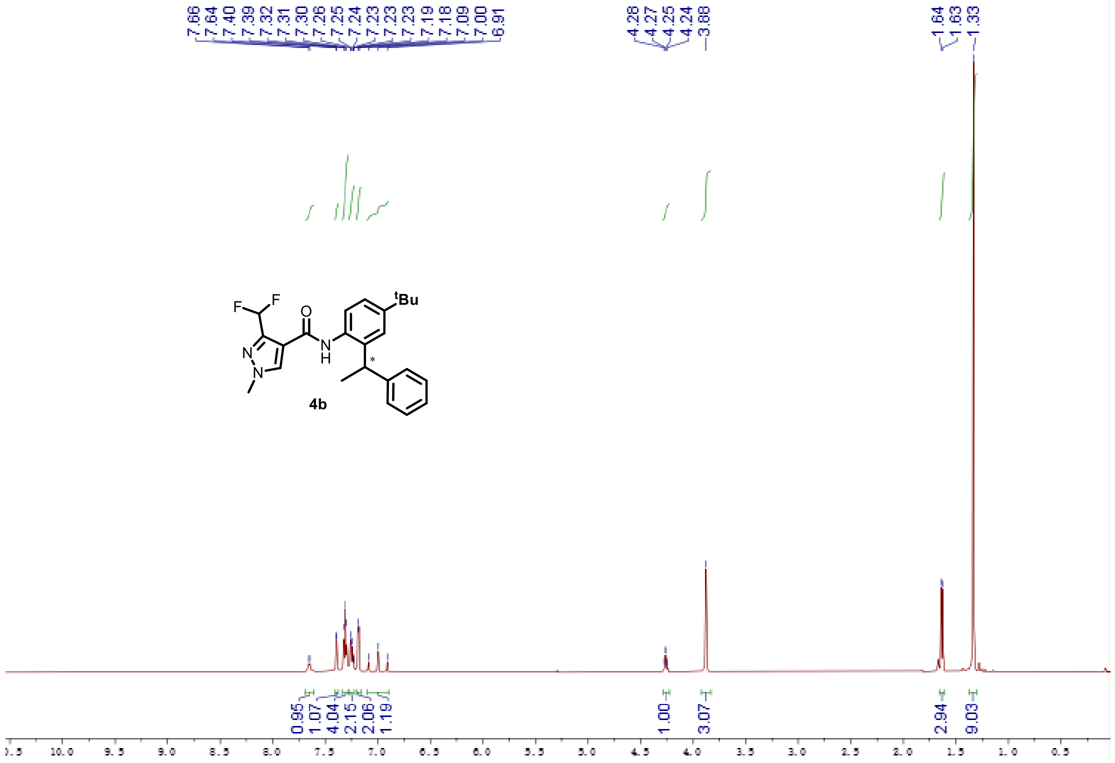

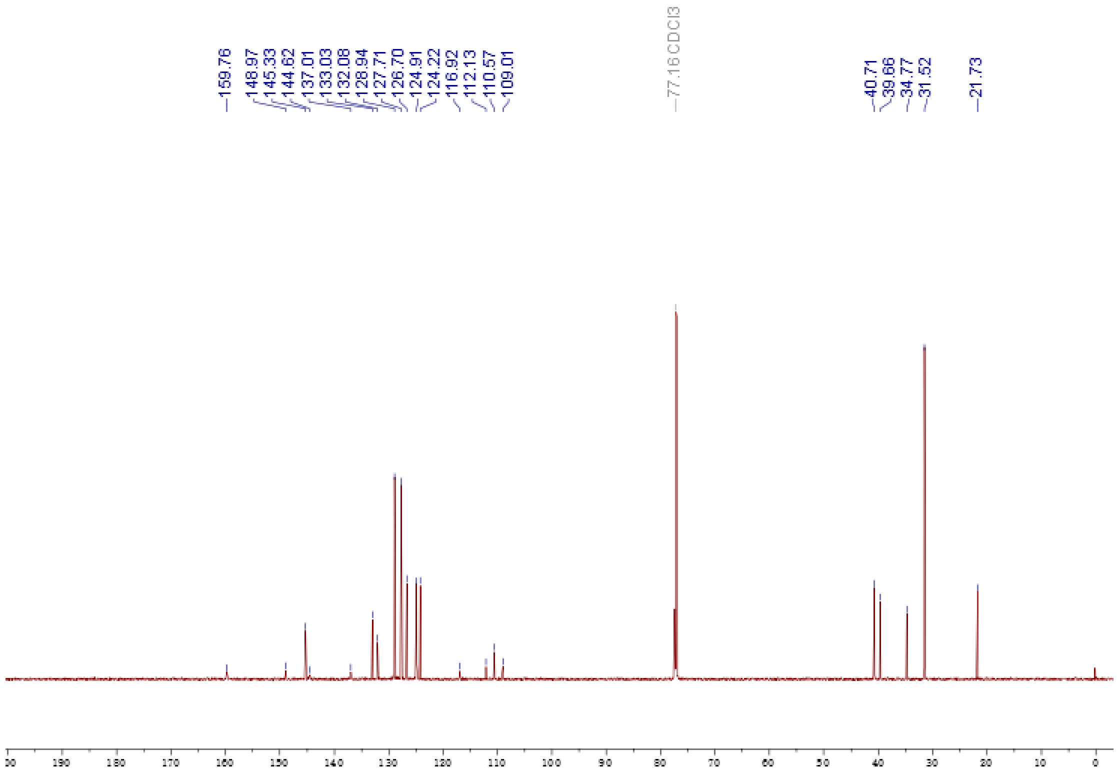

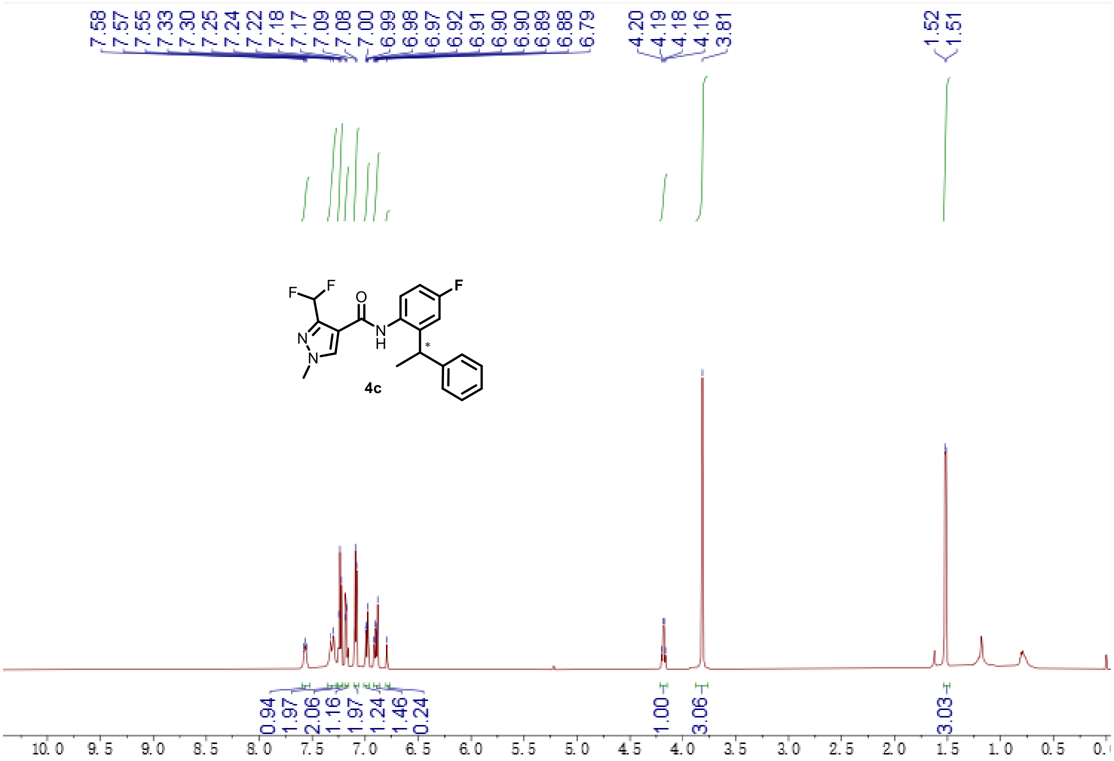
7.58 7.57 7.55 7.33 7.30 7.25 7.24 7.22 7.18 7.17 7.09 7.08 7.00 6.99 6.98 6.97 6.92 6.91 6.90 6.89 6.88 6.79
4.20 4.19 4.18 4.16 3.81
1.52 1.51
F F O N H F N N *
4c
0.94 1.97 2.06 1.16 1.97 1.24 1.46 0.24
1.00 3.06
3.03
10.0 9.5 9.0 8.5 8.0 7.5 7.0 6.5 6.0 5.5 5.0 4.5 4.0 3.5 3.0 2.5 2.0 1.5 1.0 0.5

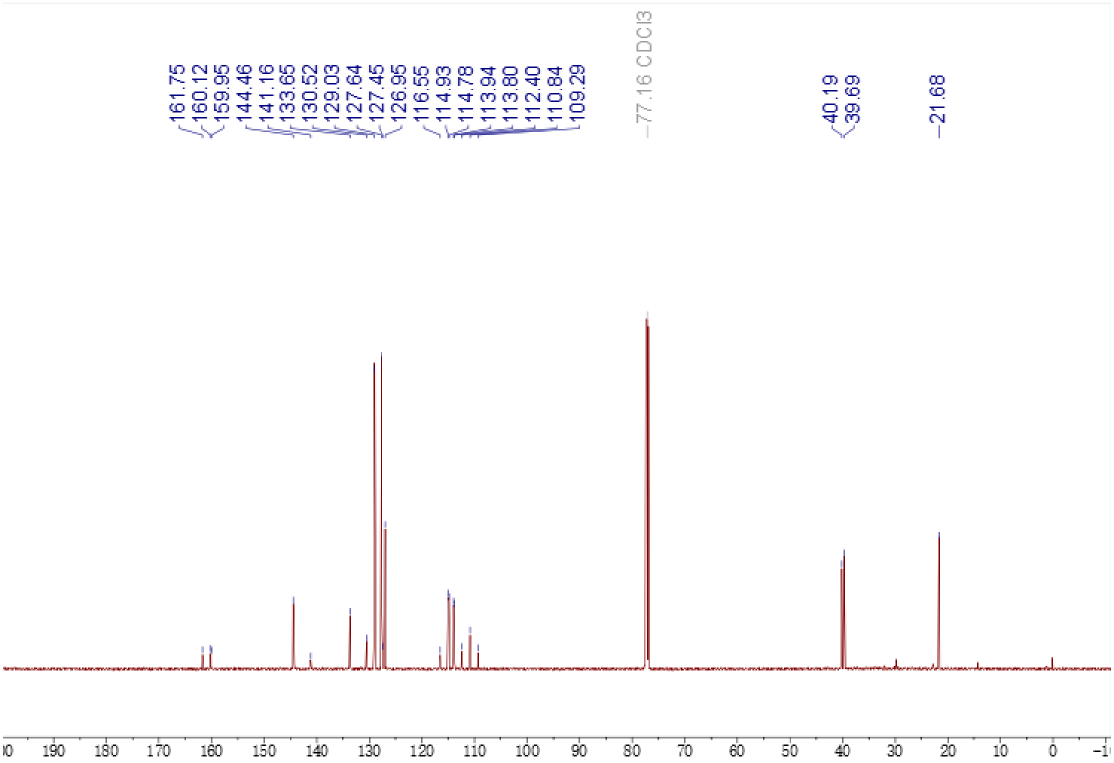
161.75 160.12 159.95 144.46 141.16 133.65 130.52 129.03 127.64 127.45 126.95 116.55 114.93 114.78 113.94 113.80 112.40 110.84 109.29
77.16 CDCl3
40.19 39.69
21.68
190 180 170 160 150 140 130 120 110 100 90 80 70 60 50 40 30 20 10 0

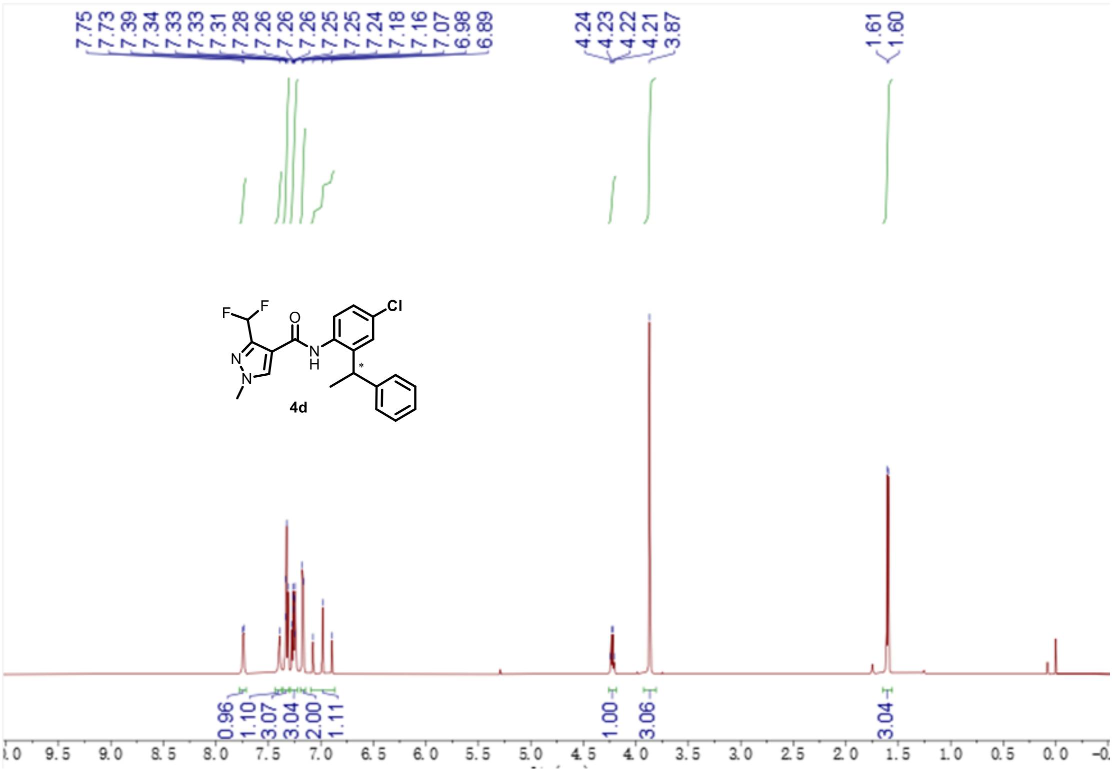
7.75 7.73 7.39 7.34 7.33 7.33 7.31 7.28 7.26 7.26 7.26 7.25 7.25 7.24 7.18 7.16 7.07 6.98 6.89
4.24 4.23 4.22 4.21 3.87
1.61 1.60
4d
0.96 1.10 3.07 3.04 2.00 1.11
1.00 3.06
3.04
9.5 9.0 8.5 8.0 7.5 7.0 6.5 6.0 5.5 5.0 4.5 4.0 3.5 3.0 2.5 2.0 1.5 1.0 0.5 0.0

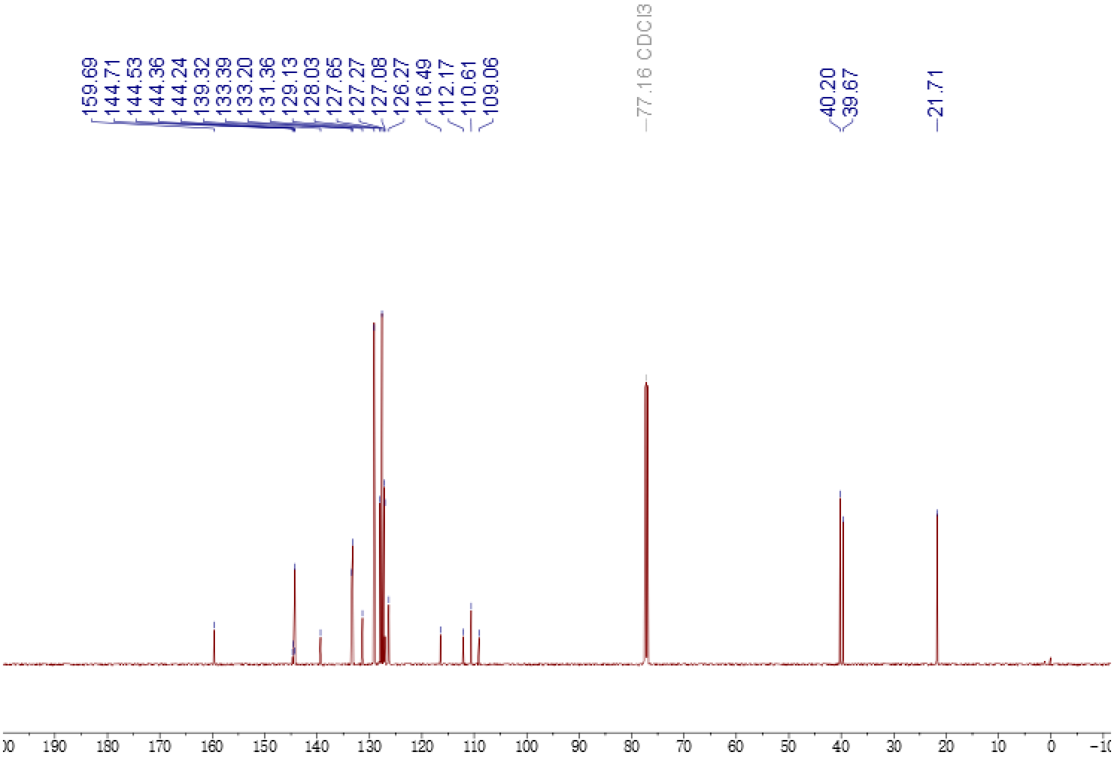
159.69 144.71 144.53 144.36 144.24 139.32 133.39 133.20 131.36 129.13 128.03 127.65 127.27 127.08 126.27 116.49 112.17 110.61 109.06
77.16 CDCl3
40.20 39.67
21.71
190 180 170 160 150 140 130 120 110 100 90 80 70 60 50 40 30 20 10 0

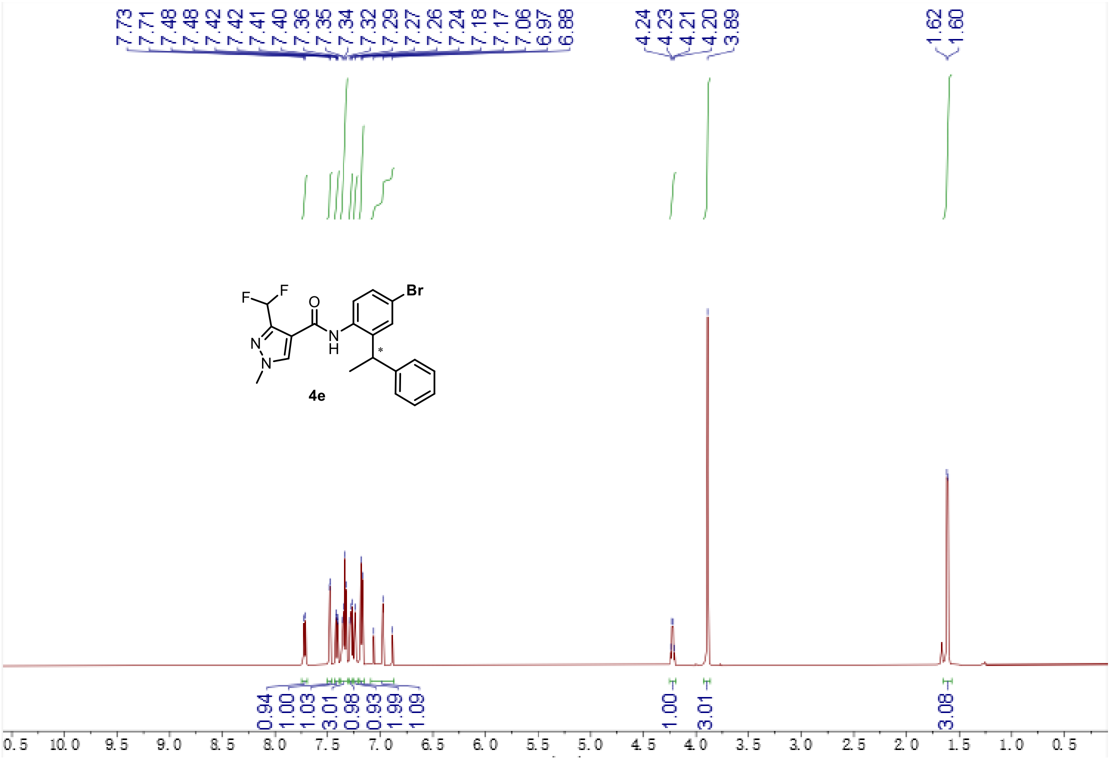
7.73
7.71
7.48
7.48
7.42
7.42
7.41
7.40
7.36
7.35
7.34
7.32
7.29
7.27
7.26
7.24
7.18
7.17
7.06
6.97
6.88
4.24
4.23
4.21
4.20
3.89
1.62
1.60
F
F
O
N
H
N
N
Br
4e
0.94
1.00
1.03
3.01
0.98
0.93
1.99
1.09
1.00
3.01
3.08

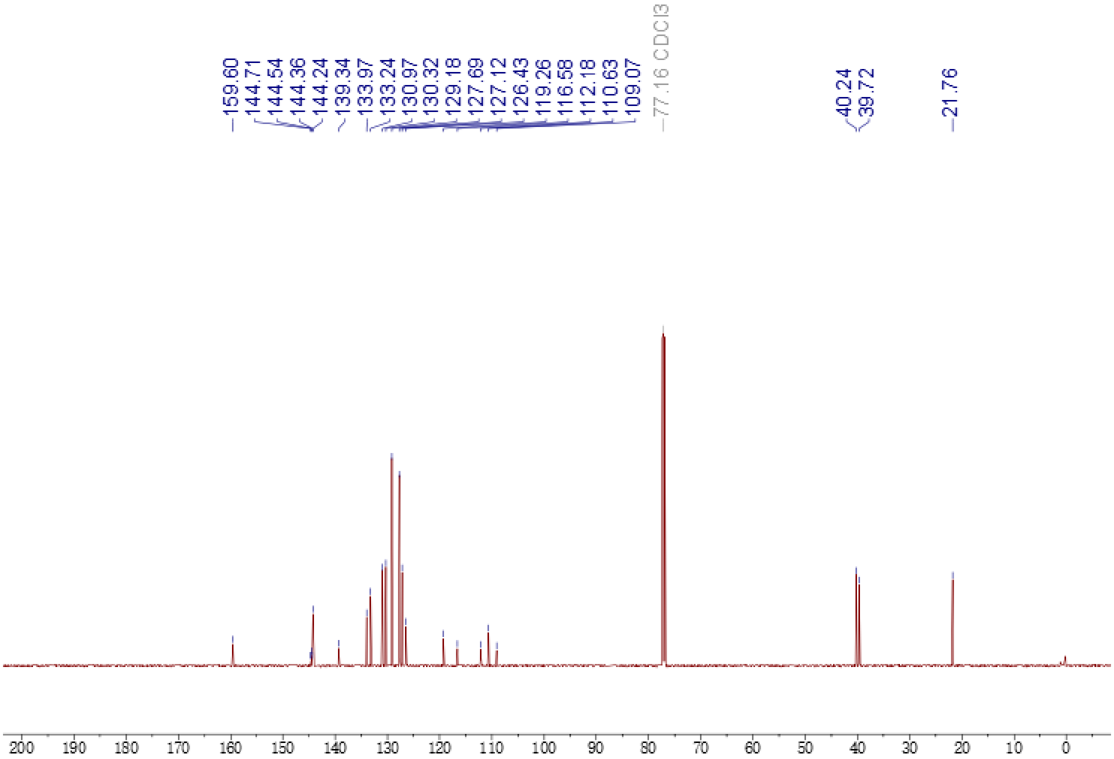
159.60
144.71
144.54
144.36
144.24
139.34
133.97
133.24
130.97
130.32
129.18
127.69
127.12
126.43
119.26
116.58
112.18
110.63
109.07
77.16 CDCl3
40.24
39.72
21.76

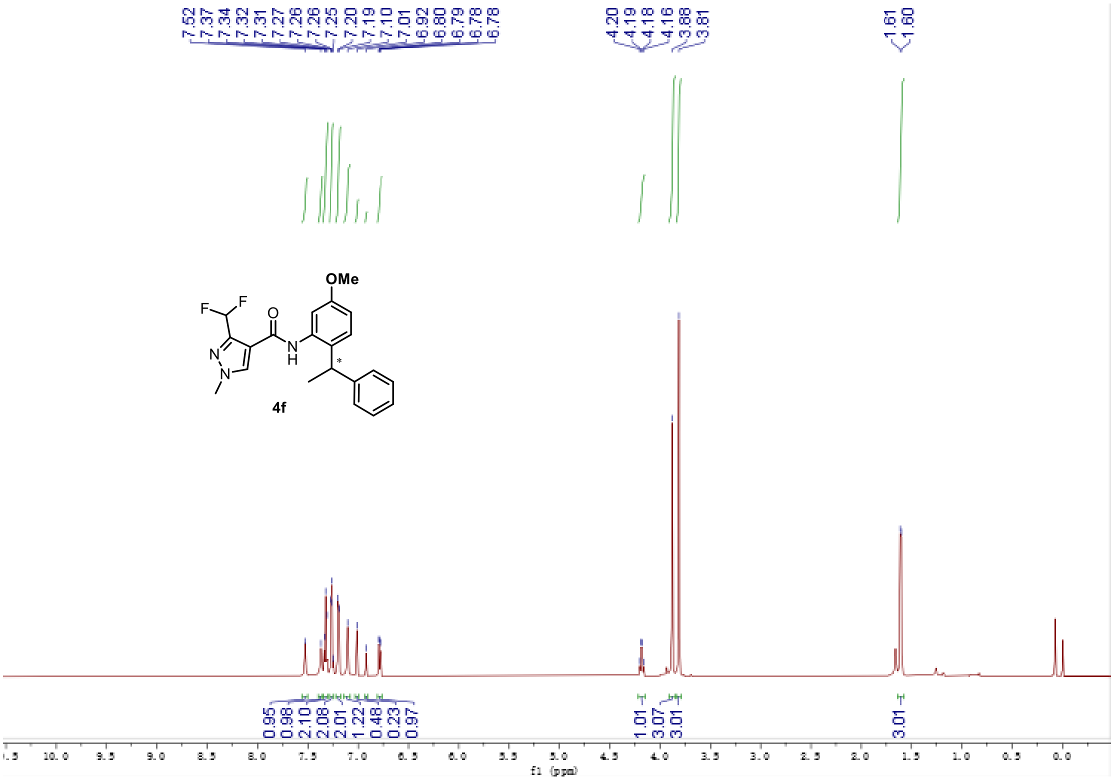
7.52 7.37 7.34 7.32 7.31 7.27 7.26 7.26 7.25 7.20 7.19 7.10 7.01 6.92 6.80 6.79 6.78 6.78
4.20 4.19 4.18 4.16 3.88 3.81
1.61 1.60
OMe
F
F
O
N
N
N
H
*
4f
0.95 0.98 2.10 2.08 2.01 1.22 0.48 0.23 0.97
1.01 3.07 3.01
3.01
f1 (ppm)

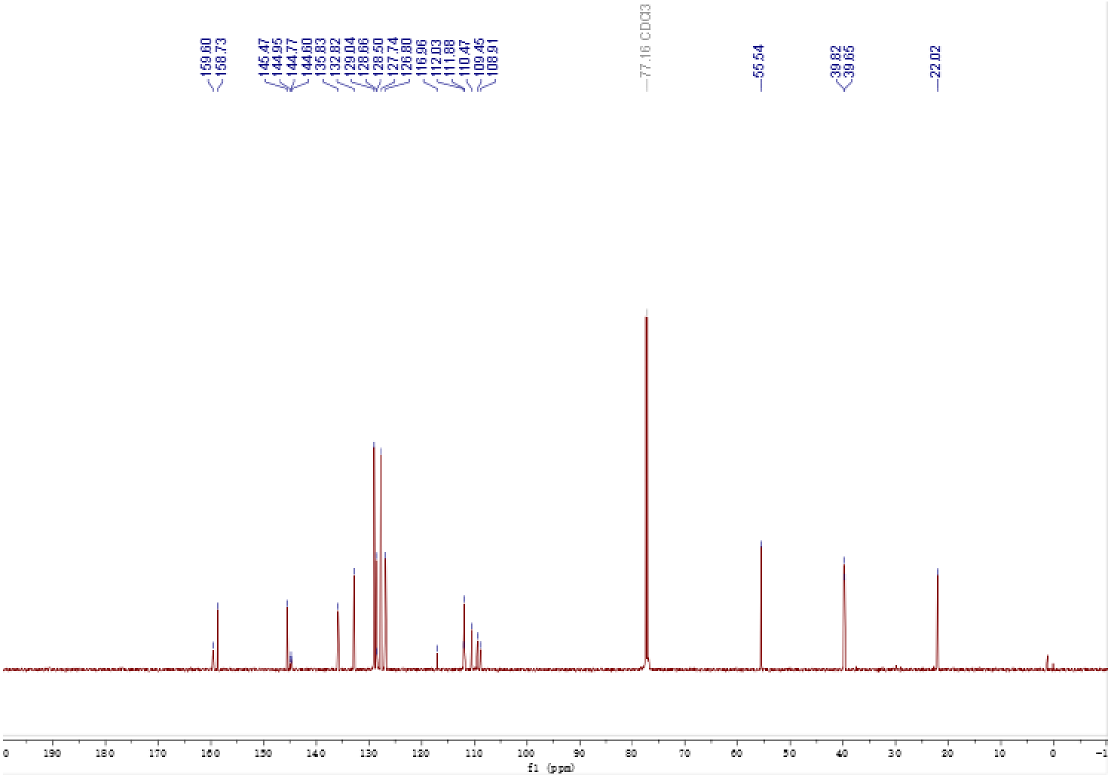
159.60 158.73
145.47 144.95 144.77 144.60 135.83 132.82 129.04 128.66 128.50 127.74 126.80 116.96 112.03 111.88 110.47 109.45 108.91
77.16 CDCl3
55.54
39.82 39.65
22.02
f1 (ppm)

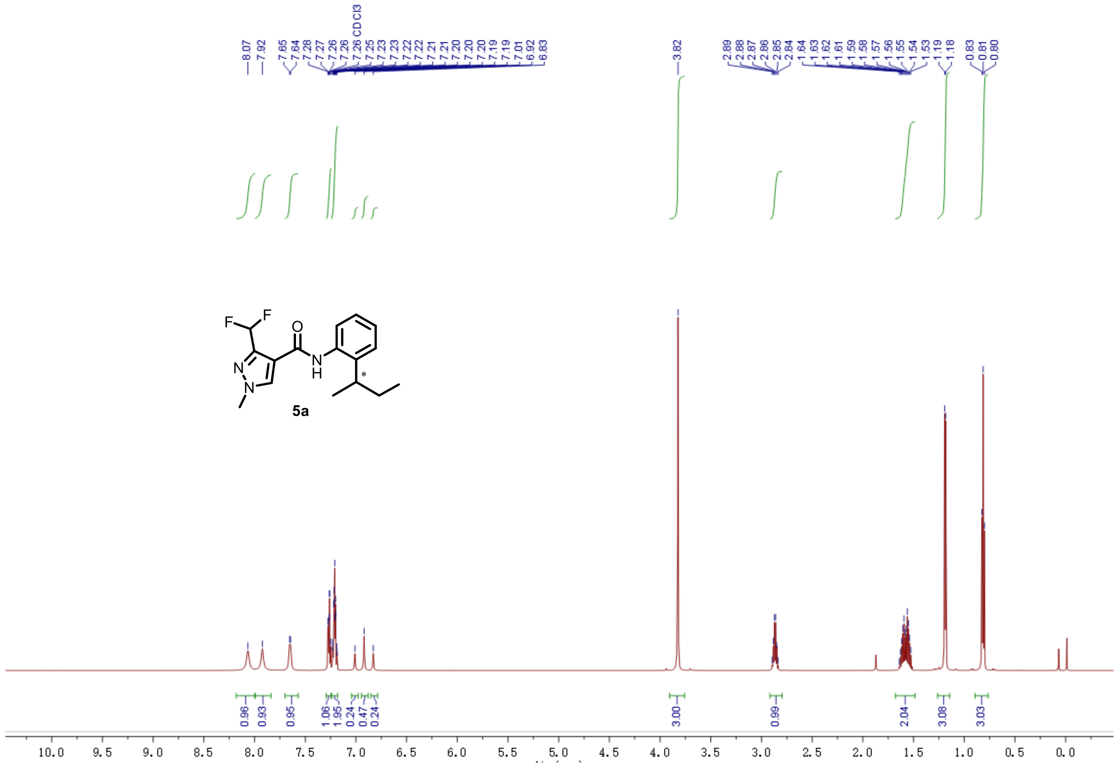
8.07
7.92
7.65
7.64
7.28
7.27
7.26
7.26
7.26 CDCl3
7.25
7.23
7.23
7.22
7.22
7.21
7.21
7.20
7.20
7.20
7.19
7.19
7.01
6.92
6.83
3.82
2.89
2.88
2.87
2.86
2.85
2.84
1.64
1.63
1.62
1.61
1.59
1.58
1.57
1.56
1.55
1.54
1.53
1.19
1.18
0.83
0.81
0.80
F
F
O
N
N
N
H
*
5a
0.96
0.93
0.95
1.06
1.95
0.24
0.47
0.24
3.00
0.99
2.04
3.08
3.03
10.0
9.5
9.0
8.5
8.0
7.5
7.0
6.5
6.0
5.5
5.0
4.5
4.0
3.5
3.0
2.5
2.0
1.5
1.0
0.5
0.0

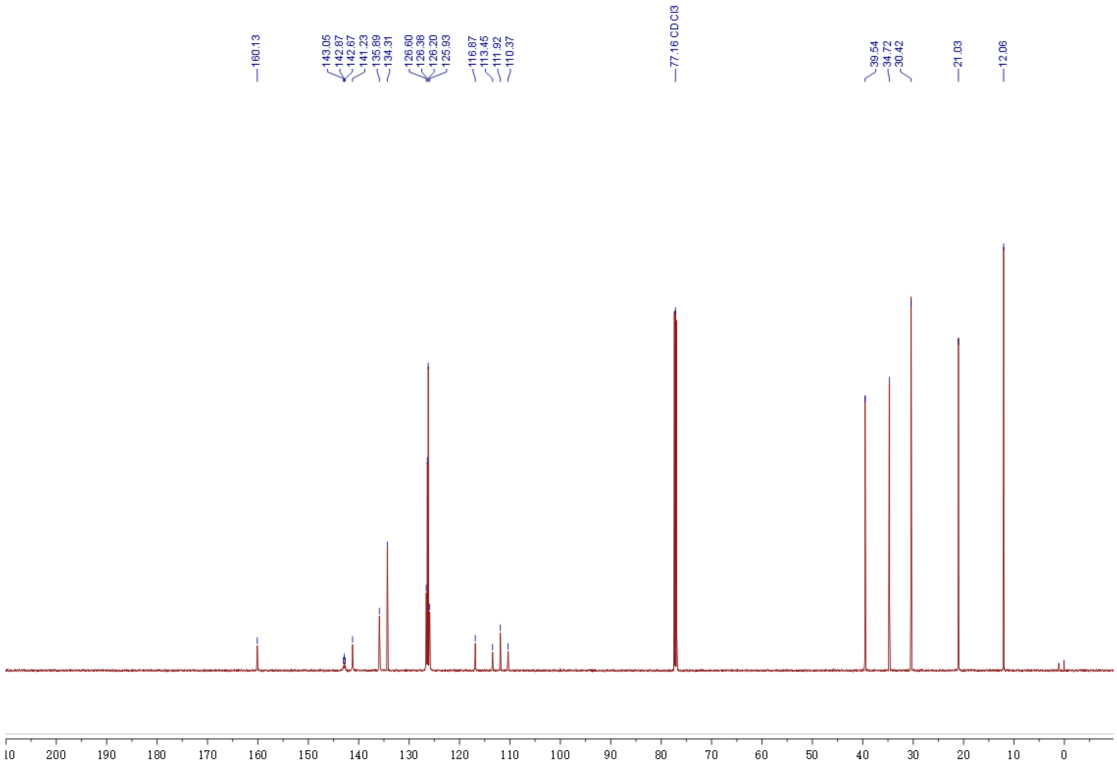
160.13
143.05
142.87
142.67
141.23
135.89
134.31
126.60
126.38
126.20
125.93
116.87
113.45
111.92
110.37
77.16 CDCl3
39.54
34.72
30.42
21.03
12.06
200
190
180
170
160
150
140
130
120
110
100
90
80
70
60
50
40
30
20
10
0

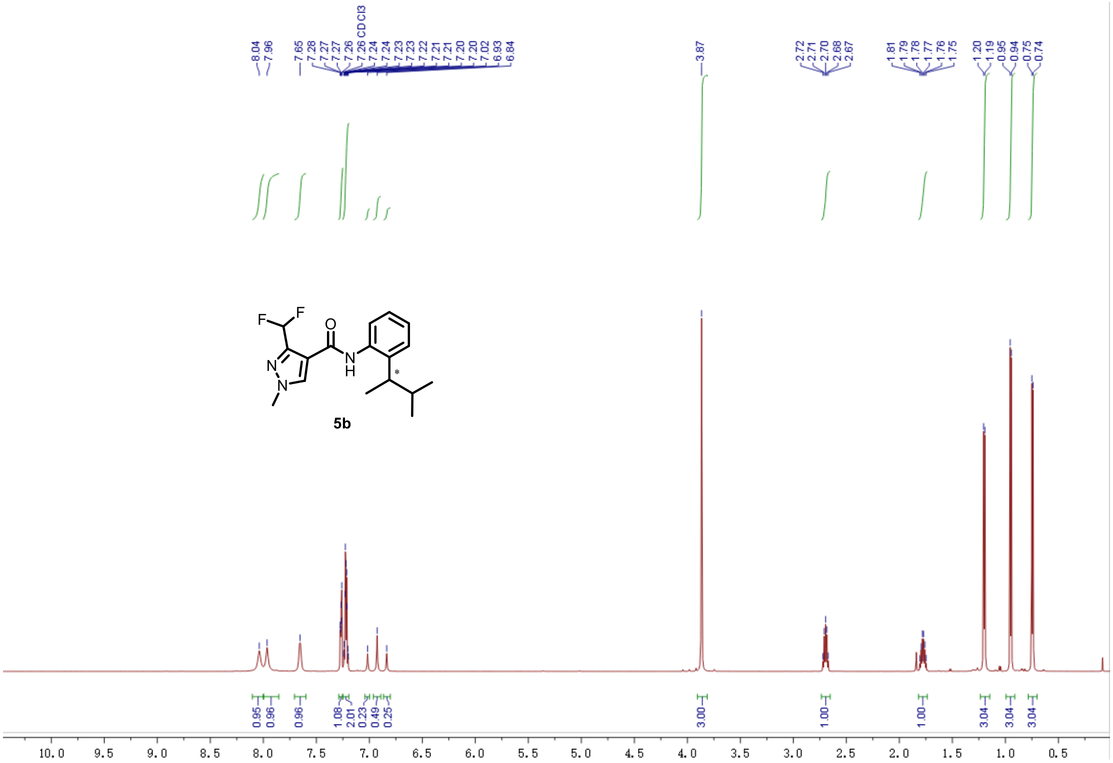
8.04
7.96
7.65
7.28
7.27
7.26
7.26 CDCl3
7.24
7.24
7.23
7.23
7.22
7.21
7.21
7.20
7.20
7.02
6.93
6.84
3.87
2.72
2.71
2.70
2.68
2.67
1.81
1.79
1.78
1.77
1.76
1.75
1.20
1.19
0.95
0.94
0.75
0.74
F
F
O
N
N
N
H
*
5b
0.95
0.96
0.96
1.08
2.01
0.23
0.49
0.25
3.00
1.00
1.00
3.04
3.04
3.04
10.0 9.5 9.0 8.5 8.0 7.5 7.0 6.5 6.0 5.5 5.0 4.5 4.0 3.5 3.0 2.5 2.0 1.5 1.0 0.5

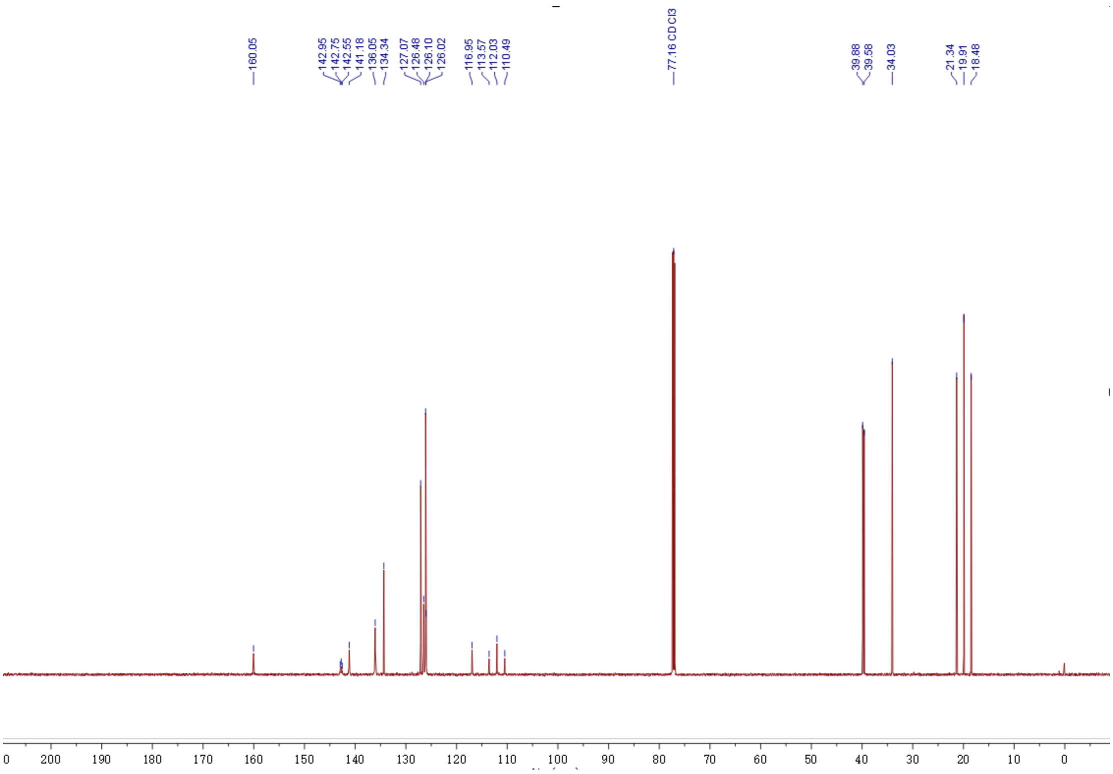
160.05
142.95
142.75
142.55
141.18
136.05
134.34
127.07
126.48
126.10
126.02
116.95
113.57
112.03
110.49
77.16 CDCl3
39.88
39.58
34.03
21.34
19.91
18.48
0 200 190 180 170 160 150 140 130 120 110 100 90 80 70 60 50 40 30 20 10 0

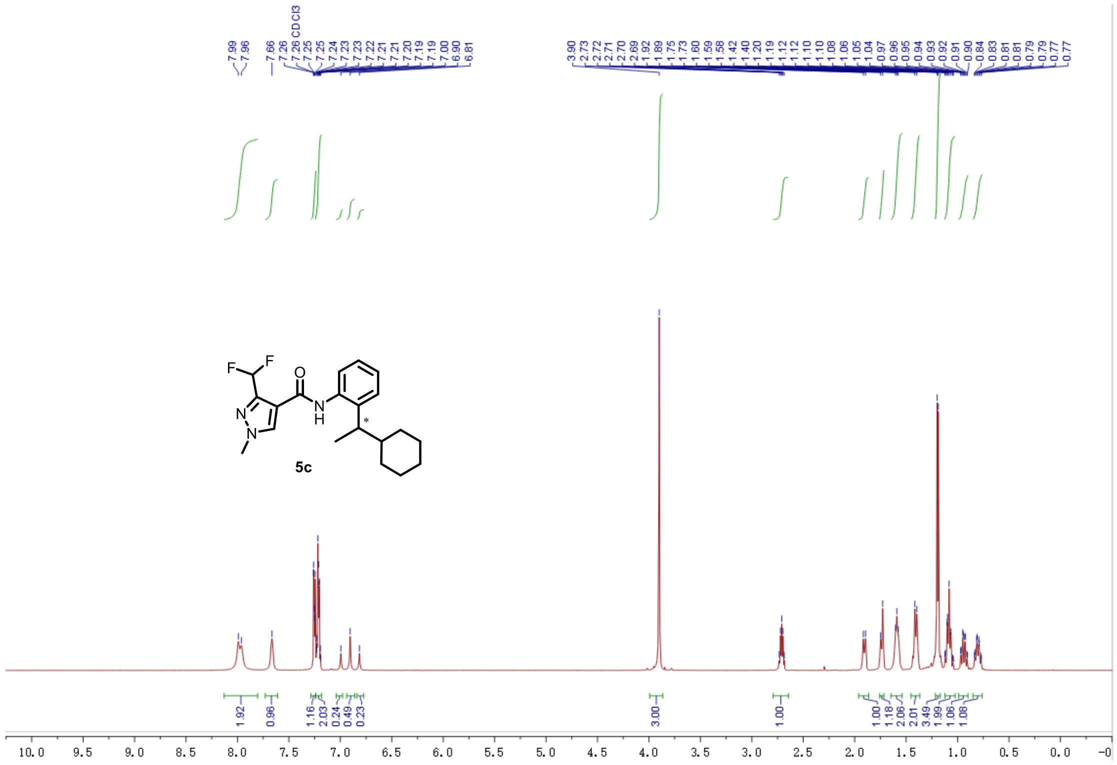
7.99
7.96
7.66
7.26 CDCl3
7.25
7.25
7.24
7.23
7.23
7.22
7.21
7.21
7.20
7.19
7.19
7.00
6.90
6.81
3.90
2.73
2.72
2.71
2.70
2.69
1.92
1.89
1.75
1.73
1.60
1.59
1.58
1.42
1.40
1.20
1.19
1.12
1.12
1.10
1.10
1.08
1.06
1.05
1.04
0.97
0.96
0.95
0.94
0.93
0.92
0.91
0.90
0.84
0.83
0.81
0.81
0.79
0.77
0.77
F
F
N
N
O
N
H
*
5c
1.92
0.96
1.16
2.03
0.24
0.49
0.23
3.00
1.00
1.00
1.18
2.06
2.01
3.49
1.99
1.06
1.08
10.0 9.5 9.0 8.5 8.0 7.5 7.0 6.5 6.0 5.5 5.0 4.5 4.0 3.5 3.0 2.5 2.0 1.5 1.0 0.5 0.0

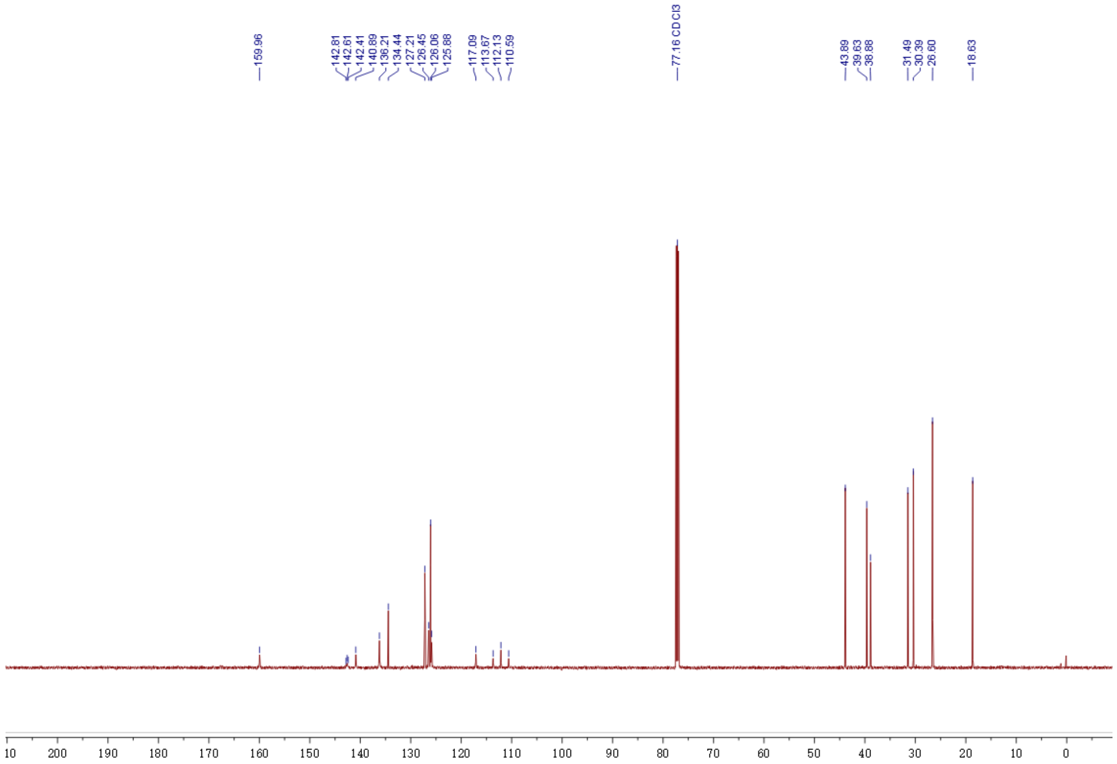
159.96
142.81
142.61
142.41
140.89
136.21
134.44
127.21
126.45
126.06
125.88
117.09
113.67
112.13
110.59
77.16 CDCl3
43.89
39.63
38.88
31.49
30.39
26.60
18.63
200 190 180 170 160 150 140 130 120 110 100 90 80 70 60 50 40 30 20 10 0

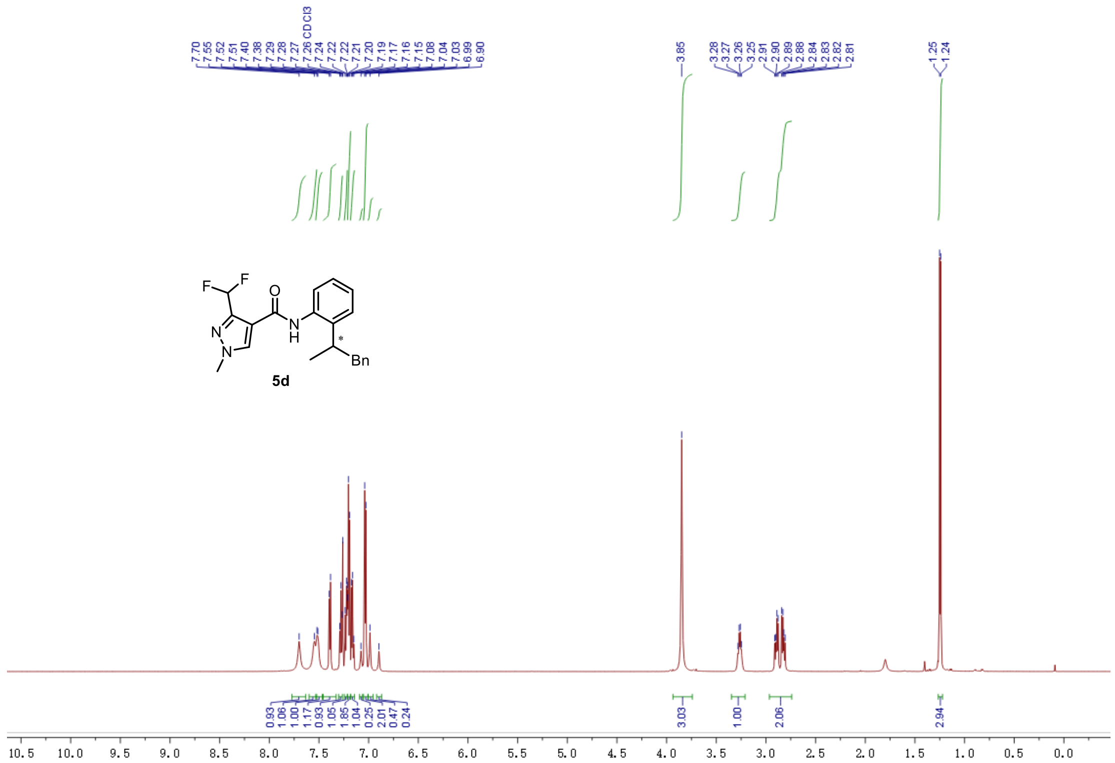
7.70 7.55 7.52 7.51 7.40 7.38 7.29 7.28 7.27 7.26 CDCl3 7.24 7.22 7.22 7.21 7.20 7.19 7.17 7.16 7.15 7.08 7.04 7.03 6.99 6.90
3.85
3.28 3.27 3.26 3.25 2.91 2.90 2.89 2.88 2.84 2.83 2.82 2.81
1.25 1.24
F F O N H N N * Bn
5d
0.93 1.06 1.00 1.17 0.93 1.05 1.85 1.04 0.25 2.01 0.47 0.24
3.03
1.00
2.06
2.94
10.5 10.0 9.5 9.0 8.5 8.0 7.5 7.0 6.5 6.0 5.5 5.0 4.5 4.0 3.5 3.0 2.5 2.0 1.5 1.0 0.5 0.0

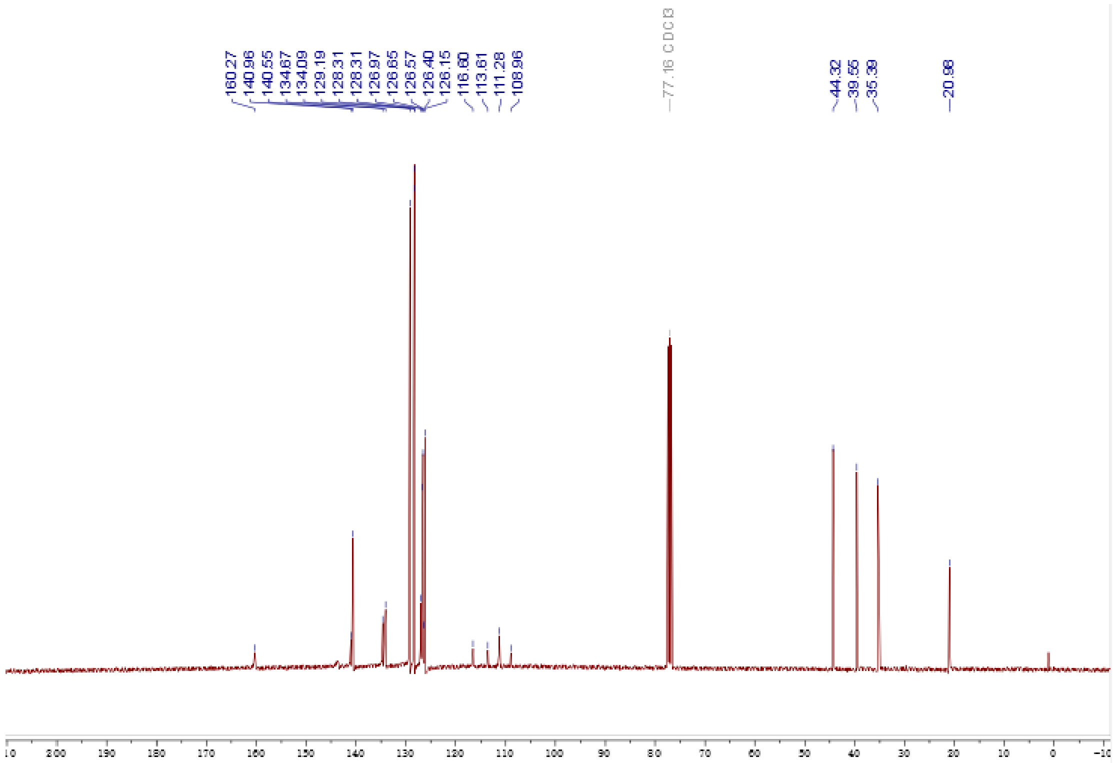
160.27 140.96 140.55 134.67 134.09 129.19 128.31 128.31 126.97 126.65 126.57 126.40 126.15 116.80 113.61 111.28 108.96
77.16 CDCl3
44.32 39.55 35.39
20.98
200 190 180 170 160 150 140 130 120 110 100 90 80 70 60 50 40 30 20 10 0

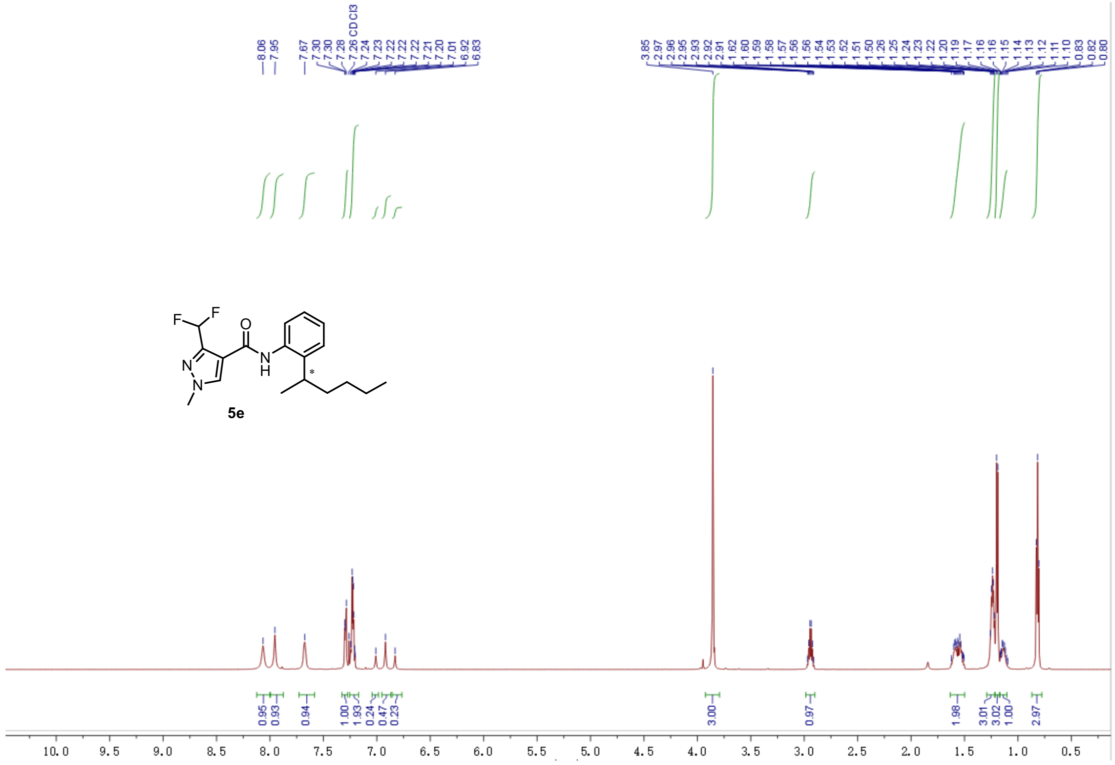
8.06
7.95
7.67
7.30
7.30
7.28
7.26 CDCl3
7.24
7.23
7.22
7.22
7.21
7.20
7.01
6.92
6.83
3.85
2.97
2.96
2.95
2.93
2.92
2.91
1.62
1.60
1.59
1.58
1.57
1.56
1.54
1.53
1.52
1.51
1.50
1.26
1.25
1.24
1.23
1.22
1.20
1.19
1.17
1.16
1.15
1.14
1.13
1.12
1.11
1.10
0.83
0.82
0.80
5e
0.95
0.93
0.94
1.00
1.93
0.24
0.47
0.23
3.00
0.97
1.98
3.01
3.02
1.00
2.97

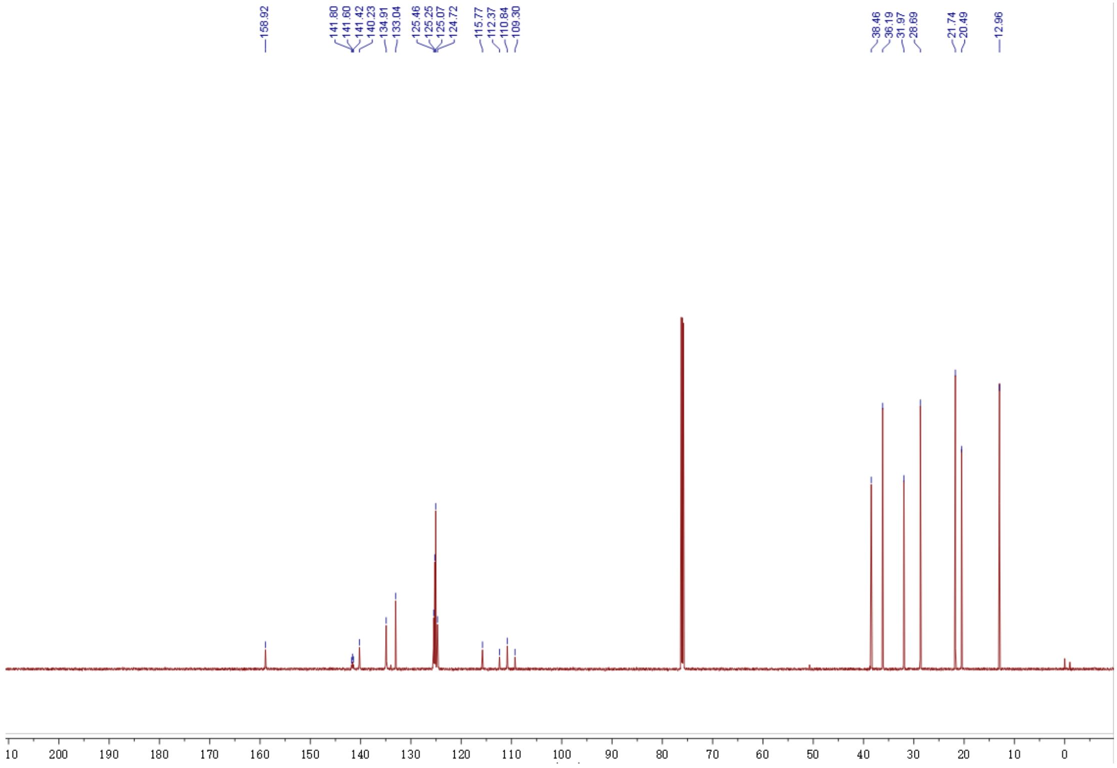
158.92
141.80
141.60
141.42
140.23
134.91
133.04
125.46
125.25
125.07
124.72
115.77
112.37
110.84
109.30
38.46
36.19
31.97
28.69
21.74
20.49
12.96

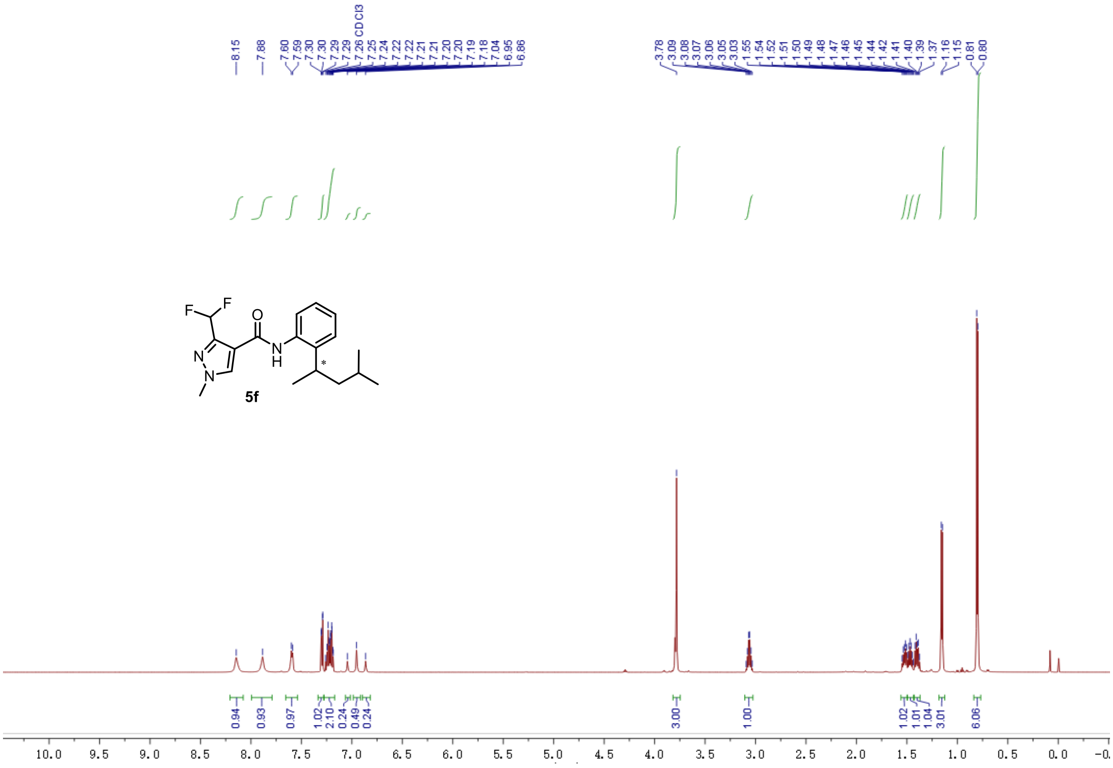

8.15
7.88
7.60
7.59
7.30
7.30
7.29
7.29
7.26 CDCl3
7.25
7.24
7.22
7.22
7.21
7.21
7.20
7.20
7.19
7.18
7.04
6.95
6.86
3.78
3.09
3.08
3.07
3.06
3.05
3.03
1.55
1.54
1.52
1.51
1.50
1.49
1.48
1.47
1.46
1.45
1.44
1.42
1.41
1.40
1.39
1.37
1.16
1.15
0.81
0.80
F
F
O
N
N
H
*
5f
0.94
0.93
0.97
1.02
2.10
0.24
0.49
0.24
3.00
1.00
1.02
1.01
1.04
3.01
6.06
10.0 9.5 9.0 8.5 8.0 7.5 7.0 6.5 6.0 5.5 5.0 4.5 4.0 3.5 3.0 2.5 2.0 1.5 1.0 0.5 0.0

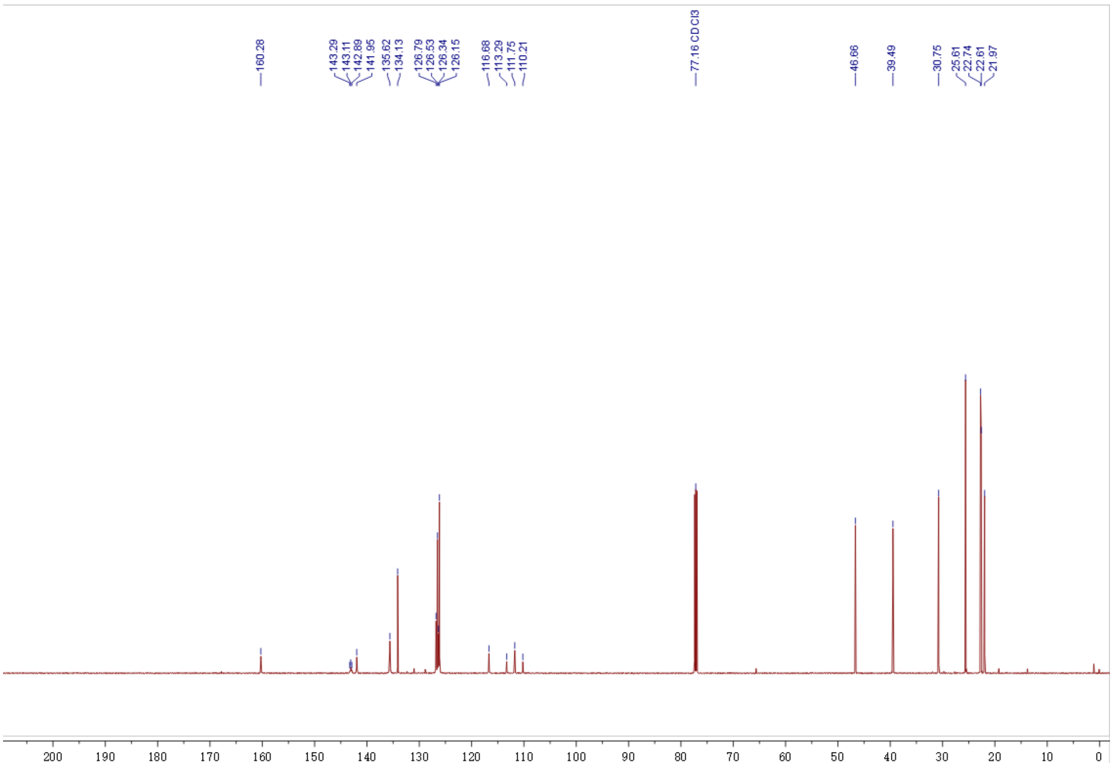

160.28
143.29
143.11
142.89
141.95
135.62
134.13
126.79
126.53
126.34
126.15
116.68
113.29
111.75
110.21
77.16 CDCl3
46.66
39.49
30.75
25.61
22.74
22.61
21.97
200 190 180 170 160 150 140 130 120 110 100 90 80 70 60 50 40 30 20 10 0

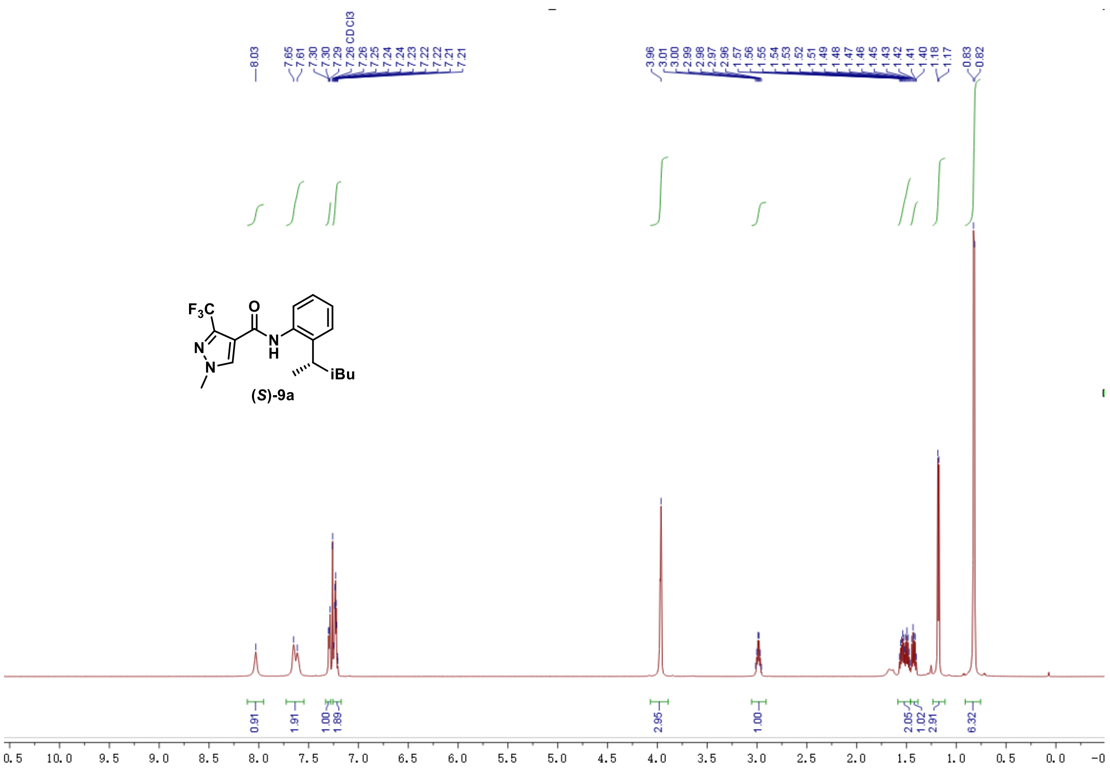
8.03
7.65
7.61
7.30
7.30
7.29
7.26 CDCl3
7.26
7.25
7.24
7.24
7.23
7.22
7.22
7.21
7.21
3.96
3.01
3.00
2.99
2.98
2.97
2.96
1.57
1.56
1.55
1.54
1.53
1.52
1.51
1.49
1.48
1.47
1.46
1.45
1.43
1.42
1.41
1.40
1.18
1.17
0.83
0.82
F3C
O
N
H
N
N
iBu
(S)-9a
0.91
1.91
1.00
1.89
2.95
1.00
2.05
1.02
2.91
6.32

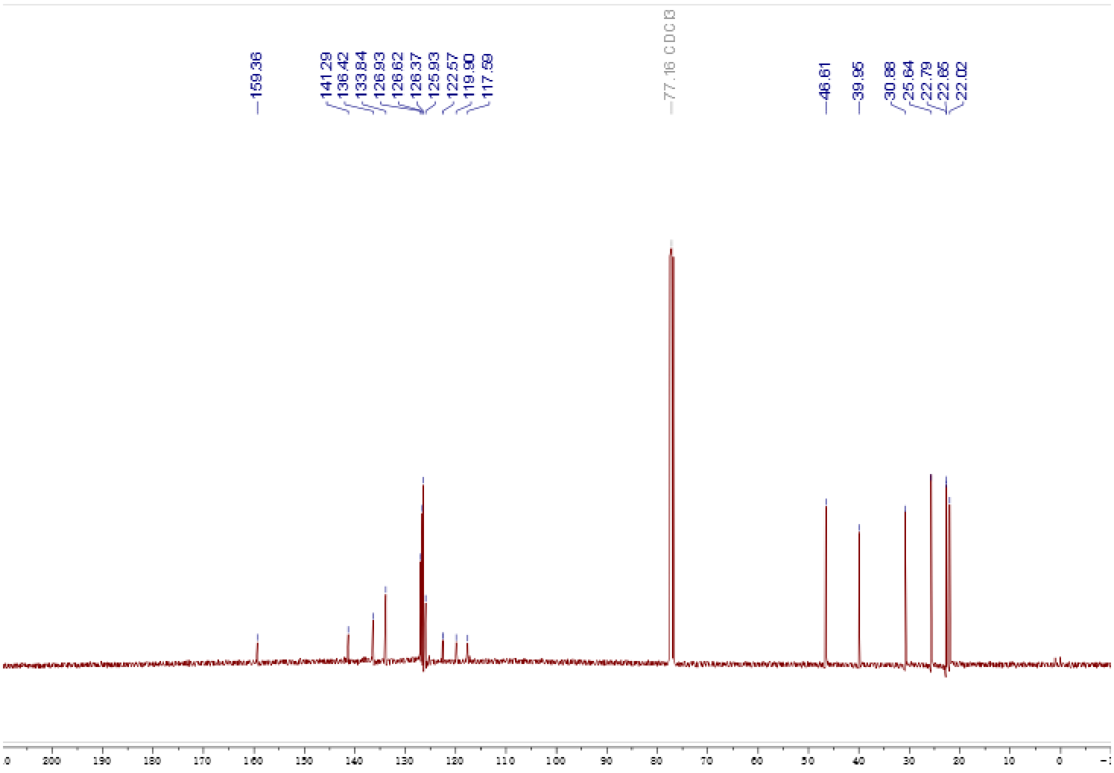
159.36
141.29
136.42
133.84
126.93
126.62
126.37
125.93
122.57
119.90
117.59
77.16 CDCl3
46.61
39.95
30.88
25.64
22.79
22.65
22.02

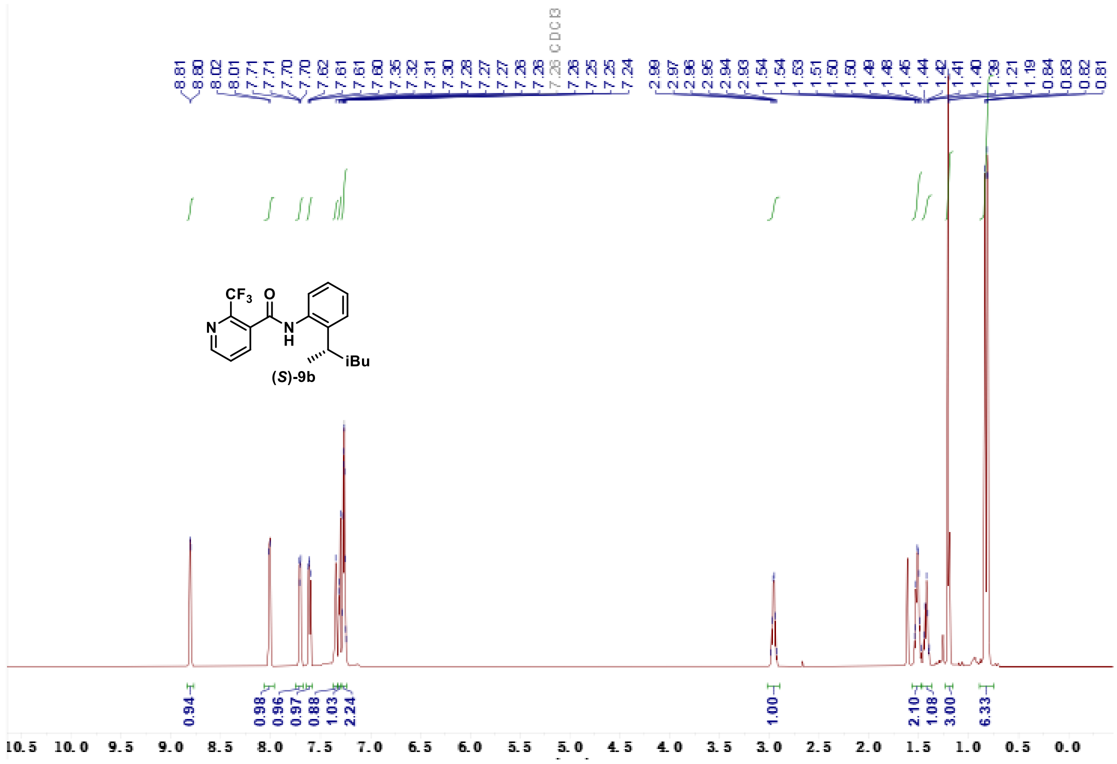

(S)-9b
iBu
CF3
N
H
O
7.26 CDCl3
0.94
0.98
0.96
0.97
0.88
1.03
2.24
1.00
2.10
1.08
3.00
6.33

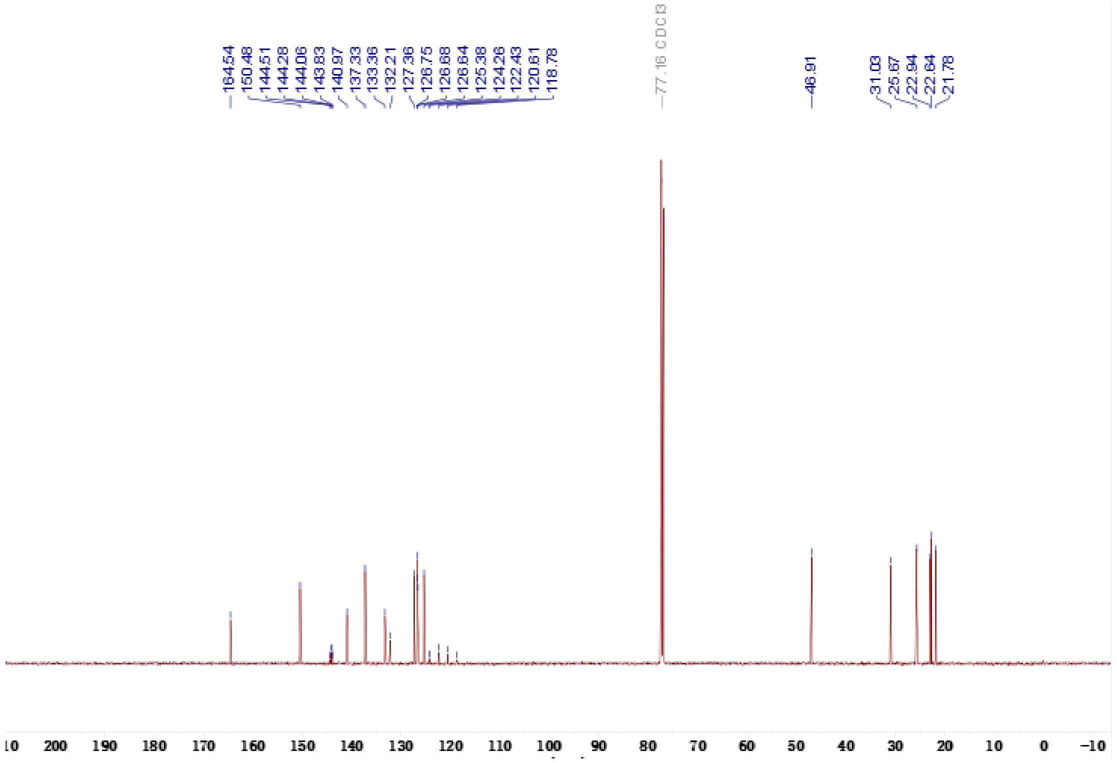

77.16 CDCl3
164.54
150.48
46.91
31.03
25.67
22.94
22.64
21.78

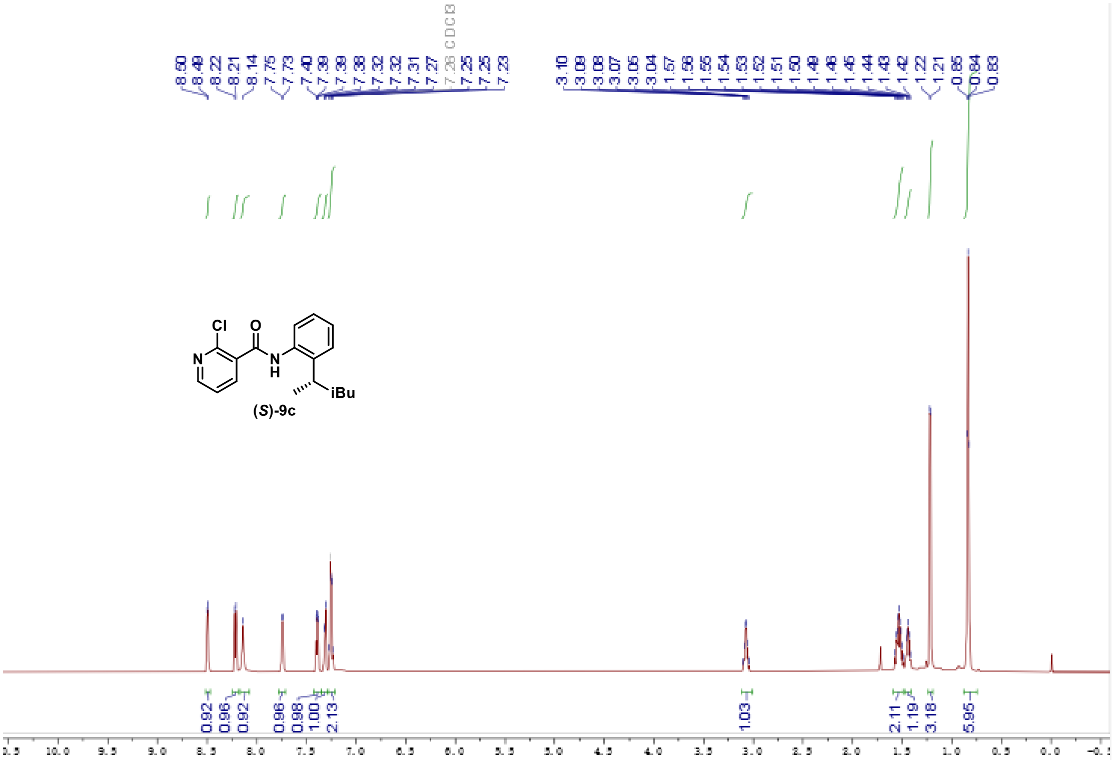
(S)-9c

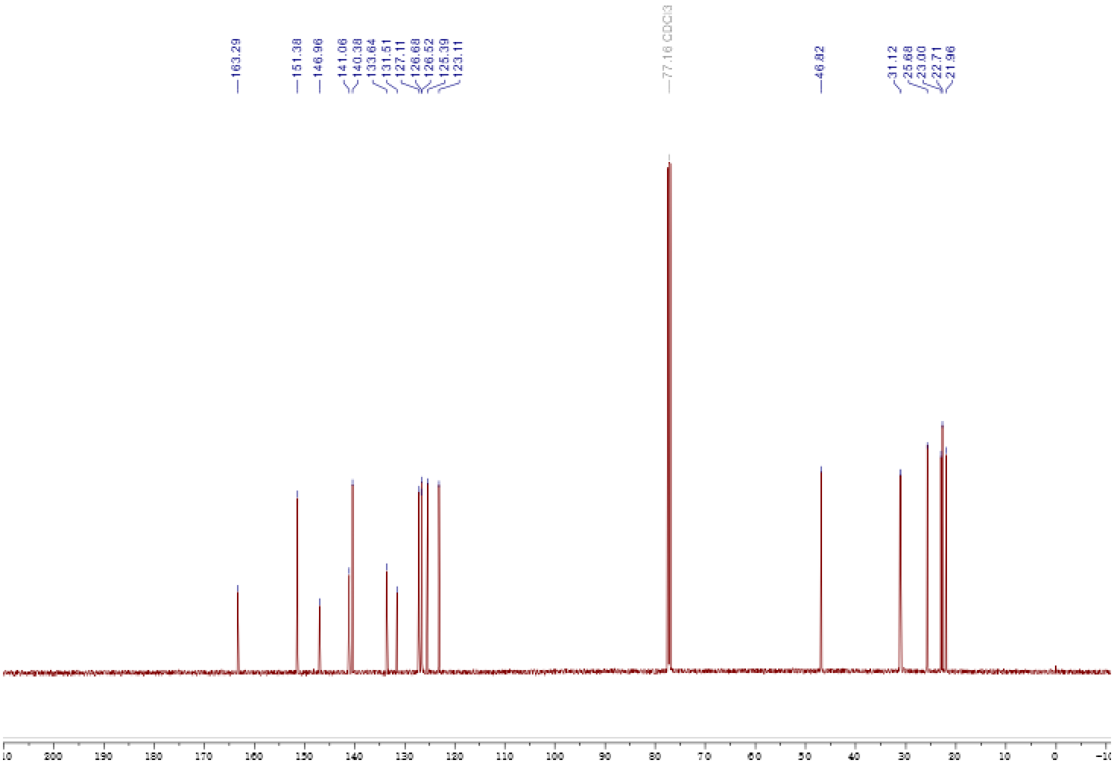

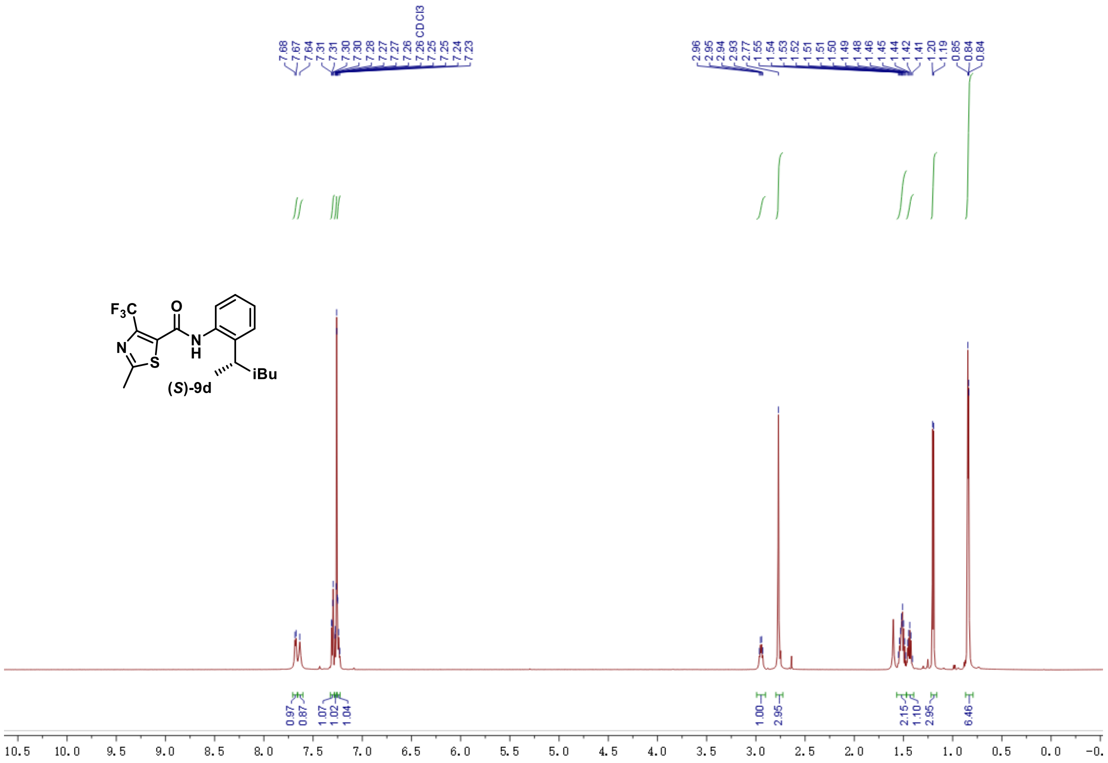
7.68 7.67 7.64 7.31 7.31 7.30 7.30 7.28 7.27 7.27 7.26 CDCl3 7.25 7.25 7.24 7.23
2.96 2.95 2.94 2.93 2.77 1.55 1.54 1.53 1.52 1.51 1.51 1.50 1.49 1.48 1.46 1.45 1.44 1.42 1.41 1.20 1.19 0.85 0.84 0.84
F3C
O
N
S
N
H
iBu
(S)-9d
0.97 0.87 1.07 1.02 1.04
1.00 2.95
2.15 1.10 2.95
6.46
10.5 10.0 9.5 9.0 8.5 8.0 7.5 7.0 6.5 6.0 5.5 5.0 4.5 4.0 3.5 3.0 2.5 2.0 1.5 1.0 0.5 0.0 -0.

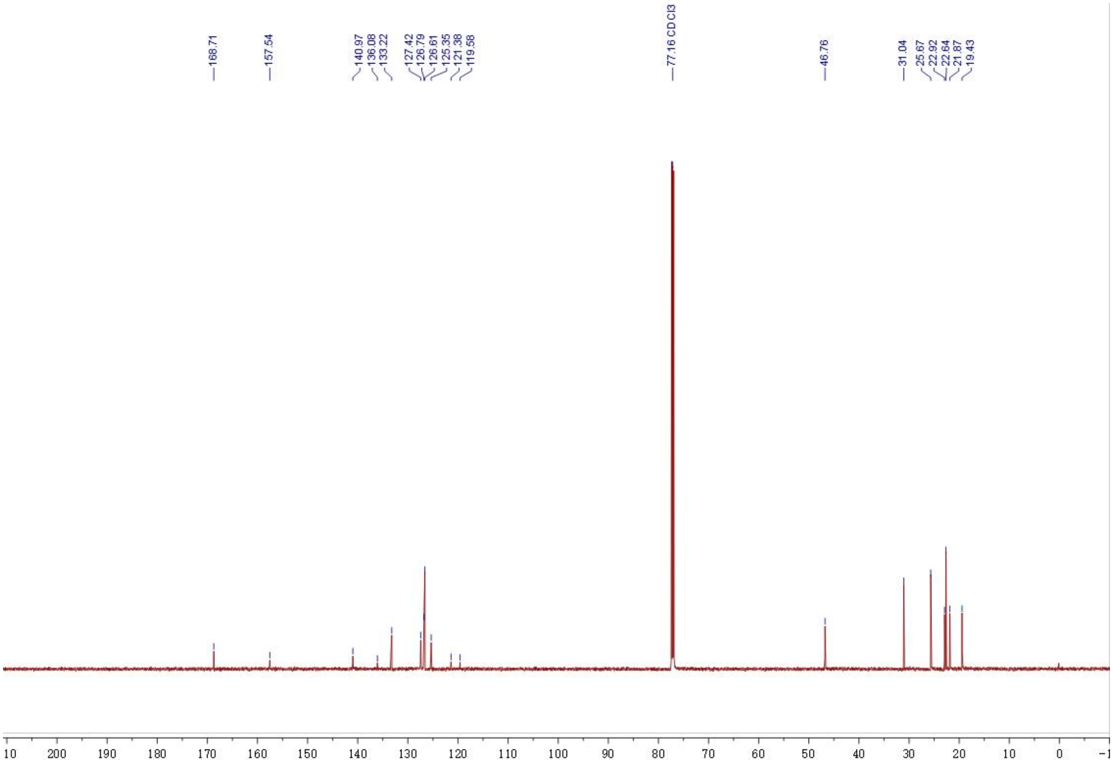
168.71
157.54
140.97 136.08 133.22
127.42 126.79 126.61 125.35 121.38 119.58
77.16 CDCl3
46.76
31.04 25.67 22.92 22.64 21.87 19.43
10 200 190 180 170 160 150 140 130 120 110 100 90 80 70 60 50 40 30 20 10 0 -1

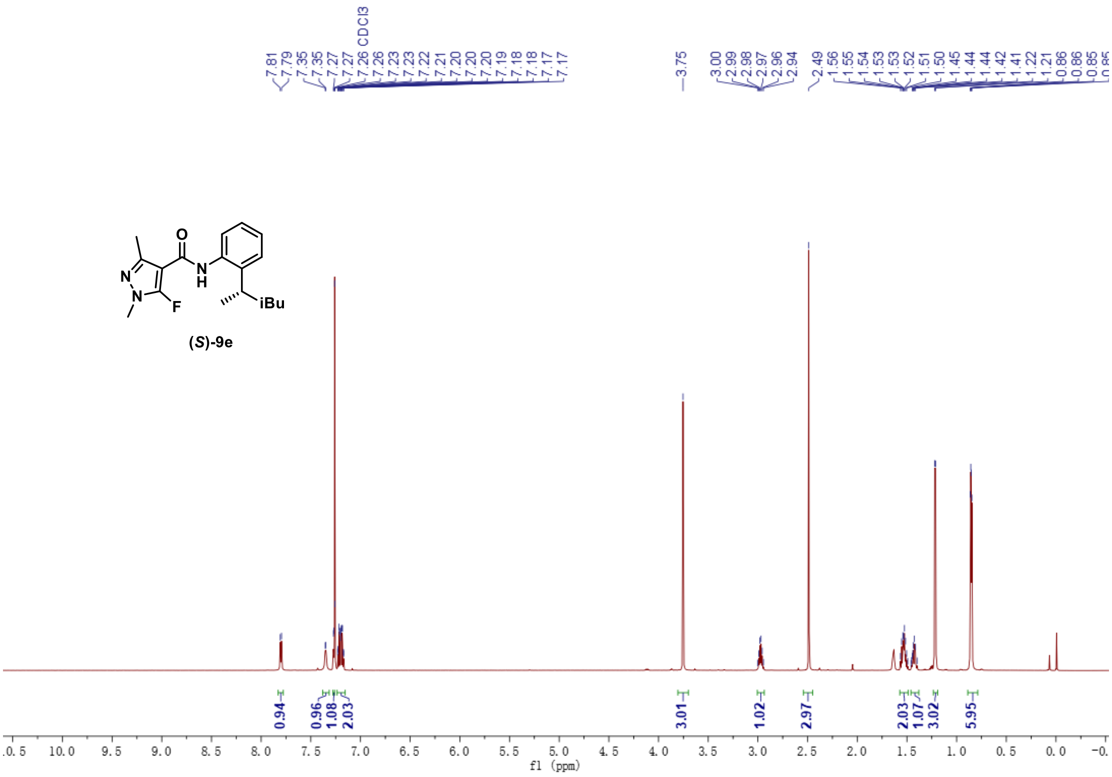
7.81
7.79
7.35
7.35
7.27
7.26 CDCl3
7.26
7.23
7.23
7.22
7.21
7.20
7.20
7.20
7.19
7.18
7.18
7.17
7.17
3.75
3.00
2.99
2.98
2.97
2.96
2.94
2.49
1.56
1.55
1.54
1.53
1.53
1.52
1.51
1.50
1.45
1.44
1.44
1.42
1.41
1.22
1.21
0.86
0.86
0.85
O
N
H
N
N
F
iBu
(S)-9e
0.94
0.96
1.08
2.03
3.01
1.02
2.97
2.03
1.07
3.02
5.95
f1 (ppm)

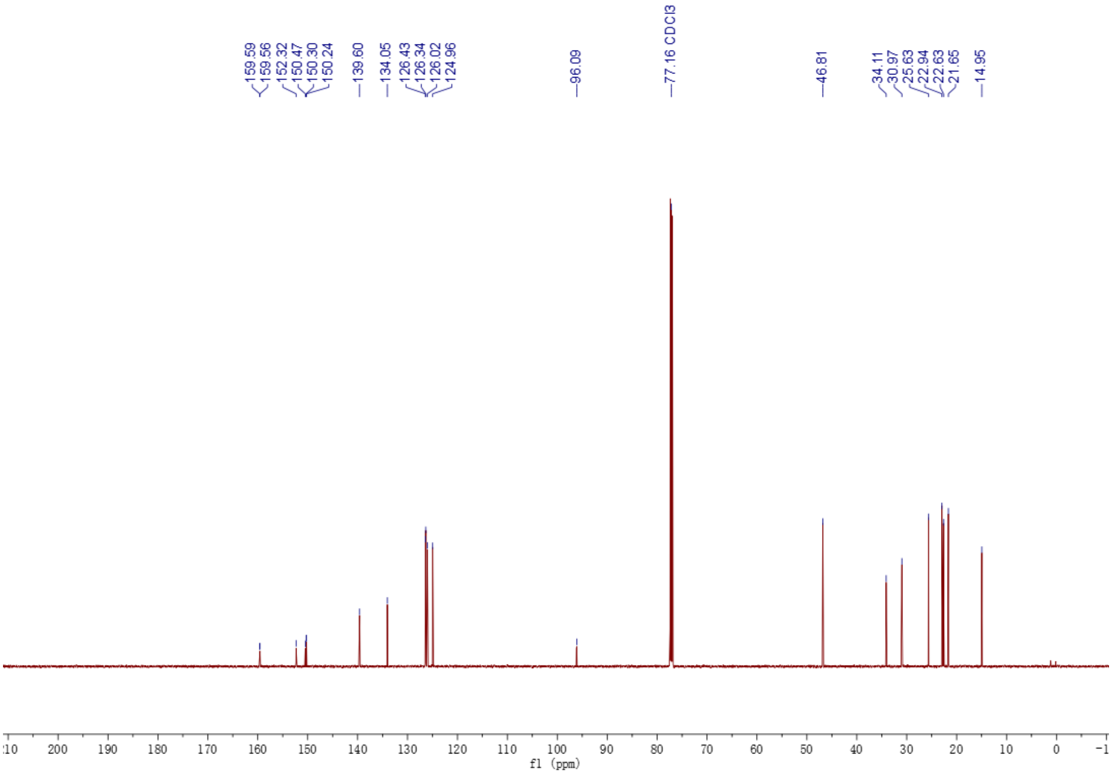
159.59
159.56
152.32
150.47
150.30
150.24
139.60
134.05
126.43
126.34
126.02
124.96
96.09
77.16 CDCl3
46.81
34.11
30.97
25.63
22.94
22.63
21.65
14.95
f1 (ppm)

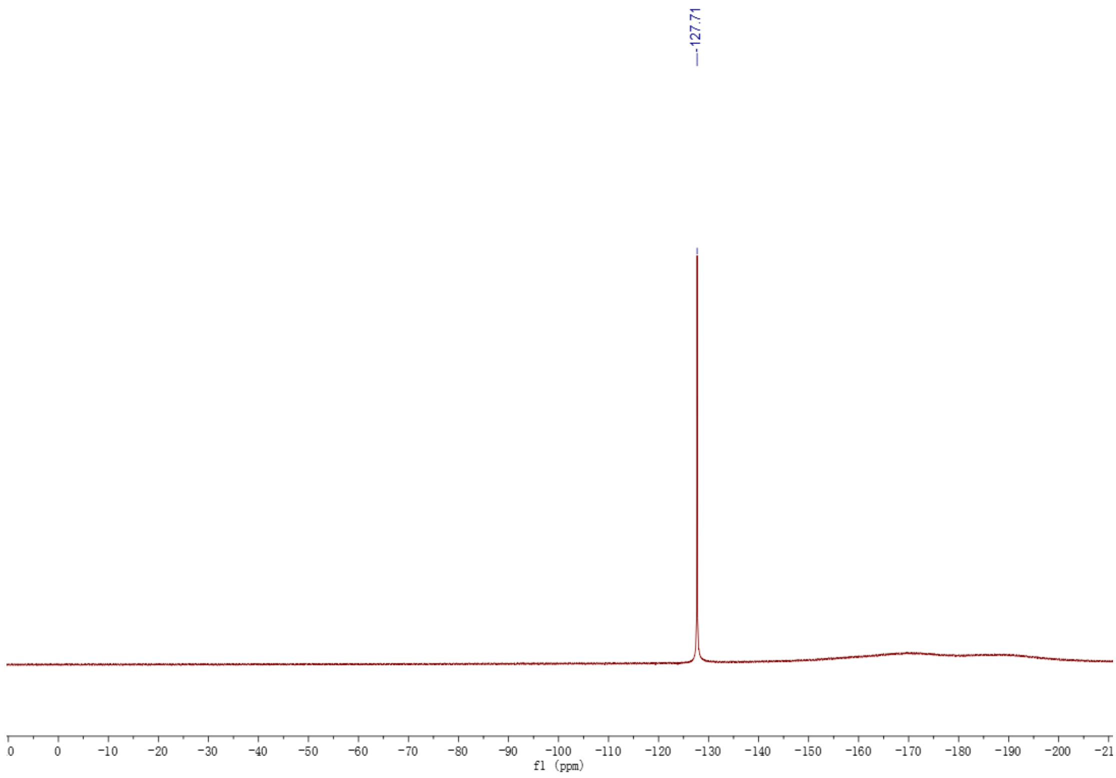
-127.71
0
0
-10
-20
-30
-40
-50
-60
-70
-80
-90
-100
-110
-120
-130
-140
-150
-160
-170
-180
-190
-200
-21
f1 (ppm)

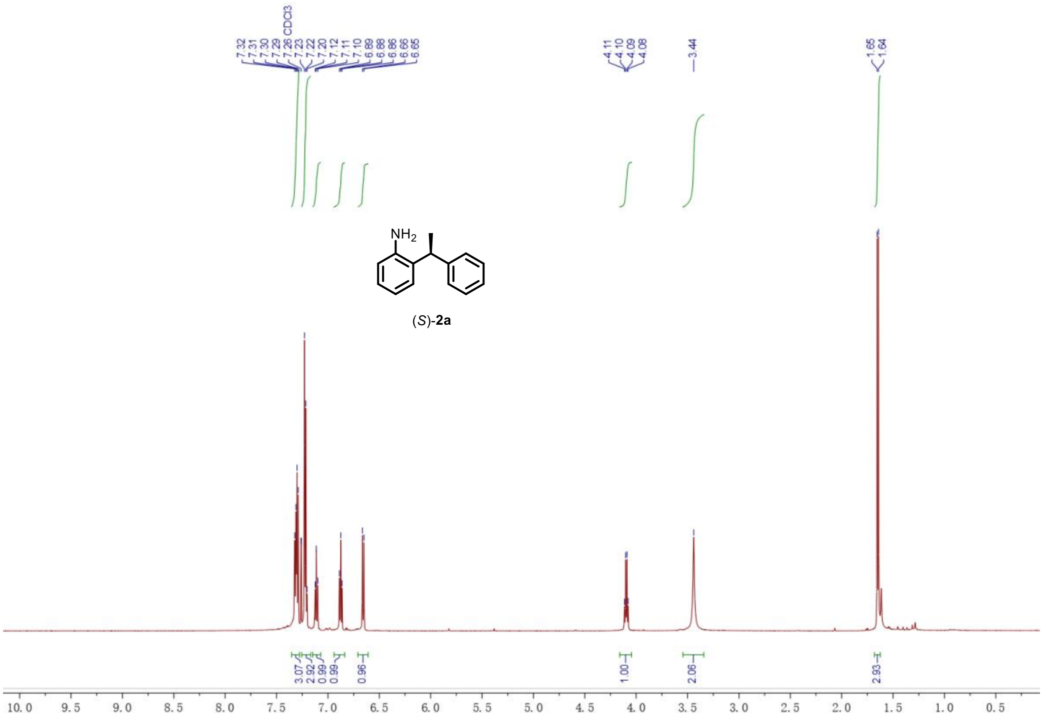
7.32
7.31
7.30
7.29
7.26 CDCl3
7.23
7.22
7.20
7.12
7.11
7.10
6.89
6.88
6.86
6.66
6.65
4.11
4.10
4.09
4.08
3.44
1.65
1.64
NH2
(S)-2a
3.07
2.92
0.99
0.99
0.96
1.00
2.06
2.93
10.0
9.5
9.0
8.5
8.0
7.5
7.0
6.5
6.0
5.5
5.0
4.5
4.0
3.5
3.0
2.5
2.0
1.5
1.0
0.5

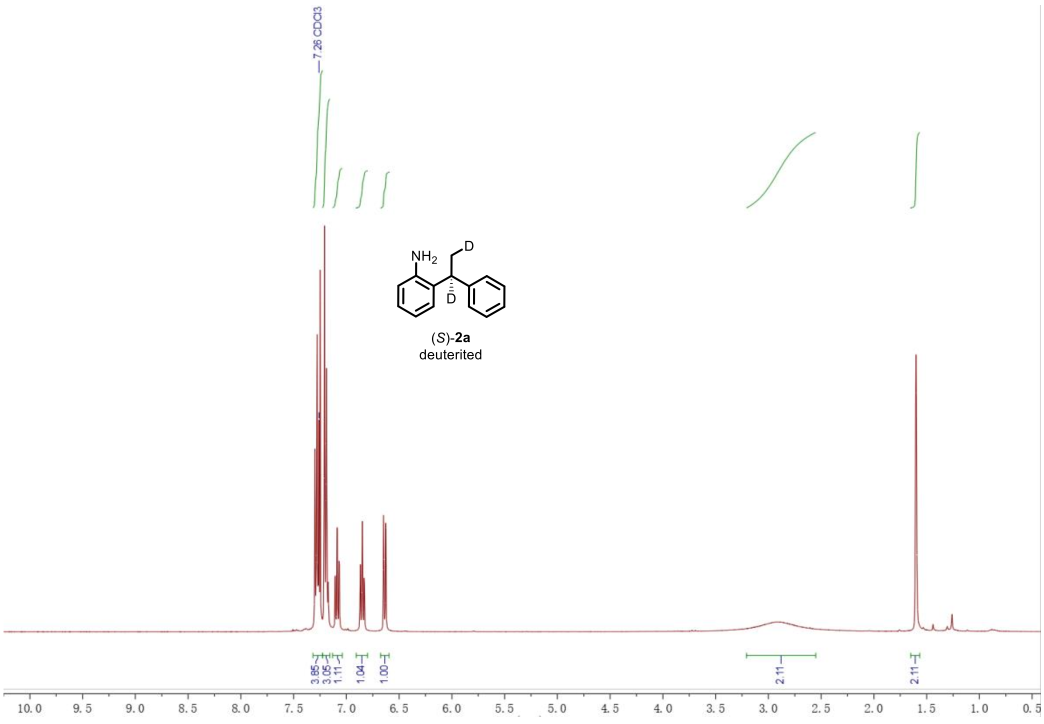
7.26 CDCl3
NH2
D
D
(S)-2a
deuterited
3.85
3.05
1.11
1.04
1.00
2.11
2.11
10.0
9.5
9.0
8.5
8.0
7.5
7.0
6.5
6.0
5.5
5.0
4.5
4.0
3.5
3.0
2.5
2.0
1.5
1.0
0.5

11. HPLC chromatograms

(*S*)-**3a**: $[\alpha]^{25}_D$ = -36.7 (c = 0.5, $CHCl_3$); 98% ee (Chiralpak AD-3 column, ipropanol/hexane = 10/90, flow rate = 1.0 mL/min, λ = 254 nm); t_S (major) = 23.7 min, t_R (minor) = 20.9 min.

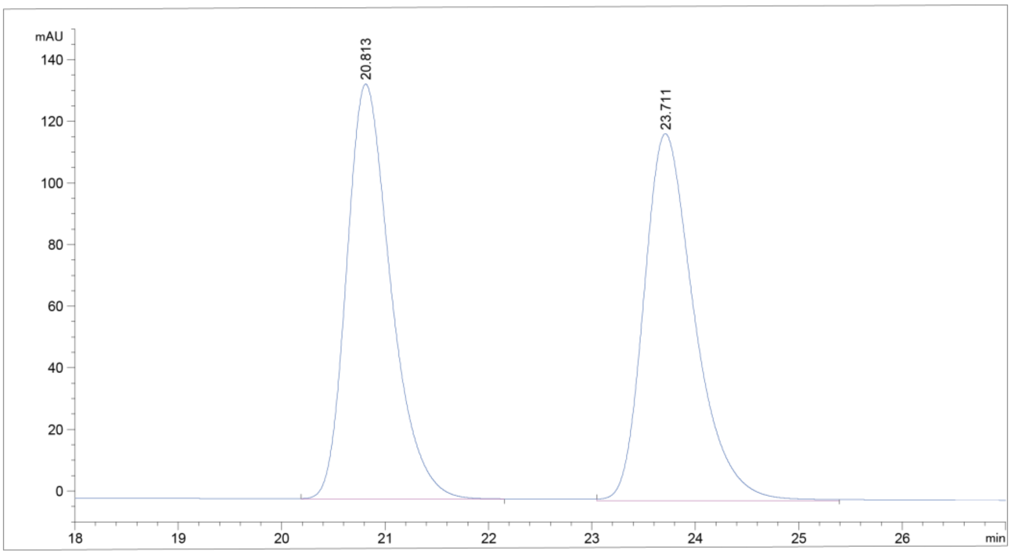

Peak #	RetTime [min]	Type	Width [min]	Area [mAU*s]	Height [mAU]	Area %
1	20.813	MM	0.4965	4014.68335	134.77484	49.7744
2	23.711	MM	0.5668	4051.08203	119.12586	50.2256

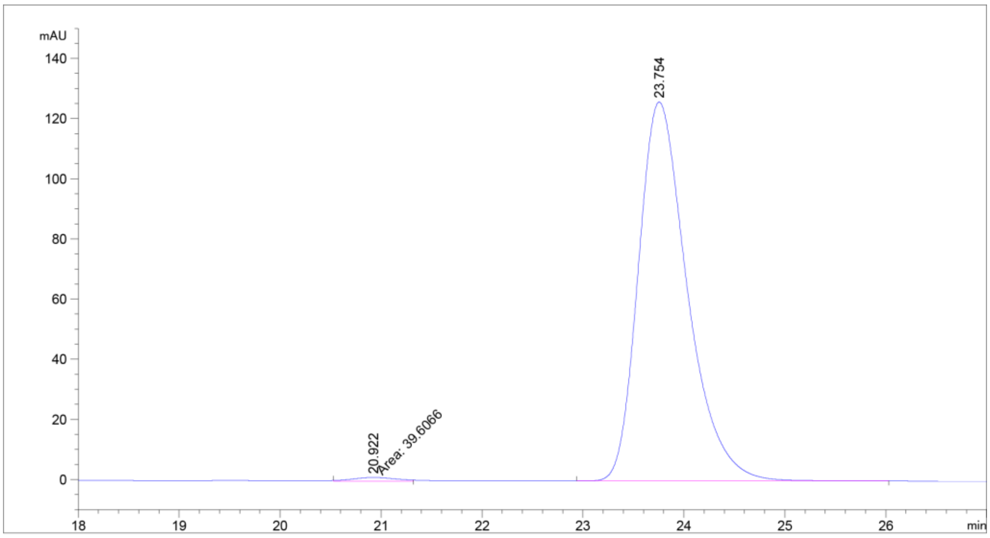

Peak #	RetTime [min]	Type	Width [min]	Area [mAU*s]	Height [mAU]	Area %
1	20.922	MM	0.5107	39.60660	1.29267	0.9255
2	23.754	BB	0.5136	4239.97607	125.98500	99.0745

(S)-**3b**: $[\alpha]^{25}_{D}$ = -162.7 (c = 0.64, $CHCl_3$); 97% ee (Chiralpak AD-3 column, ipropanol/hexane = 20/80, flow rate = 1.0 mL/min, λ = 254 nm); t_S (major) = 8.4 min, t_R (minor) = 9.1 min.

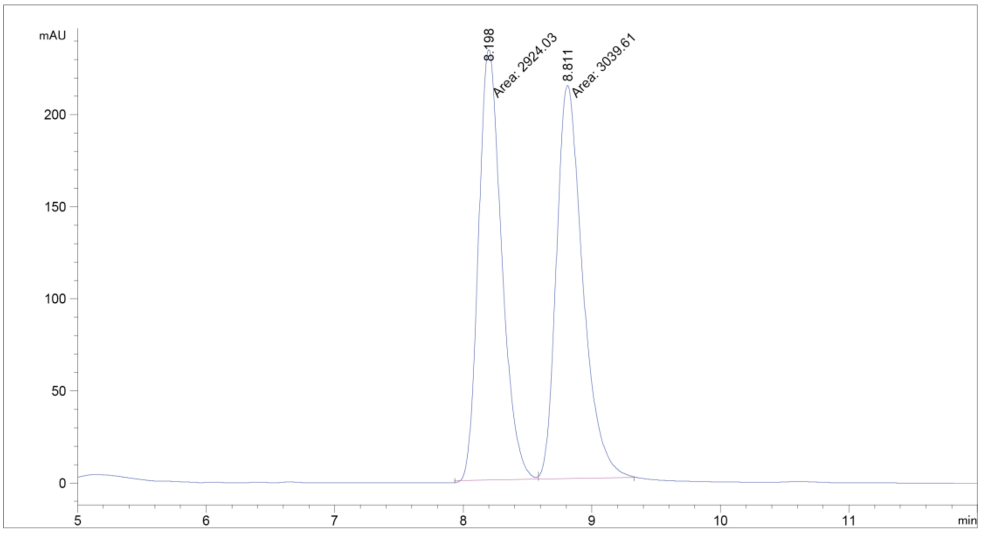

Peak #	RetTime [min]	Type	Width [min]	Area [mAU*s]	Height [mAU]	Area %
1	8.198	MF	0.2086	2924.02734	233.57990	49.0309
2	8.811	FM	0.2373	3039.61255	213.47617	50.9691

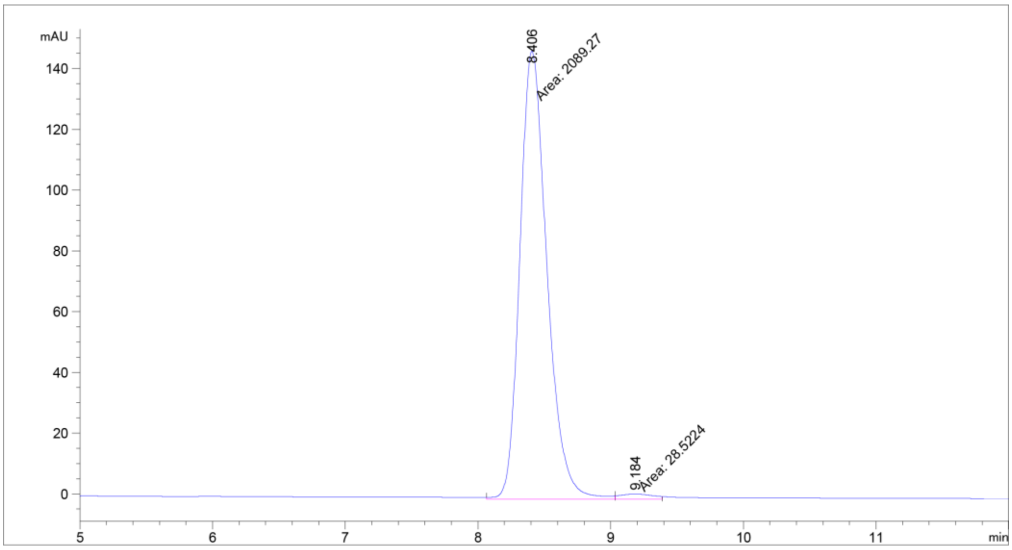

Peak #	RetTime [min]	Type	Width [min]	Area [mAU*s]	Height [mAU]	Area %
1	8.406	MF	0.2362	2089.27197	147.39900	98.6532
2	9.184	FM	0.2836	28.52237	1.67622	1.3468

(S)-**3c**: $[\alpha]^{25}_{D}$ = -54.5 (c = 0.53, $CHCl_3$); 98% ee (Chiralpak AD-3 column, ipropanol/hexane = 20/80, flow rate = 1.0 mL/min, λ = 254 nm); t_S (major) = 23.7 min, t_R (minor) = 16.0 min.

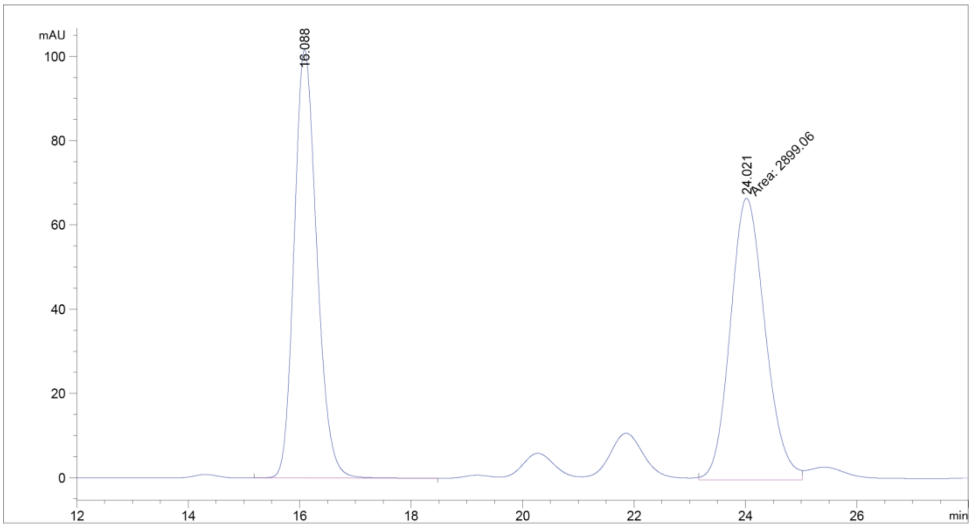

Peak #	RetTime [min]	Type	Width [min]	Area [mAU*s]	Height [mAU]	Area %
1	16.088	BB	0.4357	2875.70630	101.77021	49.7978
2	24.021	MF	0.7231	2899.05713	66.82116	50.2022

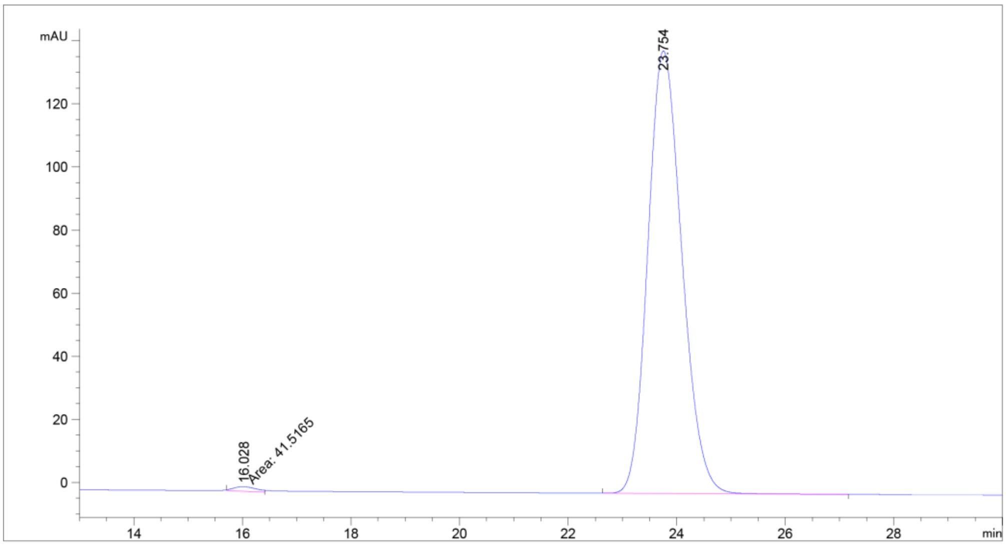

Peak #	RetTime [min]	Type	Width [min]	Area [mAU*s]	Height [mAU]	Area %
1	16.028	MM	0.4747	41.51650	1.45774	0.6747
2	23.754	BB	0.6763	6111.87793	140.27602	99.3253

(*S*)-**3d**: $[\alpha]^{25}_{D}$ = 85.6 (c = 0.45, $CHCl_3$); 98% ee (Chiralpak AD-3 column, ipropanol/hexane = 15/85, flow rate = 1.0 mL/min, λ = 254 nm); t_S (major) = 19.9 min, t_R (minor) = 12.3 min.

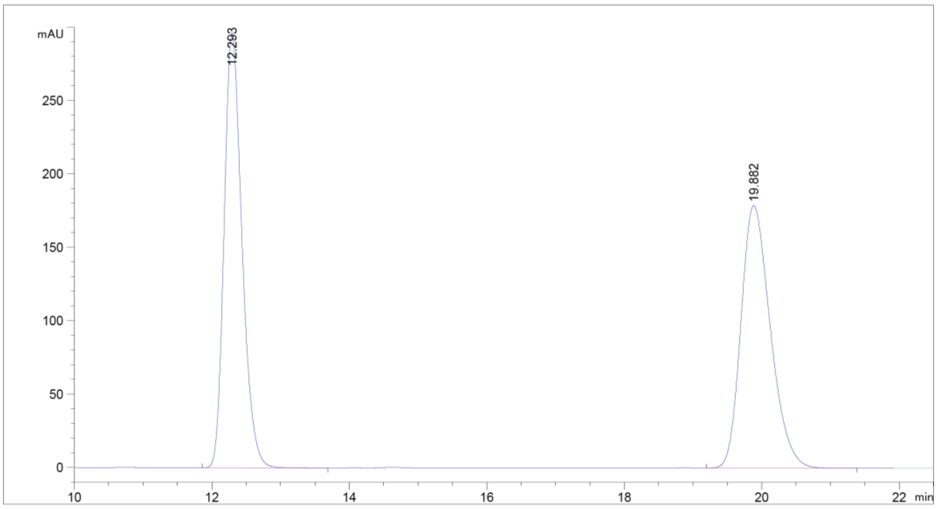

Peak #	RetTime [min]	Type	Width [min]	Area [mAU*s]	Height [mAU]	Area %
1	12.293	BB	0.2775	5352.95166	296.62482	50.2151
2	19.882	BB	0.4572	5307.09961	178.86583	49.7849

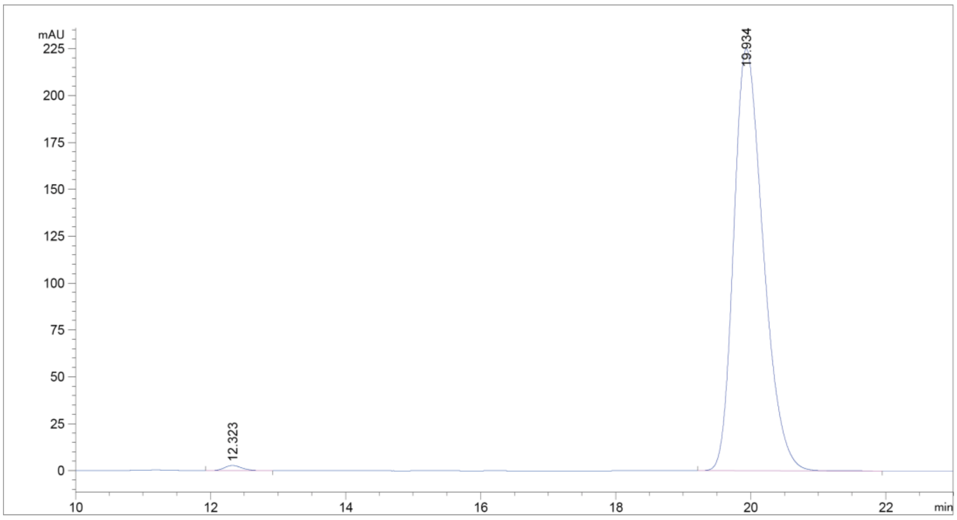

Peak #	RetTime [min]	Type	Width [min]	Area [mAU*s]	Height [mAU]	Area %
1	12.323	BB	0.2807	50.87212	2.79020	0.7410
2	19.934	BB	0.4674	6814.80518	224.97766	99.2590

(*S*)-**3e**: $[\alpha]^{25}_{D}$ = -51.5 (c = 0.37, $CHCl_3$); 98% ee (Chiralpak AD-3 column, ipropanol/hexane = 15/85, flow rate = 1.0 mL/min, λ = 254 nm); t_S (major) = 34.6 min, t_R (minor) = 20.4 min.

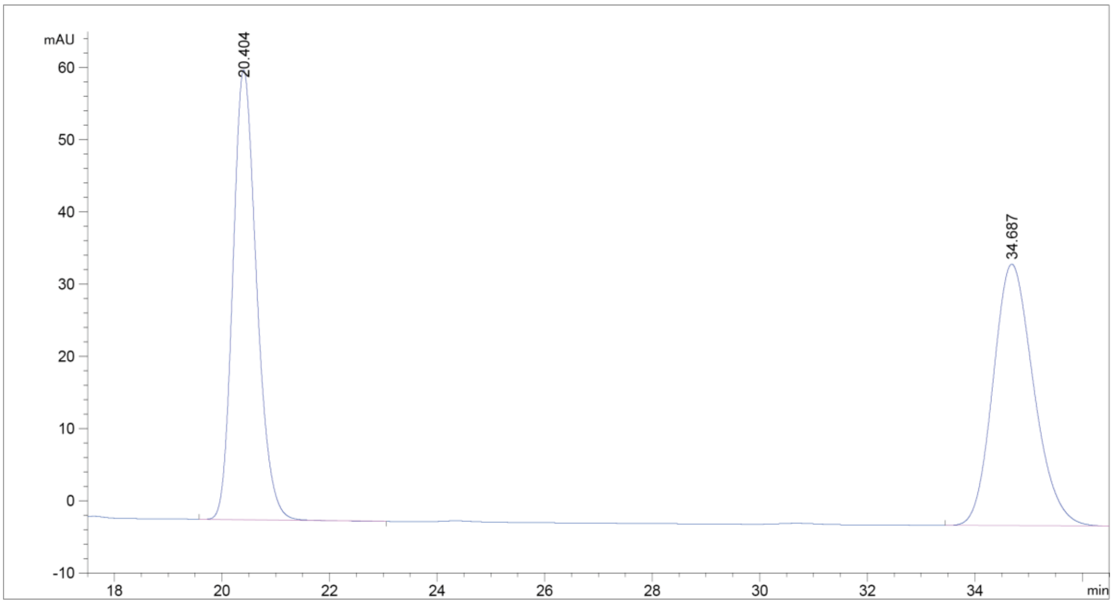

Peak #	RetTime [min]	Type	Width [min]	Area [mAU*s]	Height [mAU]	Area %
1	20.404	BB	0.4719	1913.14856	62.36538	50.1849
2	34.687	BB	0.8090	1899.05469	36.25887	49.8151

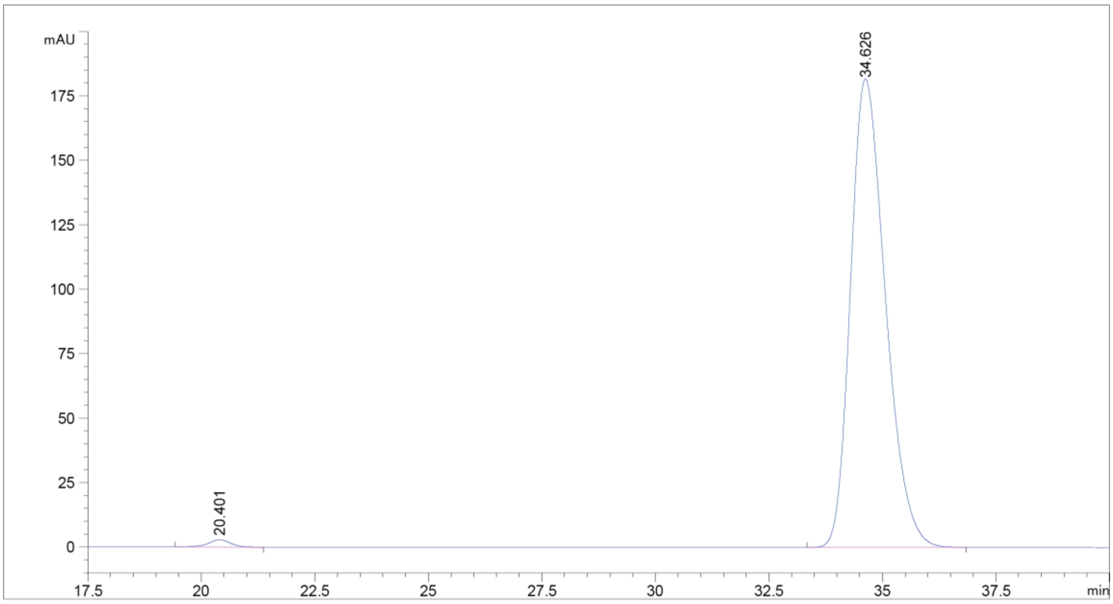

Peak #	RetTime [min]	Type	Width [min]	Area [mAU*s]	Height [mAU]	Area %
1	20.401	BB	0.5127	99.11852	2.87891	1.0109
2	34.626	BB	0.8253	9705.75391	181.68015	98.9891

(*S*)-**3f**: $[\alpha]^{25}_{D}$ = -23.9 (c = 0.43, $CHCl_3$); 98% ee (Chiralpak AD-3 column, ipropanol/hexane = 15/85, flow rate = 1.0 mL/min, λ = 254 nm); t_S (major) = 18.6 min, t_R (minor) = 17.9 min.

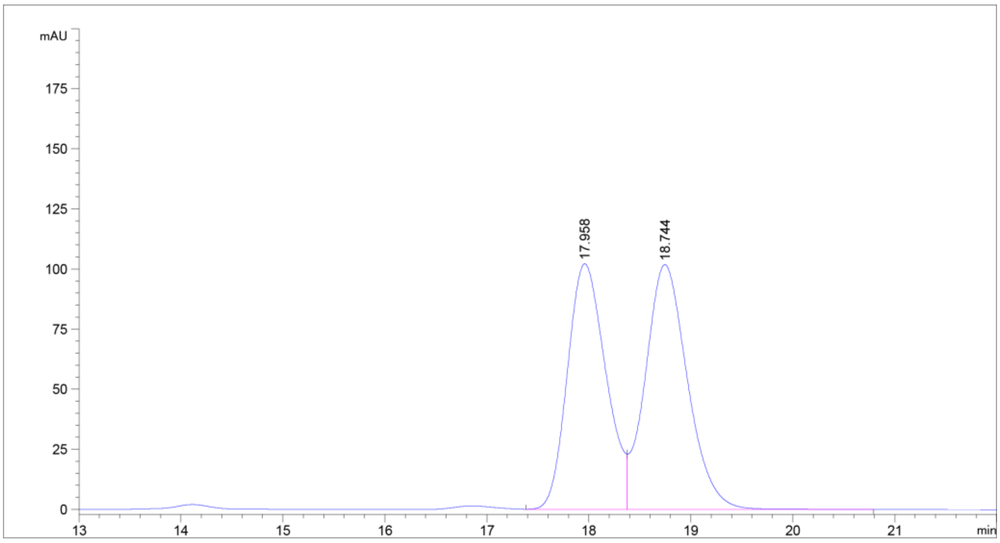

Peak #	RetTime [min]	Type	Width [min]	Area [mAU*s]	Height [mAU]	Area %
1	17.958	BV	0.4049	2677.15869	102.10434	47.7936
2	18.744	VB	0.4362	2924.33740	101.78834	52.2064

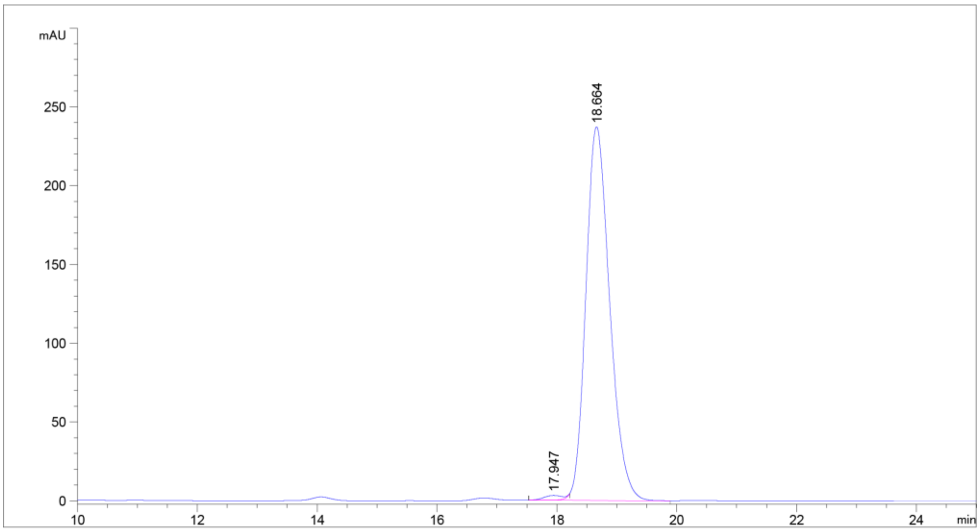

Peak #	RetTime [min]	Type	Width [min]	Area [mAU*s]	Height [mAU]	Area %
1	17.947	BV E	0.3149	60.52693	2.99287	0.9151
2	18.664	VB R	0.4265	6553.35693	237.04134	99.0849

(*S*)-**3g**: $[\alpha]^{25}_{D}$ = -76.3 (c = 0.52, CHCl3); 97% ee (Chiralpak AD-3 column, ipropanol/hexane = 15/85, flow rate = 1.0 mL/min, λ = 254 nm); t_S (major) = 27.9 min, t_R (minor) = 15.9 min.

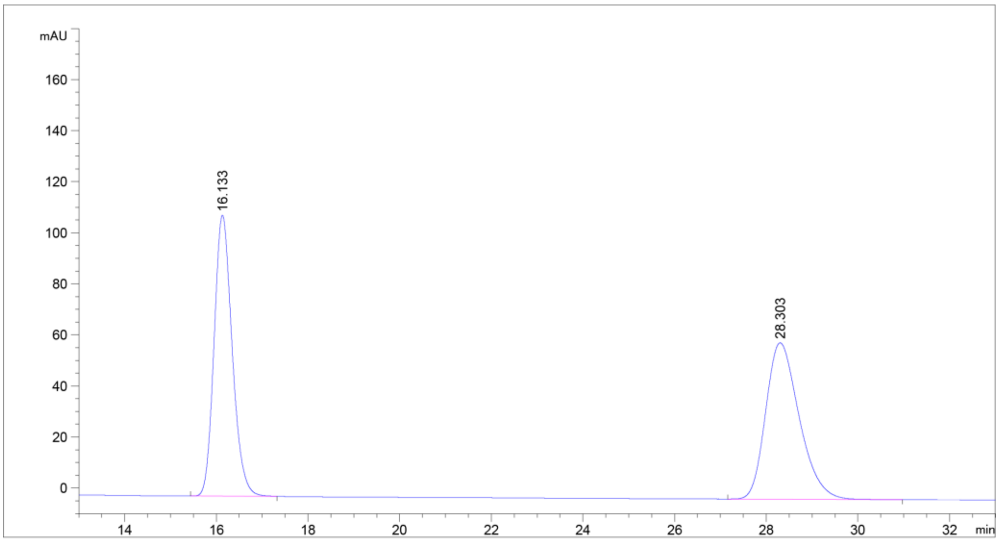

Peak #	RetTime [min]	Type	Width [min]	Area [mAU*s]	Height [mAU]	Area %
1	16.133	BB	0.4199	3008.73853	110.06474	49.2353
2	28.303	BB	0.7778	3102.19873	61.25700	50.7647

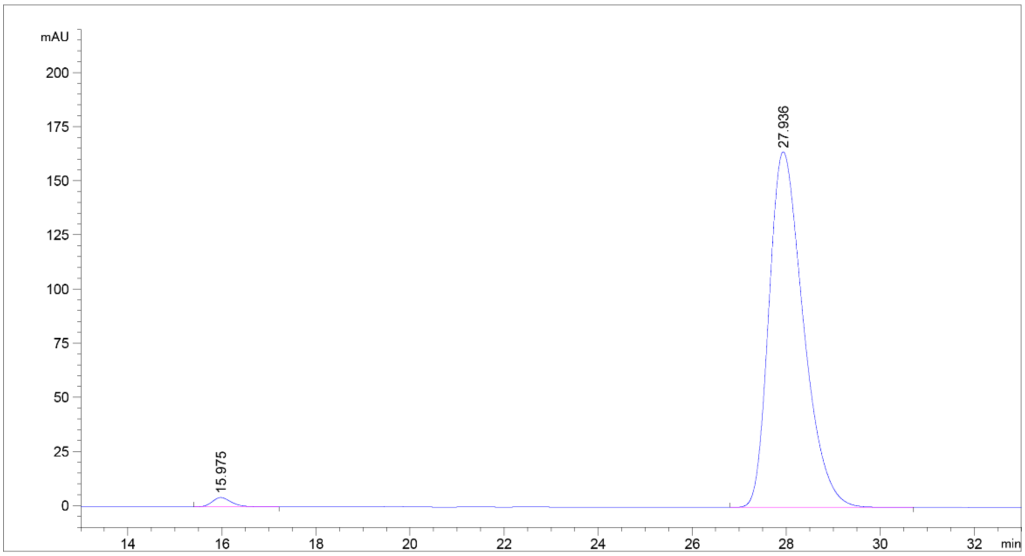

Peak #	RetTime [min]	Type	Width [min]	Area [mAU*s]	Height [mAU]	Area %
1	15.975	BB	0.4307	120.82797	4.27555	1.4324
2	27.936	BB	0.7789	8314.52539	164.16393	98.5676

(*S*)-**3h**: $[\alpha]^{25}_{D}$ = -57.1 (c = 0.40, $CHCl_3$); 99% ee (Chiralpak AD-3 column, ipropanol/hexane = 10/90, flow rate = 1.0 mL/min, λ = 254 nm); t_S (major) = 24.1 min, t_R (minor) = 20.5 min.

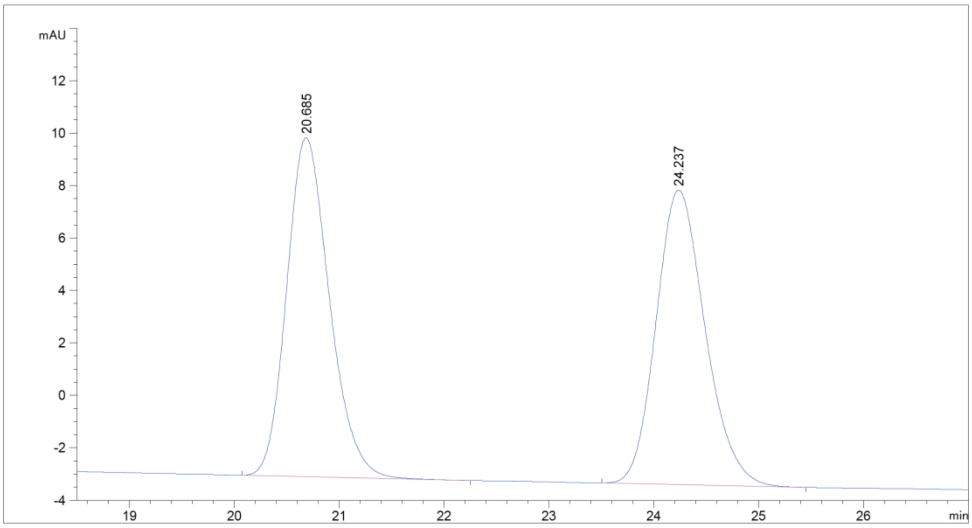

Peak #	RetTime [min]	Type	Width [min]	Area [mAU*s]	Height [mAU]	Area %
1	20.685	BB	0.4369	366.52847	12.92353	50.1146
2	24.237	BB	0.5000	364.85214	11.23235	49.8854

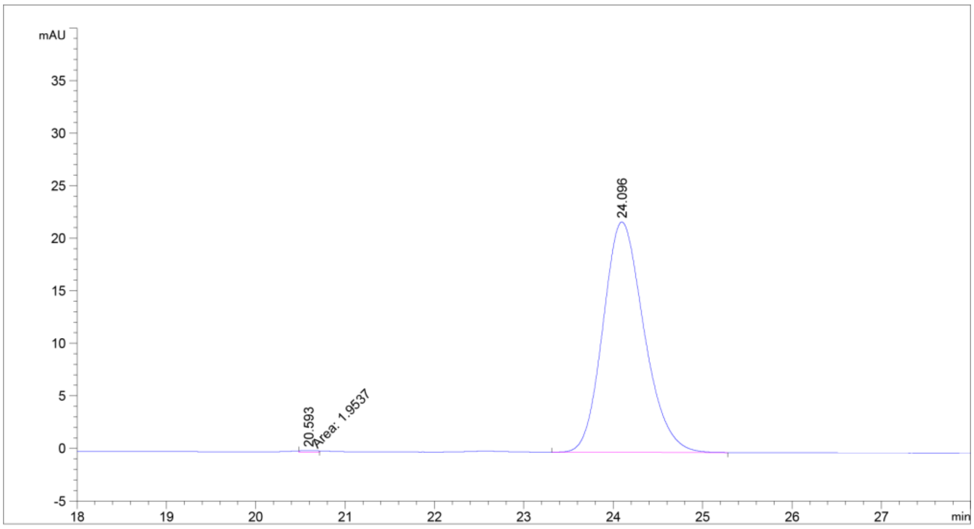

Peak #	RetTime [min]	Type	Width [min]	Area [mAU*s]	Height [mAU]	Area %
1	20.593	MM	0.2221	1.95370	1.46621e-1	0.2788
2	24.096	BB	0.4910	698.79230	21.91452	99.7212

(*S*)-**3i**: $[\alpha]^{25}_{D}$ = -93.4 (c = 0.36, $CHCl_3$); 98% ee (Chiralpak AD-3 column, ipropanol/hexane = 20/80, flow rate = 1.0 mL/min, λ = 254 nm); t_S (major) = 17.6 min, t_R (minor) = 10.9 min.

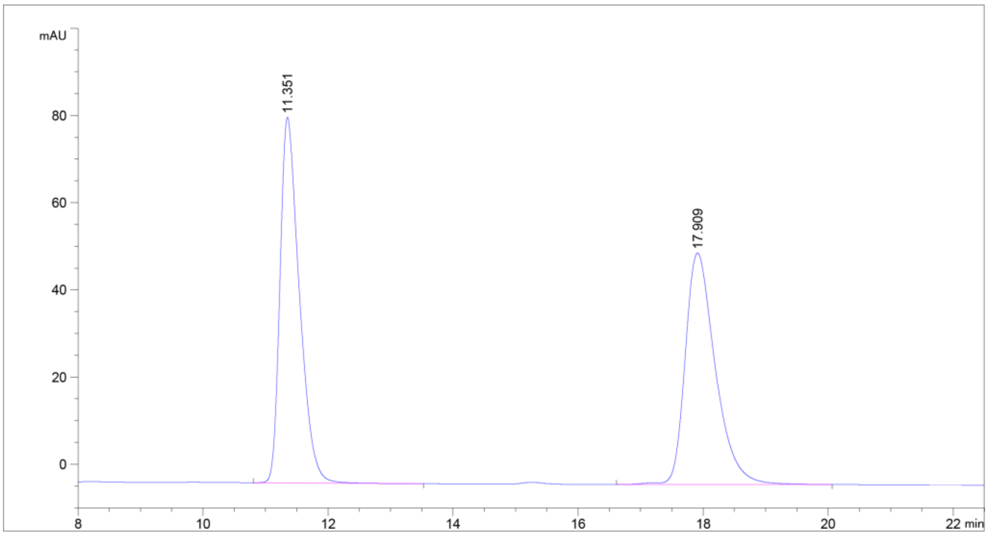

Peak #	RetTime [min]	Type	Width [min]	Area [mAU*s]	Height [mAU]	Area %
1	11.351	BB	0.3175	1800.59485	83.87106	50.2956
2	17.909	BB	0.5098	1779.43079	53.11843	49.7044

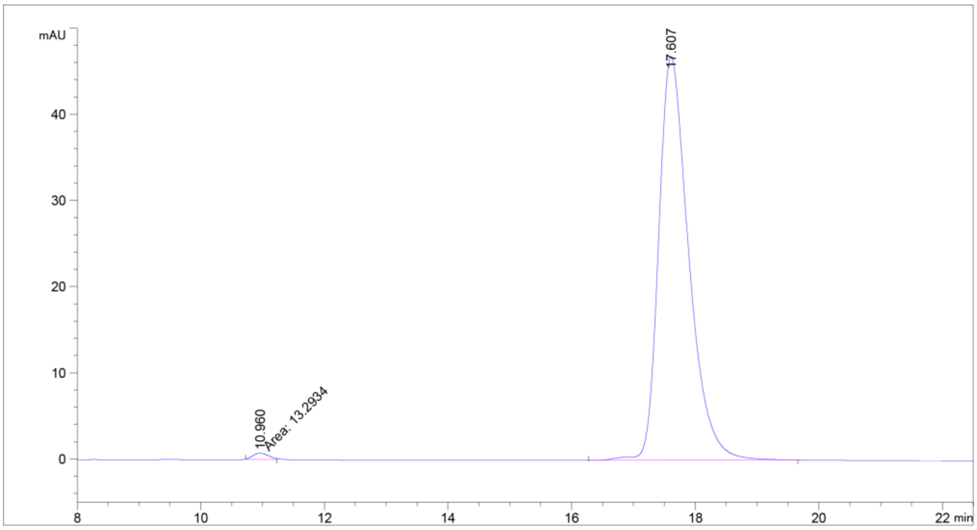

Peak #	RetTime [min]	Type	Width [min]	Area [mAU*s]	Height [mAU]	Area %
1	10.960	MM	0.2957	13.29343	7.49197e-1	0.8314
2	17.607	BB	0.5141	1585.57703	46.81987	99.1686

(S)-**3j**: $[\alpha]^{25}_{D}$ = -54.8 (c = 0.56, $CHCl_3$); 98% ee (Chiralpak AD-3 column, ipropanol/hexane = 15/85, flow rate = 1.0 mL/min, λ = 254 nm); t_S (major) = 11.4 min, t_R (minor) = 10.8 min.

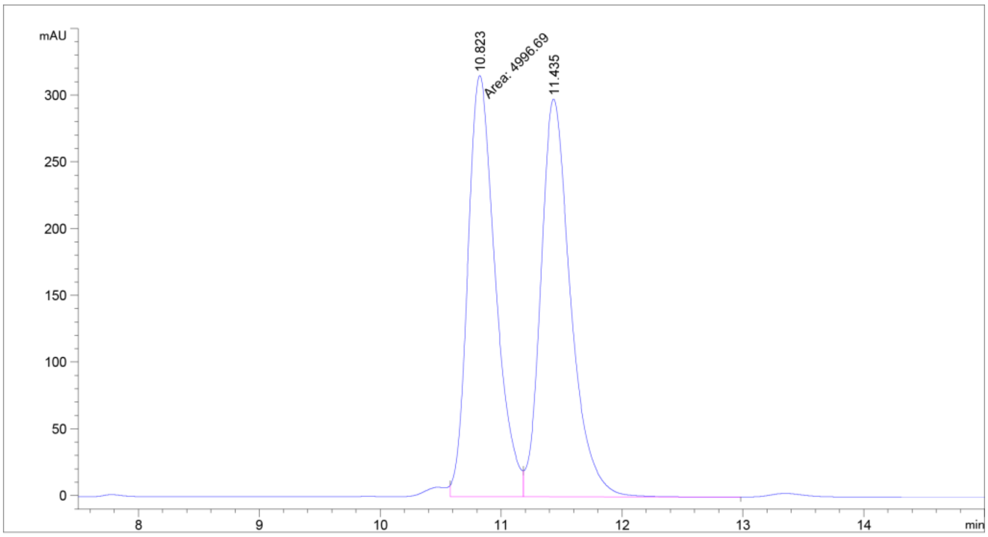

Peak #	RetTime [min]	Type	Width [min]	Area [mAU*s]	Height [mAU]	Area %
1	10.823	FM	0.2639	4996.68896	315.52139	49.4520
2	11.435	VB	0.2590	5107.42725	297.93396	50.5480

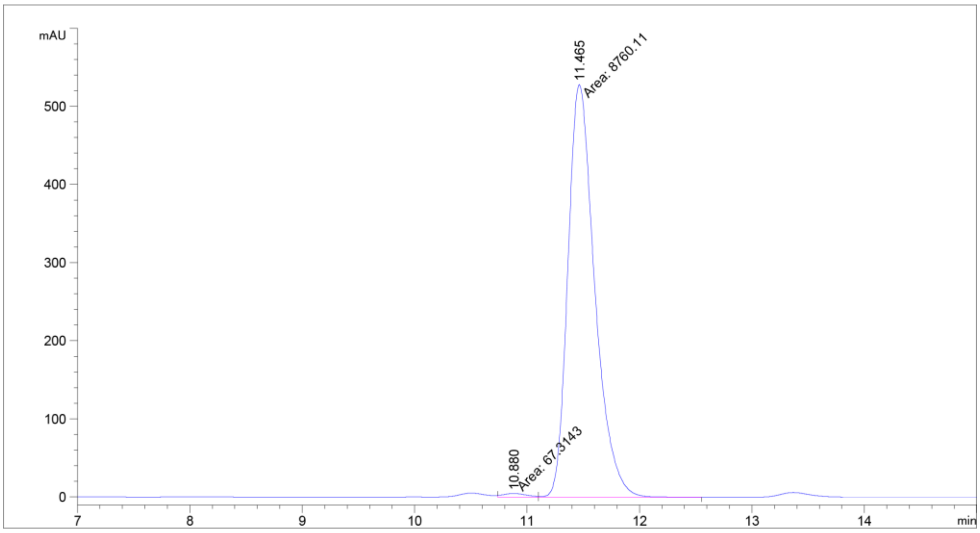

Peak #	RetTime [min]	Type	Width [min]	Area [mAU*s]	Height [mAU]	Area %
1	10.880	MF	0.2402	67.31434	4.67031	0.7626
2	11.465	FM	0.2766	8760.10547	527.82635	99.2374

(S)-**3k**: $[\alpha]^{25}_{D}$ = -26.1 (c = 0.34, $CHCl_3$); 98% ee (Chiralpak AD-3 column, ipropanol/hexane = 10/90, flow rate = 1.0 mL/min, λ = 254 nm); t_S (major) = 31.2 min, t_R (minor) = 22.7 min.

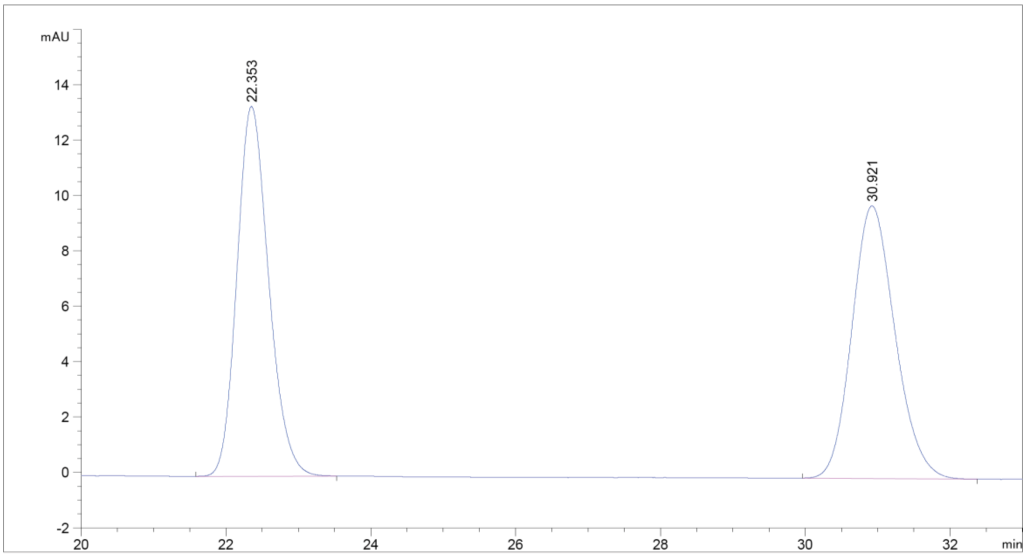

Peak #	RetTime [min]	Type	Width [min]	Area [mAU*s]	Height [mAU]	Area %
1	22.353	BB	0.4689	407.30624	13.35223	50.0096
2	30.921	BB	0.6337	407.14975	9.84145	49.9904

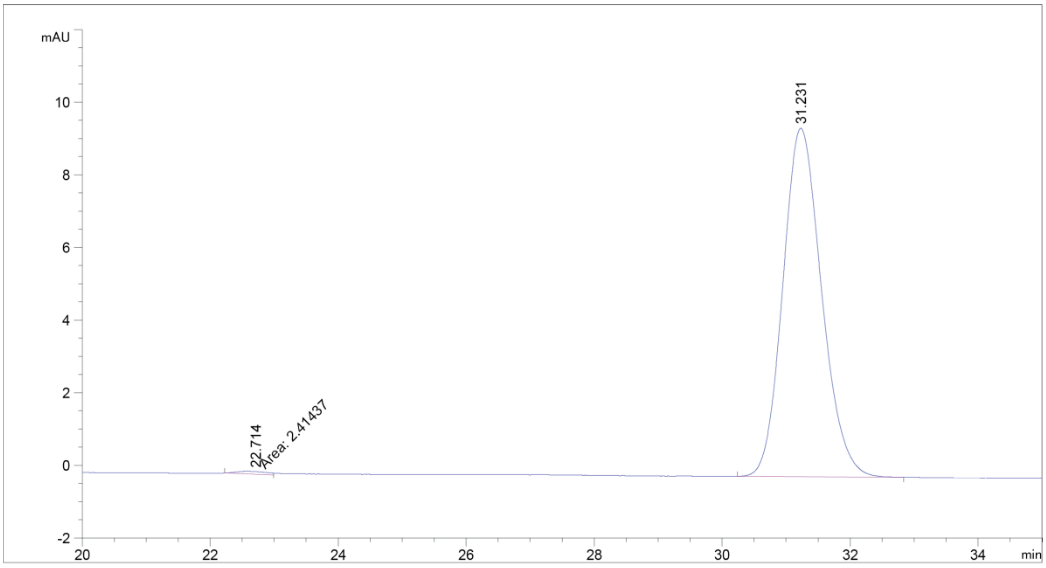

Peak #	RetTime [min]	Type	Width [min]	Area [mAU*s]	Height [mAU]	Area %
1	22.714	MM	0.5362	2.41437	7.50479e-2	0.5951
2	31.231	BB	0.6394	403.29242	9.59426	99.4049

(S)-**3l**: $[\alpha]^{25}_{D}$ = -46.0 (c = 0.28, $CHCl_3$); 97% ee (Chiralpak AD-3 column, ipropanol/hexane = 15/85, flow rate = 1.0 mL/min, λ = 254 nm); t_S (major) = 21.1 min, t_R (minor) = 13.3 min.

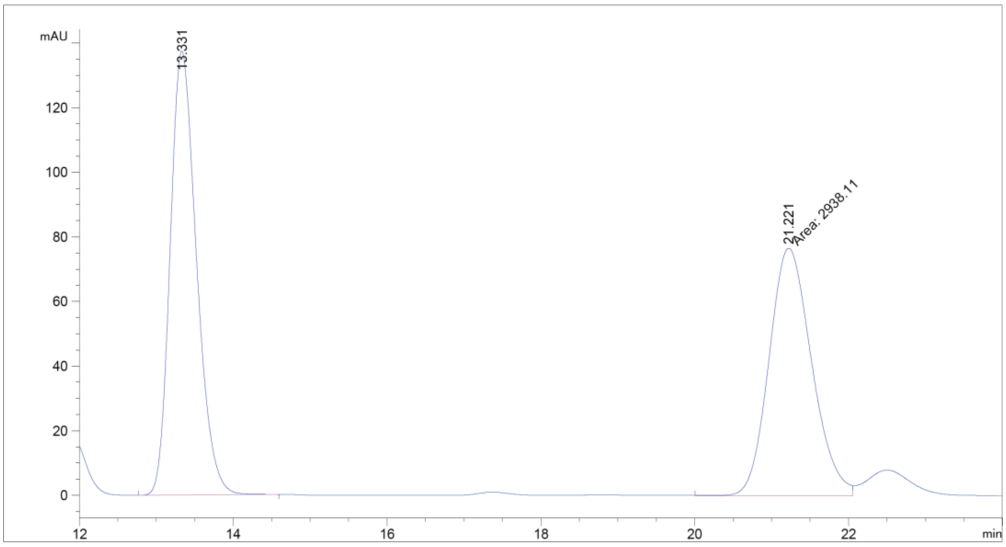

Peak #	RetTime [min]	Type	Width [min]	Area [mAU*s]	Height [mAU]	Area %
1	13.331	BB	0.3668	3271.64551	137.41414	52.6856
2	21.221	MF	0.6398	2938.10547	76.54034	47.3144

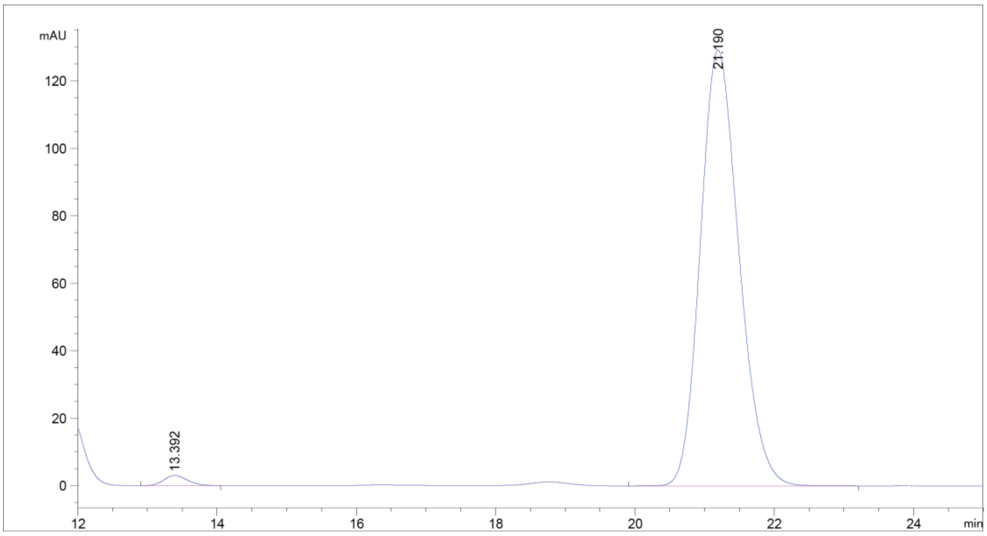

Peak #	RetTime [min]	Type	Width [min]	Area [mAU*s]	Height [mAU]	Area %
1	13.392	BB	0.3631	71.57476	3.02457	1.4169
2	21.190	BB	0.5977	4979.90576	129.19354	98.5831

(*S*)-**3m**: $[\alpha]^{25}_{D}$ = -109.3 (c = 0.44, $CHCl_3$); 98% ee (Chiralpak AD-3 column, ipropanol/hexane = 15/85, flow rate = 1.0 mL/min, λ = 254 nm); t_S (major) = 35.6 min, t_R (minor) = 30.6 min.

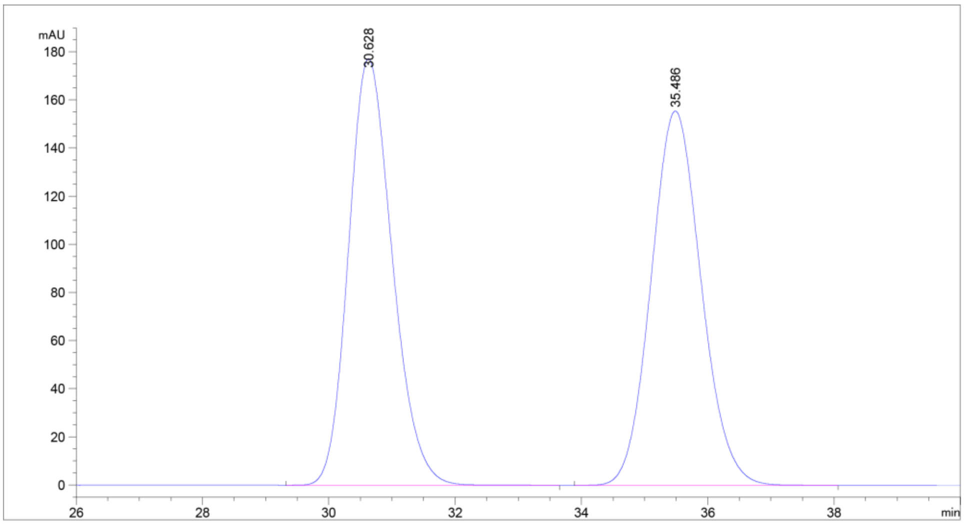

Peak #	RetTime [min]	Type	Width [min]	Area [mAU*s]	Height [mAU]	Area %
1	30.628	BB	0.7435	8529.90234	177.24715	49.8647
2	35.486	BB	0.8552	8576.19238	155.55103	50.1353

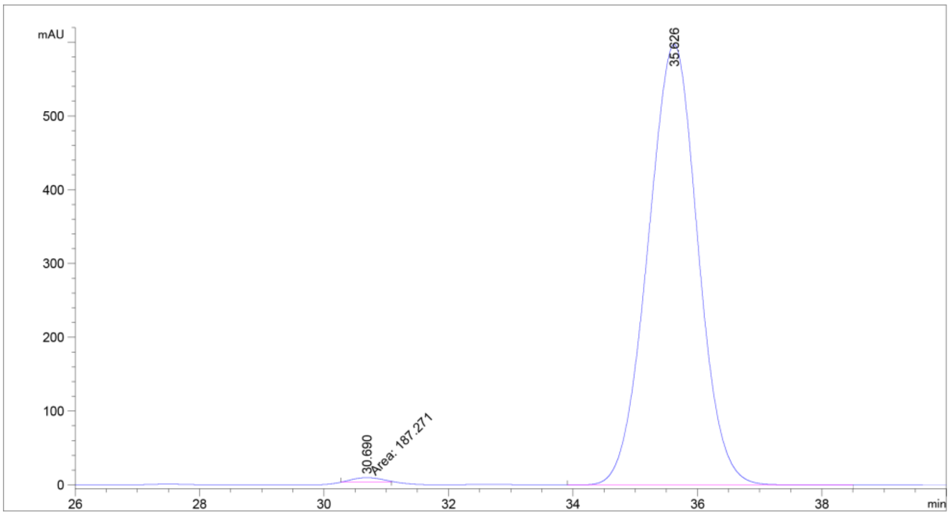

Peak #	RetTime [min]	Type	Width [min]	Area [mAU*s]	Height [mAU]	Area %
1	30.690	MM	0.5176	187.27101	6.03051	0.5602
2	35.626	BB	0.8646	3.32433e4	597.01233	99.4398

(*S*)-**3n**: $[\alpha]^{25}_{D}$ = -51.1 (c = 0.58, $CHCl_3$); 97% ee (Chiralpak AD-3 column, ipropanol/hexane = 20/80, flow rate = 1.0 mL/min, λ = 254 nm); t_S (major) = 10.1 min, t_R (minor) = 9.1 min.

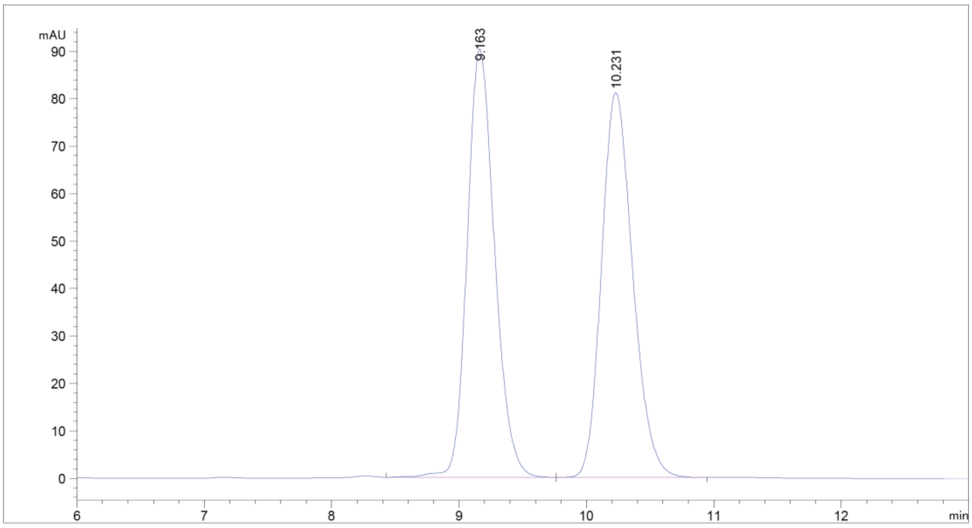

Peak #	RetTime [min]	Type	Width [min]	Area [mAU*s]	Height [mAU]	Area %
1	9.163	BB	0.2310	1352.84473	90.20933	49.5150
2	10.231	BB	0.2635	1379.34827	81.06705	50.4850

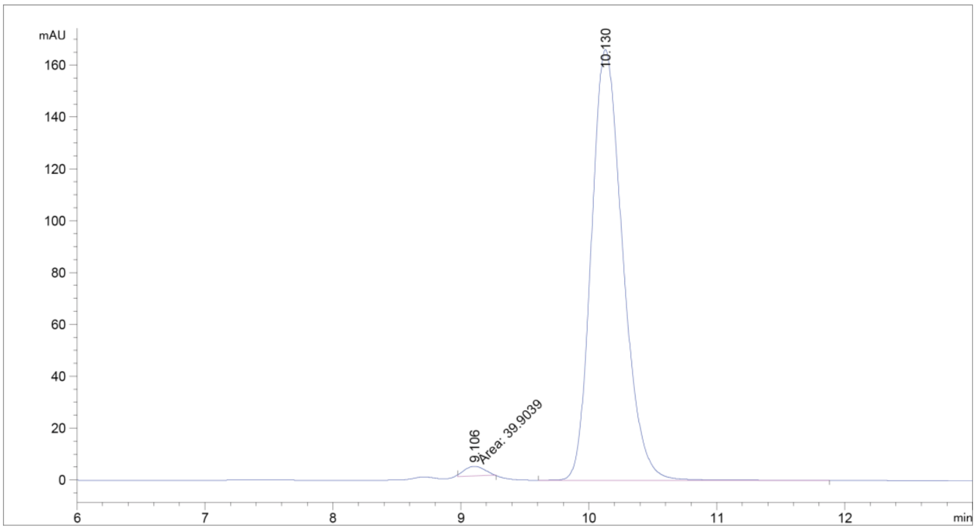

Peak #	RetTime [min]	Type	Width [min]	Area [mAU*s]	Height [mAU]	Area %
1	9.106	MM	0.1789	39.90387	3.71726	1.3889
2	10.130	BB	0.2630	2833.13428	166.08717	98.6111

(*S*)-**3o**: $[\alpha]^{25}_{D}$ = 94.5 (c = 0.7, $CHCl_3$); 99% ee (Chiralcel OD-3 column, ipropanol/hexane = 20/80, flow rate = 1.0 mL/min, λ = 254 nm); t_S (major) = 16.1 min, t_R (minor) = 20.3 min.

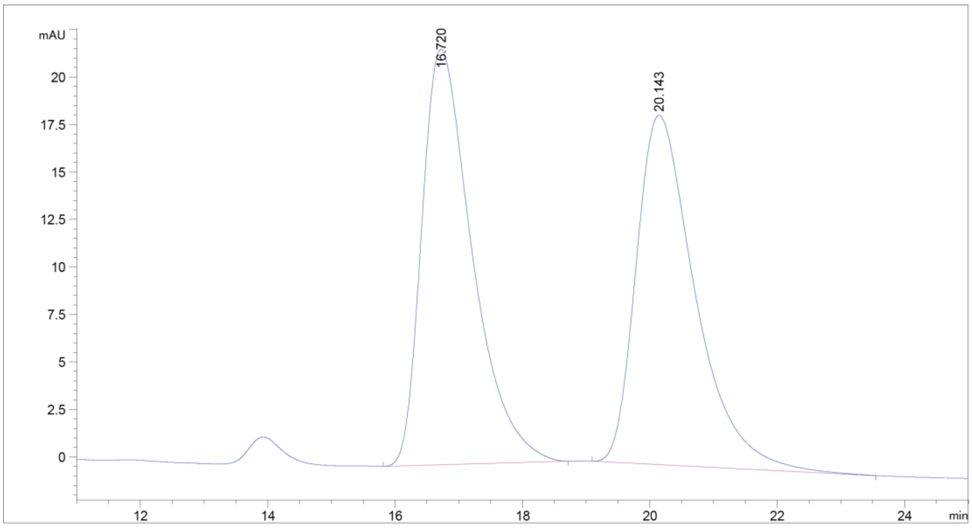

Peak #	RetTime [min]	Type	Width [min]	Area [mAU*s]	Height [mAU]	Area %
1	16.720	BB	0.8346	1203.06641	21.87730	50.4628
2	20.143	BB	0.9532	1181.00037	18.38982	49.5372

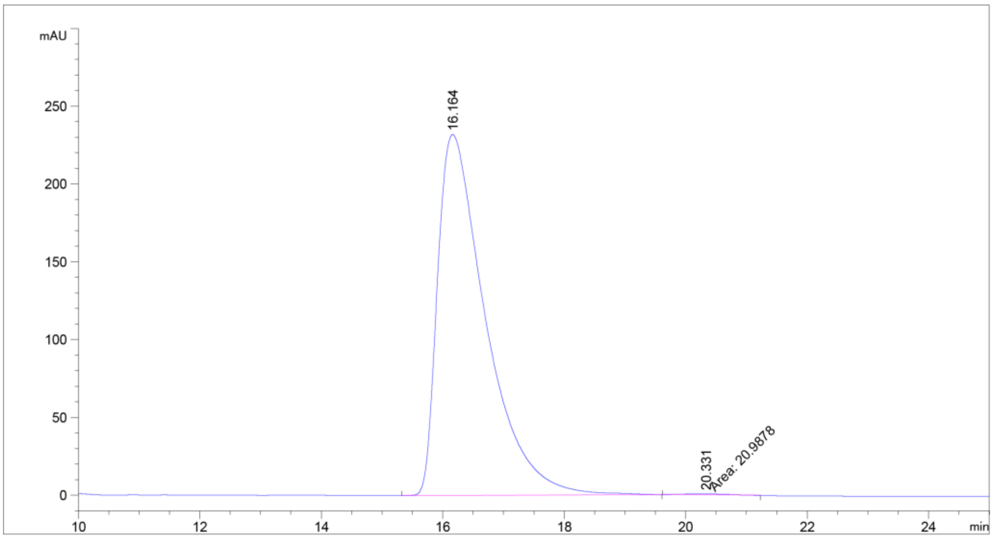

Peak #	RetTime [min]	Type	Width [min]	Area [mAU*s]	Height [mAU]	Area %
1	16.164	BB	0.8230	1.27264e4	231.98557	99.8354
2	20.331	MM	0.6657	20.98778	5.25445e-1	0.1646

(*S*)-**3p**: $[\alpha]^{25}_{D}$ = -40.7 (c = 0.51, $CHCl_3$); 97% ee (Chiralpak AD-3 column, ipropanol/hexane = 15/85, flow rate = 1.0 mL/min, λ = 254 nm); t_S (major) = 13.4 min, t_R (minor) = 16.1 min.

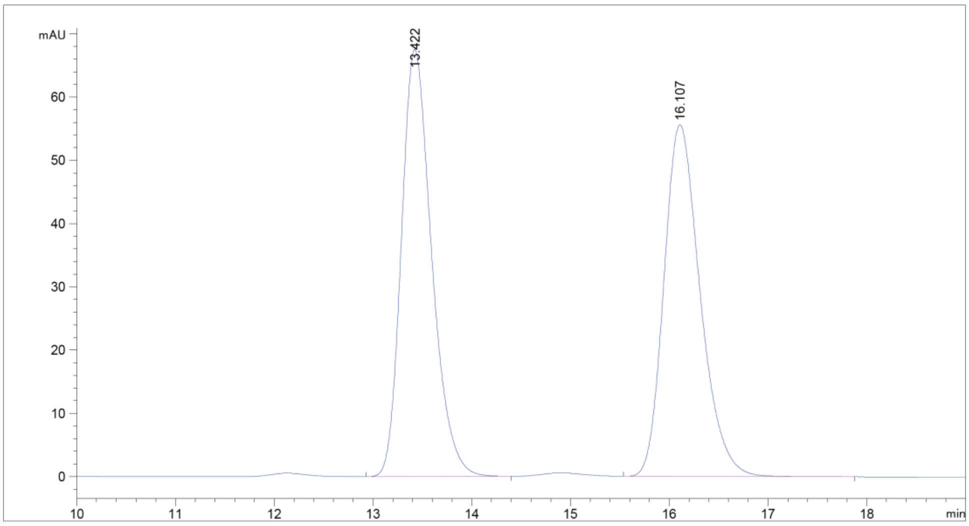

Peak #	RetTime [min]	Type	Width [min]	Area [mAU*s]	Height [mAU]	Area %
1	13.422	BB	0.3263	1437.07397	67.52413	50.3703
2	16.107	BB	0.3901	1415.94690	55.60960	49.6297

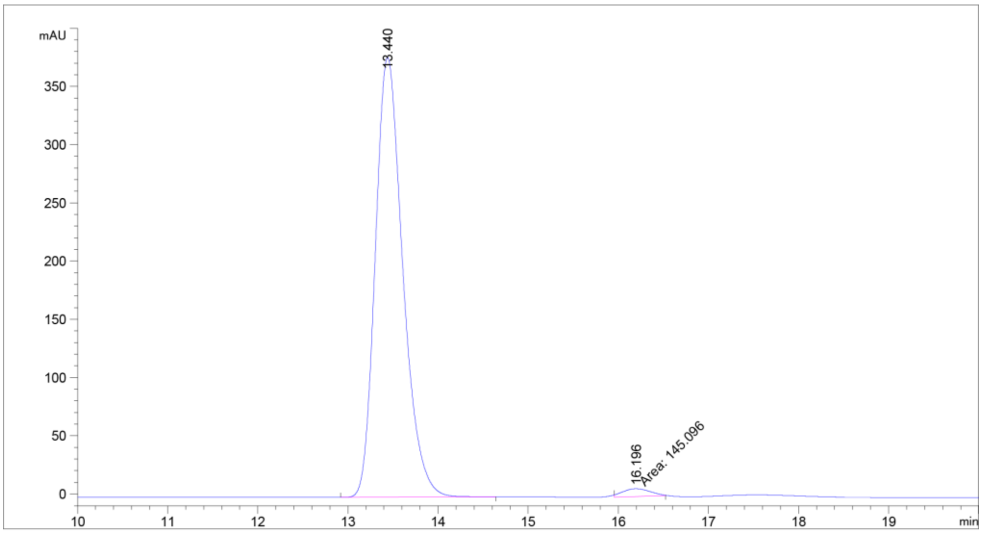

Peak #	RetTime [min]	Type	Width [min]	Area [mAU*s]	Height [mAU]	Area %
1	13.440	BB	0.3251	7996.59912	377.54703	98.2179
2	16.196	MM	0.3558	145.09550	6.79646	1.7821

(*S*)-**3q**: $[\alpha]^{25}_{D}$ = 39.8 (c = 0.5, $CHCl_3$); 93% ee (Chiralpak AD-3 column, ipropanol/hexane = 20/80, flow rate = 1.0 mL/min, λ = 254 nm); t_S (major) = 8.4 min, t_R (minor) = 9.8 min.

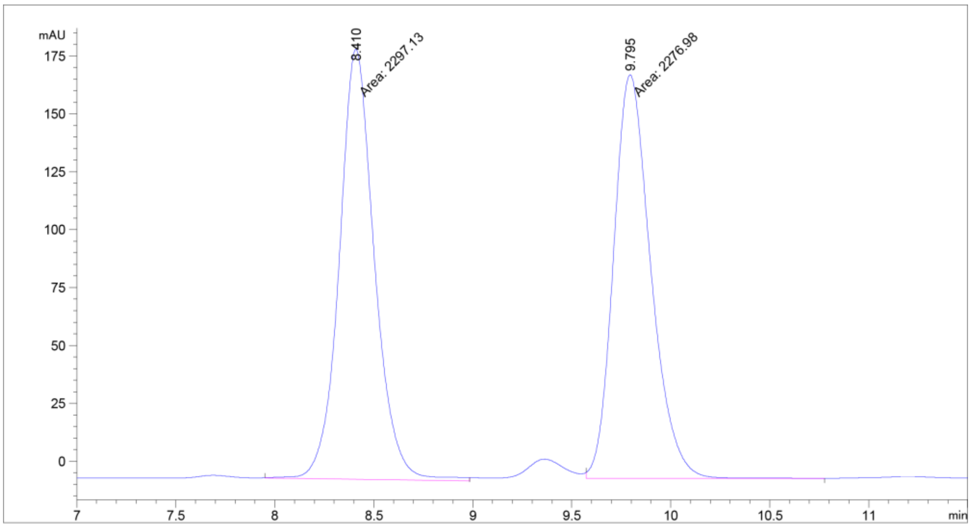

Peak #	RetTime [min]	Type	Width [min]	Area [mAU*s]	Height [mAU]	Area %
1	8.410	MM	0.2062	2297.12695	185.69632	50.2202
2	9.795	FM	0.2178	2276.97803	174.22192	49.7798

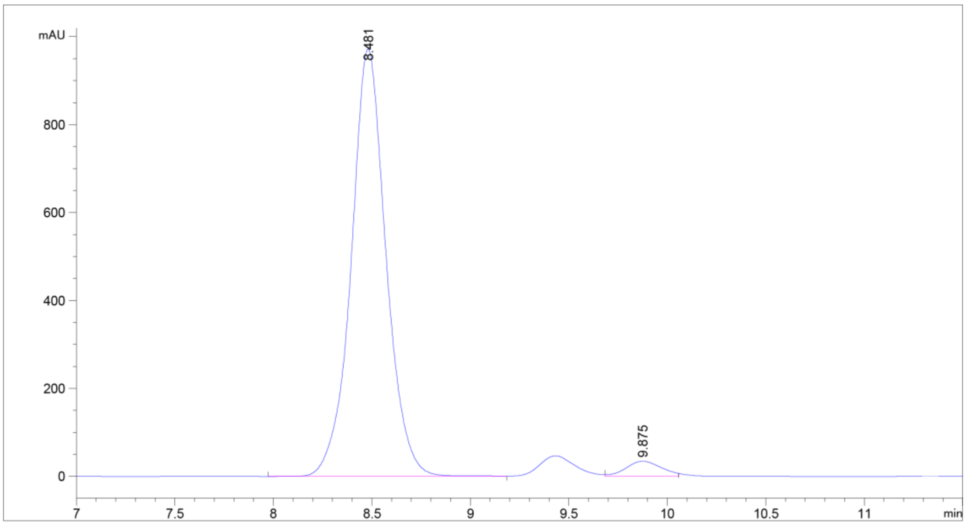

Peak #	RetTime [min]	Type	Width [min]	Area [mAU*s]	Height [mAU]	Area %
1	8.481	BV	0.1832	1.19275e4	971.88739	96.5171
2	9.875	MF	0.2083	430.41763	34.44402	3.4829

(*S*)-**3r**: $[\alpha]^{25}_{D}$ = -63.2 (c = 0.44, $CHCl_3$); 97% ee (Chiralpak AD-3 column, ipropanol/hexane = 20/80, flow rate = 1.0 mL/min, λ = 254 nm); t_S (major) = 23.8 min, t_R (minor) = 14.1 min.

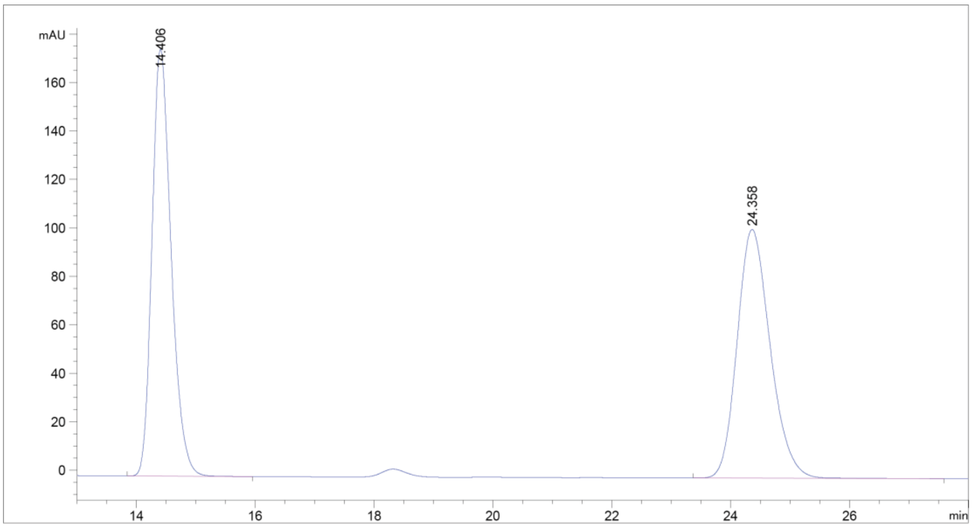

Peak #	RetTime [min]	Type	Width [min]	Area [mAU*s]	Height [mAU]	Area %
1	14.406	BB	0.3445	3947.68677	176.10376	49.9273
2	24.358	BBA	0.5953	3959.19043	102.57844	50.0727

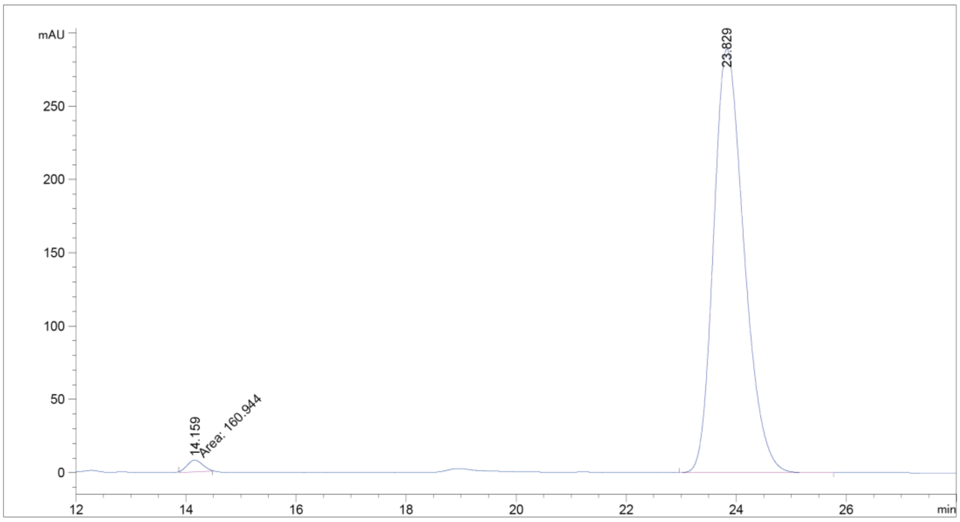

Peak #	RetTime [min]	Type	Width [min]	Area [mAU*s]	Height [mAU]	Area %
1	14.159	MM	0.3321	160.94429	8.07827	1.4322
2	23.829	BB	0.5904	1.10769e4	288.84744	98.5678

(*S*)-**3s**: $[\alpha]^{25}_{D}$ = -19.6 (c = 0.42, $CHCl_3$); 17% ee (Chiralpak OD-3 column, ipropanol/hexane = 20/80, flow rate = 1.0 mL/min, λ = 254 nm); t_S (major) = 13.4 min, t_R (minor) = 16.1 min.

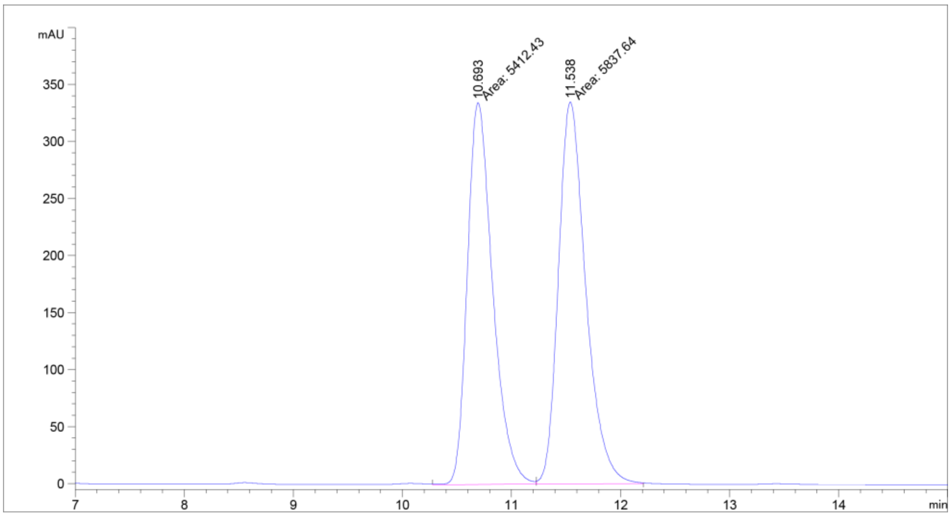

Peak #	RetTime [min]	Type	Width [min]	Area [mAU*s]	Height [mAU]	Area %
1	10.693	MF	0.2694	5412.42676	334.86349	48.1102
2	11.538	FM	0.2905	5837.64355	334.88184	51.8898

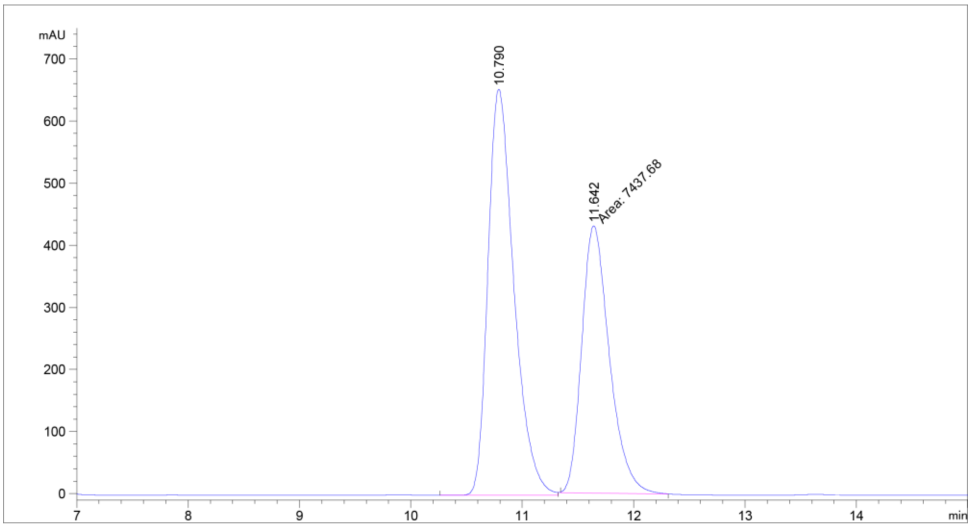

Peak #	RetTime [min]	Type	Width [min]	Area [mAU*s]	Height [mAU]	Area %
1	10.790	BV	0.2470	1.05911e4	653.61652	58.7454
2	11.642	MM	0.2881	7437.68115	430.31018	41.2546

(S)-**4a**: $[\alpha]^{25}_{D}$ = -0.6 (c = 0.59, $CHCl_3$); 98% ee (Chiralpak AD-3 column, ipropanol/hexane = 20/80, flow rate = 1.0 mL/min, λ = 254 nm); t_S (major) = 13.0 min, t_R (minor) = 17.3 min.

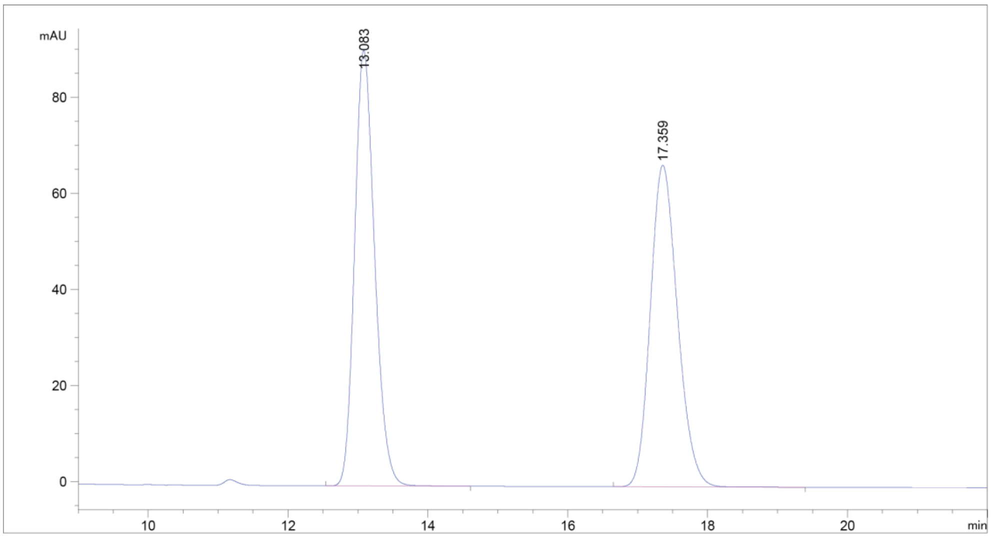

Peak #	RetTime [min]	Type	Width [min]	Area [mAU*s]	Height [mAU]	Area %
1	13.083	BB	0.3076	1808.28369	90.68888	49.9955
2	17.359	BB	0.4182	1808.60852	66.93791	50.0045

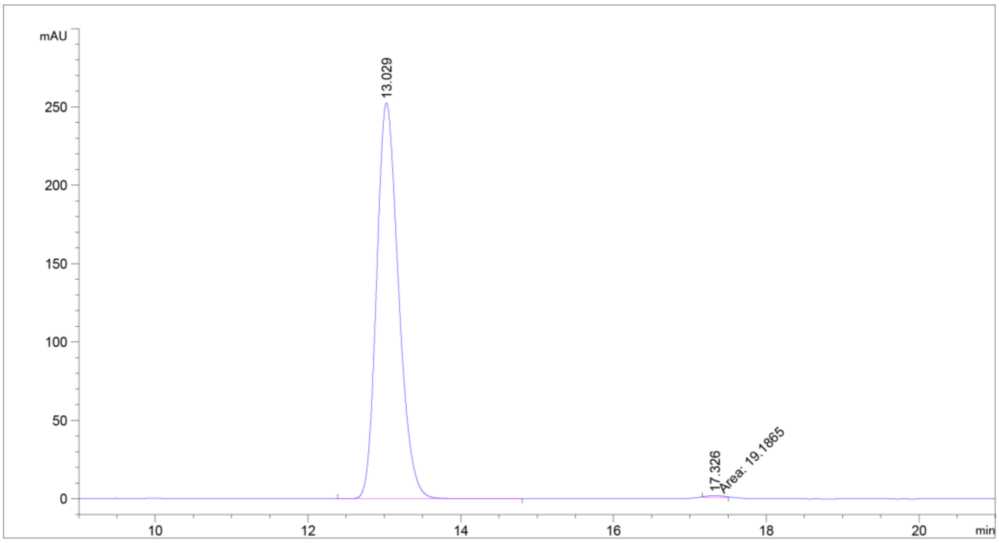

Peak #	RetTime [min]	Type	Width [min]	Area [mAU*s]	Height [mAU]	Area %
1	13.029	BB	0.3067	5037.71240	252.58574	99.6206
2	17.326	MM	0.2633	19.18654	1.21462	0.3794

(*S*)-**4b**: $[\alpha]^{25}_{D}$ = -7.1 (c = 0.33, $CHCl_3$); 97% ee (Chiralpak AD-3 column, ipropanol/hexane = 20/80, flow rate = 1.0 mL/min, λ = 254 nm); t_S (major) = 9.5 min, t_R (minor) = 11.9 min.

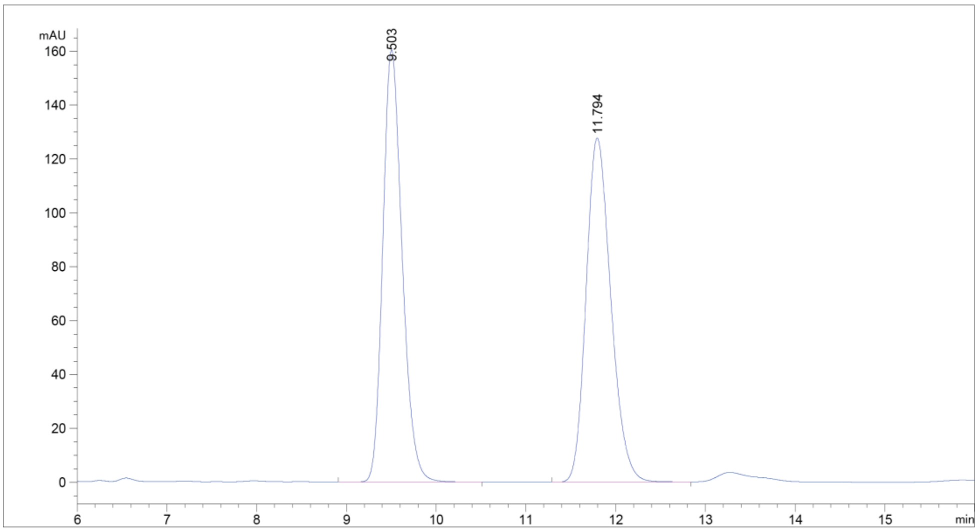

Peak #	RetTime [min]	Type	Width [min]	Area [mAU*s]	Height [mAU]	Area %
1	9.503	BB	0.2299	2405.69116	160.46025	49.5662
2	11.794	BB	0.2955	2447.79639	127.75167	50.4338

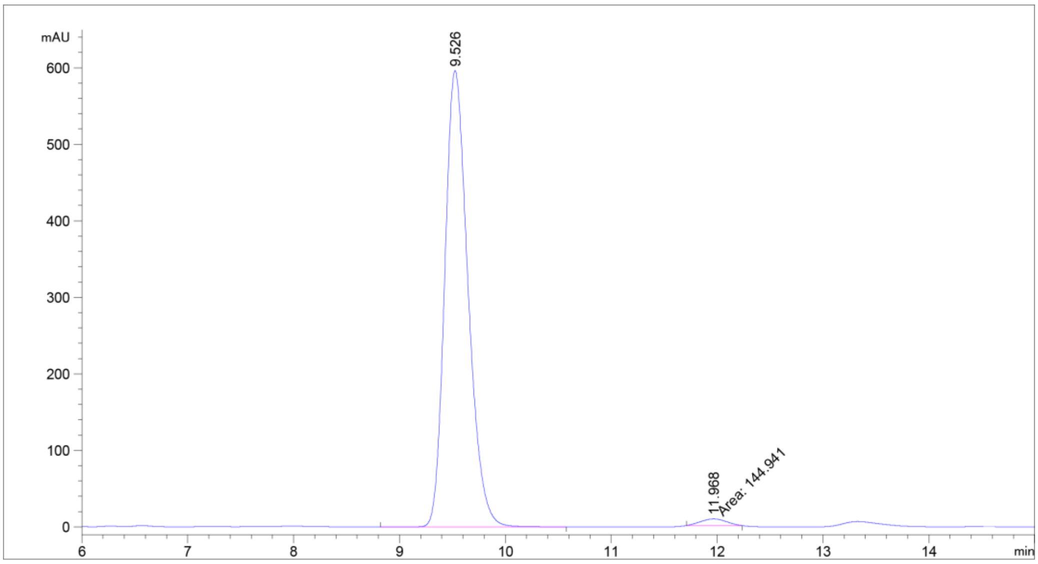

Peak #	RetTime [min]	Type	Width [min]	Area [mAU*s]	Height [mAU]	Area %
1	9.526	BB	0.2346	9080.82129	596.39551	98.4289
2	11.968	MM	0.2794	144.94144	8.64674	1.5711

(*S*)-**4c**: $[\alpha]^{25}_{D}$ = -41.4 (c = 0.32, $CHCl_3$); 99% ee (Chiralpak AD-3 column, ipropanol/hexane = 10/90, flow rate = 1.0 mL/min, λ = 254 nm); t_S (major) = 16.6 min, t_R (minor) = 17.6 min.

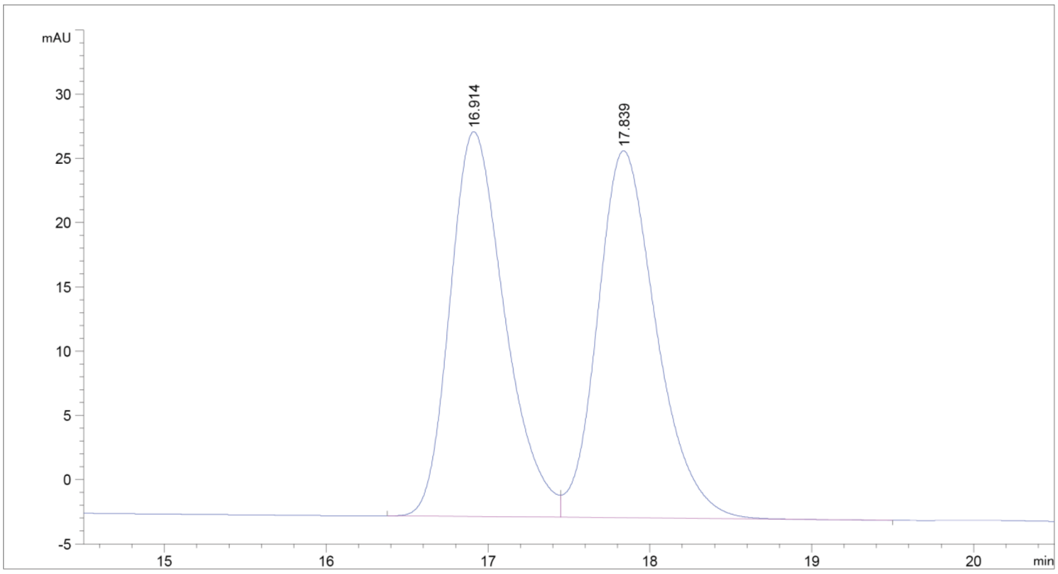

Peak #	RetTime [min]	Type	Width [min]	Area [mAU*s]	Height [mAU]	Area %
1	16.914	BV	0.3609	705.70459	29.94815	49.4137
2	17.839	VB	0.3859	722.45056	28.57774	50.5863

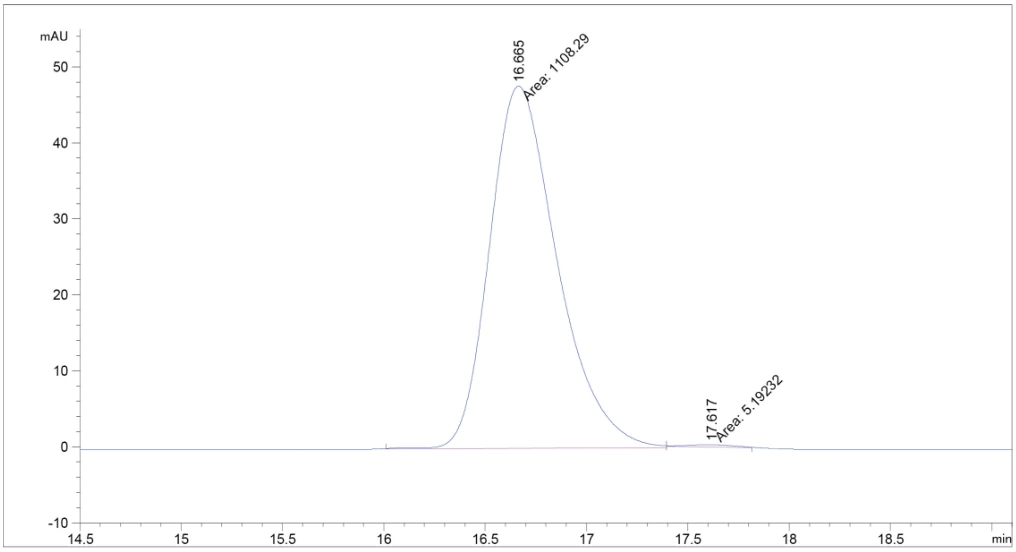

Peak #	RetTime [min]	Type	Width [min]	Area [mAU*s]	Height [mAU]	Area %
1	16.665	MM	0.3874	1108.28699	47.68507	99.5337
2	17.617	MM	0.2769	5.19232	3.12498e-1	0.4663

(S)-**4d**: $[\alpha]^{25}_{D}$ = -3.9 (c = 0.42, $CHCl_3$); 97% ee (Chiralpak AD-3 column, ipropanol/hexane = 10/90, flow rate = 1.0 mL/min, λ = 254 nm); t_S (major) = 25.3 min, t_R (minor) = 36.2 min.

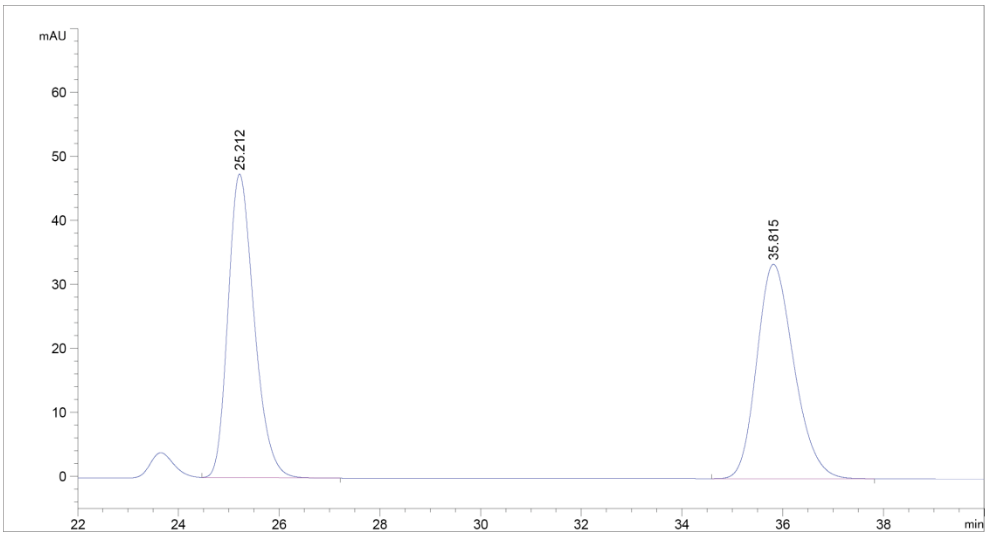

Peak #	RetTime [min]	Type	Width [min]	Area [mAU*s]	Height [mAU]	Area %
1	25.212	BB	0.5542	1708.95325	47.44477	49.8919
2	35.815	BB	0.7894	1716.35962	33.51901	50.1081

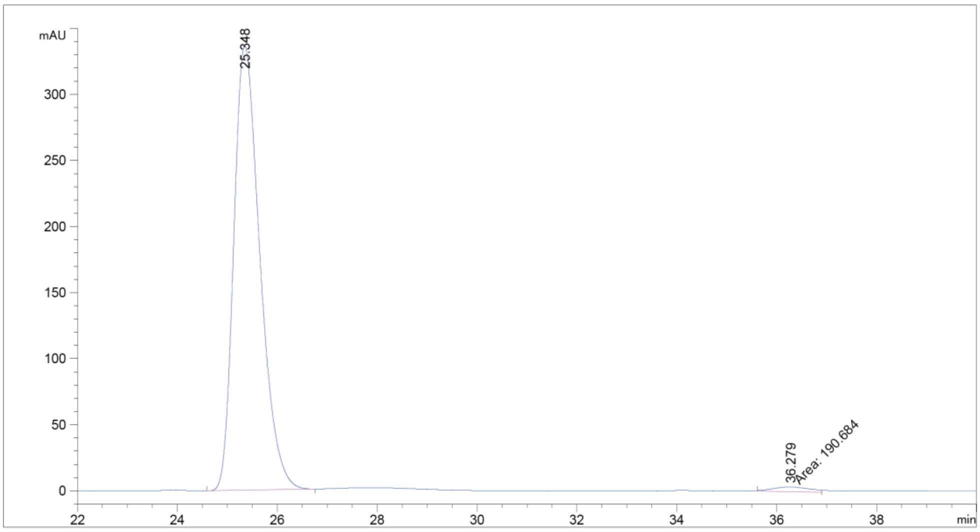

Peak #	RetTime [min]	Type	Width [min]	Area [mAU*s]	Height [mAU]	Area %
1	25.348	BB	0.5591	1.22283e4	336.41254	98.4646
2	36.279	MM	0.8747	190.68379	3.63330	1.5354

(*S*)-**4e**: $[\alpha]^{25}_{D}$ = 9.1 (c = 0.54, $CHCl_3$); 98% ee (Chiralpak AD-3 column, ipropanol/hexane = 10/90, flow rate = 1.0 mL/min, λ = 254 nm); t_S (major) = 30.4 min, t_R (minor) = 44.3 min.

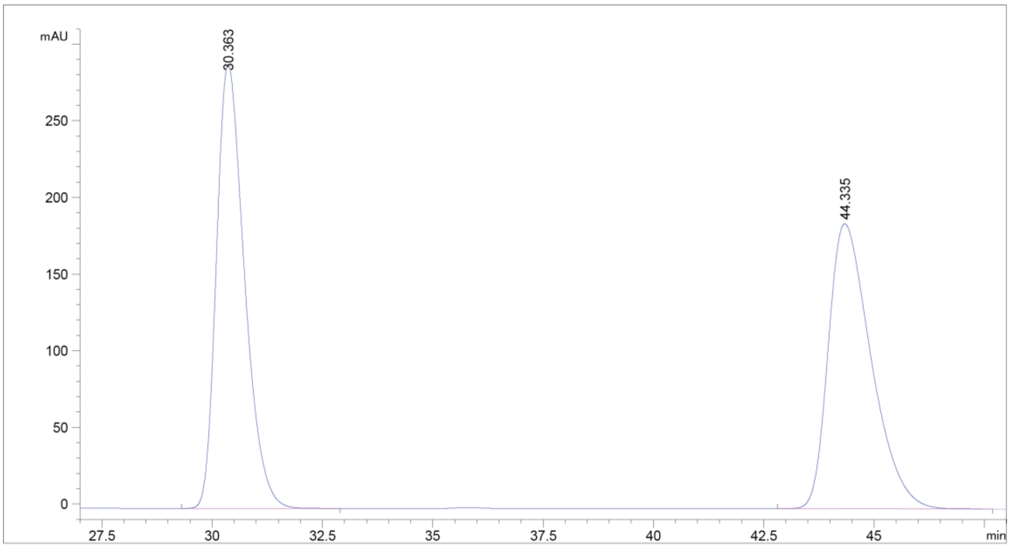

Peak #	RetTime [min]	Type	Width [min]	Area [mAU*s]	Height [mAU]	Area %
1	30.363	BB	0.6790	1.27867e4	289.68906	50.5537
2	44.335	BB	1.0357	1.25066e4	185.93443	49.4463

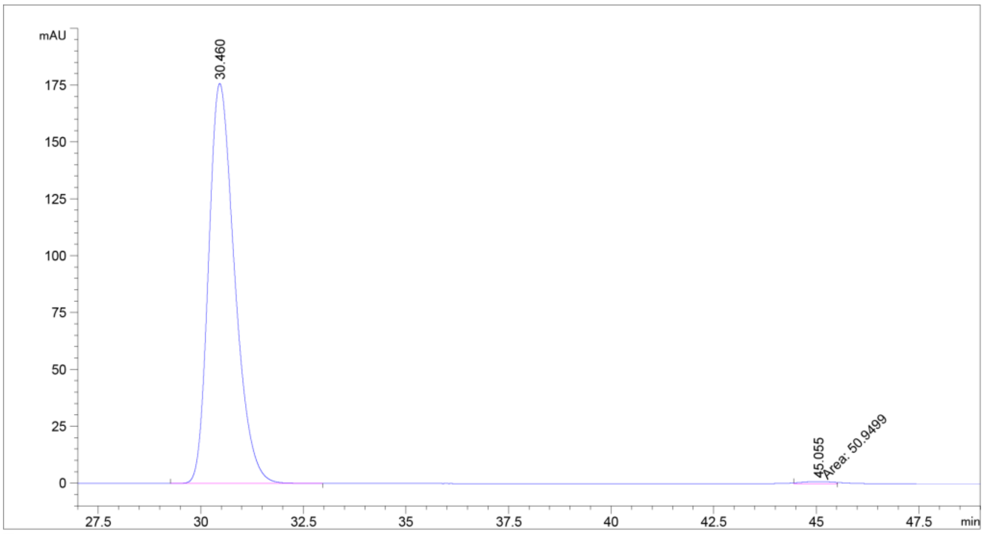

Peak #	RetTime [min]	Type	Width [min]	Area [mAU*s]	Height [mAU]	Area %
1	30.460	BB	0.6781	7747.62256	175.83356	99.3467
2	45.055	MM	0.8507	50.94991	9.98144e-1	0.6533

(*S*)-**4f**: $[\alpha]^{25}_{D}$ = -26.0 (c = 0.92, $CHCl_3$); 96% ee (Chiralpak AD-3 column, ipropanol/hexane = 20/80, flow rate = 1.0 mL/min, λ = 254 nm); t_S (major) = 9.7 min, t_R (minor) = 12.8 min.

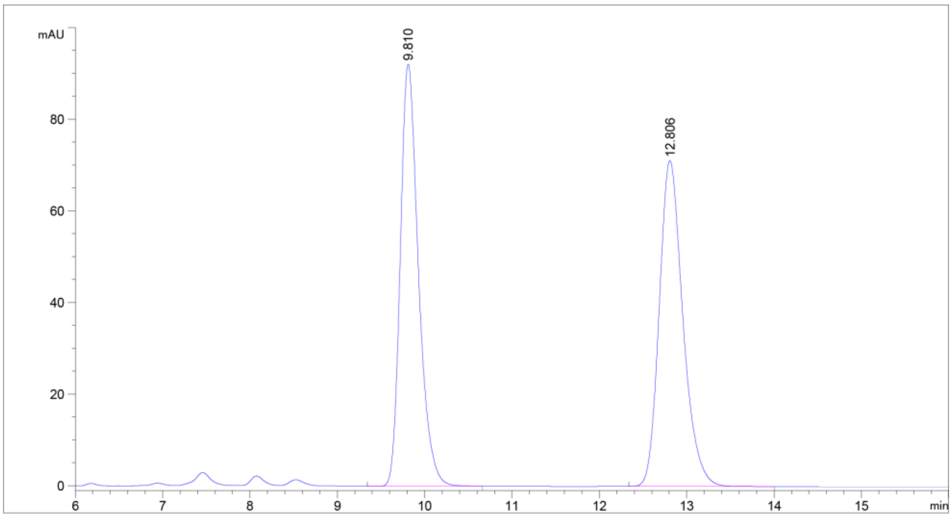

Peak #	RetTime [min]	Type	Width [min]	Area [mAU*s]	Height [mAU]	Area %
1	9.810	BB	0.2193	1328.66687	92.09854	49.8456
2	12.806	BB	0.2876	1336.89856	71.01742	50.1544

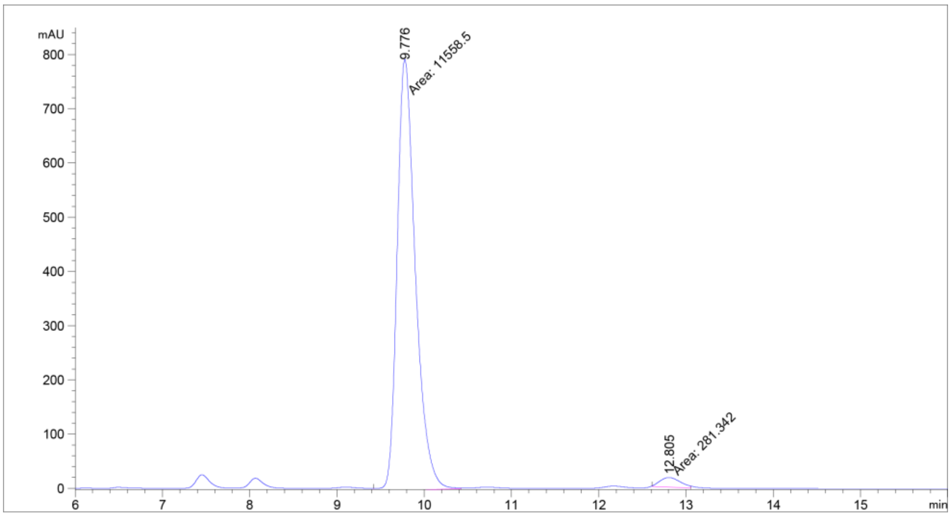

Peak #	RetTime [min]	Type	Width [min]	Area [mAU*s]	Height [mAU]	Area %
1	9.776	MM	0.2425	1.15585e4	794.36542	97.6238
2	12.805	MM	0.2666	281.34198	17.59090	2.3762

(*S*)-**5a**: $[\alpha]^{25}_D$ = 6.8 (c = 0.43, $CHCl_3$); 98% ee (Chiralpak AD-3 column, ipropanol/hexane = 20/80, flow rate = 1.0 mL/min, λ = 254 nm); t_S (major) = 6.1 min, t_R (minor) = 6.7 min.

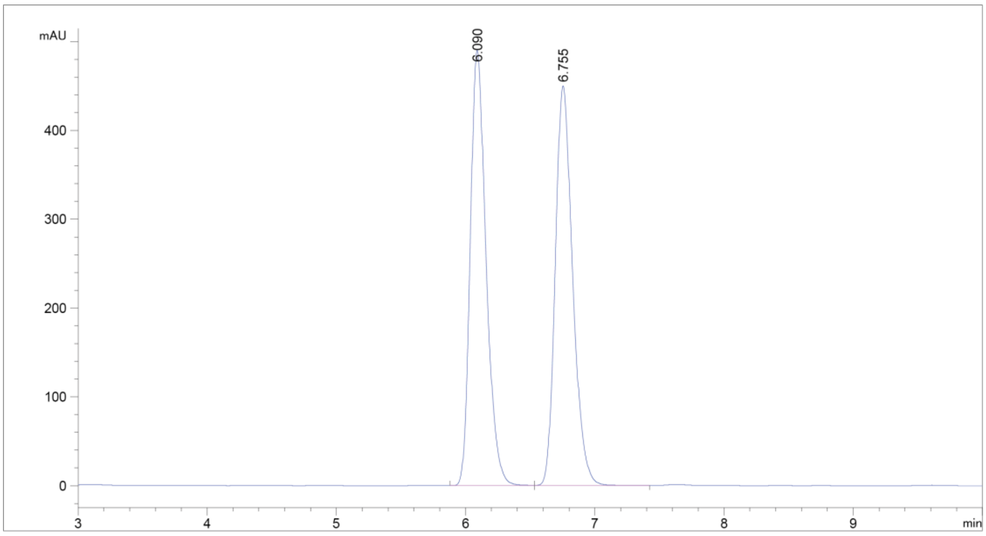

Peak #	RetTime [min]	Type	Width [min]	Area [mAU*s]	Height [mAU]	Area %
1	6.090	BB	0.1292	4169.65381	490.25839	49.7885
2	6.755	BB	0.1420	4205.07568	449.89063	50.2115

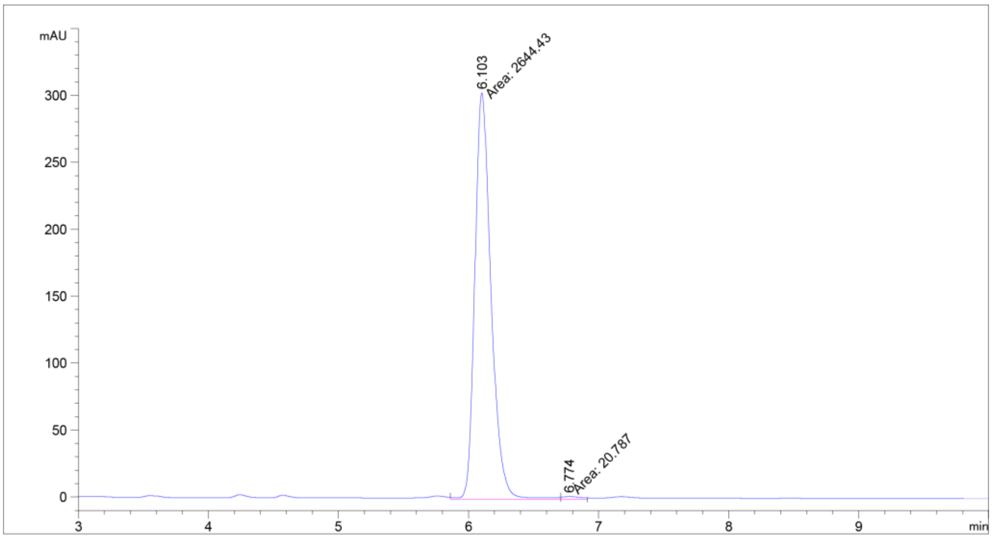

Peak #	RetTime [min]	Type	Width [min]	Area [mAU*s]	Height [mAU]	Area %
1	6.103	MF	0.1451	2644.43384	303.72220	99.2201
2	6.774	FM	0.1661	20.78698	2.08558	0.7799

(*S*)-**5b**: $[\alpha]^{25}_{D}$ = 42.2 (c = 0.45, $CHCl_3$); 98% ee (Chiralpak AD-3 column, ipropanol/hexane = 20/80, flow rate = 1.0 mL/min, λ = 254 nm); t_S (major) = 6.5 min, t_R (minor) = 8.1 min.

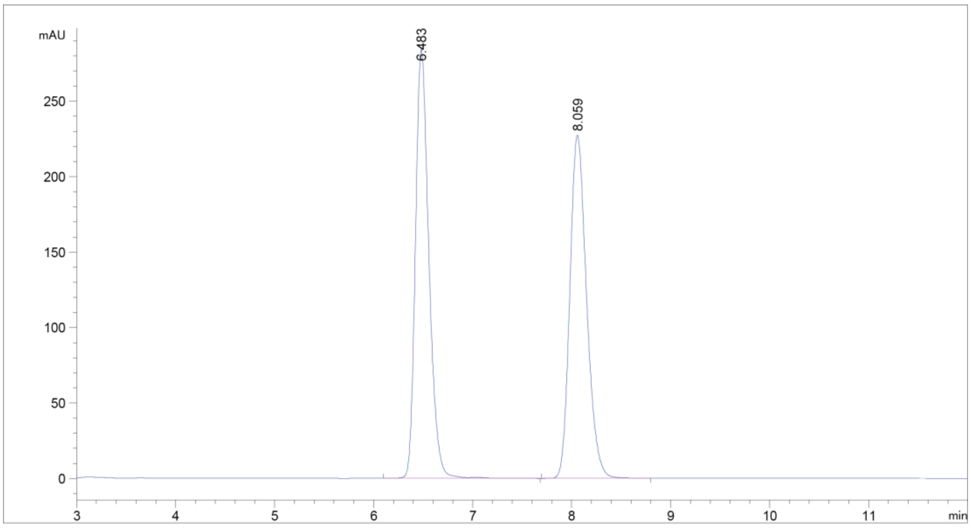

Peak #	RetTime [min]	Type	Width [min]	Area [mAU*s]	Height [mAU]	Area %
1	6.483	BV R	0.1442	2686.79199	284.47055	50.1718
2	8.059	BB	0.1798	2668.39453	227.53583	49.8282

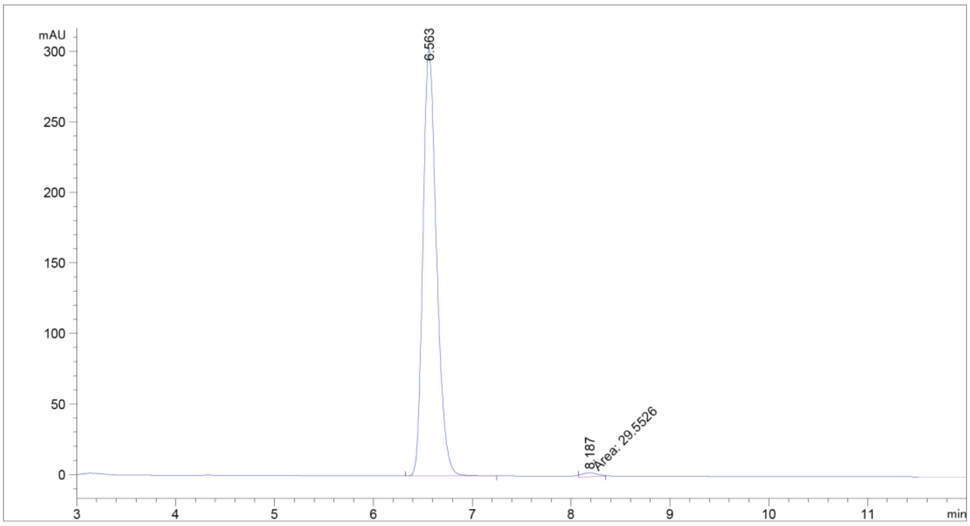

Peak #	RetTime [min]	Type	Width [min]	Area [mAU*s]	Height [mAU]	Area %
1	6.563	BB	0.1464	2889.52954	302.66861	98.9876
2	8.187	MM	0.1803	29.55256	2.73254	1.0124

(S)-**5c**: $[\alpha]^{25}_{D}$ = -14.2 (c = 0.44, $CHCl_3$); 96% ee (Chiralpak AD-3 column, ipropanol/hexane = 20/80, flow rate = 1.0 mL/min, λ = 254 nm); t_S (major) = 6.7 min, t_R (minor) = 12.5 min.

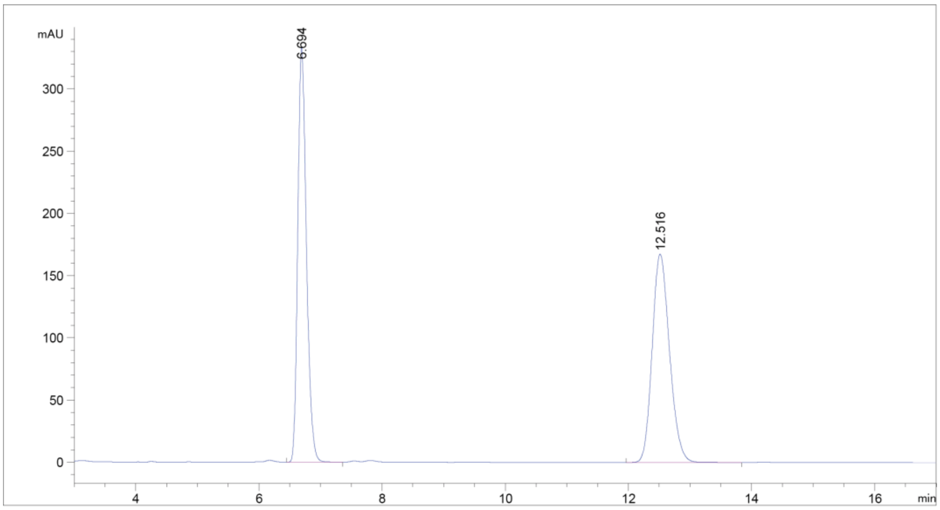

Peak #	RetTime [min]	Type	Width [min]	Area [mAU*s]	Height [mAU]	Area %
1	6.694	BB	0.1501	3258.42407	333.08322	49.9266
2	12.516	BB	0.3018	3268.00879	167.34959	50.0734

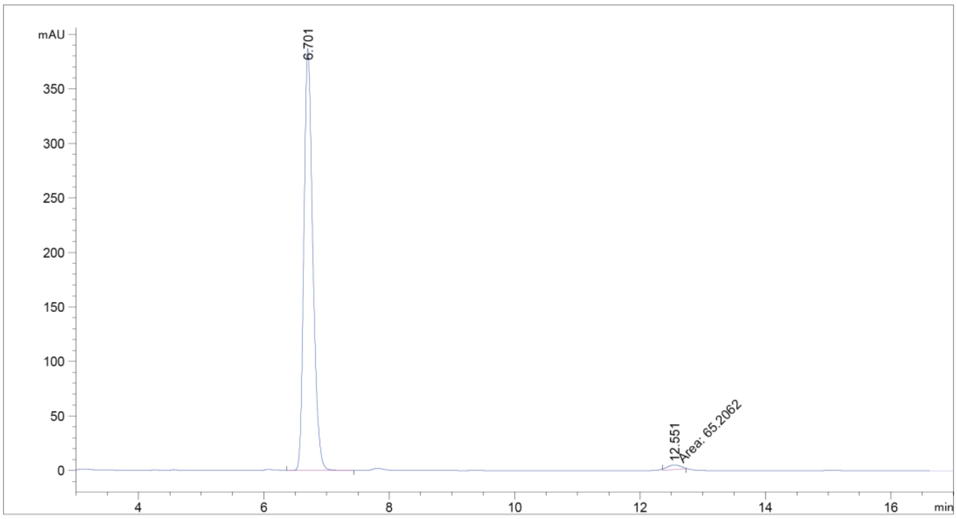

Peak #	RetTime [min]	Type	Width [min]	Area [mAU*s]	Height [mAU]	Area %
1	6.701	BB	0.1521	3816.72778	386.87695	98.3203
2	12.551	MM	0.2551	65.20621	4.25943	1.6797

(*S*)-**5d**: $[\alpha]^{25}_{D}$ = 1.0 (c = 0.52, $CHCl_3$); 98% ee (Chiralpak AD-3 column, ipropanol/hexane = 15/85, flow rate = 1.0 mL/min, λ = 254 nm); t_S (major) = 7.5 min, t_R (minor) = 8.3 min.

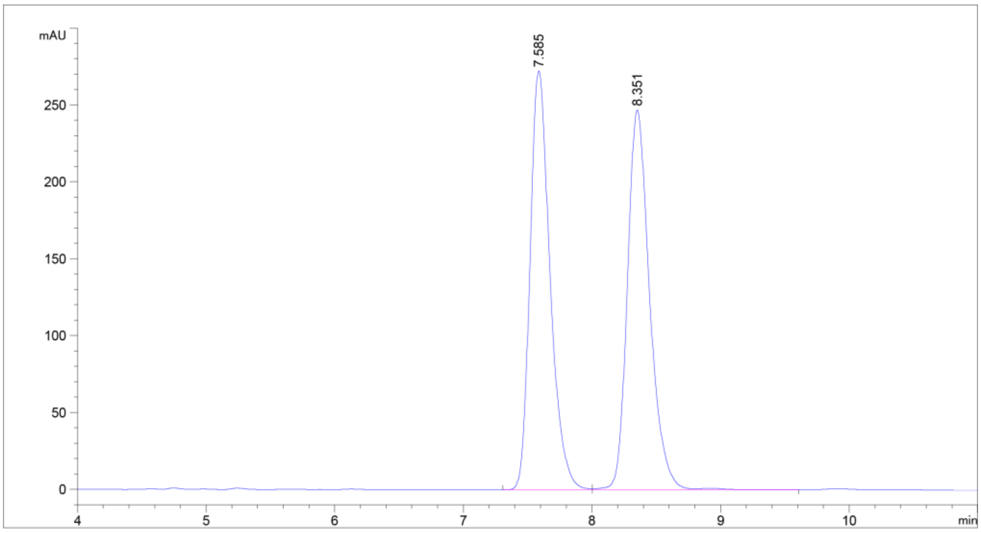

Peak #	RetTime [min]	Type	Width [min]	Area [mAU*s]	Height [mAU]	Area %
1	7.585	BV	0.1639	2940.09717	272.52869	49.7247
2	8.351	VV R	0.1824	2972.65308	247.08623	50.2753

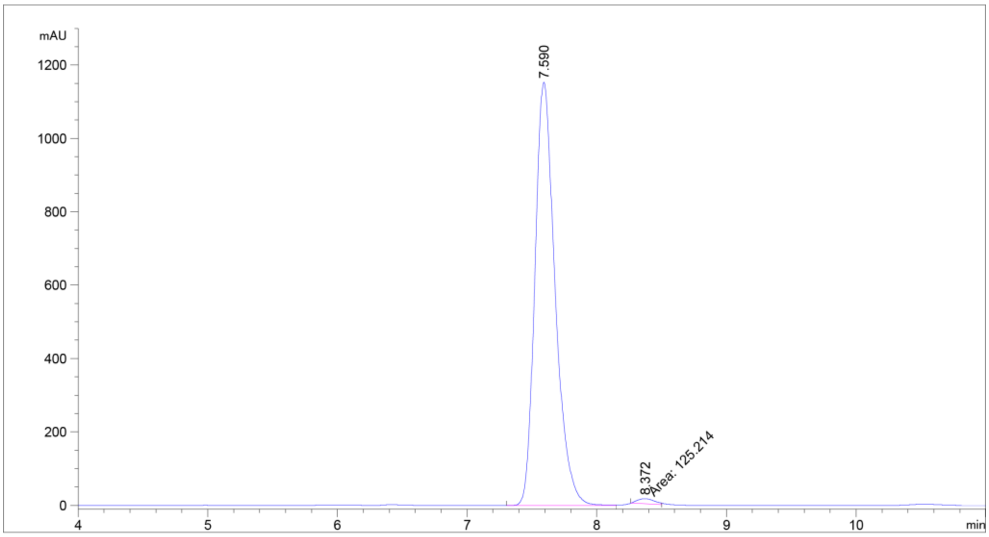

Peak #	RetTime [min]	Type	Width [min]	Area [mAU*s]	Height [mAU]	Area %
1	7.590	BV	0.1652	1.24738e4	1152.97986	99.0062
2	8.372	MM	0.1514	125.21367	13.78676	0.9938

(*S*)-**5e**: $[\alpha]^{25}_{D}$ = -11.3 (c = 0.80, $CHCl_3$); 98% ee (Chiralpak AD-3 column, ipropanol/hexane = 15/85, flow rate = 1.0 mL/min, λ = 254 nm); t_S (major) = 7.0 min, t_R (minor) = 10.3 min.

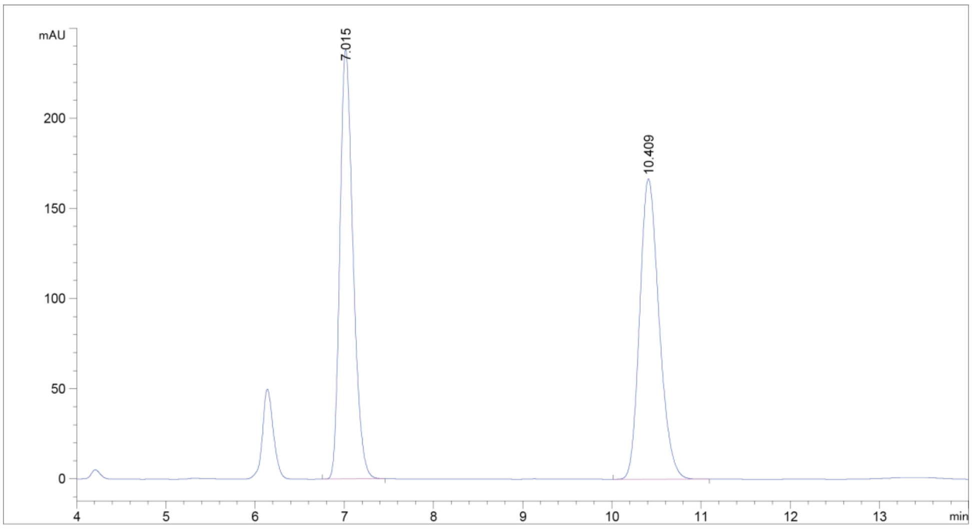

Peak #	RetTime [min]	Type	Width [min]	Area [mAU*s]	Height [mAU]	Area %
1	7.015	BB	0.1580	2450.48706	238.28854	49.2879
2	10.409	BB	0.2325	2521.29419	166.62601	50.7121

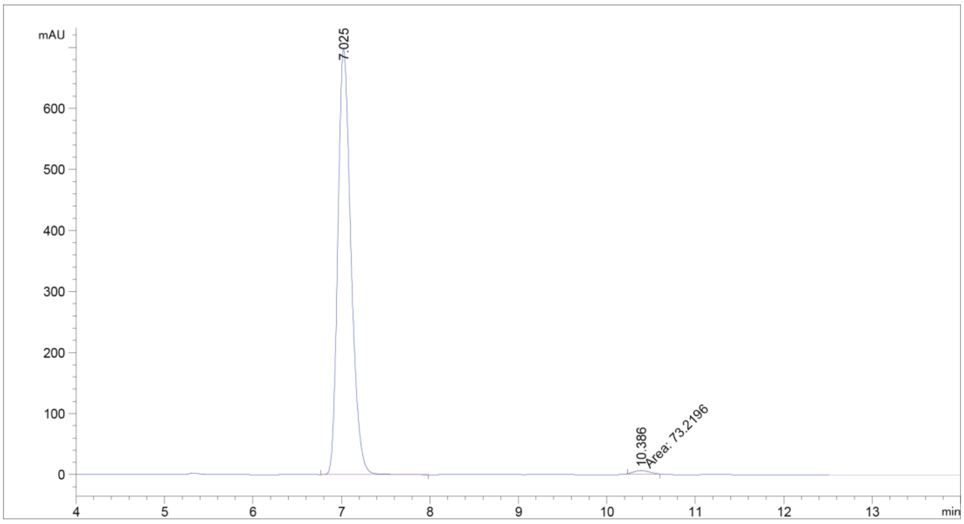

Peak #	RetTime [min]	Type	Width [min]	Area [mAU*s]	Height [mAU]	Area %
1	7.025	BB	0.1586	7210.68213	697.66528	98.9948
2	10.386	MM	0.2104	73.21957	5.80046	1.0052

(*S*)-**5f**: $[\alpha]^{25}_{D}$ = 0.5 (c = 0.35, $CHCl_3$); 95% ee (Chiralpak AD-3 column, ipropanol/hexane = 15/85, flow rate = 1.0 mL/min, λ = 254 nm); t_S (major) = 5.6 min, t_R (minor) = 7.0 min.

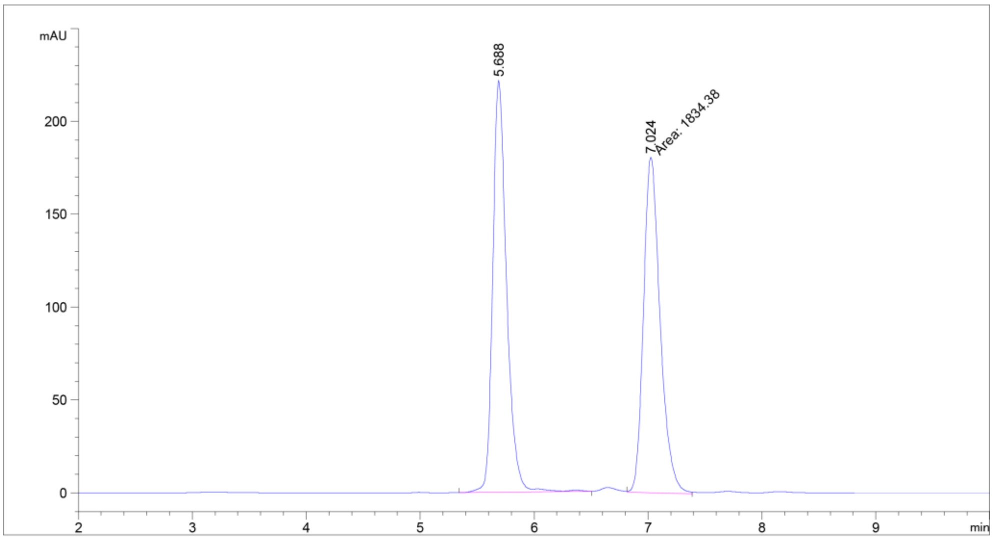

Peak #	RetTime [min]	Type	Width [min]	Area [mAU*s]	Height [mAU]	Area %
1	5.688	BV R	0.1266	1871.95667	221.56970	50.5070
2	7.024	MM	0.1694	1834.37793	180.50447	49.4930

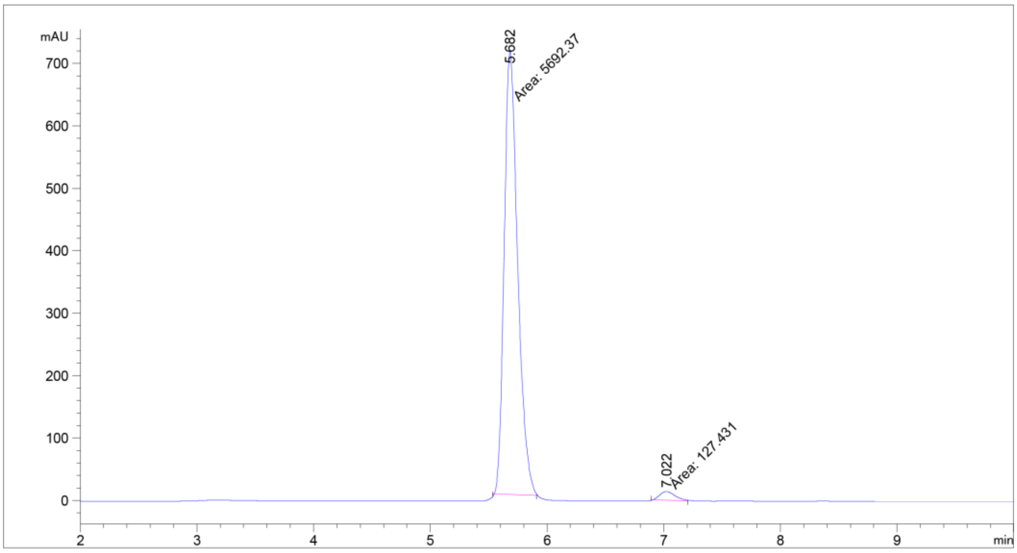

Peak #	RetTime [min]	Type	Width [min]	Area [mAU*s]	Height [mAU]	Area %
1	5.682	MM	0.1337	5692.37354	709.35907	97.8104
2	7.022	MM	0.1513	127.43066	14.03972	2.1896

(*S*)-**9a**: $[\alpha]^{25}_{D}$ = -8.1 (c = 0.52, $CHCl_3$); 94% ee (Chiralpak AD-3 column, ipropanol/hexane = 15/85, flow rate = 1.0 mL/min, λ = 254 nm); t_S (major) = 7.1 min, t_R (minor) = 8.7 min.

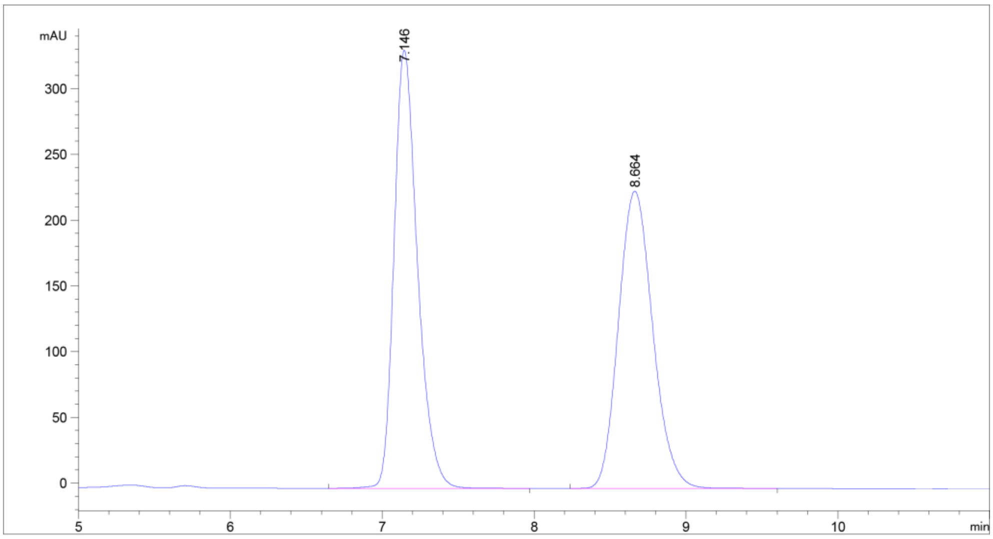

Peak #	RetTime [min]	Type	Width [min]	Area [mAU*s]	Height [mAU]	Area %
1	7.146	BB	0.1632	3576.57642	333.18561	50.2216
2	8.664	BB	0.2450	3545.01245	226.05901	49.7784

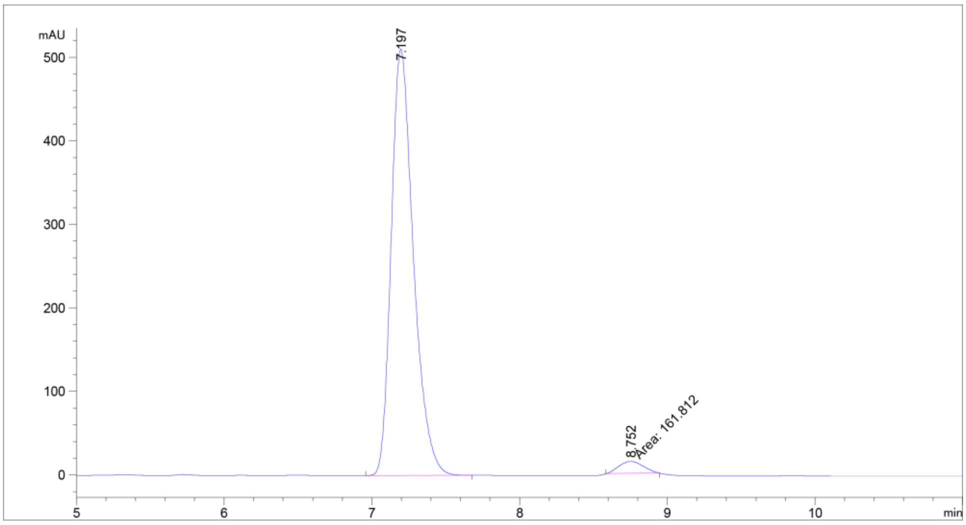

Peak #	RetTime [min]	Type	Width [min]	Area [mAU*s]	Height [mAU]	Area %
1	7.197	BB	0.1590	5337.41895	510.57211	97.0575
2	8.752	MM	0.1905	161.81215	14.15545	2.9425

(S)-**9b**: $[\alpha]^{25}_{D}$ = -9.4 (c = 0.64, $CHCl_3$); 94% ee (Chiralpak AD-3 column, ipropanol/hexane = 10/90, flow rate = 0.5 mL/min, λ = 254 nm); t_S (major) = 21.6 min, t_R (minor) = 23.0 min.

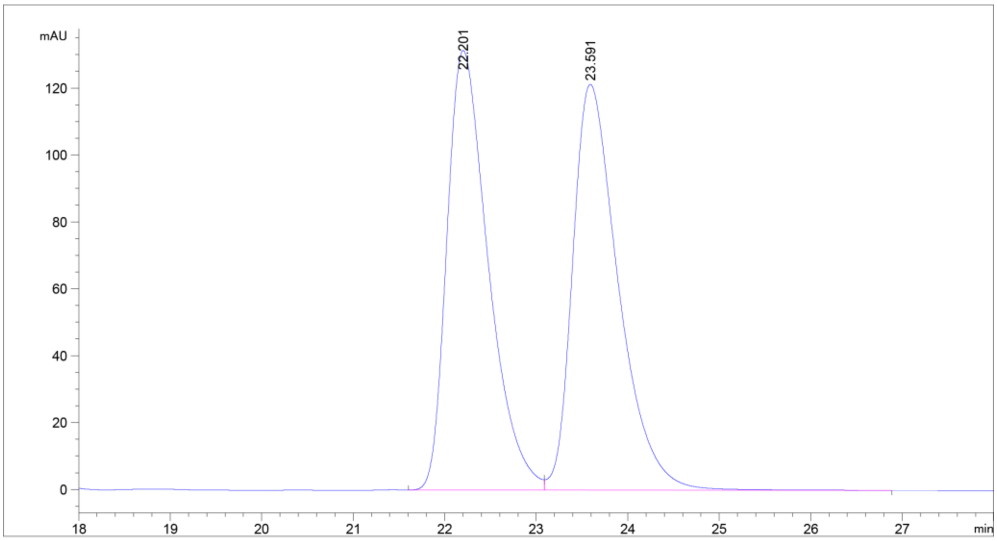

Peak #	RetTime [min]	Type	Width [min]	Area [mAU*s]	Height [mAU]	Area %
1	22.201	BV	0.4790	4122.84668	131.41022	49.4550
2	23.591	VB	0.5272	4213.71729	121.32672	50.5450

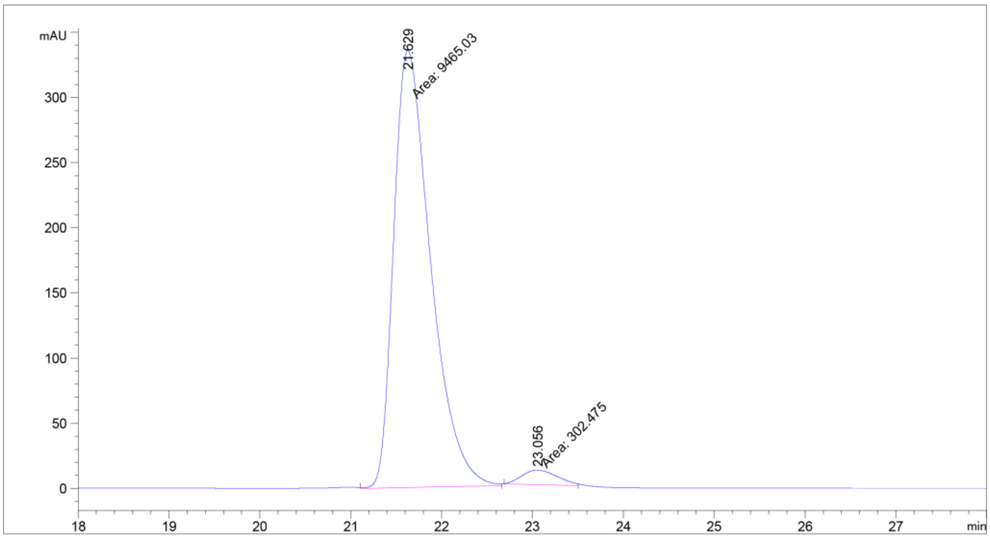

Peak #	RetTime [min]	Type	Width [min]	Area [mAU*s]	Height [mAU]	Area %
1	21.629	MM	0.4703	9465.02637	335.43005	96.9033
2	23.056	MM	0.4510	302.47488	11.17870	3.0967

(*S*)-**9c**: $[\alpha]^{25}_{D}$ = -4.4 (c = 0.49, $CHCl_3$); 94% ee (Chiralpak AD-3 column, ipropanol/hexane = 15/85, flow rate = 0.5 mL/min, λ = 254 nm); t_S (major) = 20.0 min, t_R (minor) = 18.0 min.

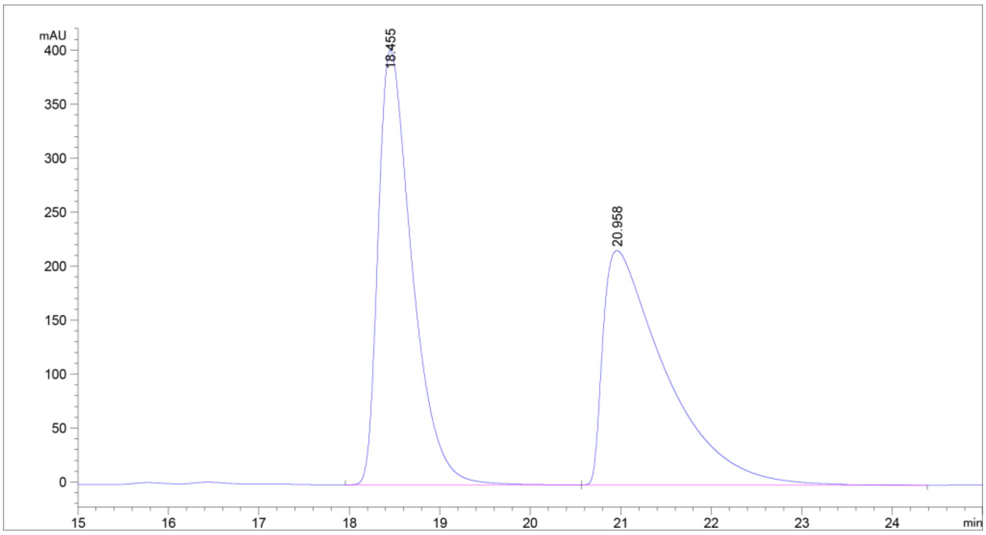

Peak #	RetTime [min]	Type	Width [min]	Area [mAU*s]	Height [mAU]	Area %
1	18.455	BB	0.3939	1.04931e4	402.82208	50.1905
2	20.958	BB	0.6917	1.04134e4	217.06602	49.8095

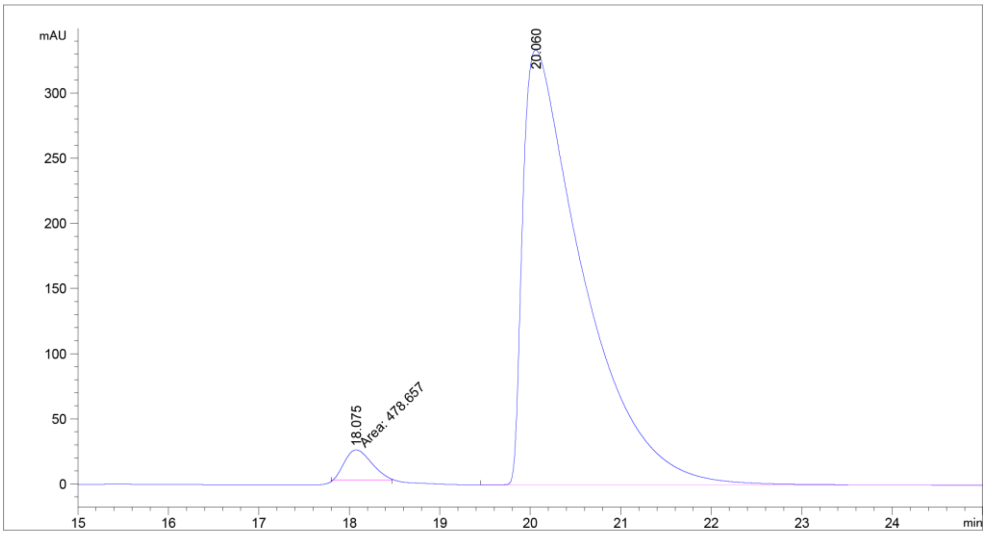

Peak #	RetTime [min]	Type	Width [min]	Area [mAU*s]	Height [mAU]	Area %
1	18.075	MM	0.3438	478.65689	23.20364	2.9986
2	20.060	BB	0.6651	1.54841e4	333.87048	97.0014

(S)-**9e**: 94% ee (Chiralpak AD-3 column, ipropanol/hexane = 15/85, flow rate = 1.0 mL/min, λ = 254 nm); t_S (major) = 4.5 min, t_R (minor) = 4.9 min.

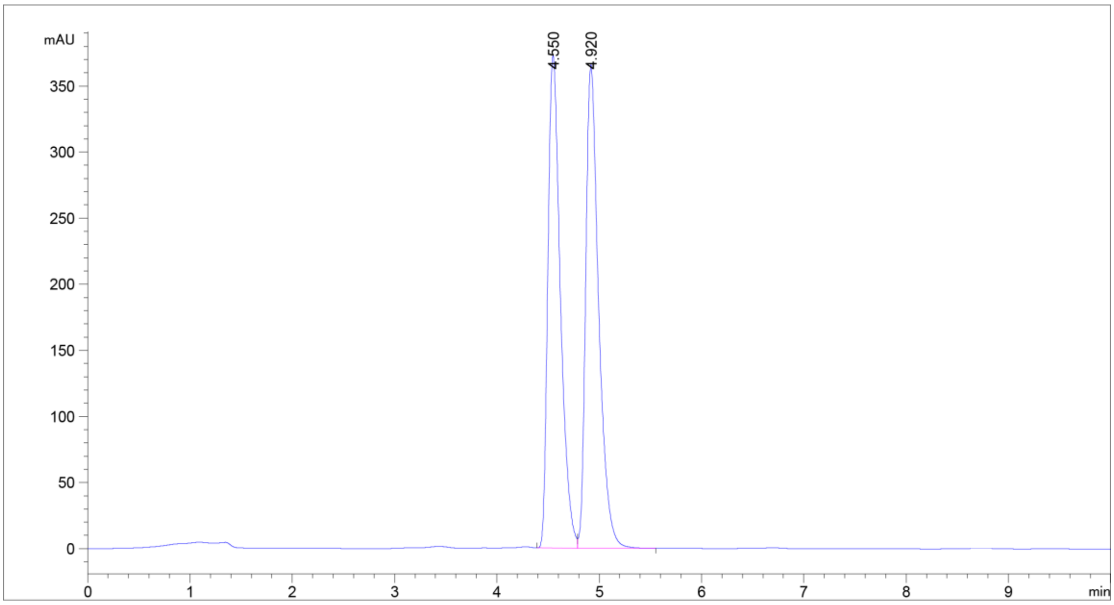

Peak #	RetTime [min]	Type	Width [min]	Area [mAU*s]	Height [mAU]	Area %
1	4.550	BV	0.1300	3160.62061	372.36292	49.6384
2	4.920	VB	0.1313	3206.66724	365.81458	50.3616

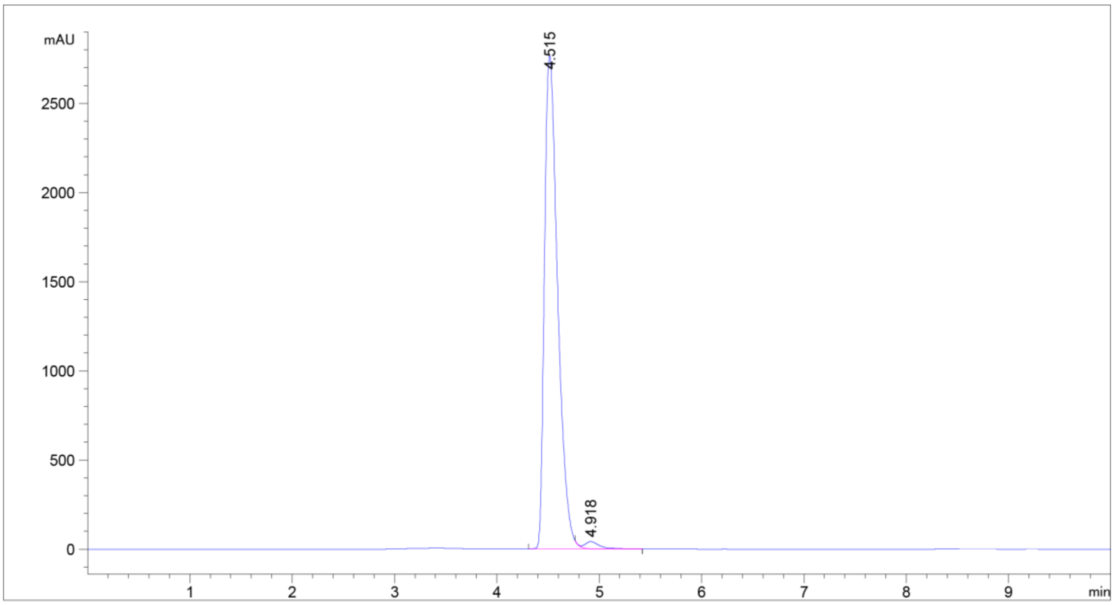

Peak #	RetTime [min]	Type	Width [min]	Area [mAU*s]	Height [mAU]	Area %
1	4.515	BV R	0.1389	2.48515e4	2763.97339	97.3799
2	4.918	VB E	0.1485	409.25858	40.30367	2.6201

(*S*)-**9d**: $[\alpha]^{25}_{D}$ = -6.3 (c = 0.49, $CHCl_3$); 94% ee (Chiralpak AD-3 column, ipropanol/hexane = 15/85, flow rate = 0.5 mL/min, λ = 254 nm); t_S (major) = 25.3 min, t_R (minor) = 18.0 min.

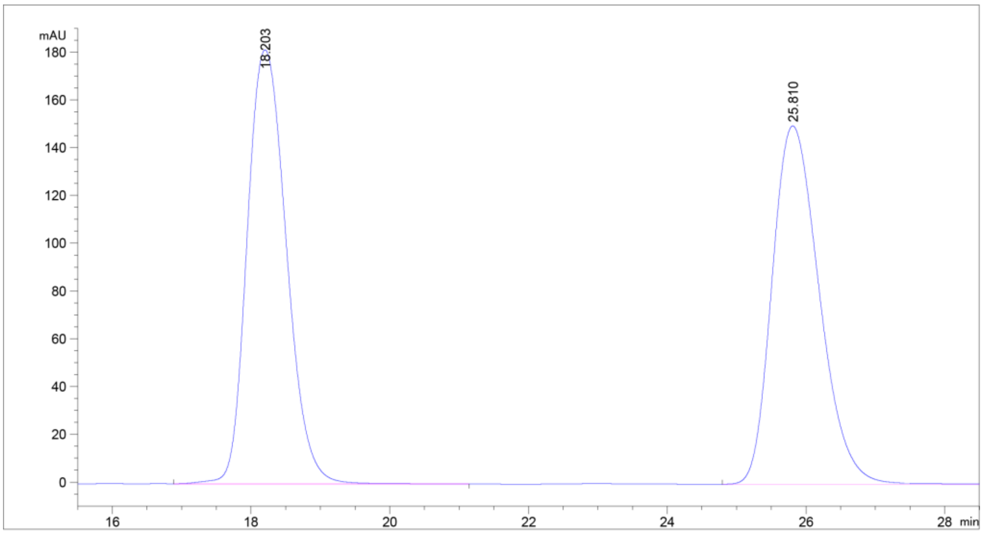

Peak #	RetTime [min]	Type	Width [min]	Area [mAU*s]	Height [mAU]	Area %
1	18.203	BB	0.6256	7140.61182	181.65553	50.2523
2	25.810	BB	0.7409	7068.91211	149.97888	49.7477

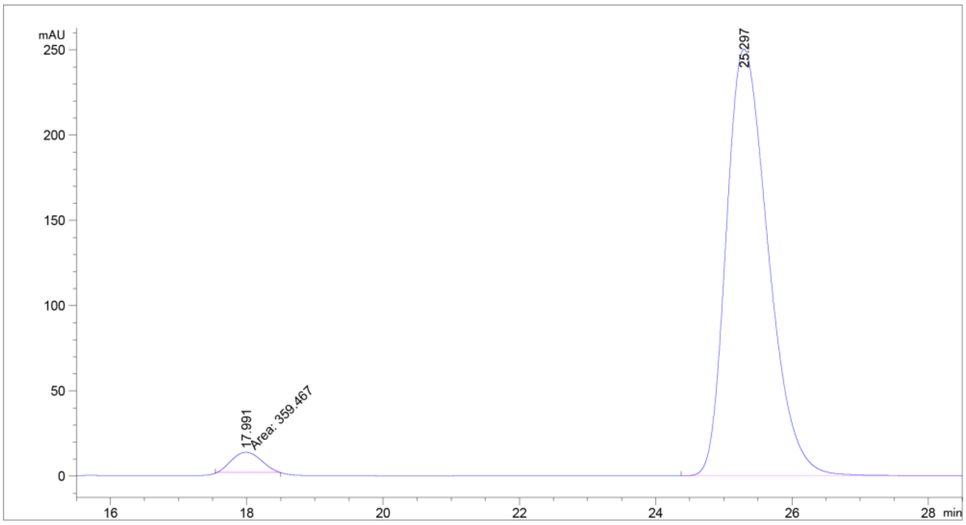

Peak #	RetTime [min]	Type	Width [min]	Area [mAU*s]	Height [mAU]	Area %
1	17.991	MM	0.5043	359.46735	11.88027	3.2585
2	25.297	BB	0.6656	1.06721e4	250.22238	96.7415

(*S*)-**9e**: 94% ee (Chiralpak AD-3 column, ipropanol/hexane = 15/85, flow rate = 1.0 mL/min, λ = 254 nm); t_S (major) = 4.5 min, t_R (minor) = 4.9 min.

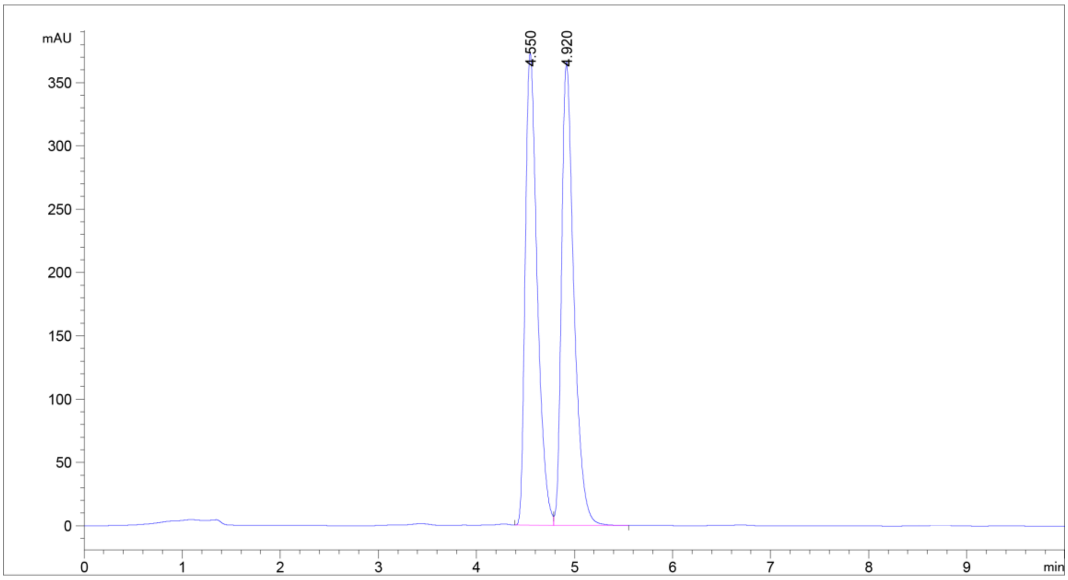

Peak #	RetTime [min]	Type	Width [min]	Area [mAU*s]	Height [mAU]	Area %
1	4.550	BV	0.1300	3160.62061	372.36292	49.6384
2	4.920	VB	0.1313	3206.66724	365.81458	50.3616

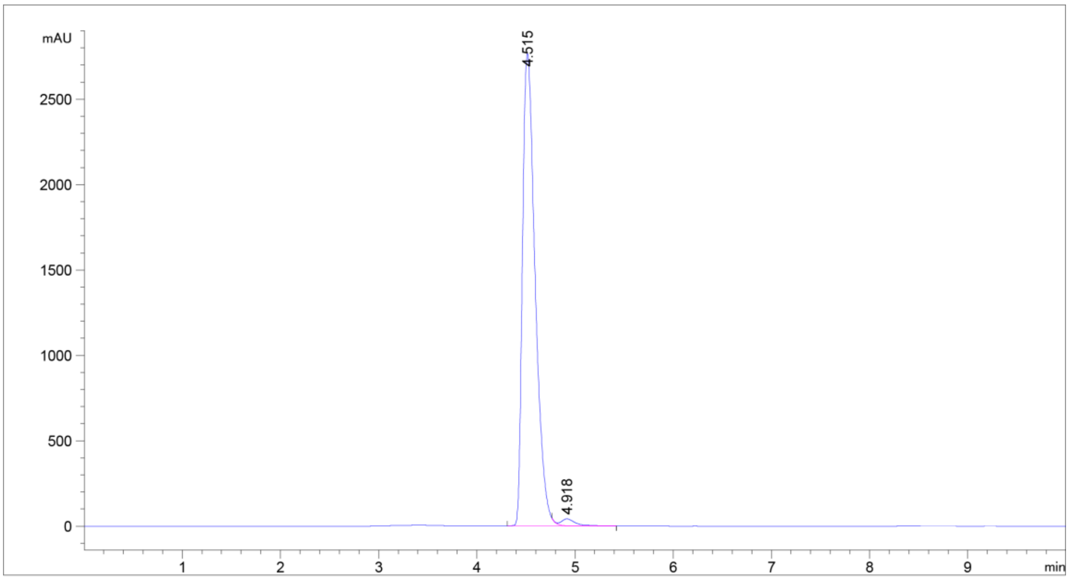

Peak #	RetTime [min]	Type	Width [min]	Area [mAU*s]	Height [mAU]	Area %
1	4.515	BV R	0.1389	2.48515e4	2763.97339	97.3799
2	4.918	VB E	0.1485	409.25858	40.30367	2.6201

(*S*)-**2a**: 98% ee (Chiralcel OD-3 column, ipropanol/hexane = 10/90, flow rate = 1.0 mL/min, λ = 220 nm); t_S (major) = 7.6 min, t_R (minor) = 8.3 min.

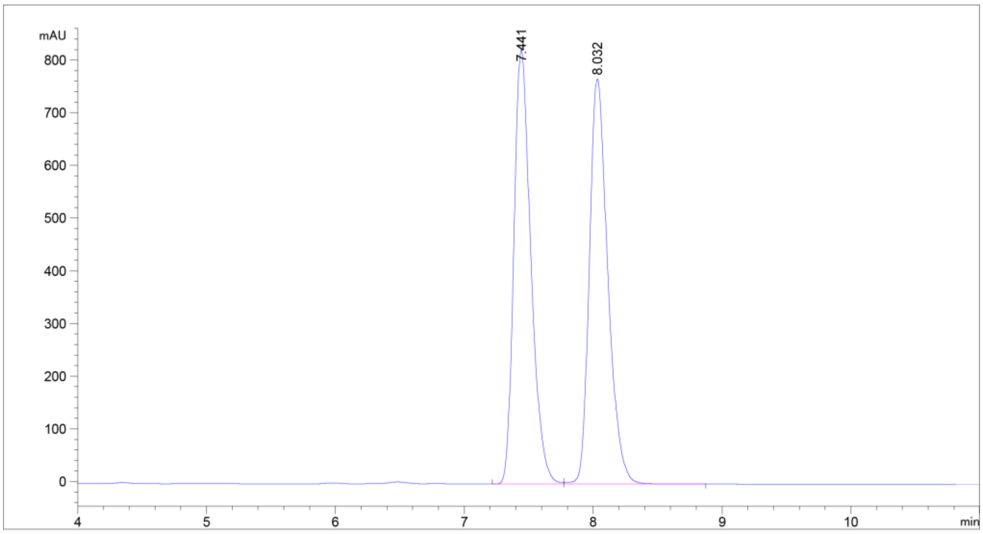

Peak #	RetTime [min]	Type	Width [min]	Area [mAU*s]	Height [mAU]	Area %
1	7.441	BV	0.1400	7564.61572	824.61041	49.7219
2	8.032	VB	0.1520	7649.22998	769.49200	50.2781

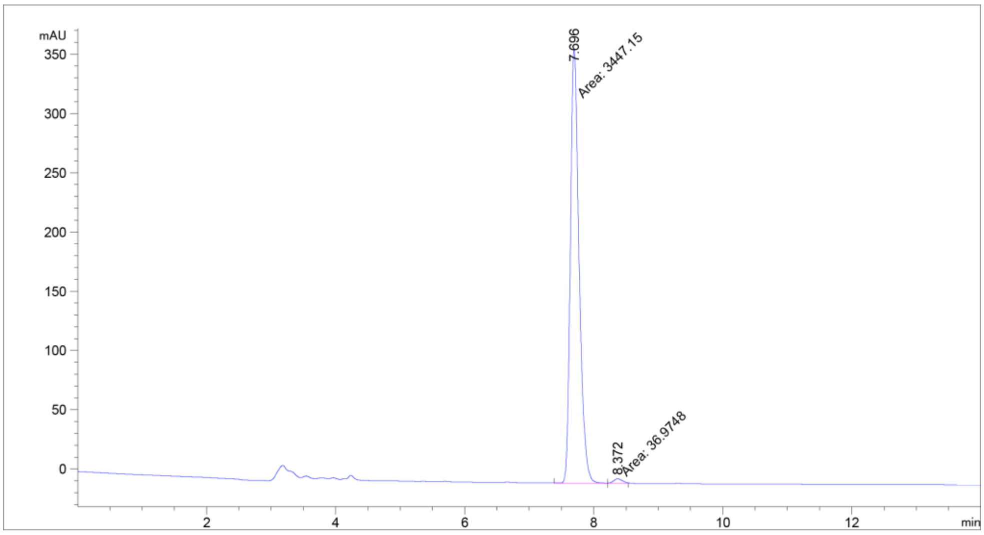

Peak #	RetTime [min]	Type	Width [min]	Area [mAU*s]	Height [mAU]	Area %
1	7.696	MF	0.1571	3447.14575	365.64771	98.9388
2	8.372	FM	0.1610	36.97480	3.82749	1.0612

(*S*)-**7c**: 60% ee (Chiralcel OJ-H column, ipropanol/hexane = 30/70, flow rate = 1.0 mL/min, λ = 220 nm); t_S (major) = 4.9 min, t_R (minor) = 4.7 min.

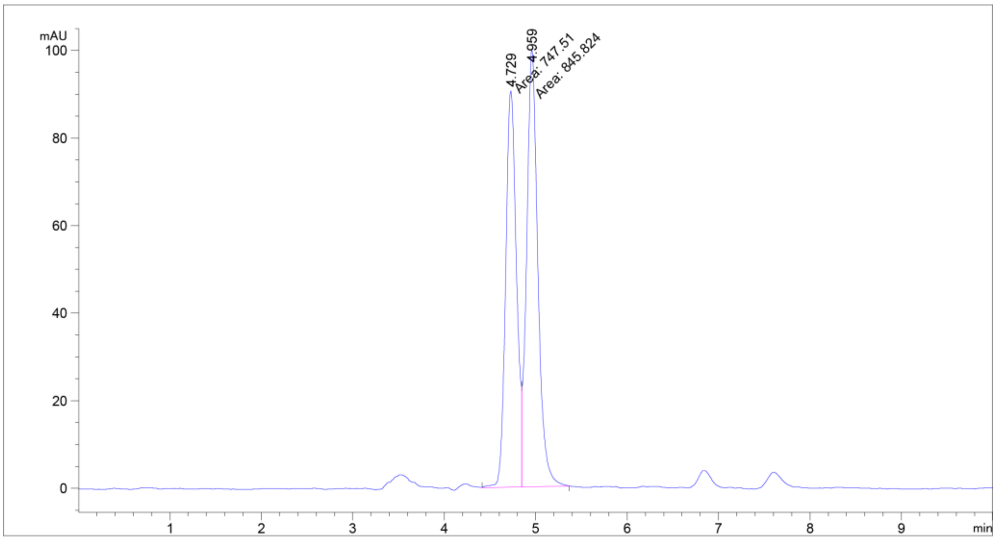

Peak #	RetTime [min]	Type	Width [min]	Area [mAU*s]	Height [mAU]	Area %
1	4.729	MF	0.1376	747.50952	90.56753	46.9148
2	4.959	FM	0.1411	845.82422	99.88034	53.0852

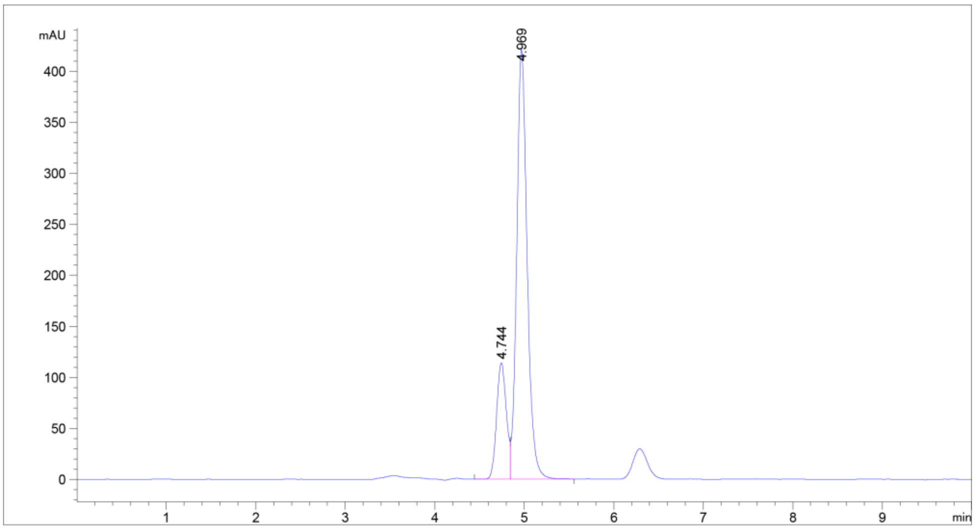

Peak #	RetTime [min]	Type	Width [min]	Area [mAU*s]	Height [mAU]	Area %
1	4.744	BV	0.1183	862.84003	113.97906	20.2122
2	4.969	VB	0.1244	3406.06592	420.84299	79.7878

(*S*)-**7d**: 81% ee (Chiralcel OD-3 column, ipropanol/hexane = 5/95, flow rate = 0.5 mL/min, λ = 220 nm); t_S (major) = 10.8 min, t_R (minor) = 9.5 min.

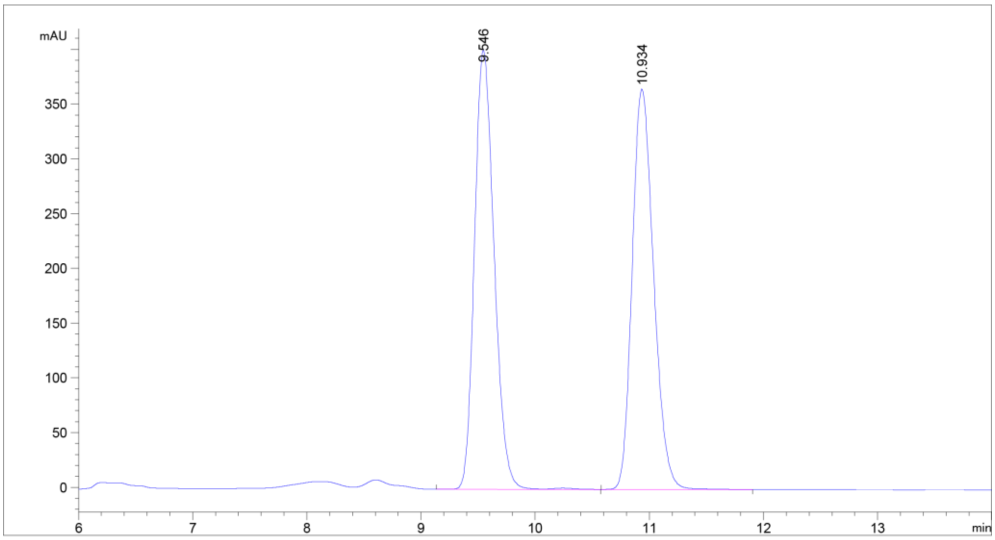

Peak #	RetTime [min]	Type	Width [min]	Area [mAU*s]	Height [mAU]	Area %
1	9.546	BV R	0.1853	4786.17725	400.74759	50.1279
2	10.934	BB	0.2020	4761.75879	365.53519	49.8721

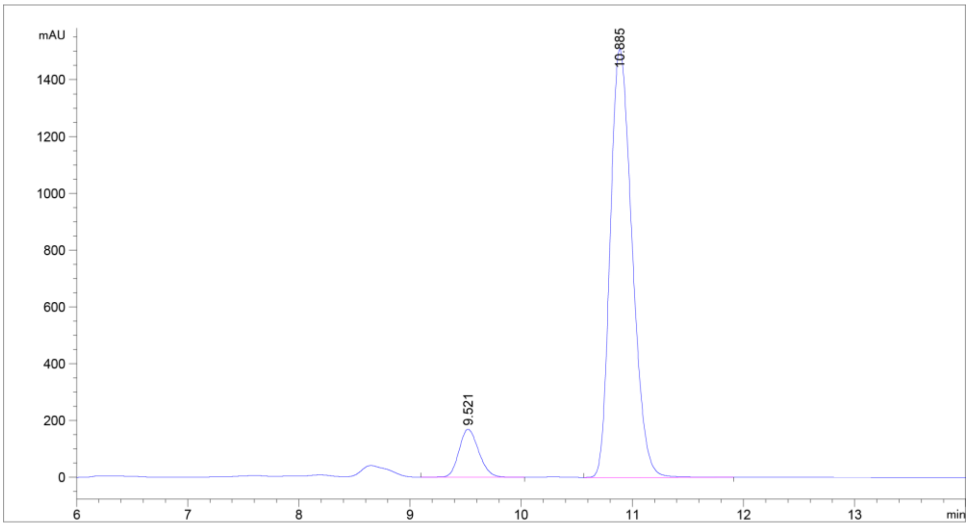

Peak #	RetTime [min]	Type	Width [min]	Area [mAU*s]	Height [mAU]	Area %
1	9.521	BB	0.1923	2093.76611	169.22714	9.2255
2	10.885	BB	0.2144	2.06017e4	1507.58203	90.7745
